# Neurochemical atlas of the cat spinal cord

**DOI:** 10.3389/fnana.2022.1034395

**Published:** 2022-10-19

**Authors:** Aleksandr Veshchitskii, Polina Shkorbatova, Natalia Merkulyeva

**Affiliations:** Neuromorphology Lab, Pavlov Institute of Physiology Russian Academy of Sciences, Saint Petersburg, Russia

**Keywords:** atlas, spinal cord, neurochemical labeling, neuronal populations, cat

## Abstract

The spinal cord is a complex heterogeneous structure, which provides multiple vital functions. The precise surgical access to the spinal regions of interest requires precise schemes for the spinal cord structure and the spatial relation between the spinal cord and the vertebrae. One way to obtain such information is a combined anatomical and morphological spinal cord atlas. One of the widely used models for the investigation of spinal cord functions is a cat. We create a single cell-resolution spinal cord atlas of the cat using a variety of neurochemical markers [antibodies to NeuN, choline acetyltransferase, calbindin 28 kDa, calretinin, parvalbumin, and non-phosphorylated heavy-chain neurofilaments (SMI-32 antibody)] allowing to visualize several spinal neuronal populations. In parallel, we present a map of the spatial relation between the spinal cord and the vertebrae for the entire length of the spinal cord.

## Introduction

The spinal cord is the most ancient part of the central nervous system, which is responsible for the autonomic control of the visceral organs and for the sensorimotor control of the muscle activity (Watson and Kayalioglu, 2009). Spinal cord injuries and diseases can be accompanied by corresponding violations or shutdowns of these functions (McDonald and Sadowsky, 2002). Treatments for the rehabilitation of these disorders are based on complex experimental data from neuromorphological, neurochemical, neurophysiological, and genetic studies.

Currently, rodents are the main experimental object in neurosciences (Ellenbroek and Youn, 2016). However, some features of rodents (e.g., small size) may be a possible source for particular limitation in using this model in neurophysiological studies. One of the limitations is increased requirement for fine manipulations during surgical procedures, which may lead to an additional increase in the sample of experimental objects. In this case, larger animals (e.g., cats, dogs, and mini-pigs) are more suitable for these experiments. Since the seminal studies of C.S. Sherrington and T.G. Brown, who elaborated the hypothesis for the spinal central pattern generators (Sherrington, 1910; Brown, 1911, 1913), the cat has become a widely used model for the investigation of spinal cord functions especially the locomotor control. Data obtained using this animal model allowed to discover a number of mechanisms of spinal cord and brainstem control for locomotion (Shik et al., 1969) and identify special triggering zones in spinal cord, epidural or subdural, stimulation of which induces coordinated locomotion (Iwahara et al., 1992; Musienko et al., 2009). Later, these data were incorporated into the neurorehabilitation approaches of spinal function recovery (Gerasimenko et al., 2018; Gill et al., 2018). The large size and high recoverability (Kuhtz-Buschbeck et al., 1996; Martinez et al., 2011) of the cat allows to use many invasive (e.g., implanted electromyographic and extracellular recording sensors) and non-invasive sensors and stimulating devices, and perform fine surgical procedures for the acute and chronic studies on both locomotor and visceral control (Musienko et al., 2012; Merkulyeva et al., 2019; Afanasenkau et al., 2020). The precise neurochemical mapping for spinal neuronal populations, combining with stereotaxic coordinates can be important for the design of a neurophysiological experiment and subsequent data interpretation. Currently, such spinal cord atlases were created for rodents (Watson et al., 2009, 2021; Sengul et al., 2012) and primates (Tokuno et al., 2011; Sengul et al., 2012; Watson et al., 2021) but not cats.

Two major classes of neurons can be distinguished within the spinal cord gray matter, i.e., motoneurones and interneurons. They can be visualized using specific neurochemical markers.

(1) Choline acetyltransferase (ChAT) is a specific marker for motoneuronal pools and neurons responsible for visceral control; ChAT catalyzes the biosynthesis of the neurotransmitter acetylcholine (Wu and Hersh, 2008) and is detected in the entire population of motoneurons (small, medium, and large-sized), in their fibers, and neuropil (Barber et al., 1984); ChAT is also found in structures responsible for the visceral control, i.e., intermediolateral nucleus (IML), intercalated nuclei (IC), intermediomedial nucleus (IMM), sacral parasympathetic nucleus (SPN), and dorsal gray commissure (DGC) (Barber et al., 1984; Phelps et al., 1984).
(2) Ca^2+^-binding proteins calbindin 28 kDa (CB), calretinin (CR), and parvalbumin (PV) have been used to label predominantly interneuronal subpopulations but also visceral neurons (Ren and Ruda, 1994). These proteins are more well-known members of Ca^2+^-binding proteins, a heterogeneous group of proteins that participate in numerous cellular functions (e.g., Ca^2+^ homeostasis and Ca^2+^ signaling pathways) (Yáñez et al., 2012).
  Calbindin labels Renshaw cells (Sanna et al., 1993; Alvarez et al., 1999; Stepien et al., 2010) and several visceral structures, e.g., IML (Zhang et al., 1990; Grkovic and Anderson, 1997; Moiseev et al., 2019), IMM (Zhang et al., 1990), IC (Porseva, 2015), DGC (Ren and Ruda, 1994; Grundy et al., 2019), SPN (Menétrey et al., 1992), and Onuf’s nucleus (Ince et al., 1993; Alexianu et al., 1994).
  It has been proposed that CR labels the population of excitatory interneurons located in the dorsal horns (Smith et al., 2019; Peirs et al., 2021). At the same time, CR is detected in all spinal laminae without clear preference and labels subpopulation of the small interneurons of the Clarke’s nuclei (CN) (Veshchitskii et al., 2021). CR also labels visceral neurons of the IML (Edwards et al., 1996) and DGC (Ren and Ruda, 1994; Merkulyeva et al., 2019).
  Parvalbumin is predominantly associated with the spinal proprioceptive system marking large-caliber afferent fibers and CN (Zhang et al., 1990; Clowry et al., 1997). PV is expressed in V1 interneuron population including Renshaw cells and Ia inhibitory interneurons (Alvarez et al., 2005; Siembab et al., 2010).
(3) SMI-32 antibody is a marker for non-phosphorylated heavy-chain neurofilaments (Sternberger and Sternberger, 1983). SMI-32 labels neurons with large soma and fast large-caliber axons (Bickford et al., 1998); in the spinal cord, these features are mainly inherent to motoneurons (Tsang et al., 2000) but also to some ascending tract neurons, e.g., large cells of the CN (the main origin of the dorsal spinocerebellar tract) (Tsang et al., 2000; Clowry et al., 2005; Merkul’eva et al., 2017).

To reveal the total population of the spinal neurons, a NeuN antibody was used. The main advantage of this protein is its absence in non-nervous tissues and in non-neuronal cells of the nervous tissue (Gusel’nikova and Korzhevskiy, 2015).

Therefore, the main aim of this study is to create a high-resolution neurochemical atlas of the cat spinal cord depicting multiple cellular populations throughout the entire spinal cord. This atlas can be used for the setting up of new neuroanatomical and neurophysiological experiments and for the interpretation of the already obtained neurophysiological data.

## Materials and methods

### Subjects

Experiments were performed in accordance with requirements of Council Directive 2010/63EU of the European Parliament on protection of animals used in experimental and other scientific purposes, and with the approval of the Ethics Commission of the Pavlov Institute of Physiology (Protocol #30/01/2020).

Spinal cord of the one intact adult domestic female cat (weighing 3.5 kg) was used for the creation of the images for the cat spinal cord atlas. Preliminary we verified all histological and immunohistochemical procedures with spinal cord, and all staining patterns using multiple animals (Merkulyeva et al., 2016, 2019; Shkorbatova et al., 2019; Veshchitskii et al., 2021). Immunohistochemical patterns are completely coincide with the present data.

According to the “3R” rule, the brain of the animal taken for the atlas was used for other studies dedicated to visual system development (Merkulyeva et al., 2018, 2020; Mikhalkin and Merkulyeva, 2021).

### Perfusion and dissection

The animal was deeply anesthetized with a mixture of Zoletil (Virbac, France; 20 mg/kg) and Xyla (Interchemie werken “De Adelaar” BV, Netherlands; 2 mg/kg), intramuscularly. Heparin (Endopharm, Russia; 0.5 ml/kg) was injected intramuscularly 10 min before the start of perfusion. Then the animal was transcardially perfused with 0.9% NaCl (2 liters, with Heparin, 0.5 ml/l) followed by 4% paraformaldehyde (2 L). Then the animal was placed in a prone position, the spinal cord was exposed by removing the vertebral arches and the dorsal part of the dura mater. The distances between the caudal-most parts of the dorsal rootlet attachment zones, connected to the neighboring dorsal root ganglia were measured as spinal cord segments (Shkorbatova et al., 2019). The lengths of all vertebrae and the positions of the segments in relation to the vertebrae were also thoroughly documented. Then, the spinal cord was cut to segments and each of them was replaced in 10, 20, and 30% sucrose, for cryoprotection, and thereafter cut into 50 μm transverse slices on a freezing microtome (Reichert, Austria). Storage of the slices was in 0.1 M PBS with 0.1% NaN_3_ at +4°C.

### Algorithm of choosing the slices for the atlas

**FIGURE 1:**
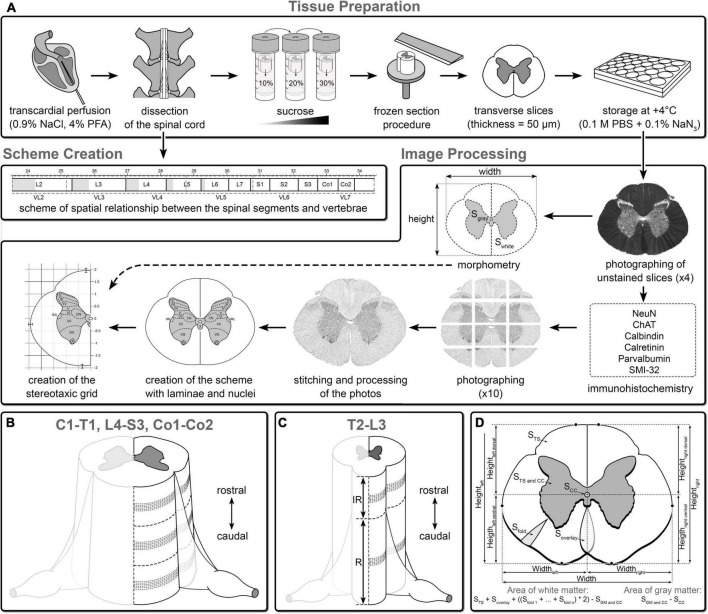
Overview of the steps of atlas creation. **(A)** Tissue preparation and image processing (see details in Section “Materials and methods”). Algorithm for selection of the slices for the atlas on the example segments L4 **(B)** and T13 **(C)** segments: IR, interrootlet part of the segment; R, rootlet part of the segment, dashed loci correspond to area from which slices were taken into immunohistochemical study. **(D)** Morphometry of the spinal cord. S TS, area of transverse slice of the segment; S GM and CC, area of gray matter and central canal; S CC, area of central canal; S fold, area of white matter fold; S overlay, area of superimposed zones of white matter; Height left, height of left half of the segment; Height right, height of right half of the segment; Height left dorsal, distance from the central canal to the highest point in the left dorsal quarter of the segment; Height left ventral, distance from the central canal to the highest point in the left ventral quarter of the segment; Height right dorsal, distance from the central canal to the highest point in the right dorsal quarter of the segment; Height right ventral, distance from the central canal to the highest point in the right ventral quarter of the segment; Width, width of the segment; Width left, distance from the central canal to the most lateral point of the left half of the segment; Width right, distance from the central canal to the most lateral point of the right half of the segment.

The spinal cord is subdivided into regions (i.e., C, T, L, S, and Co) and corresponding segments; however, some peculiarities of structural organization can be observed even within the segment (Vanderhorst and Holstege, 1997). Therefore, for all segments, 2–3 regions of interest were used for the atlas. For the more homogeneous segments (T2–L3), two regions of interest were used, i.e., from the rostral (interrootlet zone) and caudal (rootlet zone) parts. For highly variable segments (C1–T1 and L4–Co2) with weakly expressed or absent interrootlet zone, three equally spaced regions of interest were used (i.e., rostral, middle, and caudal). Six slices were chosen for immunohistochemical processing (Figures 1B,C).

### Immunohistochemical processing

Slices were processed as free floating. Between all procedures, the slices were washed in 0.01M PBS. Antigens were unmasked in 1% NaBH_4_, an endogenous peroxidase activity was blocked by 0.3% H_2_O_2_, unspecific immunoreactivity was lowered by incubation in 5% normal goat serum (NGS, Vector Labs). Thereafter slices were incubated for 70 h in primary antibody (Table 1) with 0.1% NaN_3_. Then the slices were incubated for 1 day in a biotinylated secondary antibody (Table 1) with 0.1% NaN_3_. Thereafter slices were subsequently processed using an avidin-biotin horseradish-peroxidase complex (ABC Elite system, Vector Laboratories) and diaminobenzidine (DAB)-NiCl-Í_2_Î_2_ reaction. After washing in distH_2_O, slices were mounted, dehydrated, cleared and placed under coverslips in Bio Mount HM (Bio-Optica Milano, Italy). To control the specificity of antibodies, we previously verified it using Western blot (Merkulyeva et al., 2022).

**TABLE 1:** The list of used antibodies (AB).

	Antibody	Host	Clonality	Dilution	Production	Lot	RRID
I-AB	Calbindin	Mouse	Monoclonal	1:3000	Sigma	C9848	AB_476894
	NeuN	Mouse	Monoclonal	1:5000	Sigma-Aldrich	MAB377	AB_2298772
	SMI-32	Mouse	Monoclonal	1:3000	BioLegend	SMI-32P	AB_2564642
	Calretinin	Rabbit	Polyclonal	1:40000	Sigma-Aldrich	AB5054	AB_2068506
	Parvalbumin	Rabbit	Polyclonal	1:3000	Abcam	ab11427	AB_298032
	Choline Acetyltransferase	Goat	Polyclonal	1:200	Sigma-Aldrich	AB144P	AB_2079751
II-AB	Anti-Rabbit IgG	Sheep		1:600	Vector Lab	BA-1000	AB_2313606
	Anti-Mouse IgG	Horse		1:600	Vector Lab	BA-2000	AB_2313581
	Anti-Goat IgG	Mouse		1:1000	Vector Lab	BA-9200	AB_2336171

### Antibody characterization

The antibody against NeuN (Sigma-Aldrich, Cat.# MAB377, RRID: AB_2298772) is a mouse monoclonal IgG1 recognizing 46 and 48 kDa bands on Western blots of a cat brain (Merkulyeva et al., 2022). The immunostaining of sections through the cat spinal cord (used at 1:5000) produced a pattern of NeuN labeling that was identical to previous descriptions (Veshchitskii et al., 2022), the total population of exclusively neuronal cells.

Calbindin antibody (Sigma, Cat.# C9848, RRID: AB_476894) is mouse monoclonal IgG1, recognizing s single 28 kDa band on Western blots of a cat brain (Merkulyeva et al., 2022). The immunostaining of sections through the spinal cord (used at 1:3000) produced a pattern of CB labeling that was identical to previous descriptions on cat spinal cord (Merkulyeva et al., 2016), specific neuronal bands in the dorsal horns and clusters of cells in intermediate gray matter of spinal enlargements. The general pattern of immunopositive neurons corresponds to the data on other species with using of other antibodies to calbindin (Ren and Ruda, 1994).

Calretinin antibody (Sigma-Aldrich, Cat.# AB5054, RRID: AB_2068506) is a rabbit polyclonal IgG recognizing 29 kDa band on Western blots of a cat brain (Merkulyeva et al., 2022). In the cat spinal cord CR is present by many immunopositive neurons throughout the gray matter in the same structures as in previous our works with this antibody (Veshchitskii et al., 2021) and works of other authors with other calretinin antibody (Anelli and Heckman, 2005).

Parvalbumin antibody (Abcam, Cat.# ab11427, RRID: AB_298032) is a rabbit polyclonal IgG recognizing 12 kDa band on Western blots of a cat brain (Merkulyeva et al., 2022). In the spinal cord as in other works PV is present in specific pre-motor neural populations and fibers related the proprioceptive system (Ren and Ruda, 1994; Anelli and Heckman, 2005).

ChAT antibody (Sigma-Aldrich, Cat.# AB144P, RRID: AB_2079751) is a goat polyclonal IgG recognizing 70/74 kDa band on Western blots of a mouse brain (manufacturer’s datasheet). No papers concerning these antibodies on cats were obtained. But data obtained using rodents illustrate the same results, with specific staining for motoneurons and visceral neurons (Mesnage et al., 2011).

SMI-32 antibody (BioLegend, Cat.# SMI-32P, RRID: AB_2564642) is a mouse monoclonal IgG1 to non-phosphorylated heavy-chain neurofilaments recognizing ∼200 kDa band on Western blots of a cat brain (Burnat et al., 2012). These neurofilaments are labeled in neurons with large soma (for example, motoneurons) (Tsang et al., 2000) and fast large-caliber axons (Bickford et al., 1998).

### Image processing

Digital images of the slices with the identified antigens were obtained using a computer setup equipped with an Olympus CX31 light microscope (Olympus Corporation, Japan; 4 × and 10 × objectives), digiCamControl software package distributed as an open source, and Nikon camera (D3200, Nikon Corporation, Japan). Image background processing was performed using the FlatBFv2 plugin in a free Fiji software package (Schindelin et al., 2012).

### Morphometry

The morphometric characteristics of the spinal cord were measured only in unstained wet slices, that lead to the only one step of tissue shrinkage—due to the fixation (Watson et al., 2009). As for the immunohistochemical processing, it led to the additional “shrinkage.” Therefore, it was not possible to obtain spinal cord statistical parameters that would be similar to those of an alive animal. Thus, for each region of interest for all segments, 5 adjacent transverse sections were used in morphometric analysis (a total of 420 slices). All slices were photographed at 4× magnification. Using the Fiji software package, the following morphometric parameters were calculated in these images, i.e., general area of transverse slice, area of gray matter with central canal, central canal area, area of white matter folds (formed as a result of different levels of “shrinkage” of white and gray matter during fixation), height and width of the spinal cord, distances from the central canal to the dorsal, as well as ventral and lateral borders of the white matter (Figure 1D). Based on these parameters, the area of gray and white matter was calculated according to the formulas presented in Figure 1D. The average areas of gray and white matter ± standard deviation (SD) and SD of distances from the central canal to the dorsal, ventral, and lateral borders of the white matter were added to the scheme of each segment.

## Results

### Spatial relationship between the spinal segments and vertebrae and morphometry

**FIGURE 2:**
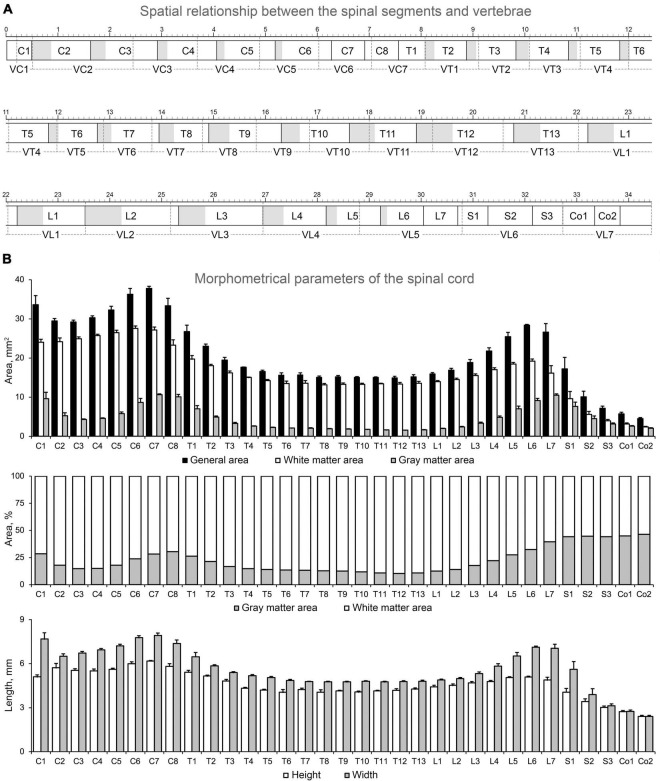
Spatial relationship between the spinal segments and vertebrae **(A)** and morphometric data about the spinal cord of the cat used **(B)**. Rectangles with black solid borders, segments; gray part, interrootlet zone; white part, rootlet zone; rectangles with dashed borders, vertebrae; the large-scale bar is 1 cm.

The first objective was to obtain a spatial relationship between the vertebrae (V) as the reference point and the spinal segment localized below it. This spatial relationship was obtained for all cervical (C), thoracic (T), lumbar (L), and sacral (S) segments and for the first and second coccygeal (Co) segments. The total length of the segments, their interrootlet and rootlet zones, and the vertebrae were also assessed. The obtained results and a schematic representation of the spatial relationship between the segments and the vertebrae for the cat used in the atlas are shown in Figure 2A and in Table 2.

**TABLE 2:** Lengths of the spinal segments and vertebrae.

Vertebrae	Length, mm	Segment	Length of IR, mm	Length of R, mm	Full length, mm
VC1	3.00	C1	–	4.80	4.80
VC2	19.40	C2	3.75	7.60	11.35
VC3	12.40	C3	2.90	10.00	12.90
VC4	12.00	C4	1.90	9.50	11.40
VC5	11.40	C5	1.50	9.80	11.30
VC6	10.20	C6	1.34	9.49	10.83
VC7	10.30	C7	–	6.43	6.43
–	–	C8	–	6.50	6.50
VT1	10.30	T1	–	5.11	5.11
VT2	9.80	T2	1.80	6.20	8.00
VT3	9.80	T3	1.87	7.67	9.54
VT4	9.30	T4	2.40	7.68	10.08
VT5	9.00	T5	1.68	8.24	9.92
VT6	9.30	T6	1.97	7.48	9.45
VT7	9.70	T7	2.70	9.20	11.90
VT8	10.40	T8	2.90	6.70	9.60
VT9	10.30	T9	4.00	10.00	14.00
VT10	11.50	T10	3.56	9.57	13.13
VT11	12.20	T11	4.90	8.00	12.90
VT12	13.60	T12	7.00	11.81	18.81
VT13	14.50	T13	5.08	9.19	14.27
VL1	15.00	L1	4.99	8.13	13.12
VL2	16.40	L2	7.00	10.95	17.95
VL3	17.80	L3	5.20	11.02	16.22
VL4	18.60	L4	4.03	8.17	12.20
VL5	19.80	L5	2.11	8.43	10.54
VL6	19.40	L6	1.26	7.08	8.34
VL7	17.10	L7	–	6.59	6.59
VS1		S1	–	5.82	5.82
VS2		S2	–	8.52	8.52
VS3		S3	–	5.89	5.89
		Co1	–	6.12	6.12
		Co2	–	4.91	4.91

IR, interrootlet zone of the segment; R, rootlet zone of the segment.

It is well-known that the positions of the spinal cord segments inside the vertebral canal do not correspond to the location of eponymous vertebrae owing to different rate of their growth during ontogeny (Maierl and Liebich, 1998), which is known as the “spinal cord ascension” (Streeter, 1919). However, ascension is a complicated process, accompanied by descension of some spinal cord regions during the formation of enlargements (Maierl and Liebich, 1998; Shkorbatova et al., 2022). Thus, in the cat, the C1 segment is not localized strictly in VC1 but extends rostrally to the occipital bone. The caudal part of C1 and entire C2 segments occupy VC1 and the rostral part of VC2 (the region of the odontoid process and atlantoaxial joint) (Figure 2A). The long segment C3 occupies almost all length of VC2 and a rostral third of VC3. Such shift (in one third) between segments and vertebrae position is maintained up to the C7 segment, which, owing to its short size, completely fits into the VC6. The last cervical vertebra (VC7) almost completely includes two segments at once, i.e., C8 and partly T1.

The VT1 contains the entire segment T2. Beginning with VT2, the following vertebrae and segments have similar sizes, and a stable shift of segments rostrally in relation to the eponymous vertebra by a half or a whole segment appears for mid-thoracic division. The gradual return of the segment to the eponymous vertebra as a result of an increase in the length of the segment relative to the length of the vertebra begins in T10 segment. The T11 segment is already mostly localized in VT11, and this pattern of localization is observed up to the L3 segment. Whereas the caudal parts of T12–L1 segments shift caudally in relation to their eponymous vertebrae, demonstrating a “descension”; L2 and L3 segments are localized in eponymous vertebrae. For the following segments, the rostral shift of the segment in relation to the eponymous vertebra appears, and it becomes stronger the more caudally the segment is located. Thus, the spinal cord of the cat ends in the lumbar vertebra. The L4 segment and the rostral half of the L5 segment are located in VL4; the caudal half of L5 segment, segments L6, L7, and the most rostral part of S1 segment share the length of VL5; the main part of S1 segment and the segments S2 and S3 are located in VL6, and the coccygeal segments are located in the VL7.

For all segments of the cat used in the analysis, several general morphometric parameters (i.e., general area of transverse slice, separate area of gray and white matter, height and width of the slice) were calculated (see details in Section “Morphometry”) and are shown in Table 3 and Figure 2B.

**TABLE 3:** Morphometric parameters of spinal segments.

Segment	General area, mm^2^	White matter area, mm^2^	Gray matter area, mm^2^	Height, mm	Width, mm
C1	33.62 ± 2.34	24.04 ± 0.72	9.58 ± 1.69	5.10 ± 0.14	7.68 ± 0.42
C2	29.45 ± 0.64	24.19 ± 0.91	5.26 ± 0.72	5.72 ± 0.30	6.51 ± 0.16
C3	29.19 ± 0.50	24.89 ± 0.49	4.30 ± 0.12	5.54 ± 0.12	6.72 ± 0.12
C4	30.27 ± 0.47	25.71 ± 0.39	4.56 ± 0.15	5.51 ± 0.13	6.95 ± 0.09
C5	32.28 ± 0.94	26.50 ± 0.58	5.78 ± 0.42	5.62 ± 0.07	7.21 ± 0.12
C6	36.24 ± 1.50	27.59 ± 0.63	8.66 ± 1.03	5.99 ± 0.13	7.76 ± 0.15
C7	37.77 ± 0.51	27.17 ± 0.72	10.61 ± 0.25	6.18 ± 0.04	7.92 ± 0.17
C8	33.37 ± 1.90	23.26 ± 1.37	10.11 ± 0.57	5.81 ± 0.17	7.38 ± 0.23
T1	26.72 ± 1.70	19.71 ± 0.89	7.00 ± 0.83	5.42 ± 0.12	6.47 ± 0.28
T2	22.98 ± 0.56	18.06 ± 0.33	4.92 ± 0.27	5.15 ± 0.06	5.84 ± 0.08
T3	19.47 ± 0.67	16.20 ± 0.47	3.27 ± 0.29	4.81 ± 0.12	5.39 ± 0.07
T4	17.61 ± 0.11	15.02 ± 0.13	2.60 ± 0.10	4.32 ± 0.06	5.18 ± 0.07
T5	16.55 ± 0.31	14.26 ± 0.28	2.29 ± 0.06	4.20 ± 0.04	5.05 ± 0.07
T6	15.61 ± 0.57	13.50 ± 0.60	2.11 ± 0.05	4.05 ± 0.20	4.85 ± 0.09
T7	15.66 ± 0.56	13.57 ± 0.64	2.08 ± 0.09	4.23 ± 0.11	4.78 ± 0.03
T8	15.03 ± 0.36	13.12 ± 0.40	1.91 ± 0.05	4.05 ± 0.16	4.76 ± 0.05
T9	15.17 ± 0.34	13.29 ± 0.35	1.88 ± 0.02	4.14 ± 0.04	4.76 ± 0.05
T10	15.05 ± 0.24	13.29 ± 0.24	1.76 ± 0.03	4.06 ± 0.07	4.80 ± 0.04
T11	15.02 ± 0.18	13.40 ± 0.19	1.62 ± 0.03	4.14 ± 0.04	4.75 ± 0.04
T12	14.91 ± 0.45	13.36 ± 0.39	1.55 ± 0.07	4.18 ± 0.11	4.78 ± 0.05
T13	15.18 ± 0.54	13.54 ± 0.47	1.64 ± 0.09	4.26 ± 0.08	4.80 ± 0.07
L1	15.93 ± 0.36	13.96 ± 0.27	1.97 ± 0.11	4.41 ± 0.12	4.89 ± 0.05
L2	16.91 ± 0.43	14.54 ± 0.32	2.37 ± 0.17	4.52 ± 0.10	4.98 ± 0.07
L3	18.85 ± 0.73	15.52 ± 0.46	3.34 ± 0.31	4.70 ± 0.09	5.32 ± 0.11
L4	21.82 ± 0.81	17.00 ± 0.49	4.82 ± 0.38	4.78 ± 0.08	5.84 ± 0.16
L5	25.45 ± 1.11	18.44 ± 0.46	7.01 ± 0.72	5.06 ± 0.05	6.53 ± 0.23
L6	28.39 ± 0.23	19.23 ± 0.52	9.16 ± 0.52	5.09 ± 0.04	7.12 ± 0.07
L7	26.65 ± 2.13	16.15 ± 1.91	10.49 ± 0.34	4.88 ± 0.19	7.04 ± 0.28
S1	17.24 ± 2.90	9.63 ± 1.80	7.61 ± 1.10	4.05 ± 0.27	5.61 ± 0.53
S2	10.06 ± 1.48	5.57 ± 0.78	4.49 ± 0.70	3.43 ± 0.18	3.90 ± 0.39
S3	7.15 ± 0.56	3.98 ± 0.30	3.16 ± 0.26	3.02 ± 0.10	3.13 ± 0.13
Co1	5.76 ± 0.37	3.17 ± 0.23	2.59 ± 0.14	2.72 ± 0.09	2.75 ± 0.09
Co2	4.48 ± 0.27	2.40 ± 0.17	2.08 ± 0.11	2.41 ± 0.06	2.41 ± 0.08

### Creation of the atlas page

**FIGURE 3:**
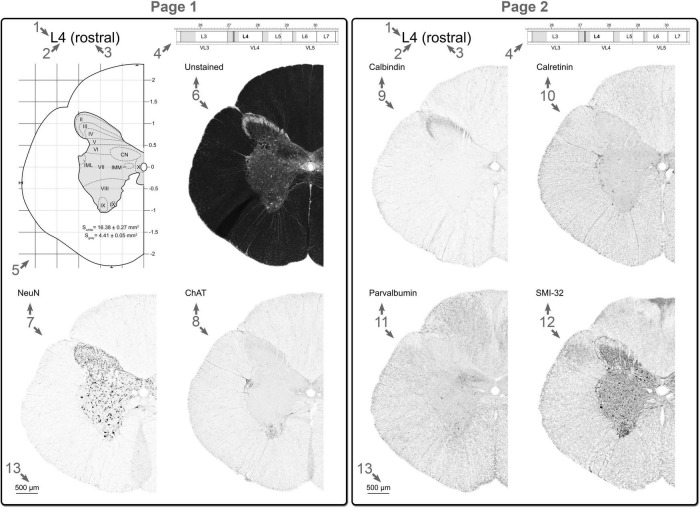
Example of the atlas pages for one region of interest of the one spinal segment: (1) the spinal cord region (C, cervical; T, thoracic; L, lumbar; S, sacral; Co, coccygeal); (2) the serial number of the segment; (3) the part of the segment (rostral, middle, caudal); (4) part of the scheme of the spatial interrelation between the segment and vertebra, region of interest is shown as the vertical grid; (5) stereotaxic grid and a schematic representation of the transverse slice with laminar and nuclear borders; (6) unstained slice; (7) NeuN stained slice; (8) ChAT stained slice; (9) calbindin stained slice; (10) calretinin stained slice; (11) parvalbumin stained slice; (12) SMI-32 stained slice; (13) Scale bar. See details in Section “Creation of the atlas page.”

All data obtained (immunohistochemical, morphometric, spatial relationship between the spinal segments and vertebrae) were grouped at two pages (Figure 3). Two-three regions of interest for every segment were illustrated.

Each page includes the following elements:

1. The spinal cord region (C—cervical, T—thoracic, L—lumbar, S—sacral, Co—coccygeal).
2. The serial number of the segment.
3. The part of the segment (rostral, middle, caudal).
4. Part of the scheme of the interrelation between the spatial position of the segment and vertebra, the slices of which are presented on the page, and the vertebrae in which this segment is localized, as well as the segments and vertebrae adjacent to it.
5. Stereotaxic grid and a schematic representation of the transverse slice and laminar and nuclear borders. The borders were defined on the basis of features of all markers used (see details in Section “Definition of laminae and nuclei in the grey matter of the cat spinal cord”). Region of interest is shown as the vertical grid.
6. Unstained—unstained slice.
7. NeuN—NeuN stained slice.
8. ChAT—choline acetyltransferase stained slice.
9. Calbindin—calbindin 28 kDa stained slice.
10. Calretinin—calretinin stained slice.
11. Parvalbumin—parvalbumin stained slice.
12. SMI-32—SMI-32 stained slice.
13. Scale bar corresponds to 500 μm.

### Definition of laminae and nuclei in the gray matter of the cat spinal cord

The gray matter of the spinal cord is divided into laminae and nuclei using several cytoarchitectonic features (Rexed, 1954). We modified the Rexed’s scheme for spinal laminae and nuclei definition using not only the total cytoarchitectonic features of the gray matter but also an additional neurochemical criteria originating from several immunohistochemical reactions. The key histological and neurochemical features distinguishing the laminae are presented below (methods that do not allow to see interlaminae or nuclear borders are not discussed). Summary data of neurochemical markers of the spinal cord laminae and nuclei in all segments is presented at Table 4 by color. All pages of the atlas are presented at Supplementary material.

**TABLE 4:** Markers of the spinal cord laminae and nuclei in all segments.

			Cervical								Thoracic													Lumbar							Sacral			Cog.	
			1	2	3	4	5	6	7	8	1	2	3	4	5	6	7	8	9	10	11	12	13	1	2	3	4	5	6	7	1	2	3	1	2
**Unstained**	**Laminae**	I	**+**	**+**	**+**	**+**	**+**	**+**	**+**	**+**	**+**	**+**	**±**	**±**	**±**	**±**	**±**	**±**	**±**	**±**	**±**	**±**	**±**	**±**	**±**	**±**	**+**	**+**	**+**	**+**	**+**	**+**	**+**	**+**	**+**
		II	**+**	**+**	**+**	**+**	**+**	**+**	**+**	**+**	**+**	**+**	**+**	**+**	**+**	**+**	**+**	**+**	**+**	**+**	**+**	**+**	**+**	**+**	**+**	**+**	**+**	**+**	**+**	**+**	**+**	**+**	**+**	**+**	**+**
		III	**+**	**+**	**+**	**±**	**±**	**–**	**–**	**–**	**–**	**–**	**–**	**–**	**–**	**–**	**–**	**–**	**–**	**–**	**–**	**–**	**–**	**–**	**–**	**–**	**–**	**–**	**–**	**–**	**–**	**±**	**±**	**+**	**+**
		IV	**+**	**+**	**+**	**±**	**±**	**–**	**–**	**–**	**–**	**–**	**–**	**–**	**–**	**–**	**–**	**–**	**–**	**–**	**–**	**–**	**–**	**–**	**–**	**–**	**–**	**–**	**–**	**–**	**–**	**±**	**±**	**+**	**+**
		V	**+**	**+**	**+**	**+**	**+**	**+**	**+**	**+**	**+**	**+**	**+**	**+**	**+**	**+**	**+**	**+**	**+**	**+**	**+**	**+**	**+**	**+**	**+**	**+**	**+**	**+**	**+**	**+**	**+**	**+**	**+**	**+**	**+**
		VI	**+**	**–**	**–**	**–**	**–**	**–**	**–**	**–**	**–**	**–**	**–**													**–**	**–**	**–**	**–**	**–**	**–**	**+**			
		VII	**–**	**–**	**–**	**–**	**–**	**–**	**–**	**–**	**–**	**–**	**–**	**–**	**–**	**–**	**–**	**–**	**–**	**–**	**–**	**–**	**–**	**–**	**–**	**–**	**–**	**–**	**–**	**–**	**–**	**–**	**–**	**–**	**–**
		VIII	**–**	**–**	**–**	**–**	**–**	**–**	**–**	**–**	**–**	**–**	**–**	**–**	**–**	**–**	**–**	**–**	**–**	**–**	**–**	**–**	**–**	**–**	**–**	**–**	**–**	**–**	**–**	**–**	**–**	**–**	**–**	**–**	**–**
		IX	**±**	**±**	**±**	**±**	**±**	**+**	**+**	**+**	**+**	**±**	**±**	**±**	**±**	**±**	**±**	**±**	**±**	**±**	**±**	**±**	**±**	**±**	**±**	**±**	**±**	**±**	**+**	**+**	**+**	**±**	**±**	**±**	**±**
		X	**–**	**–**	**–**	**–**	**–**	**–**	**–**	**–**	**–**	**–**	**–**	**–**	**–**	**–**	**–**	**–**	**–**	**–**	**–**	**–**	**–**	**–**	**–**	**–**	**–**	**–**	**–**	**–**	**–**	**–**	**–**	**–**	**–**
	**Nuclei**	CCN	**–**	**–**	**–**	**–**																													
		LCN	**+**	**+**	**+**																														
		CDN	**+**																																
		CoN	**+**	**+**	**+**	**+**																										**+**	**+**	**+**	**+**
		IMM	**–**	**–**	**–**	**–**	**–**	**–**	**–**	**–**	**–**	**–**	**–**	**–**	**–**	**–**	**–**	**–**	**–**	**–**	**–**	**–**	**–**	**–**	**–**	**–**	**–**	**–**	**–**	**–**	**–**	**–**	**–**	**–**	**–**
		IC								**–**	**–**	**–**	**–**	**–**	**–**	**–**	**–**	**–**	**–**	**–**	**–**	**–**	**–**	**–**	**–**	**–**	**–**								
		IML									**+**	**+**	**+**	**+**	**+**	**+**	**+**	**+**	**+**	**+**	**+**	**+**	**+**	**+**	**+**	**+**	**+**								
		CN											**+**	**+**	**+**	**+**	**+**	**+**	**+**	**+**	**+**	**+**	**+**	**+**	**+**	**+**	**+**								
		DGC																												**–**	**–**	**–**	**–**	**–**	**–**
		SPN																													**–**	**+**	**+**		
		ON																													**+**	**+**			
**NeuN**	**Laminae**	I	**+**	**+**	**+**	**+**	**+**	**+**	**+**	**+**	**+**	**+**	**+**	**±**	**±**	**±**	**±**	**±**	**±**	**±**	**±**	**±**	**±**	**±**	**±**	**±**	**+**	**+**	**+**	**+**	**+**	**+**	**+**	**+**	**+**
		II	**+**	**+**	**+**	**+**	**+**	**+**	**+**	**+**	**+**	**+**	**+**	**+**	**+**	**+**	**+**	**+**	**+**	**+**	**+**	**+**	**+**	**+**	**+**	**+**	**+**	**+**	**+**	**+**	**+**	**+**	**+**	**+**	**+**
		III	**+**	**+**	**+**	**+**	**+**	**+**	**+**	**+**	**+**	**+**	**+**	**+**	**+**	**+**	**+**	**+**	**+**	**+**	**+**	**+**	**+**	**+**	**+**	**+**	**+**	**+**	**+**	**+**	**+**	**+**	**+**	**+**	**+**
		IV	**+**	**+**	**+**	**+**	**+**	**+**	**+**	**+**	**+**	**+**	**+**	**+**	**+**	**+**	**+**	**+**	**+**	**+**	**+**	**+**	**+**	**+**	**+**	**+**	**+**	**+**	**+**	**+**	**+**	**+**	**+**	**+**	**+**
		V	**+**	**+**	**+**	**+**	**+**	**+**	**+**	**±**	**±**	**±**	**+**	**+**	**+**	**+**	**+**	**+**	**+**	**+**	**+**	**+**	**+**	**+**	**+**	**+**	**+**	**+**	**+**	**+**	**+**	**+**	**+**	**+**	**+**
		VI	**+**	**±**	**±**	**±**	**±**	**±**	**±**	**±**	**±**	**–**	**–**													**+**	**+**	**+**	**±**	**±**	**±**	**±**			
		VII	**±**	**±**	**±**	**±**	**±**	**±**	**±**	**±**	**±**	**±**	**±**	**±**	**±**	**±**	**±**	**±**	**±**	**±**	**±**	**±**	**±**	**±**	**±**	**±**	**±**	**+**	**±**	**±**	**±**	**±**	**±**	**±**	**±**
		VIII	**±**	**±**	**±**	**±**	**±**	**+**	**+**	**+**	**+**	**+**	**±**	**±**	**±**	**±**	**±**	**±**	**±**	**±**	**±**	**±**	**±**	**±**	**±**	**±**	**±**	**+**	**+**	**+**	**+**	**±**	**±**	**±**	**±**
		IX	**+**	**+**	**+**	**+**	**+**	**+**	**+**	**+**	**+**	**+**	**+**	**+**	**+**	**+**	**+**	**+**	**+**	**+**	**+**	**+**	**+**	**+**	**+**	**+**	**+**	**+**	**+**	**+**	**+**	**+**	**+**	**+**	**+**
		X	**+**	**+**	**+**	**+**	**+**	**+**	**+**	**+**	**+**	**+**	**+**	**+**	**+**	**+**	**+**	**+**	**+**	**+**	**+**	**+**	**+**	**+**	**+**	**+**	**+**	**+**	**+**	**+**	**+**	**+**	**+**	**+**	**+**
	**Nuclei**	CCN	**+**	**+**	**±**	**±**																													
		LCN	**+**	**+**	**+**																														
		CDN	**+**																																
		CoN	**+**	**+**	**+**	**+**																										**+**	**+**	**+**	**+**
		IMM	**–**	**–**	**–**	**–**	**–**	**–**	**–**	**–**	**–**	**–**	**–**	**–**	**–**	**–**	**–**	**–**	**–**	**–**	**–**	**–**	**–**	**–**	**–**	**–**	**–**	**–**	**–**	**–**	**–**	**–**	**–**	**–**	**–**
		IC								**–**	**–**	**–**	**–**	**–**	**–**	**–**	**–**	**–**	**–**	**–**	**–**	**–**	**–**	**–**	**–**	**–**	**–**								
		IML										**+**	**+**	**+**	**+**	**+**	**+**	**+**	**+**	**+**	**+**	**+**	**+**	**+**	**+**	**+**	**+**								
		CN											**±**	**±**	**±**	**±**	**±**	**±**	**±**	**±**	**±**	**±**	**±**	**±**	**±**	**±**	**±**								
		DGC																												**–**	**–**	**±**	**±**	**+**	**+**
		SPN																													**–**	**+**	**+**		
		ON																													**+**	**+**			
**ChAT**	**Laminae**	I	**–**	**–**	**–**	**–**	**–**	**–**	**–**	**–**	**–**	**–**	**–**	**–**	**–**	**–**	**–**	**–**	**–**	**–**	**–**	**–**	**–**	**–**	**–**	**–**	**–**	**–**	**–**	**–**	**–**	**–**	**–**	**–**	**–**
		II	**–**	**–**	**–**	**–**	**–**	**–**	**–**	**–**	**–**	**–**	**–**	**–**	**–**	**–**	**–**	**–**	**–**	**–**	**–**	**–**	**–**	**–**	**–**	**–**	**–**	**–**	**–**	**–**	**–**	**–**	**–**	**–**	**–**
		III	**–**	**–**	**–**	**–**	**–**	**–**	**–**	**–**	**–**	**–**	**–**	**–**	**–**	**–**	**–**	**–**	**–**	**–**	**–**	**–**	**–**	**–**	**–**	**–**	**–**	**–**	**–**	**–**	**–**	**–**	**–**	**–**	**–**
		IV	**–**	**–**	**–**	**–**	**–**	**–**	**–**	**–**	**–**	**–**	**–**	**–**	**–**	**–**	**–**	**–**	**–**	**–**	**–**	**–**	**–**	**–**	**–**	**–**	**–**	**–**	**–**	**–**	**–**	**–**	**–**	**–**	**–**
		V	**–**	**–**	**–**	**–**	**–**	**–**	**–**	**–**	**–**	**–**	**–**	**–**	**–**	**–**	**–**	**–**	**–**	**–**	**–**	**–**	**–**	**–**	**–**	**–**	**–**	**–**	**–**	**–**	**–**	**–**	**–**	**–**	**–**
		VI	**–**	**–**	**–**	**–**	**–**	**–**	**–**	**–**	**–**	**–**	**–**													**–**	**–**	**–**	**–**	**–**	**–**	**–**			
		VII	**–**	**–**	**–**	**–**	**–**	**–**	**–**	**–**	**–**	**–**	**–**	**–**	**–**	**–**	**–**	**–**	**–**	**–**	**–**	**–**	**–**	**–**	**–**	**–**	**–**	**–**	**–**	**–**	**–**	**–**	**–**	**–**	**–**
		VIII	**–**	**–**	**–**	**–**	**–**	**–**	**–**	**–**	**–**	**–**	**–**	**–**	**–**	**–**	**–**	**–**	**–**	**–**	**–**	**–**	**–**	**–**	**–**	**–**	**–**	**–**	**–**	**–**	**–**	**–**	**–**	**–**	**–**
		IX	**+**	**+**	**+**	**+**	**+**	**+**	**+**	**+**	**+**	**+**	**+**	**+**	**+**	**+**	**+**	**+**	**+**	**+**	**+**	**+**	**+**	**+**	**+**	**+**	**+**	**+**	**+**	**+**	**+**	**+**	**+**	**+**	**+**
		X	**–**	**–**	**–**	**–**	**–**	**–**	**–**	**–**	**–**	**–**	**–**	**–**	**–**	**–**	**–**	**–**	**–**	**–**	**–**	**–**	**–**	**–**	**–**	**–**	**–**	**–**	**–**	**–**	**–**	**–**	**–**	**–**	**–**
	**Nuclei**	CCN	**–**	**–**	**–**	**–**																													
		LCN	**–**	**–**	**–**																														
		CDN	**+**																																
		CoN	**+**	**+**	**+**	**+**																										**+**	**+**	**+**	**+**
		IMM	**+**	**+**	**+**	**+**	**+**	**+**	**+**	**+**	**+**	**+**	**+**	**+**	**+**	**+**	**+**	**+**	**+**	**+**	**+**	**+**	**+**	**+**	**+**	**+**	**+**	**+**	**+**	**+**	**+**	**+**	**+**	**+**	**+**
		IC								**±**	**±**	**±**	**–**	**–**	**±**	**±**	**±**	**±**	**±**	**±**	**±**	**±**	**±**	**–**	**–**	**±**	**±**								
		IML									**+**	**+**	**+**	**+**	**+**	**+**	**+**	**+**	**+**	**+**	**+**	**+**	**+**	**+**	**+**	**+**	**+**								
		CN											**–**	**–**	**–**	**–**	**–**	**–**	**–**	**–**	**–**	**–**	**–**	**–**	**–**	**–**	**–**								
		DGC																												**–**	**–**	**–**	**–**	**–**	**–**
		SPN																													**+**	**+**	**–**		
		ON																													**+**	**+**			
**Calbindin**	**Laminae**	I	**±**	**±**	**±**	**±**	**±**	**±**	**±**	**±**	**±**	**±**	**±**	**±**	**±**	**±**	**±**	**±**	**±**	**±**	**±**	**±**	**±**	**±**	**±**	**±**	**±**	**+**	**±**	**±**	**±**	**±**	**±**	**±**	**±**
		II	**+**	**+**	**+**	**+**	**+**	**+**	**+**	**+**	**+**	**+**	**+**	**+**	**+**	**+**	**+**	**+**	**+**	**+**	**+**	**+**	**+**	**+**	**+**	**+**	**+**	**+**	**+**	**+**	**+**	**+**	**+**	**+**	**+**
		III	**±**	**±**	**±**	**±**	**±**	**±**	**±**	**±**	**±**	**±**	**±**	**±**	**±**	**±**	**±**	**±**	**±**	**±**	**±**	**±**	**±**	**±**	**±**	**±**	**±**	**+**	**±**	**±**	**±**	**±**	**±**	**±**	**±**
		IV	**–**	**–**	**–**	**–**	**–**	**–**	**–**	**–**	**–**	**–**	**–**	**–**	**–**	**–**	**–**	**–**	**–**	**–**	**–**	**–**	**–**	**–**	**–**	**–**	**–**	**–**	**–**	**–**	**–**	**–**	**–**	**–**	**–**
		V	**–**	**–**	**–**	**–**	**–**	**–**	**–**	**–**	**–**	**–**	**–**	**–**	**–**	**–**	**–**	**–**	**–**	**–**	**–**	**–**	**–**	**–**	**–**	**–**	**–**	**–**	**–**	**–**	**–**	**–**	**–**	**–**	**–**
		VI	**–**	**–**	**–**	**–**	**–**	**–**	**–**	**–**	**–**	**–**	**–**													**–**	**–**	**–**	**–**	**–**	**–**	**–**			
		VII	**–**	**–**	**–**	**–**	**–**	**–**	**–**	**–**	**–**	**–**	**–**	**–**	**–**	**–**	**–**	**–**	**–**	**–**	**–**	**–**	**–**	**–**	**–**	**–**	**–**	**–**	**–**	**–**	**–**	**–**	**–**	**–**	**–**
		VIII	**–**	**–**	**–**	**–**	**–**	**–**	**–**	**–**	**–**	**–**	**–**	**–**	**–**	**–**	**–**	**–**	**–**	**–**	**–**	**–**	**–**	**–**	**–**	**–**	**–**	**–**	**–**	**–**	**–**	**–**	**–**	**–**	**–**
		IX	**–**	**–**	**–**	**–**	**–**	**–**	**–**	**–**	**–**	**–**	**–**	**–**	**–**	**–**	**–**	**–**	**–**	**–**	**–**	**–**	**–**	**–**	**–**	**–**	**–**	**–**	**–**	**–**	**–**	**–**	**–**	**–**	**–**
		X	**–**	**–**	**–**	**–**	**–**	**–**	**–**	**–**	**–**	**–**	**–**	**–**	**–**	**–**	**–**	**–**	**–**	**–**	**–**	**–**	**–**	**–**	**–**	**–**	**–**	**–**	**–**	**–**	**–**	**–**	**–**	**–**	**–**
**Calbindin**	**Nuclei**	CCN	**–**	**–**	**–**	**–**																													
		LCN	**–**	**–**	**–**																														
		CDN	**–**																																
		CoN	**–**	**–**	**–**	**–**																										**–**	**–**	**–**	**–**
		IMM	**–**	**–**	**–**	**–**	**–**	**–**	**–**	**–**	**–**	**–**	**–**	**–**	**–**	**–**	**–**	**–**	**–**	**–**	**–**	**–**	**–**	**–**	**–**	**–**	**–**	**–**	**–**	**–**	**–**	**–**	**–**	**–**	**–**
		IC								**–**	**–**	**–**	**–**	**–**	**–**	**–**	**–**	**–**	**–**	**–**	**–**	**–**	**–**	**–**	**–**	**–**	**–**								
		IML									**–**	**–**	**–**	**–**	**–**	**–**	**–**	**–**	**–**	**–**	**–**	**–**	**–**	**–**	**–**	**–**	**–**								
		CN											**–**	**–**	**–**	**–**	**–**	**–**	**–**	**–**	**–**	**–**	**–**	**–**	**–**	**–**	**–**								
		DGC																												**–**	**–**	**–**	**–**	**–**	**–**
		SPN																													**–**	**–**	**–**		
		ON																													**–**	**–**			
**Calretinin**	**Laminae**	I	**–**	**–**	**–**	**–**	**–**	**–**	**–**	**–**	**–**	**–**	**–**	**–**	**–**	**–**	**–**	**–**	**–**	**–**	**–**	**–**	**–**	**–**	**–**	**–**	**–**	**–**	**–**	**–**	**–**	**–**	**–**	**–**	**–**
		II	**–**	**–**	**–**	**–**	**–**	**–**	**–**	**–**	**–**	**–**	**–**	**–**	**–**	**–**	**–**	**–**	**–**	**–**	**–**	**–**	**–**	**–**	**–**	**–**	**–**	**–**	**–**	**–**	**–**	**–**	**–**	**–**	**–**
		III	**–**	**–**	**–**	**–**	**–**	**–**	**–**	**–**	**–**	**–**	**–**	**–**	**–**	**–**	**–**	**–**	**–**	**–**	**–**	**–**	**–**	**–**	**–**	**–**	**–**	**–**	**–**	**–**	**–**	**–**	**–**	**–**	**–**
		IV	**–**	**–**	**–**	**–**	**–**	**–**	**–**	**–**	**–**	**–**	**–**	**–**	**–**	**–**	**–**	**–**	**–**	**–**	**–**	**–**	**–**	**–**	**–**	**–**	**–**	**–**	**–**	**–**	**–**	**–**	**–**	**–**	**–**
		V	**–**	**–**	**–**	**–**	**–**	**–**	**–**	**–**	**–**	**–**	**–**	**–**	**–**	**–**	**–**	**–**	**–**	**–**	**–**	**–**	**–**	**–**	**–**	**–**	**–**	**–**	**–**	**–**	**–**	**–**	**–**	**–**	**–**
		VI	**–**	**–**	**–**	**–**	**–**	**–**	**–**	**–**	**–**	**–**	**–**													**–**	**–**	**–**	**–**	**–**	**–**	**–**			
		VII	**–**	**–**	**–**	**–**	**–**	**–**	**–**	**–**	**–**	**–**	**–**	**–**	**–**	**–**	**–**	**–**	**–**	**–**	**–**	**–**	**–**	**–**	**–**	**–**	**–**	**–**	**–**	**–**	**–**	**–**	**–**	**–**	**–**
		VIII	**–**	**–**	**–**	**–**	**–**	**–**	**–**	**–**	**–**	**–**	**–**	**–**	**–**	**–**	**–**	**–**	**–**	**–**	**–**	**–**	**–**	**–**	**–**	**–**	**–**	**–**	**–**	**–**	**–**	**–**	**–**	**–**	**–**
		IX	**–**	**–**	**–**	**–**	**–**	**–**	**–**	**–**	**–**	**–**	**–**	**–**	**–**	**–**	**–**	**–**	**–**	**–**	**–**	**–**	**–**	**–**	**–**	**–**	**–**	**–**	**–**	**–**	**–**	**–**	**–**	**–**	**–**
		X	**–**	**–**	**–**	**–**	**–**	**–**	**–**	**–**	**–**	**–**	**–**	**–**	**–**	**–**	**–**	**–**	**–**	**–**	**–**	**–**	**–**	**–**	**–**	**–**	**–**	**–**	**–**	**–**	**–**	**–**	**–**	**–**	**–**
	**Nuclei**	CCN	**–**	**–**	**–**	**–**																													
		LCN	**+**	**+**	**+**																														
		CDN	**–**																																
		CoN	**–**	**–**	**–**	**–**																										**–**	**–**	**–**	**–**
		IMM	**–**	**–**	**–**	**–**	**–**	**–**	**–**	**–**	**–**	**–**	**–**	**–**	**–**	**–**	**–**	**–**	**–**	**–**	**–**	**–**	**–**	**–**	**–**	**–**	**–**	**–**	**–**	**–**	**–**	**–**	**–**	**–**	**–**
		IC								**–**	**–**	**–**	**–**	**–**	**–**	**–**	**–**	**–**	**–**	**–**	**–**	**–**	**–**	**–**	**–**	**±**	**±**								
		IML									**±**	**+**	**+**	**+**	**+**	**+**	**+**	**+**	**+**	**+**	**+**	**+**	**+**	**+**	**+**	**+**	**+**								
		CN											**–**	**–**	**–**	**–**	**–**	**–**	**–**	**–**	**–**	**–**	**–**	**–**	**–**	**–**	**–**								
		DGC																												**±**	**±**	**±**	**±**	**±**	**±**
		SPN																													**–**	**–**	**–**		
		ON																													**–**	**–**			
**Parvalbumin**	**Laminae**	I	**–**	**–**	**–**	**–**	**–**	**–**	**–**	**–**	**–**	**–**	**–**	**–**	**–**	**–**	**–**	**–**	**–**	**–**	**–**	**–**	**–**	**–**	**–**	**–**	**–**	**–**	**–**	**–**	**–**	**–**	**–**	**–**	**–**
		II	**–**	**–**	**–**	**–**	**–**	**–**	**–**	**–**	**–**	**–**	**–**	**–**	**–**	**–**	**–**	**–**	**–**	**–**	**–**	**–**	**–**	**–**	**–**	**–**	**–**	**–**	**–**	**–**	**–**	**–**	**–**	**–**	**–**
		III	**–**	**–**	**–**	**–**	**–**	**–**	**–**	**–**	**–**	**–**	**–**	**–**	**–**	**–**	**–**	**–**	**–**	**–**	**–**	**–**	**–**	**–**	**–**	**–**	**–**	**–**	**–**	**–**	**–**	**–**	**–**	**–**	**–**
		IV	**–**	**–**	**–**	**–**	**–**	**–**	**–**	**–**	**–**	**–**	**–**	**–**	**–**	**–**	**–**	**–**	**–**	**–**	**–**	**–**	**–**	**–**	**–**	**–**	**–**	**–**	**–**	**–**	**–**	**–**	**–**	**–**	**–**
		V	**–**	**–**	**–**	**–**	**–**	**–**	**–**	**–**	**–**	**–**	**–**	**–**	**–**	**–**	**–**	**–**	**–**	**–**	**–**	**–**	**–**	**–**	**–**	**–**	**–**	**–**	**–**	**–**	**–**	**–**	**–**	**–**	**–**
		VI	**–**	**–**	**–**	**–**	**–**	**–**	**–**	**–**	**–**	**–**	**–**													**–**	**–**	**–**	**–**	**–**	**–**	**–**			
		VII	**–**	**–**	**–**	**–**	**–**	**–**	**–**	**–**	**–**	**–**	**–**	**–**	**–**	**–**	**–**	**–**	**–**	**–**	**–**	**–**	**–**	**–**	**–**	**–**	**–**	**–**	**–**	**–**	**–**	**–**	**–**	**–**	**–**
		VIII	**–**	**–**	**–**	**–**	**–**	**–**	**–**	**–**	**–**	**–**	**–**	**–**	**–**	**–**	**–**	**–**	**–**	**–**	**–**	**–**	**–**	**–**	**–**	**–**	**–**	**–**	**–**	**–**	**–**	**–**	**–**	**–**	**–**
		IX	**–**	**–**	**–**	**–**	**–**	**–**	**–**	**–**	**–**	**–**	**–**	**–**	**–**	**–**	**–**	**–**	**–**	**–**	**–**	**–**	**–**	**–**	**–**	**–**	**–**	**–**	**–**	**–**	**–**	**–**	**–**	**–**	**–**
		X	**–**	**–**	**–**	**–**	**–**	**–**	**–**	**–**	**–**	**–**	**–**	**–**	**–**	**–**	**–**	**–**	**–**	**–**	**–**	**–**	**–**	**–**	**–**	**–**	**–**	**–**	**–**	**–**	**–**	**–**	**–**	**–**	**–**
	**Nuclei**	CCN	**–**	**–**	**–**	**–**																													
		LCN	**+**	**+**	**+**																														
		CDN	**–**																																
		CoN	**–**	**–**	**–**	**–**																										**–**	**–**	**–**	**–**
		IMM	**–**	**–**	**–**	**–**	**–**	**–**	**–**	**–**	**–**	**–**	**–**	**–**	**–**	**–**	**–**	**–**	**–**	**–**	**–**	**–**	**–**	**–**	**–**	**–**	**–**	**–**	**–**	**–**	**–**	**–**	**–**	**–**	**–**
		IC								**–**	**–**	**–**	**–**	**–**	**–**	**–**	**–**	**–**	**–**	**–**	**–**	**–**	**–**	**–**	**–**	**–**	**–**								
		IML									**–**	**–**	**–**	**–**	**–**	**–**	**–**	**–**	**–**	**–**	**–**	**–**	**–**	**–**	**–**	**–**	**–**								
		CN											**+**	**+**	**+**	**+**	**+**	**+**	**+**	**+**	**+**	**+**	**+**	**+**	**+**	**+**	**+**								
		DGC																												**–**	**–**	**–**	**–**	**–**	**–**
		SPN																													**–**	**–**	**–**		
		ON																													**–**	**–**			
**SMI-32**	**Laminae**	I	**+**	**+**	**+**	**+**	**+**	**+**	**+**	**+**	**+**	**+**	**+**	**+**	**+**	**+**	**+**	**+**	**+**	**+**	**+**	**+**	**+**	**+**	**+**	**+**	**+**	**+**	**+**	**+**	**+**	**+**	**+**	**+**	**+**
		II	**+**	**+**	**+**	**+**	**+**	**+**	**+**	**+**	**+**	**+**	**+**	**+**	**+**	**+**	**+**	**+**	**+**	**+**	**+**	**+**	**+**	**+**	**+**	**+**	**+**	**+**	**+**	**+**	**+**	**+**	**+**	**+**	**+**
		III	**+**	**+**	**±**	**±**	**–**	**±**	**±**	**±**	**±**	**±**	**±**	**–**	**–**	**–**	**–**	**–**	**–**	**–**	**–**	**–**	**–**	**–**	**–**	**–**	**–**	**–**	**–**	**–**	**–**	**–**	**–**	**–**	**–**
		IV	**+**	**+**	**±**	**±**	**–**	**±**	**±**	**±**	**±**	**±**	**±**	**–**	**–**	**–**	**–**	**–**	**–**	**–**	**–**	**–**	**–**	**–**	**–**	**–**	**–**	**–**	**–**	**–**	**–**	**–**	**–**	**–**	**–**
		V	**+**	**+**	**+**	**+**	**+**	**±**	**±**	**±**	**±**	**±**	**+**	**+**	**+**	**+**	**+**	**+**	**+**	**+**	**+**	**+**	**+**	**+**	**+**	**+**	**+**	**+**	**+**	**+**	**+**	**+**	**+**	**+**	**+**
		VI	**–**	**–**	**–**	**–**	**–**	**–**	**–**	**–**	**–**	**–**	**–**													**–**	**–**	**–**	**–**	**–**	**–**	**–**			
		VII	**–**	**–**	**–**	**–**	**–**	**–**	**–**	**–**	**–**	**–**	**–**	**–**	**–**	**–**	**–**	**–**	**–**	**–**	**–**	**–**	**–**	**–**	**–**	**–**	**–**	**–**	**–**	**–**	**–**	**–**	**–**	**–**	**–**
		VIII	**–**	**–**	**–**	**–**	**–**	**–**	**–**	**–**	**–**	**–**	**–**	**–**	**–**	**–**	**–**	**–**	**–**	**–**	**–**	**–**	**–**	**–**	**–**	**–**	**–**	**–**	**–**	**–**	**–**	**–**	**–**	**–**	**–**
		IX	**+**	**+**	**+**	**+**	**+**	**+**	**+**	**+**	**+**	**+**	**+**	**+**	**+**	**+**	**+**	**+**	**+**	**+**	**+**	**+**	**+**	**+**	**+**	**+**	**+**	**+**	**+**	**+**	**+**	**+**	**+**	**+**	**+**
		X	**–**	**–**	**–**	**–**	**–**	**–**	**–**	**–**	**–**	**–**	**–**	**–**	**–**	**–**	**–**	**–**	**–**	**–**	**–**	**–**	**–**	**–**	**–**	**–**	**–**	**–**	**–**	**–**	**–**	**–**	**–**	**–**	**–**
	**Nuclei**	CCN	**–**	**–**	**–**	**–**																													
		LCN	**+**	**+**	**+**																														
		CDN	**+**																																
		CoN	**+**	**+**	**+**	**+**																										**+**	**+**	**+**	**+**
		IMM	**–**	**–**	**–**	**–**	**–**	**–**	**–**	**–**	**–**	**–**	**–**	**–**	**–**	**–**	**–**	**–**	**–**	**–**	**–**	**–**	**–**	**–**	**–**	**–**	**–**	**–**	**–**	**–**	**–**	**–**	**–**	**–**	**–**
		IC								**–**	**–**	**–**	**–**	**–**	**–**	**–**	**–**	**–**	**–**	**–**	**–**	**–**	**–**	**–**	**–**	**–**	**–**								
		IML									**–**	**±**	**±**	**±**	**±**	**±**	**±**	**±**	**±**	**±**	**±**	**±**	**±**	**±**	**±**	**±**	**±**								
		CN											**±**	**±**	**±**	**±**	**±**	**±**	**±**	**±**	**±**	**±**	**±**	**±**	**±**	**±**	**±**								
		DGC																												**–**	**–**	**–**	**–**	**–**	**–**
		SPN																													**–**	**–**	**–**		
		ON																													**+**	**+**			

Cog, coccygeal; CCN, Central Cervical Nucleus; CN, Clarke’s Nucleus; CDN, Nucleus Centrodorsalis; CoN, Nucleus Commissuralis; DGC, Dorsal Gray Commissure; IC, Intercalated Nucleus; IMM, Intermediomedial Nucleus; IML, Intermediolateral Nucleus; LCN, Lateral Cervical Nucleus; ON, Onuf’s Nucleus; SPN, Sacral Parasympathetic Nucleus; “–”, doesn’t mark, “±”, marks poorly, “+”, marks well.

#### Lamina I

##### Unstained

**FIGURE 4:**
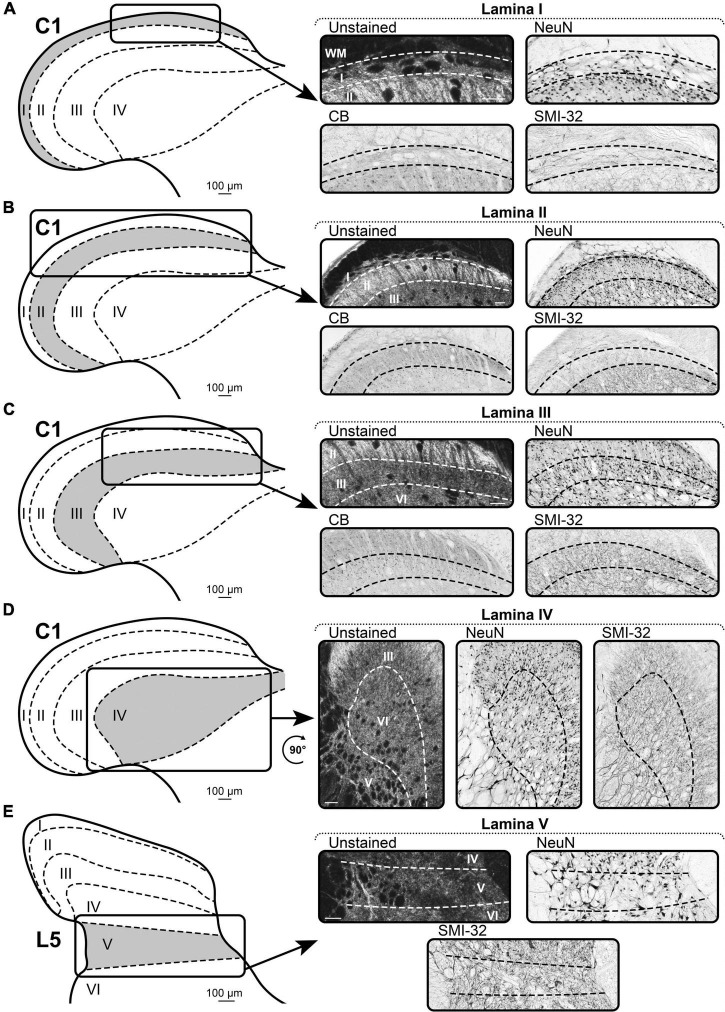
A representative example of the markers of Rexed’s laminae I–V of spinal cord. **(A–D)** Spinal segment C1. **(E)** Spinal segment L5. **(A)** Laminae I. **(B)** Laminae II. **(C)** Laminae III. **(D)** Laminae IV. **(E)** Laminae V. Scale bar is 100 μm.

In all segments, lamina I is clearly distinguishable from the white matter (by brightness) and lamina II (by its reticular structure) (Figure 4A). The lateral border of lamina I has a less reticular appearance; thus, it is more difficult to distinguish it from lamina II. In addition, the definition of the laminae I/II border significantly depends on the segment and, accordingly, the thickness of lamina I. It is well-distinguishable in segments C1–T2 and L4–Co2. In segments T3–L3, lamina I is too thin to be clearly separated from lamina II. In the sacral segments, lamina I is well-visualized by a dark strip of fibers parallel to curvature of the dorsal border of lamina II. Sometimes, these fibers are visible in the coccygeal segments.

##### NeuN

Lamina I is well-visualized by the distinct type of NeuN-immunopositive neurons, which are characterized by larger sizes and lower density, compared to the neurons of the underlying lamina II (Figure 4A).

##### Calbindin

In all segments, the dorsal border of lamina I is visualized by CB-immunopositive neuropil (Figure 4A). The ventral border is defined by fusiform neurons of lamina II. Lamina I contains rare CB-immunopositive neurons, which are clearly distinguishable in size from the smaller neurons of the underlying lamina II.

##### SMI-32

In all segments, lamina I is defined by bundles of SMI-32-immunopositive fibers following along the curvature of the dorsal horn (Figure 4A).

#### Lamina II

##### Unstained

In unstained slices, lamina II is represented by a light stripe following the contour of the dorsal horn (Figure 4B). It differs from lamina I by the absence of a reticular structure, and from lamina III by a much lighter appearance.

##### NeuN

NeuN labeling allows to define two layers of lamina II, i.e., the outer layer (dorsal) and the inner layer (ventral) (Figure 4B). These two layers differ in the density of the NeuN-immunopositive neurons; the outer layer has a higher density than the inner layer. The entire lamina II is distinguished from laminae I and III by the size of the NeuN-immunopositive neurons, which are smaller than those in adjacent laminae.

##### Calbindin

Lamina II can be distinguished from lamina III by brightly colored CB-immunopositive neuropil and from lamina I by smaller and more frequent CB-immunopositive neurons (Figure 4B).

##### SMI-32

Lamina II is well-visualized as a light strip of tissue with a lower density of SMI-32-immunopositive fibers than in adjacent laminae I and III (Figure 4B).

#### Lamina III

##### Unstained

In unstained slices of all segments, only the dorsal border of lamina III can be easily determined owing to the well-defined light lamina II (Figure 4C). The ventral border can be identified only in the most rostral (C1–C3) and caudal segments (S2–Co2), where lamina III is separated from lamina IV by the absence of the reticular structure of lamina IV. In other segments, laminae III and IV are indistinguishable in unstained slices.

##### NeuN

Lamina III differs from adjacent laminae by the type of NeuN-immunopositive neurons, i.e., they are larger than in lamina II and smaller than in lamina IV (Figure 4C).

##### Calbindin

The dorsal border of lamina III is clearly defined by the absence of CB-immunopositive neuropil, which is a feature of lamina II (Figure 4C). Lamina III itself contains small CB-immunopositive neurons, which are non-homogeneously distributed over the lamina; this feature does not allow to identify the distinct laminae III/IV border.

##### SMI-32

The dorsal border of lamina III is clearly visible owing to the sharp difference in the density of SMI-32-immunopositive fibers between laminae II and III (Figure 4C). The ventral border with lamina IV can be seen only in the rostral segments of the spinal cord (cervical and partially thoracic). In these segments, a higher density of SMI-32-immunopositive fibers can be seen in lamina III than in lamina IV. In other segments, laminae III and IV are indistinguishable.

#### Lamina IV

##### Unstained

In unstained slices of all segments, only the ventral border of lamina IV is well-defined by the presence of strong reticularity of the lateral part of lamina V (Figure 4D). The dorsal border of lamina IV can be defined only in the most rostral (C1–C3) and caudal segments (S2–Co2), where lamina IV is separated from lamina III by the presence of a small reticular structure.

##### NeuN

Lamina IV differs from lamina III by larger NeuN-immunopositive neurons and from lamina V by a lower density of NeuN-immunopositive neurons (Figure 4D).

##### SMI-32

The ventral border of lamina IV is defined by the presence of strong reticularity of the lateral part of lamina V (Figure 4D). The dorsal border with lamina III can be seen only in the rostral segments of the spinal cord (cervical and partially thoracic). In these segments, a lower density of SMI-32-immunopositive fibers can be seen in lamina IV than in lamina III. In other segments, laminae III and IV are indistinguishable.

#### Lamina V

##### Unstained

Lamina V differs from adjacent laminae by the reticular structure of its lateral part, which is clearly visible in unstained slices (Figure 4E).

##### NeuN

Lamina V differs from adjacent laminae by the reticular structure of its lateral part, which is clearly visible after NeuN labeling (Figure 4E).

##### SMI-32

Lamina V differs from adjacent laminae by the reticular structure of its lateral part, which is clearly visible after SMI-32 labeling, and sometimes by noticeable direction of the SMI-32 fibers running horizontally in the slice (Figure 4E).

#### Lamina VI

##### NeuN

**FIGURE 5:**
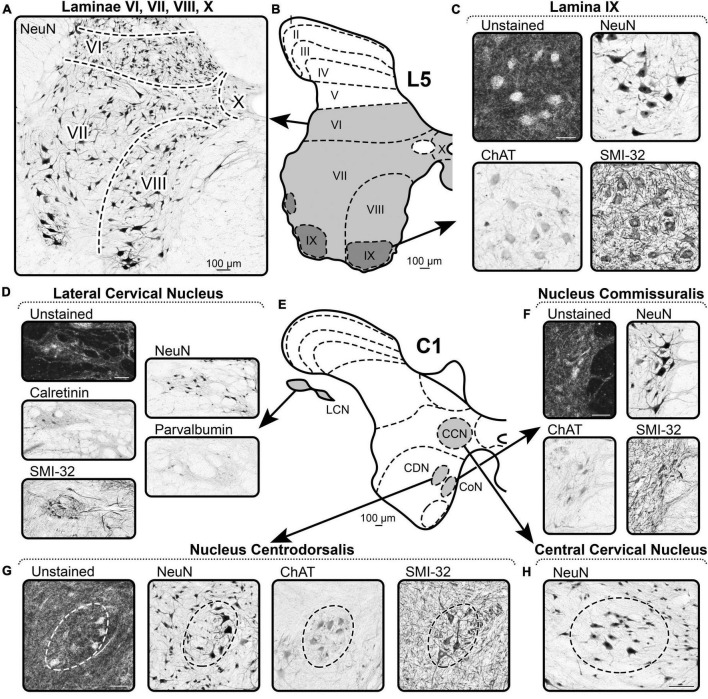
A representative example of the markers of Rexed’s laminae VI–X and nuclei of spinal cord. **(A–C)** Spinal segment L5. **(D–H)** Spinal segment C1. **(A)** NeuN labeling in laminae VI, VII, VIII, and X. **(B)** Scheme of L5 segment. **(C)** Distribution of the markers in lamina IX. **(D)** Distribution of the markers in the Lateral Cervical Nucleus. **(E)** Scheme of C1 segment. **(F)** Distribution of the markers in the Nucleus Commissuralis. **(G)** Distribution of the markers in the Nucleus Centrodorsalis. **(H)** Distribution of the markers in the Central Cervical Nucleus. Scale bar is 100 μm.

Lamina VI differs from lamina V by the absence of a reticular structure of the lateral part as well as a greater density of NeuN-immunopositive neurons (Figures 5A,B). Lamina VI differs from lamina VII by the smaller size of NeuN-immunopositive neurons.

#### Lamina VII

##### NeuN

Borders of lamina VII are defined by lower density of NeuN-immunopositive neurons than in adjacent laminae (Figures 5A,B).

#### Lamina VIII

##### NeuN

Lamina VIII is clearly visible only in the cervical and lumbar enlargements of the spinal cord; the ventral border is determined by the well-defined motoneuron pools (lamina IX), and the lateral and dorsal borders are determined by the lower density of NeuN-immunopositive neurons of lamina VII (Figures 5A,B).

#### Lamina IX

##### Unstained

In unstained slices, large motoneurons of lamina IX, like many other neurons of the ventral horns, stand out as lighter cells on the dark background of gray matter (Figures 5B,C). Specifically, motoneuron pools can be defined only in cervical and lumbar enlargements of the spinal cord.

##### NeuN

Motoneurons are clearly visible as large multipolar NeuN-immunopositive neurons that form pools (Figures 5B,C).

##### SMI-32

Motoneurons are clearly visible as large multipolar SMI-32-immunopositive neurons that form pools (Figures 5B,C).

##### ChAT

Motoneurons are clearly visible as large multipolar ChAT-immunopositive neurons that form pools (Figures 5B,C).

#### Lamina X

##### NeuN

The borders of lamina X are difficult to identify, but its characteristic feature is small NeuN-immunopositive neurons that have lower density compared to the adjacent gray matter (Figures 5A,B).

#### Central cervical nucleus

##### NeuN

Central cervical nucleus is located symmetrically in the left and right sides of the spinal cord lateral to the central canal in the C1–C4 segments and can be identified by the sparsity of NeuN-immunopositive neurons compared to the adjacent gray matter (Figures 5E,H).

#### Lateral cervical nucleus

##### Unstained

Lateral cervical nucleus is located in the lateral funiculus of the C1–C2 segments (partially C3), and it is clearly distinguishable from the surrounding white matter by its lighter color in unstained slices (Figures 5D,E). Both fibers and neurons with a lighter appearance are well-visualized.

##### NeuN

Lateral cervical nucleus is well-separated from adjacent white matter by background staining of the neuropil and NeuN-immunopositive neurons (Figures 5D,E).

##### Calretinin

Lateral cervical nucleus is well-separated from the adjacent white matter by the background staining of the neuropil and rare CR-immunopositive neurons (Figures 5D,E).

##### Parvalbumin

Lateral cervical nucleus is well-separated from adjacent white matter by background staining of the neuropil and PV-immunopositive neurons (Figures 5D,E).

##### SMI-32

Lateral cervical nucleus is well-separated from the adjacent white matter by the background staining of the neuropil and numerous SMI-32-immunopositive neurons (Figures 5D,E).

#### Nucleus centrodorsalis

##### Unstained

Nucleus centrodorsalis is located in the centromedial part of the lamina VIII of C1 segment and is clearly distinguishable by the lighter appearance relative to adjacent gray matter (Figures 5E,G).

##### NeuN

Nucleus centrodorsalis contains larger NeuN-immunopositive neurons relative to the adjacent gray matter (Figures 5E,G).

##### ChAT

Nucleus centrodorsalis is represented by a group of large ChAT-immunopositive neurons (Figures 5E,G).

##### SMI-32

Nucleus centrodorsalis is represented by a group of large SMI-32-immunopositive neurons (Figures 5E,G).

#### Nucleus commissuralis

##### Unstained

Nucleus commissuralis is located on the medial border of the lamina VIII in the segments C1–C4 and S2–Co2 and can be detected by the group of light neurons (Figures 5E,F).

##### NeuN

NeuN labels the group of large neurons in the CoN (Figures 5E,F).

##### ChAT

Nucleus commissuralis is represented by the group of the large ChAT-immunopositive neurons (Figures 5E,F).

##### SMI-32

Nucleus commissuralis is represented by the group of the large SMI-32-immunopositive neurons (Figures 5E,F).

#### Intermediomedial nucleus

##### ChAT

**FIGURE 6:**
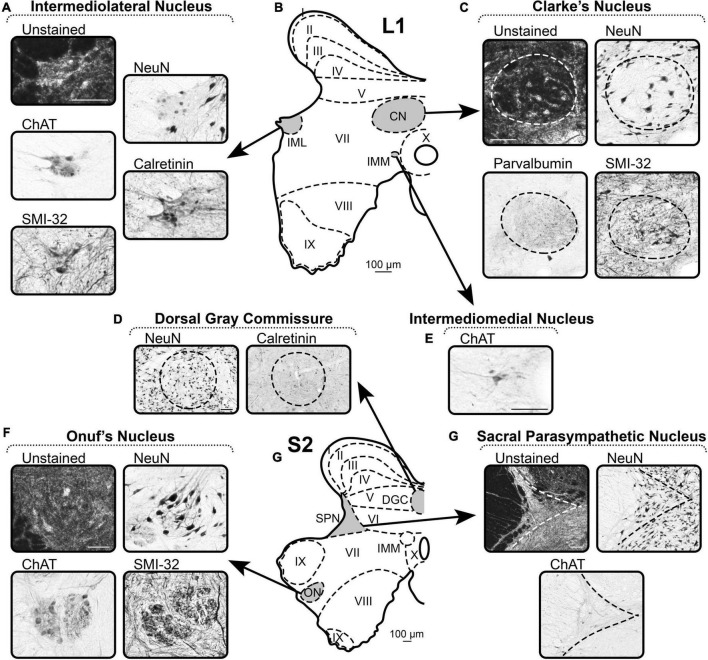
A representative example of the markers of nuclei of spinal cord. **(A–E)** Spinal segment L1. **(F–H)** Spinal segment S2. **(A)** Distribution of the markers in the Intermediolateral Nucleus. **(B)** Scheme of L1 segment. **(C)** Distribution of the markers in the Clarke’s Nucleus. **(D)** Distribution of the markers in the Dorsal Gray Commissure. **(E)** Distribution of the markers in the Intermediomedial Nucleus. **(F)** Distribution of the markers in the Onuf’s Nucleus. **(G)** Scheme of S2 segment. **(H)** Distribution of the markers in the Sacral Parasympathetic Nucleus. Scale bar is 100 μm.

Intermediomedial nucleus is located throughout the entire spinal cord on the sides of the central canal and can be visualized by several well-distinguished ChAT-immunopositive neurons with immunopositive processes (Figures 6B,E).

#### Intercalated nucleus

##### ChAT

Intercalated nucleus is represented by ChAT-immunopositive neurons and fibers localized in lamina VII as a band connecting IMM and IML. The nucleus is poorly marked by ChAT in the cat spinal cord, unlike other animal species (e.g., rodents), and rarely visualized. Therefore, IC is not depicted in the schematic images.

#### Intermediolateral nucleus

##### Unstained

Intermediolateral nucleus is well-visualized as a lighter zone of the most lateral part of intermediate gray matter (Figures 6A,B).

##### NeuN

Intermediolateral nucleus is well-defined by the lower density of NeuN-labeling compared to adjacent gray matter (Figures 6A,B). The nucleus looks “empty,” containing a small number of NeuN-immunopositive neurons and neuronal nuclei without labeled soma.

##### ChAT

ChAT labels well the neuronal soma and the neuropil (Figures 6A,B). Thus, IML is clearly visualized after ChAT labeling.

##### Calretinin

CR labels well the neuronal soma and the neuropil (Figures 6A,B). Thus, IML is clearly visualized after CR labeling.

##### SMI-32

SMI-32 labels neurons of IML; however, in many slices, it is difficult to determine the exact borders of the nucleus with the adjacent gray matter due to the similar SMI-32-immunoreactivity of the fibers in the intermediate gray matter (Figures 6A,B).

#### Clarke’s nucleus

##### Unstained

In unstained slices, CN are visualized as dark round areas symmetrically located above the central canal in the left and right halves of the spinal cord (Figures 6B,C). Some large light neurons can be identified.

##### NeuN

Clarke’s nucleus borders are defined by NeuN-immunopositive neurons elongated along the edge of the nucleus (Figures 6B,C). CN is also visualized by the lower density of the NeuN-immunopositive neurons compared with the adjacent gray matter.

##### Parvalbumin

Clarke’s nucleus is well-defined by the PV-immunopositive neuropil, which is darker than the gray matter surrounding the nucleus (Figures 6B,C).

##### SMI-32

Clarke’s nucleus borders are defined by SMI-32-immunopositive fibers that follow the edge of the nucleus and have a lower density compared to the adjacent gray matter (Figures 6B,C). In addition, CN have large oval SMI-32-immunopositive neurons.

#### Dorsal gray commissure

##### NeuN

Dorsal gray commissure is a round formation with NeuN-immunopositive neurons localized above the central canal in S2–S3 segments (Figures 6D,G). On the sides, this nucleus can be visually separated from the adjacent laminae by light stripes of NeuN-negative fibers.

##### Calretinin

Dorsal gray commissure can be partially detected by CR immunolabeling because only a small proportion of neurons in the nucleus express CR (Figures 6D,G).

#### Sacral parasympathetic nucleus

##### Unstained

The clear border of SPN is difficult to detect in unstained slices; however, the general outline can be identified by a lighter appearance relative to adjacent laminae (Figures 6G,H).

##### NeuN

Sacral parasympathetic nucleus can be identified by NeuN-immunopositive neurons that have bright labeled nuclei and light somas (Figures 6G,H).

##### ChAT

Sacral parasympathetic nucleus is well-visualized by ChAT-immunopositive neurons and fibers that actually form this nucleus (Figures 6G,H).

#### Onuf’s nucleus

##### Unstained

Onuf’s nucleus is located in segment S1 and rostral part of S2, within the ventral horns, between the somatic motoneuronal pools, and can be detected as a light area with small-sized motoneurons (Figures 6F,G).

##### NeuN

Onuf’s nucleus can be detected as a separate pool of small-sized NeuN-immunopositive motoneurons (Figures 6F,G).

##### ChAT

Onuf’s nucleus can be detected as a separate pool of small-sized ChAT-immunopositive motoneurons surrounded by the ChAT-immunopositive neuropil (Figures 6F,G).

##### SMI-32

Onuf’s nucleus can be detected as a separate pool of small SMI-32-immunopositive motoneurons in small-sized SMI-32-immunopositive neuropil (Figures 6F,G).

## Discussion

Currently, there is only one atlas of the cat spinal cord, authored by Rexed (1954), which is still used by everyone who studies the structure and function of the feline spinal cord. Despite the importance of Rexed’s atlas, it is clear that data on only the total cytoarchitectonic division do not provide information about the structure and location of multiple neuronal populations of the spinal cord. Moreover, some thoracic segments and some structures (e.g., SPN, DGC, and ON) are missing in the schemes of Rexed’s atlas.

At the same time, using various neurochemical markers, complex neuromorphological atlases have been created for mice and rats (Watson et al., 2009, 2021; Sengul et al., 2012), marmosets (Tokuno et al., 2011; Sengul et al., 2012; Watson et al., 2021), rhesus monkeys, and humans (Sengul et al., 2012; Watson et al., 2021). Moreover, another source for information about neurochemical diversity of spinal neuronal networks is interactive online databases that present data on the expression of multiple genes in the spinal cord of mice obtained using RNA *in situ* hybridization or transgenic mouse techniques (e.g., https://mousespinal.brain-map.org, http://gensat.org/index.html, https://seqseek.ninds.nih.gov). However, no such databases have been created for the cat.

Our atlas illustrates not only cervical-coccygeal segments but also different parts of their segments because network organization is not uniform within the same segment and may significantly differ in its rostral and caudal parts. Using 6 neurochemical markers (i.e., NeuN, ChAT, CB, CR, PV, and SMI-32), we identified multiple populations of motoneurons and interneurons. The high resolution of images used allows to count and measure different neurons; this approach is useful for the morphological screening of the neuronal populations of the total spinal cord. Currently, only limited data exist about the neurochemical organization of the gray matter of the feline spinal cord, and attention has been mainly focused on particular segments or particular cell populations of the spinal cord (Arvidsson et al., 1989; Traub et al., 1989; Carr et al., 1998, 1999; Ritz et al., 2001; Christiansen et al., 2003; Anelli and Heckman, 2005; Merkulyeva et al., 2016; Veshchitskii et al., 2021, 2022). We believe that this atlas will help to fill the gaps in the neuromorphological knowledge of the cat spinal cord.

Another feature of this atlas is the detailed spatial relationship between the spinal segments and vertebrae. One part of this data (for the lumbosacral region) has been previously published (Shkorbatova et al., 2019). We also present morphometric data about all spinal segments; this data are consistent with the results of other morphometric studies on the adult cat spinal cord (Thomas and Combs, 1962; Maierl and Liebich, 1998). A stereotactic grid for each segment is necessary for the surgical access during neurotracing and neurophysiological studies. We believe that this atlas will help to minimize the number of experimental animals and the area of surgical intervention. Moreover, our atlas can help with the interpretation of the already obtained neurophysiological data.

### Limitations

Images for the atlas were created using a single female cat; however, it is clear that gender may influence some neuronal features [e.g., the size of gender-specific nuclei (Sakamoto, 2014)]. Moreover, individual peculiarities are also possible. Of note, some neurochemical patterns identified here have been previously verified (Merkulyeva et al., 2016, 2019; Veshchitskii et al., 2021), and we are sure that for the animal used in this study, the images were not influenced by the unique staining patterns. Owing to the difficulty in defining the intersegmental borders for the coccygeal region (very thin tightly located roots), only first two segments were processed for the atlas.

## Data availability statement

The original contributions presented in this study are included in the article/Supplementary material, further inquiries can be directed to the corresponding author.

## Ethics statement

The animal study was reviewed and approved by Ethics Commission of the Pavlov Institute of Physiology (Protocol #30/01/2020).

## Author contributions

AV and NM: conceptualization and investigation. AV: software, formal analysis, and visualization. PS and NM: validation. NM: resources, data curation, supervision, project administration, and funding acquisition. All authors: methodology, writing—original draft preparation, review, and editing, approved the final version, and evaluated the accuracy and integrity of the work.
