# Supplementary material for: Neurochemical atlas of the cat spinal cord
Source: Front Neuroanat. 2022 Oct 19;16:1034395. doi: 10.3389/fnana.2022.1034395 (PMC9627295; doi:10.3389/fnana.2022.1034395)
Supplement: Supplementary file 1 [file Data_Sheet_1.PDF]

Supplementary Material

1 Abbreviations

I – lamina I

II – lamina II

III – lamina III

IV – lamina IV

V – lamina V

VI – lamina VI

VII – lamina VII

VIII – lamina VIII

IX – lamina IX

X – lamina X

CCN – Central Cervical Nucleus

CDN – Nucleus Centrodorsalis

CoN – Nucleus Commissuralis

IC – Intercalated Nucleus

IMM – Intermediomedial Nucleus

LCN – Lateral Cervical Nucleus

S_{white} – area of the white matter

S_{gray} – area of the gray matter

2 Supplementary Figures

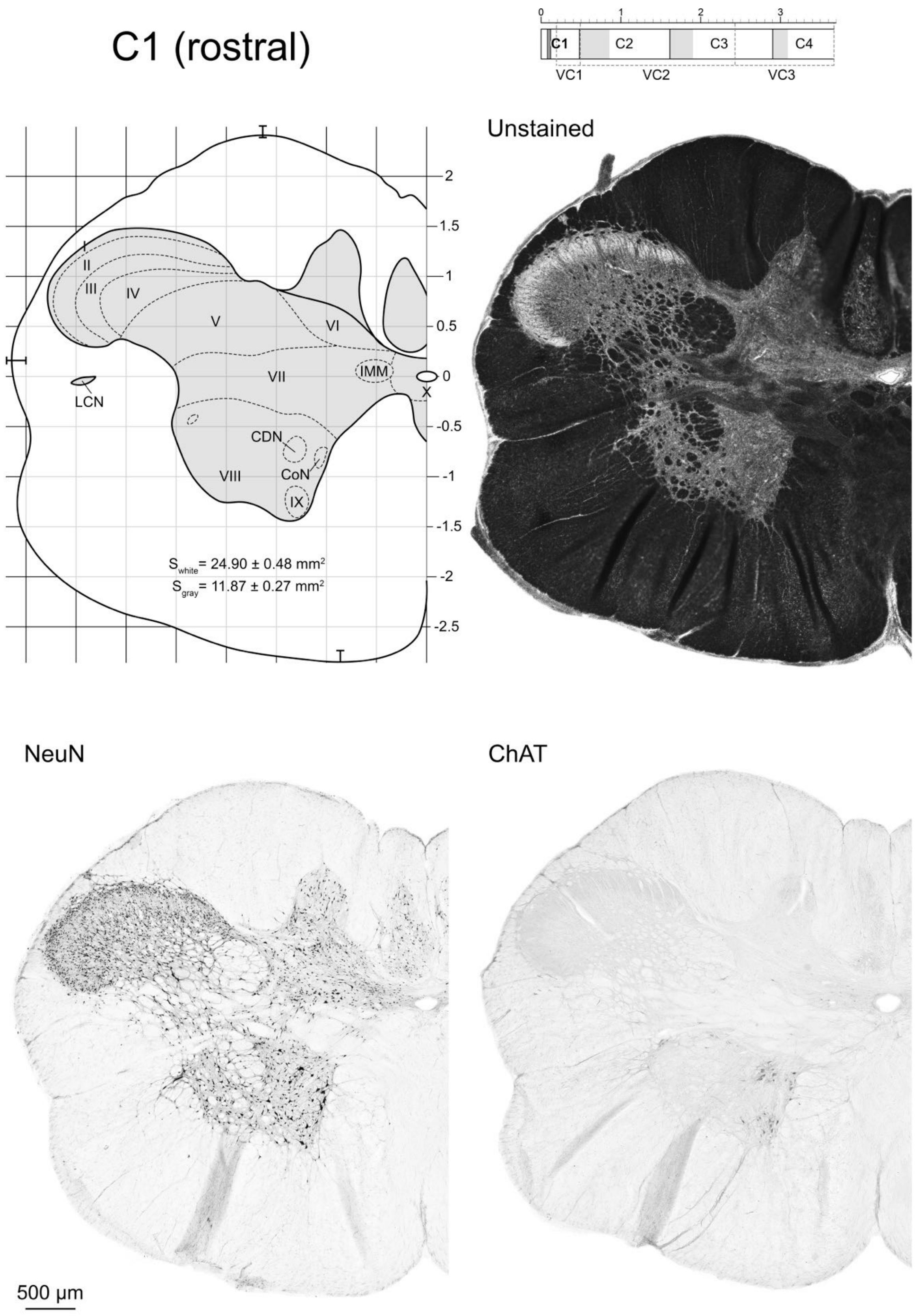

Supplementary Figure 1. Rostral part of C1 segment of the cat spinal cord.

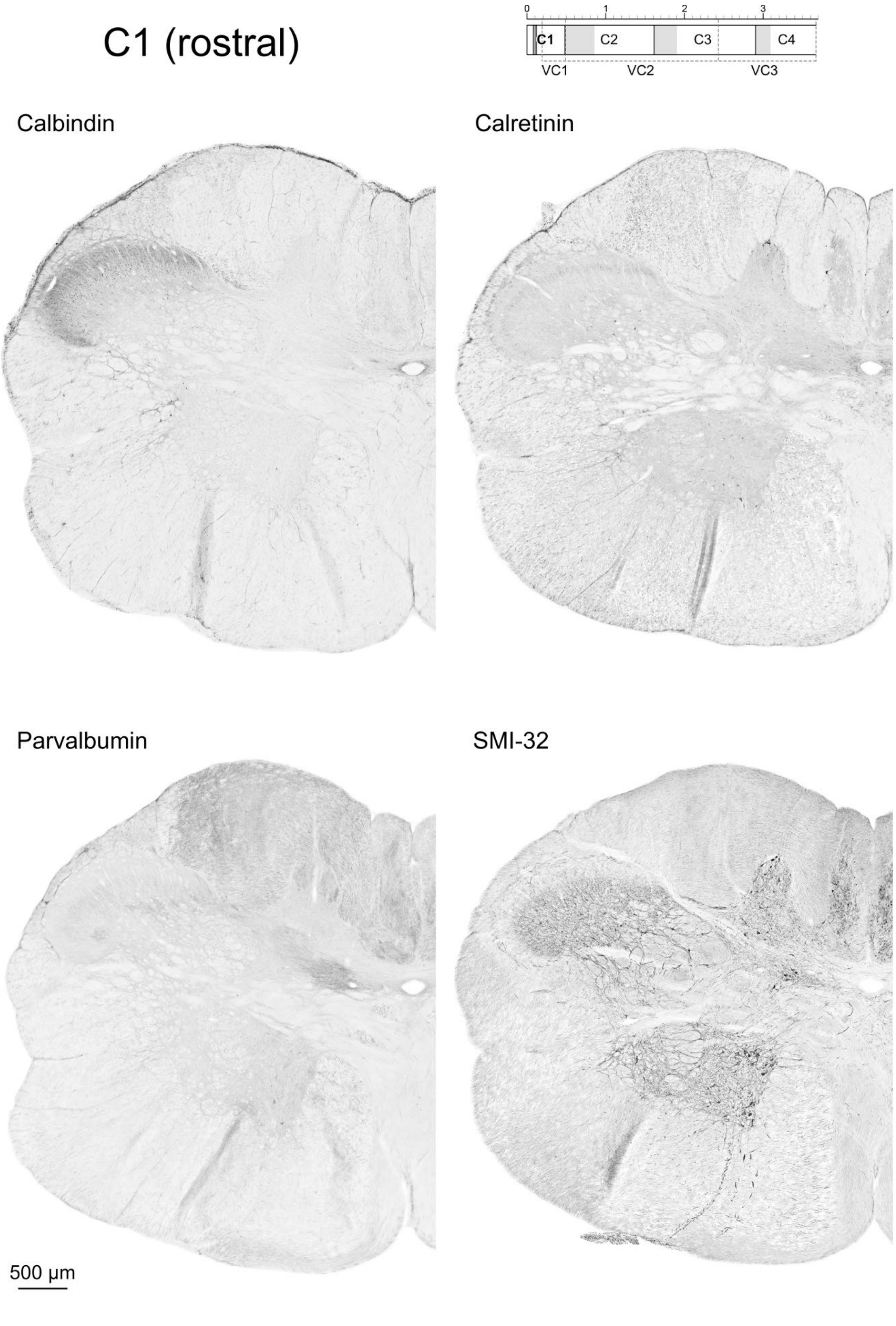

Supplementary Figure 1. Continued.

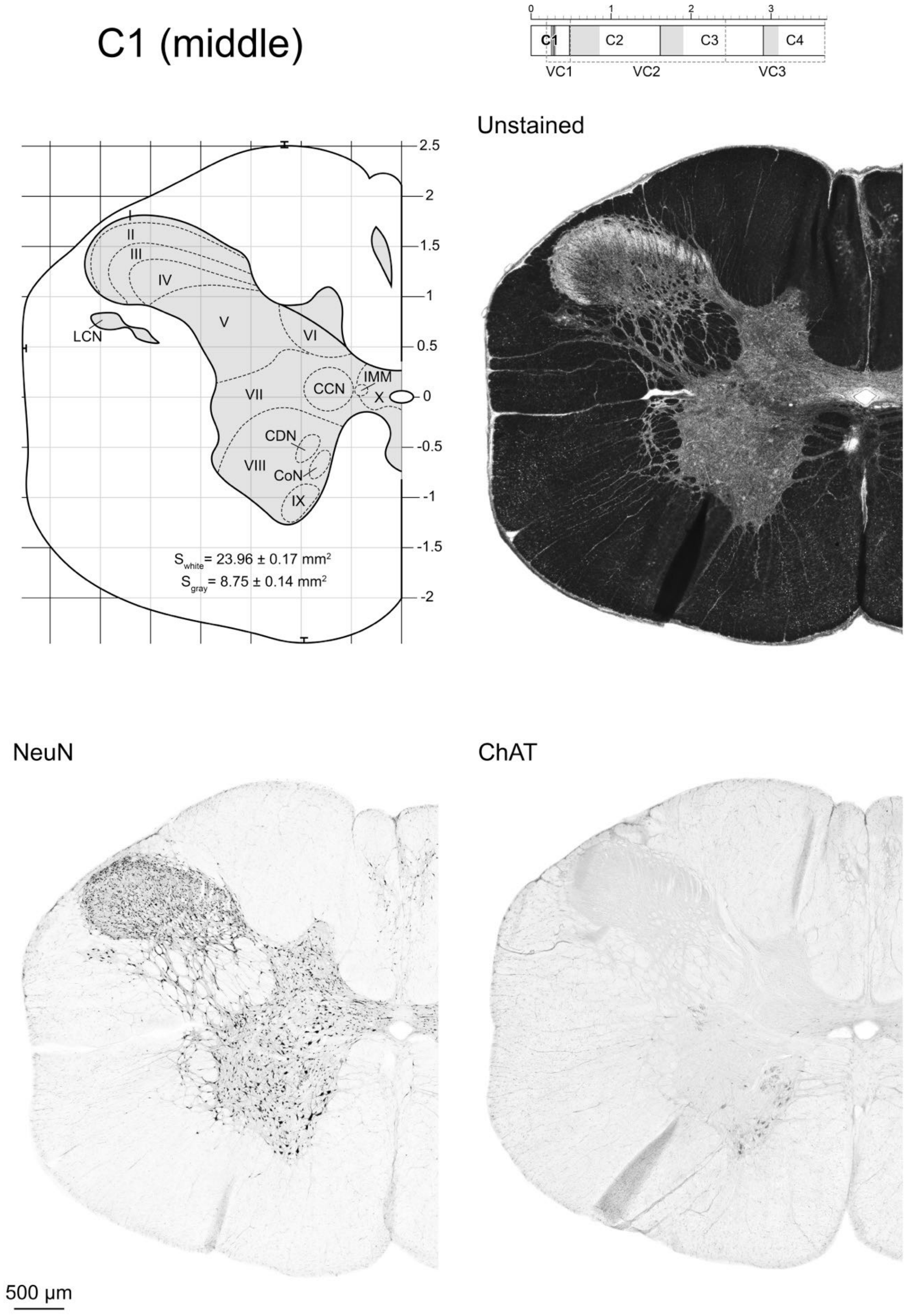

Supplementary Figure 2. Middle part of C1 segment of the cat spinal cord.

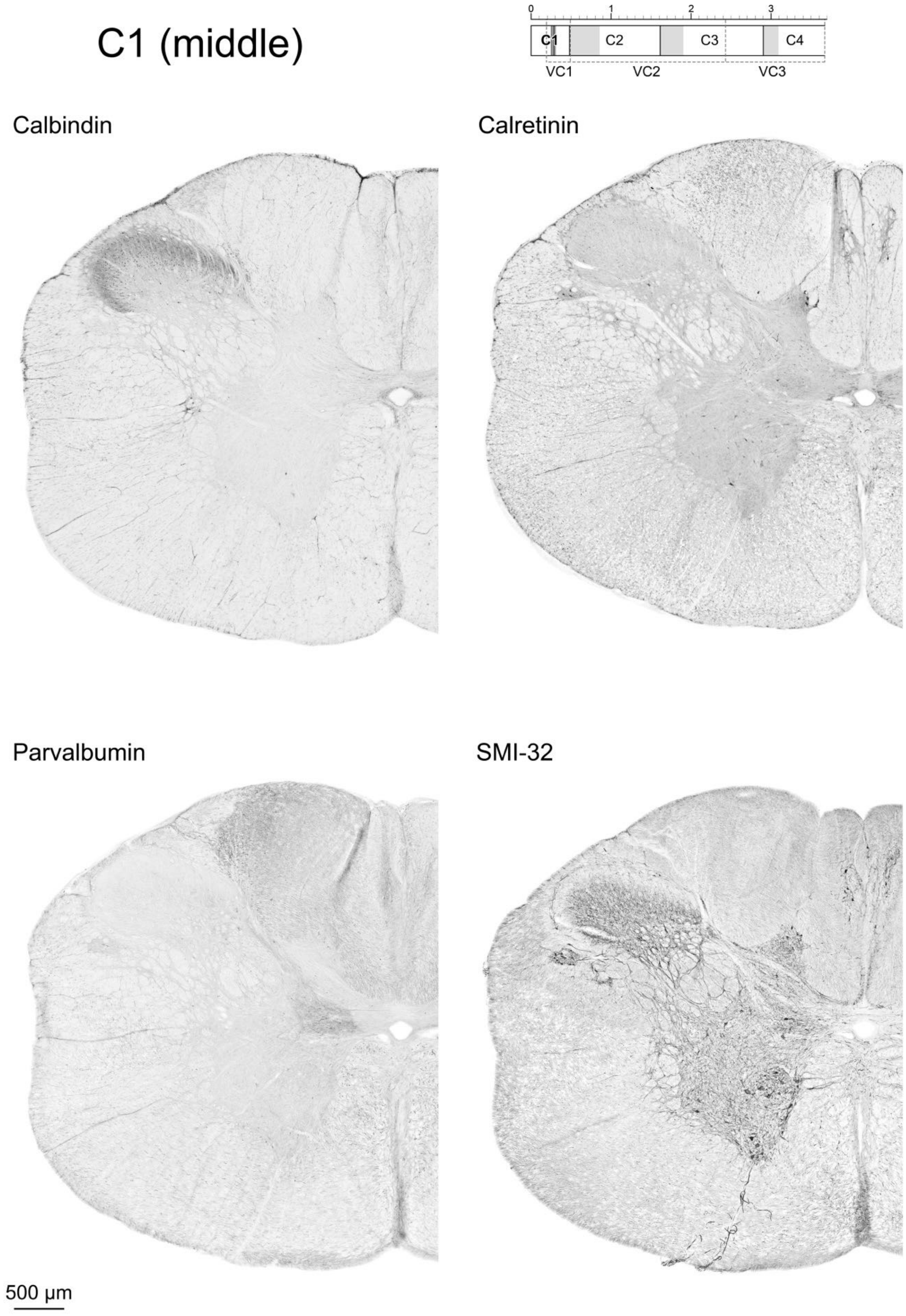

Supplementary Figure 2. Continued.

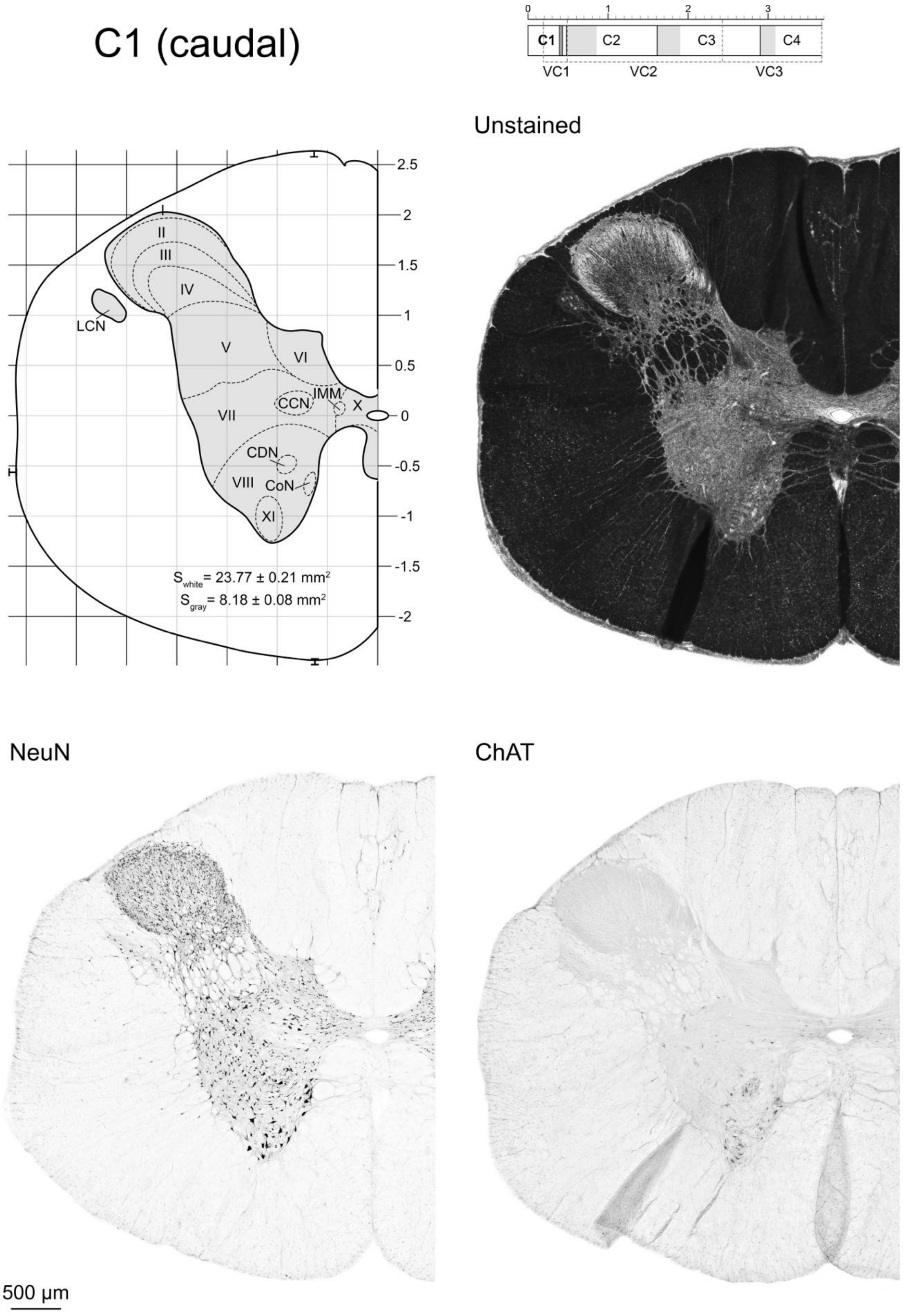

Supplementary Figure 3. Caudal part of C1 segment of the cat spinal cord.

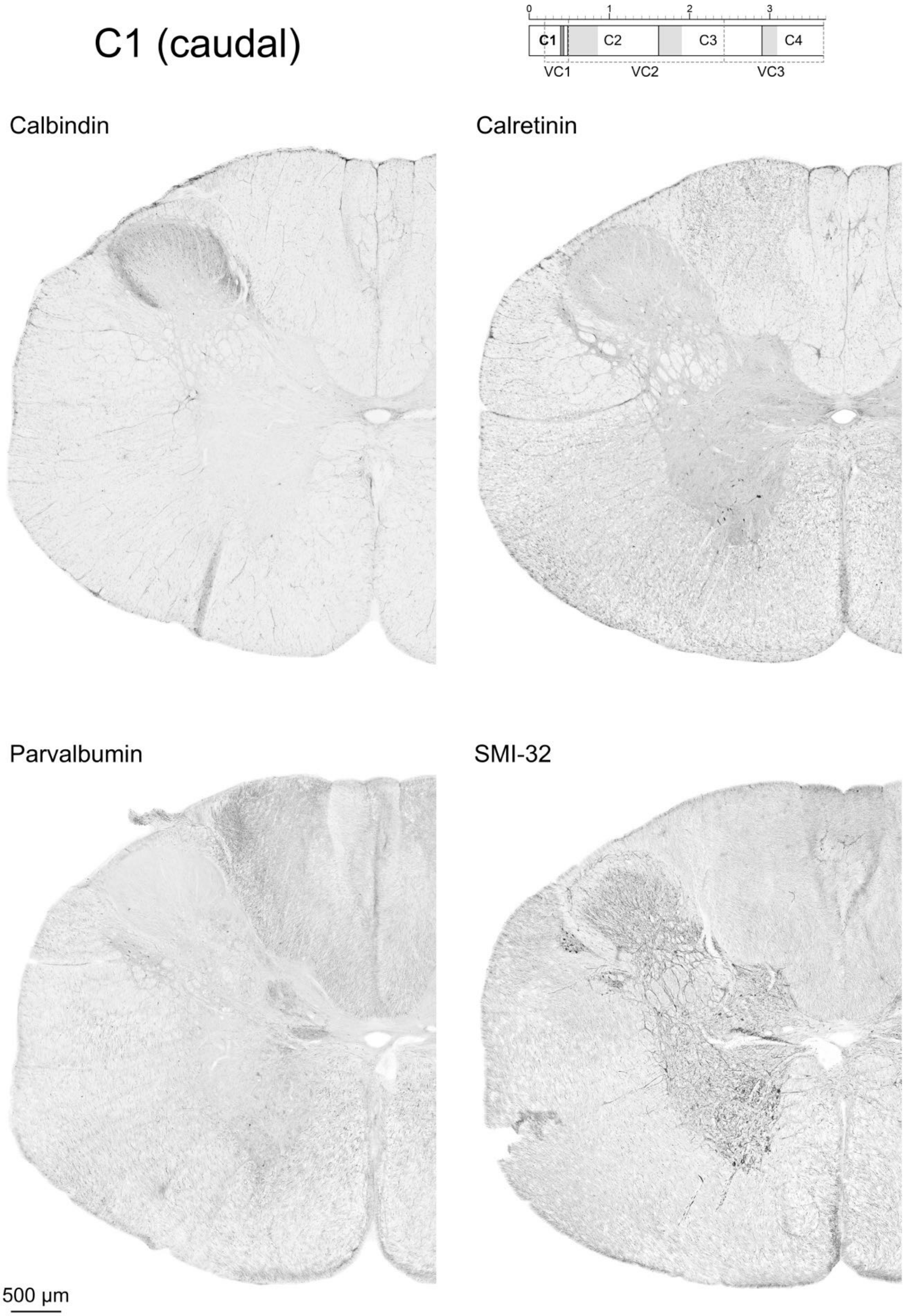

Supplementary Figure 3. Continued.

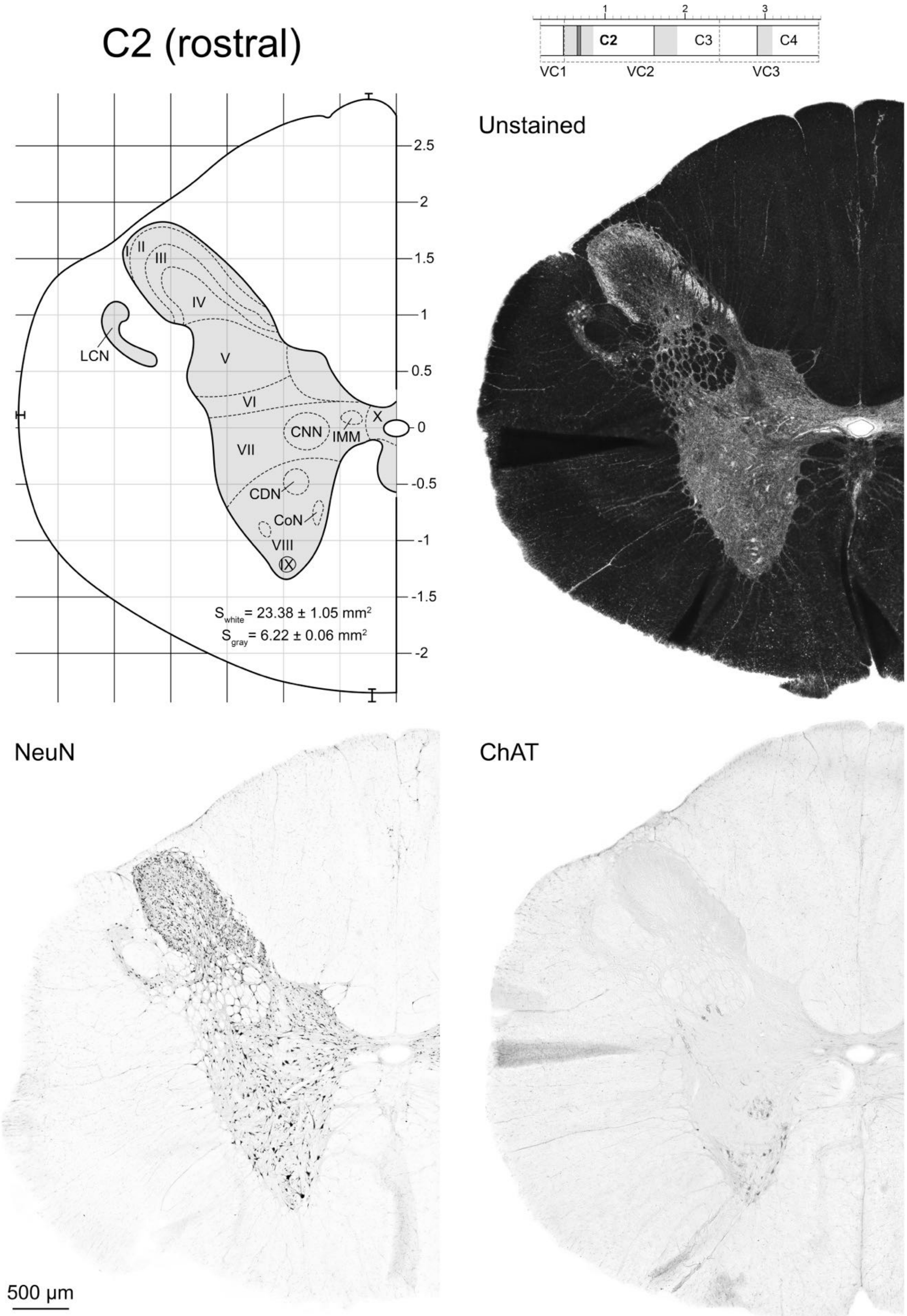

Supplementary Figure 4. Rostral part of C2 segment of the cat spinal cord.

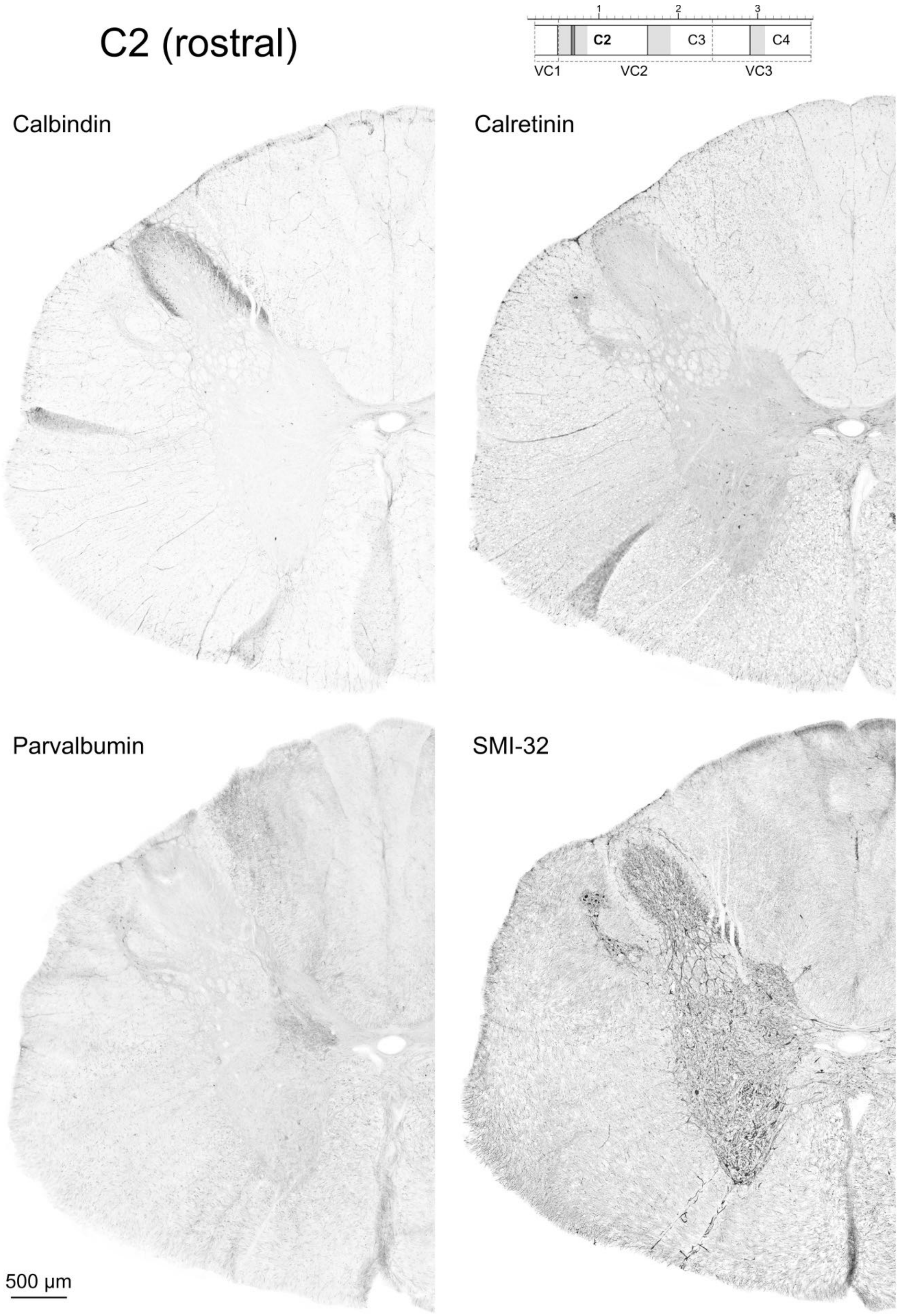

Supplementary Figure 4. Continued.

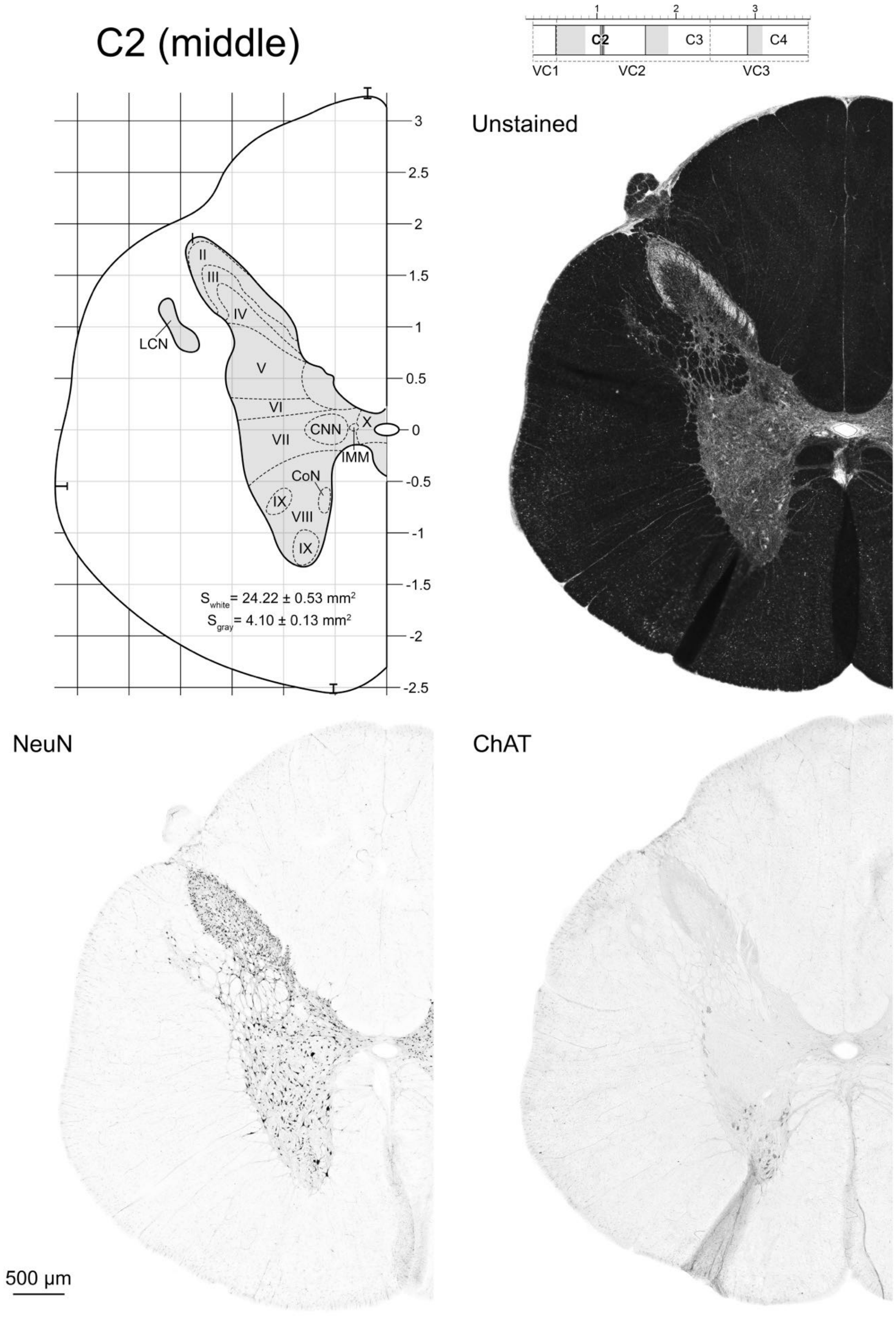

Supplementary Figure 5. Middle part of C2 segment of the cat spinal cord.

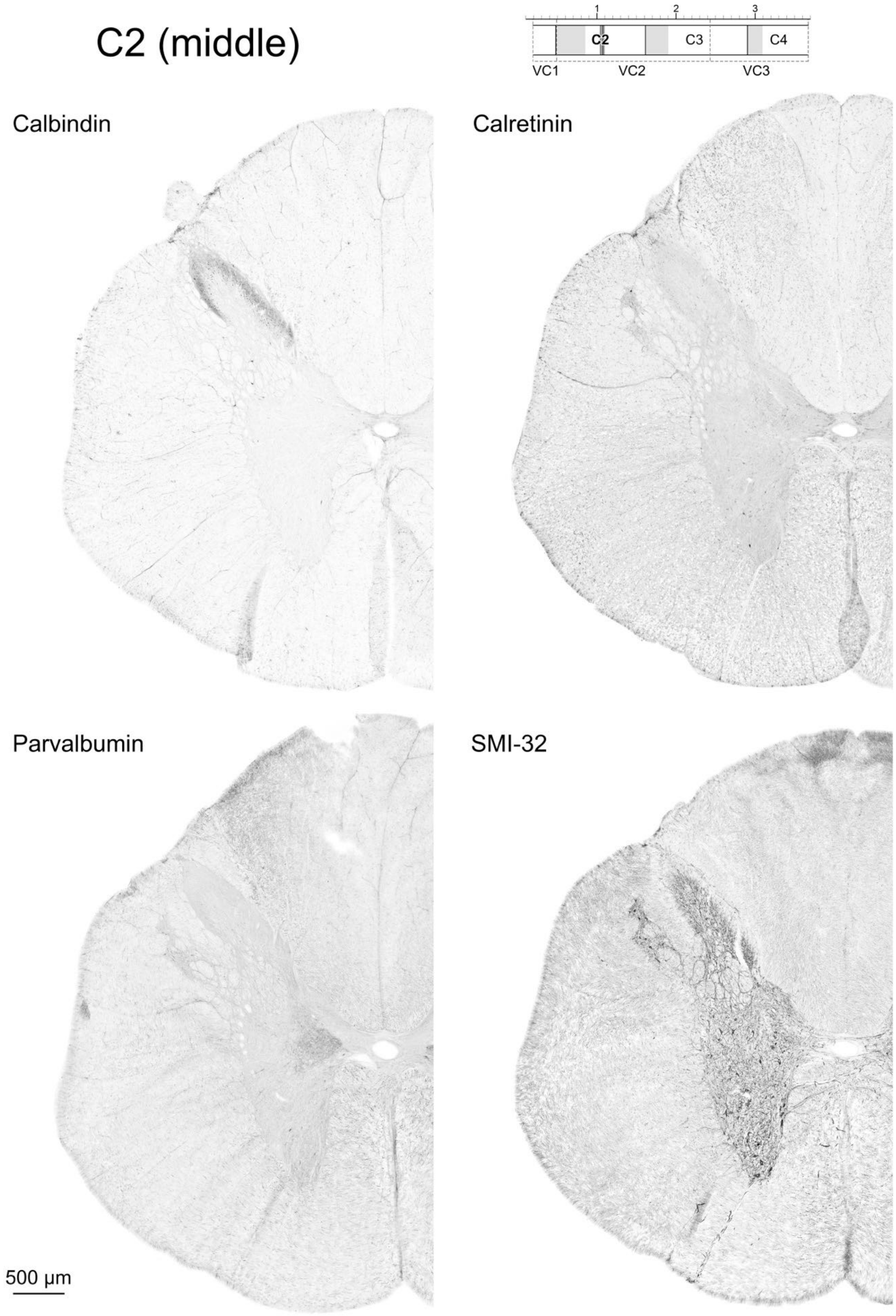

Supplementary Figure 5. Continued.

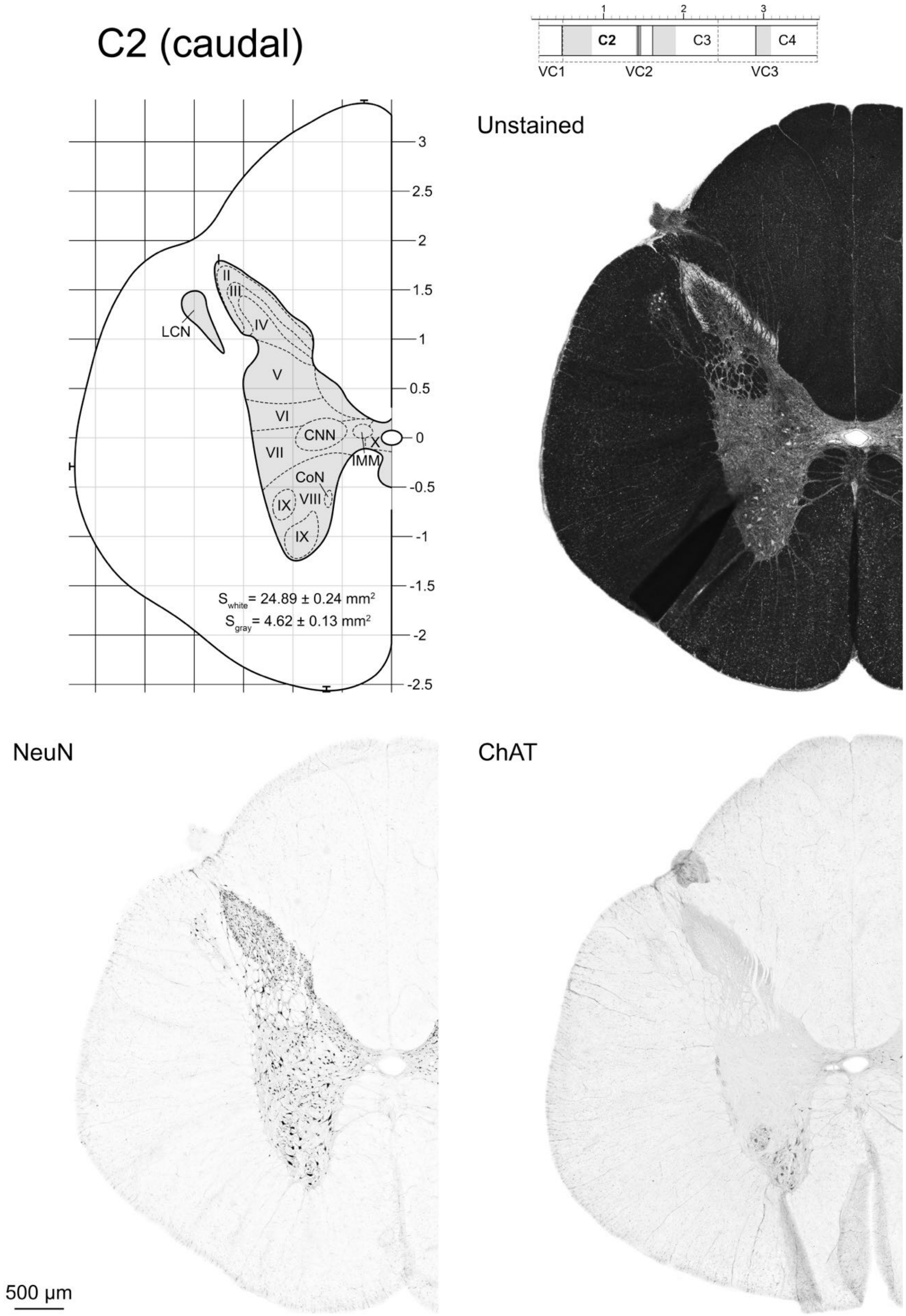

Supplementary Figure 6. Caudal part of C2 segment of the cat spinal cord.

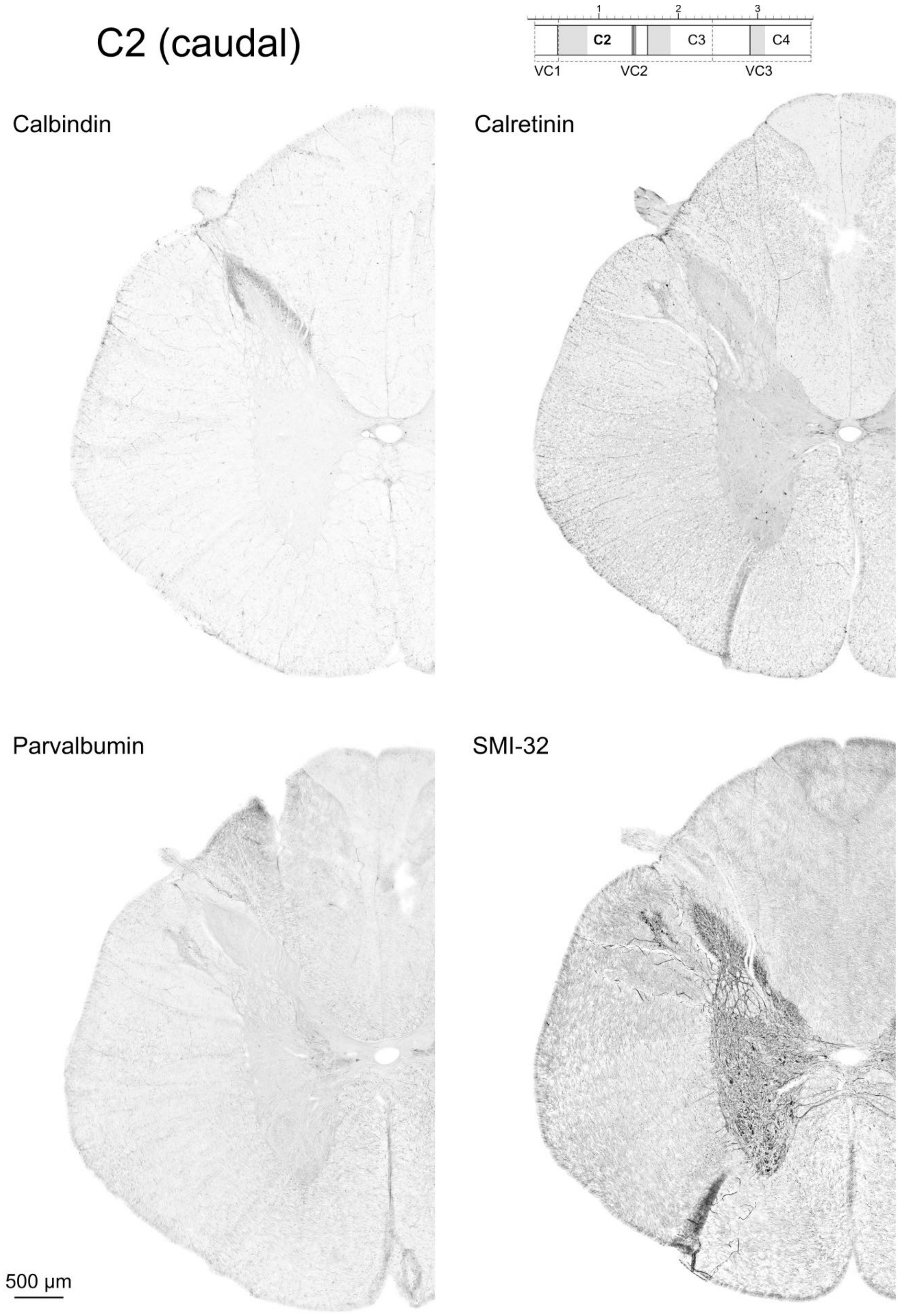

Supplementary Figure 6. Continued.

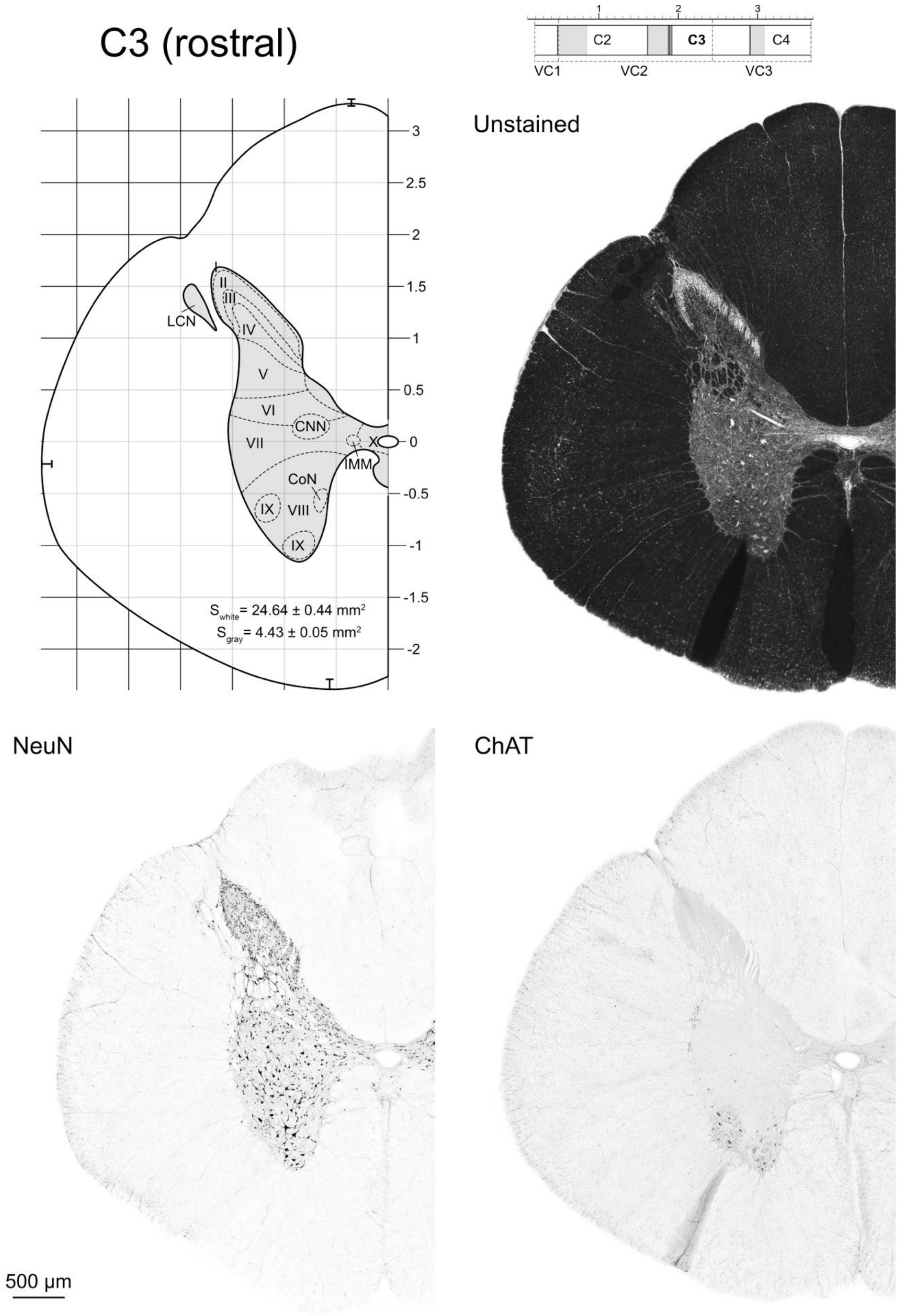

Supplementary Figure 7. Rostral part of C3 segment of the cat spinal cord.

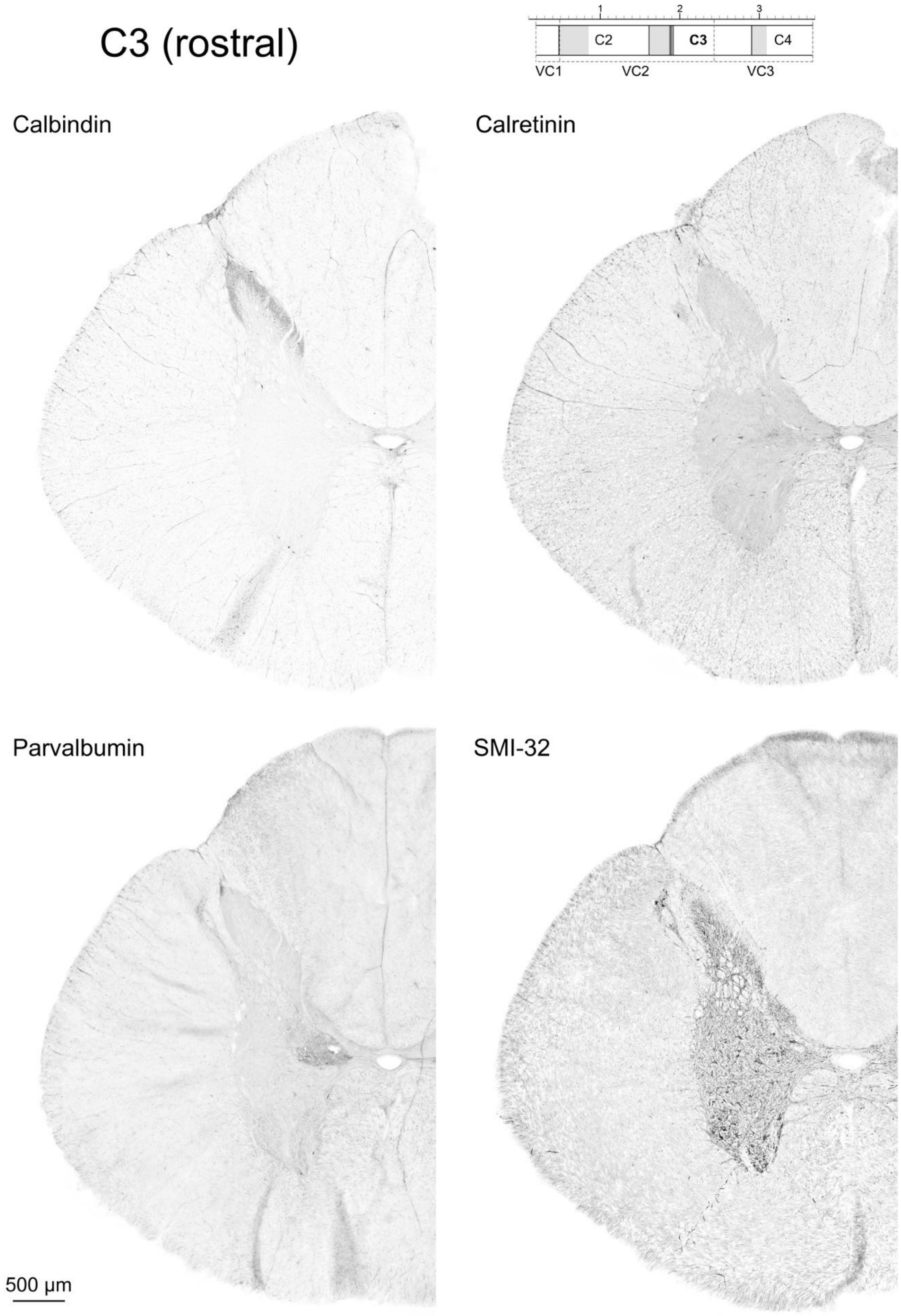

Supplementary Figure 7. Continued.

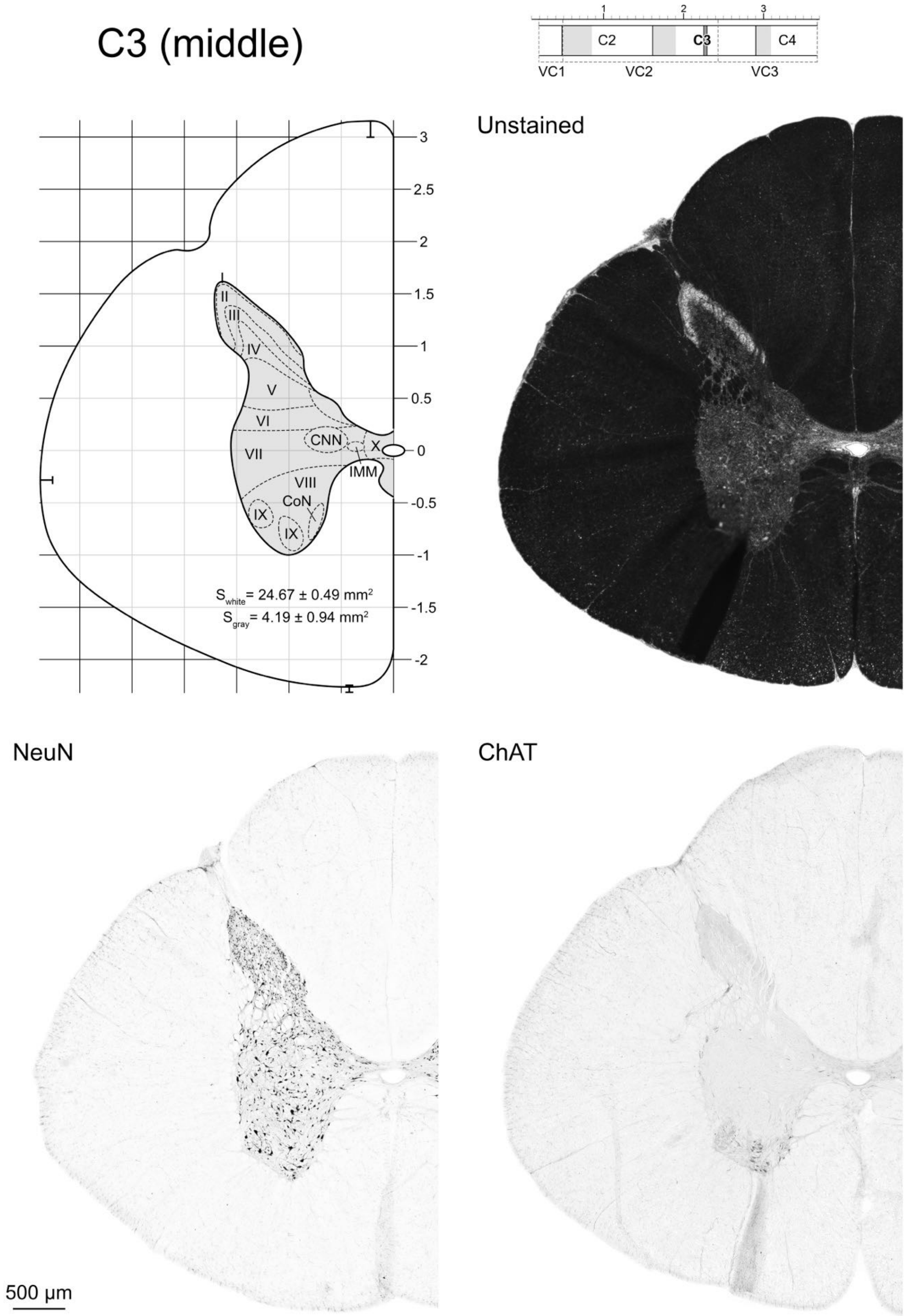

Supplementary Figure 8. Middle part of C3 segment of the cat spinal cord.

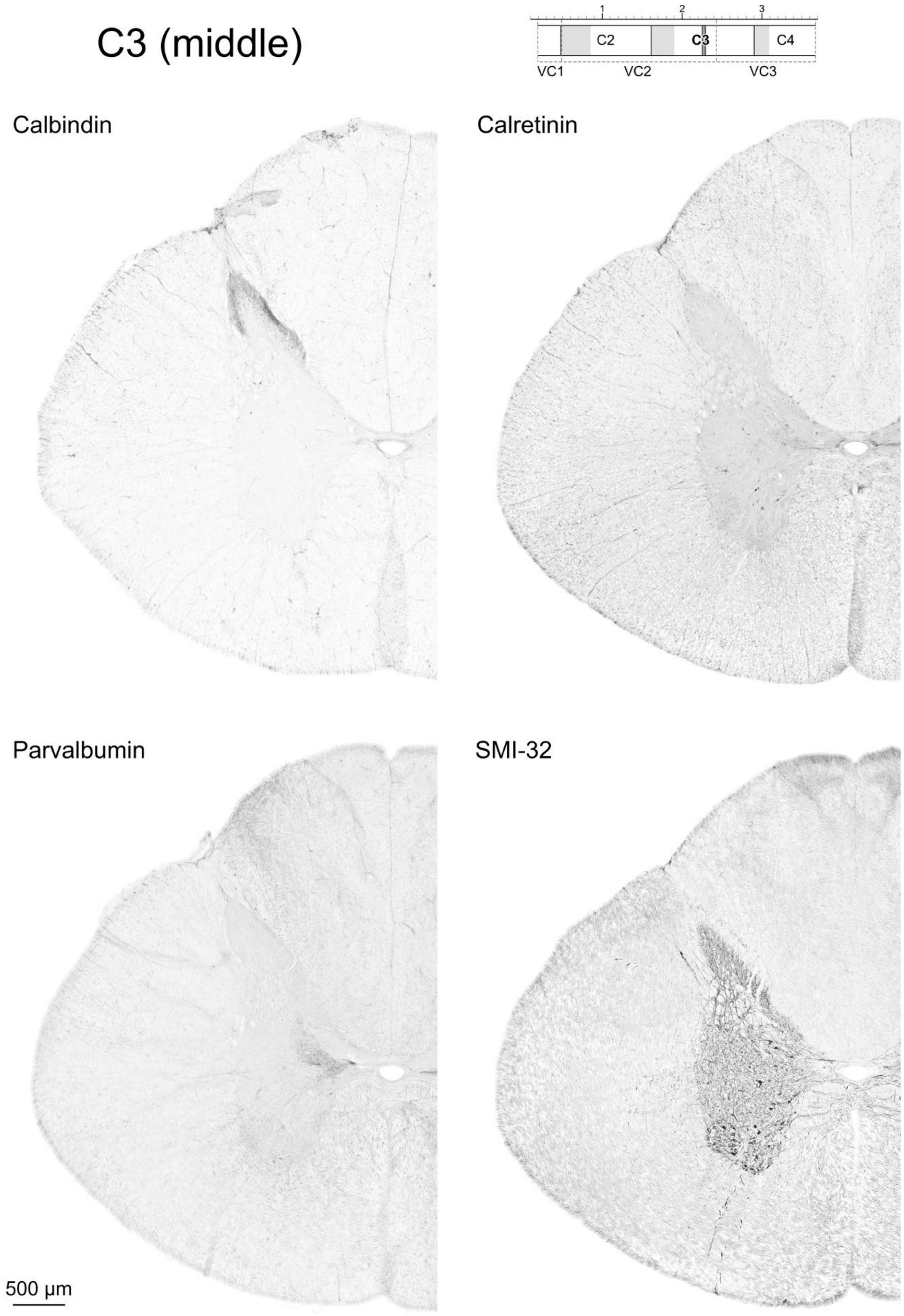

Supplementary Figure 8. Continued.

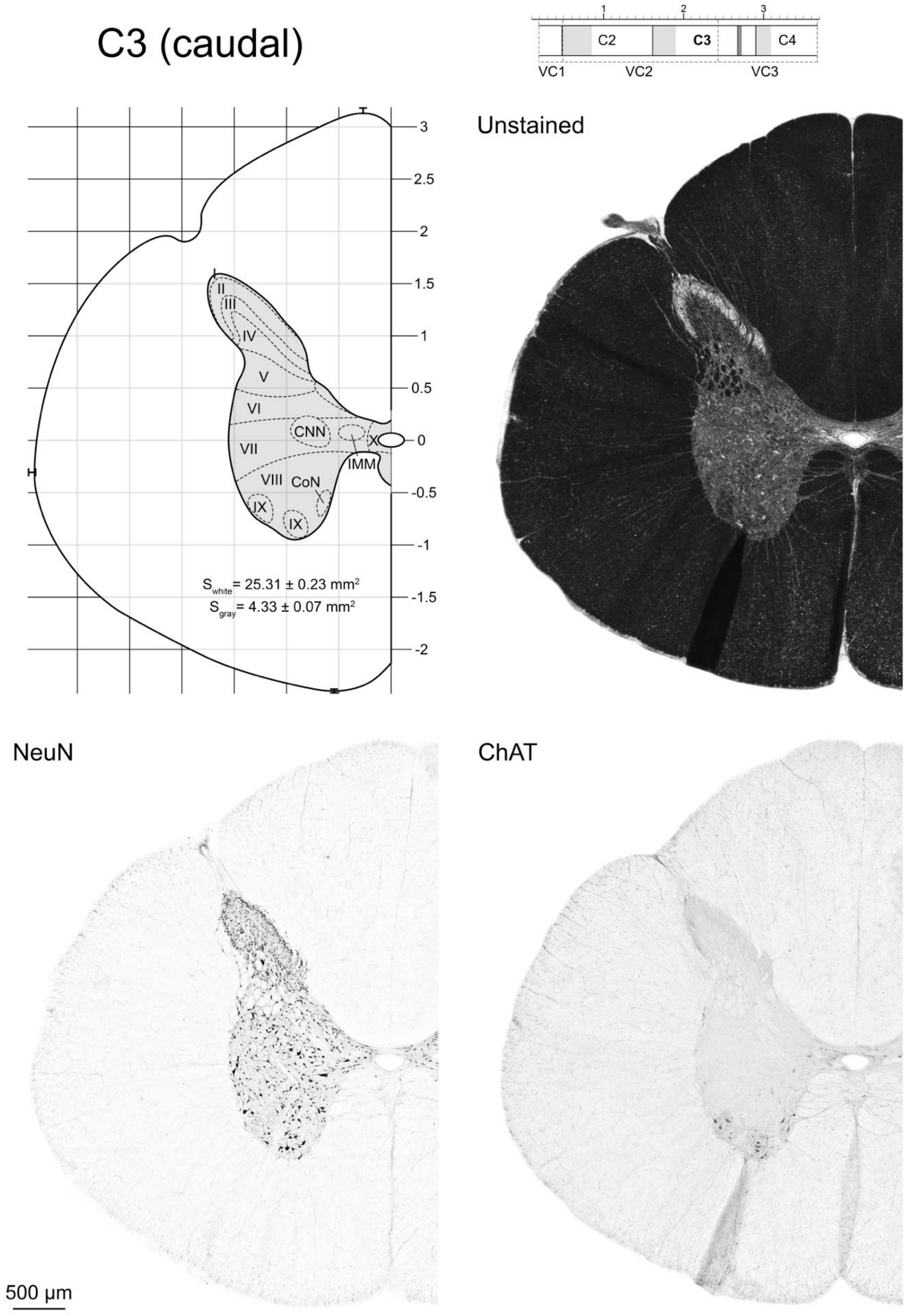

Supplementary Figure 9. Caudal part of C3 segment of the cat spinal cord.

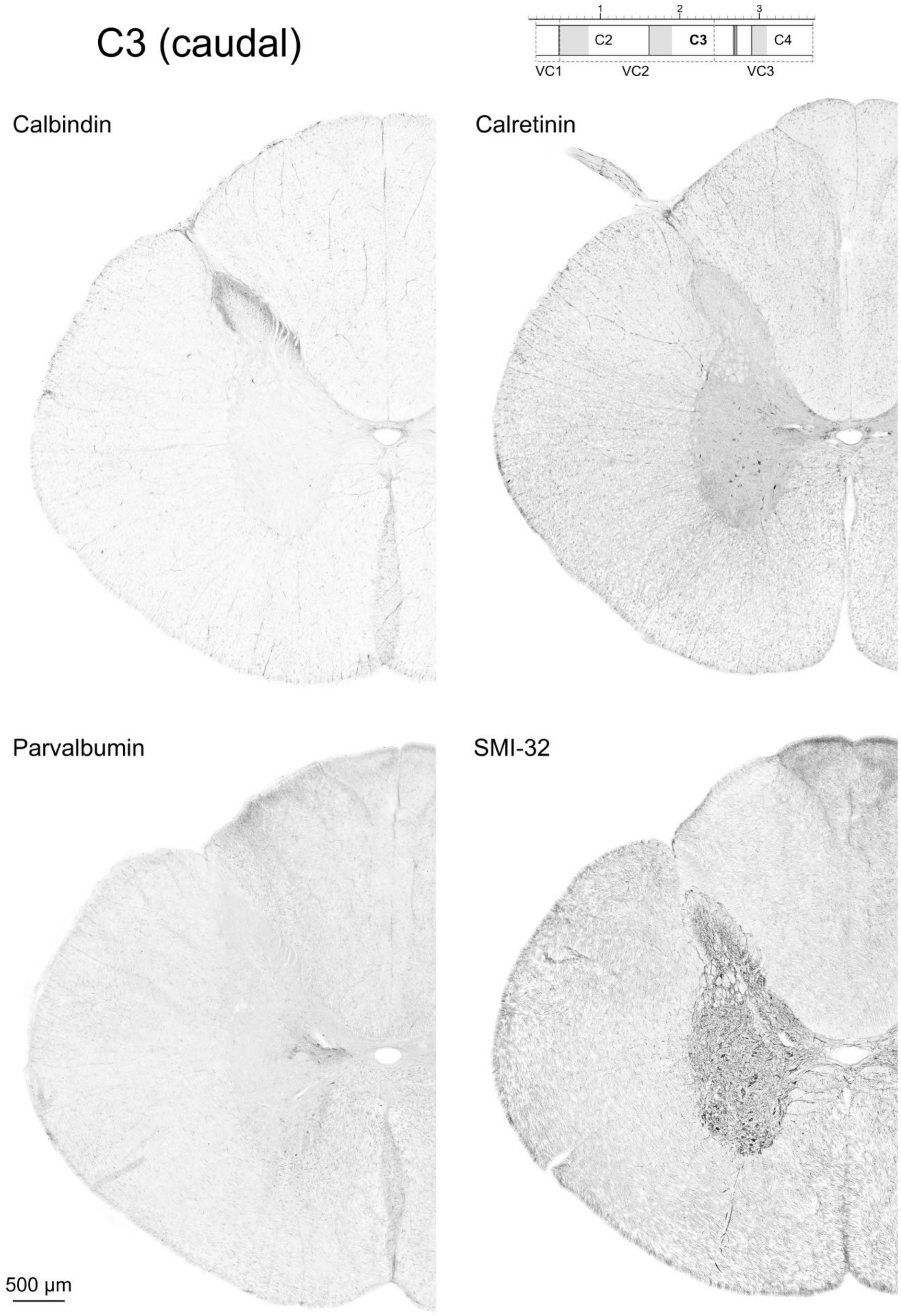

Supplementary Figure 9. Continued.

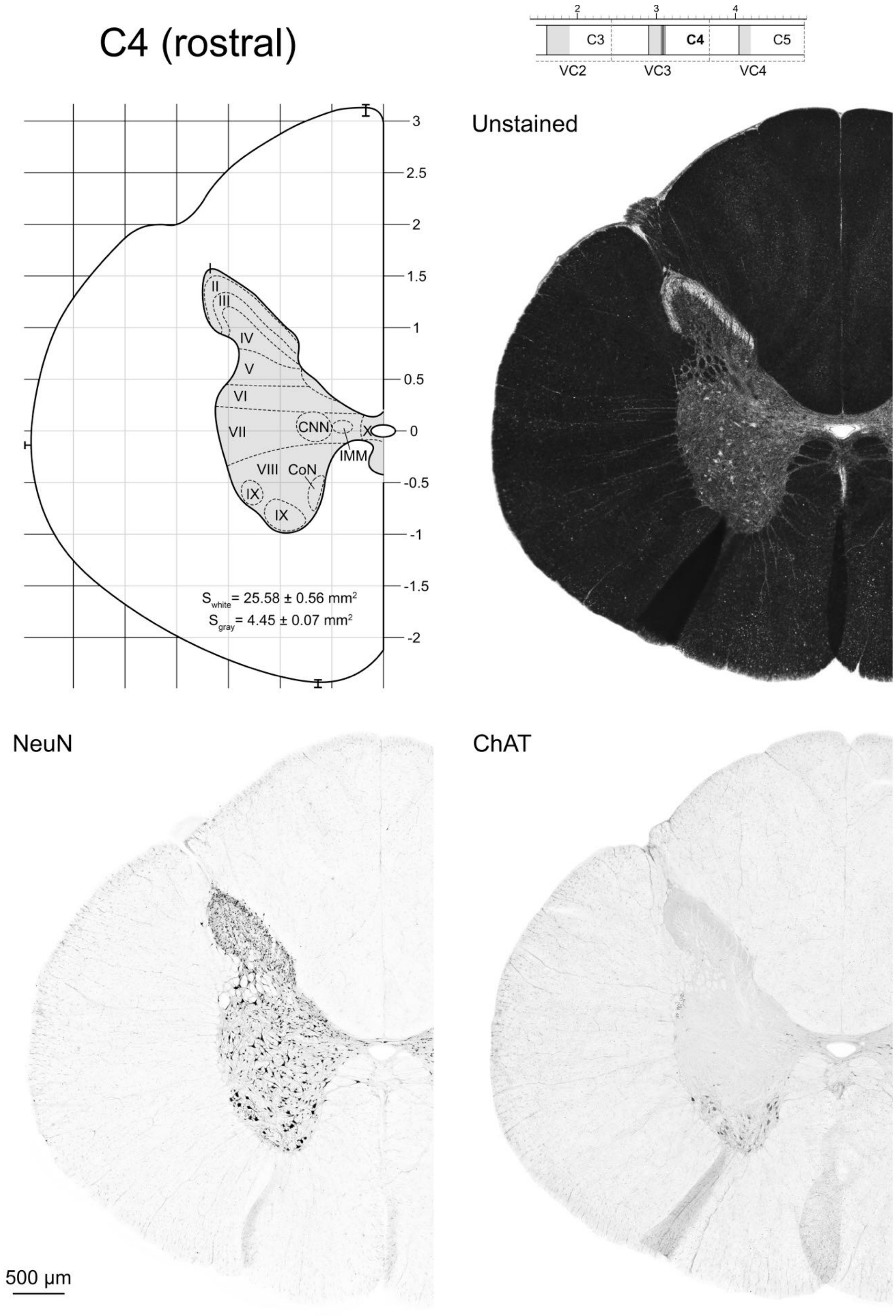

Supplementary Figure 10. Rostral part of C4 segment of the cat spinal cord.

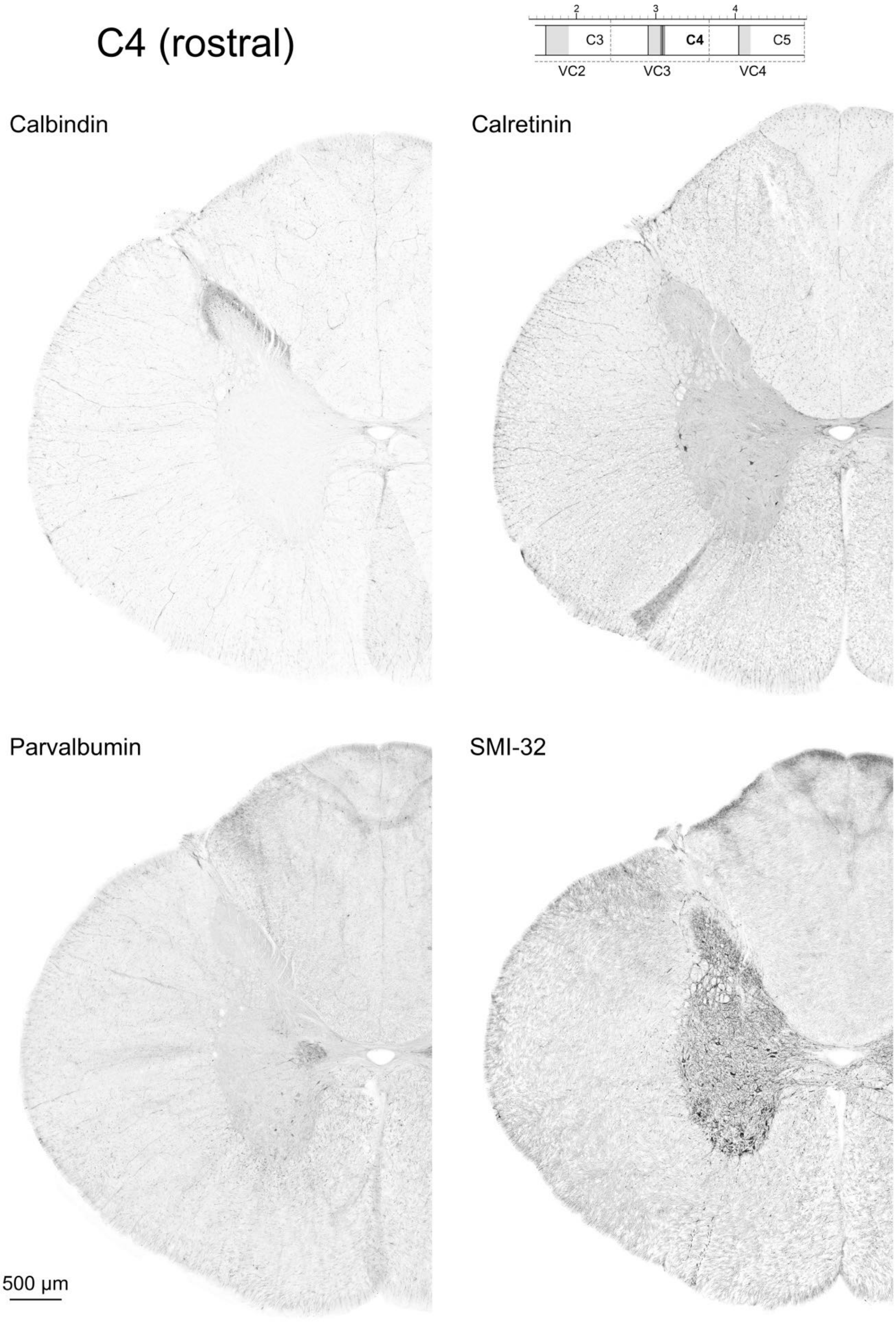

Supplementary Figure 10. Continued.

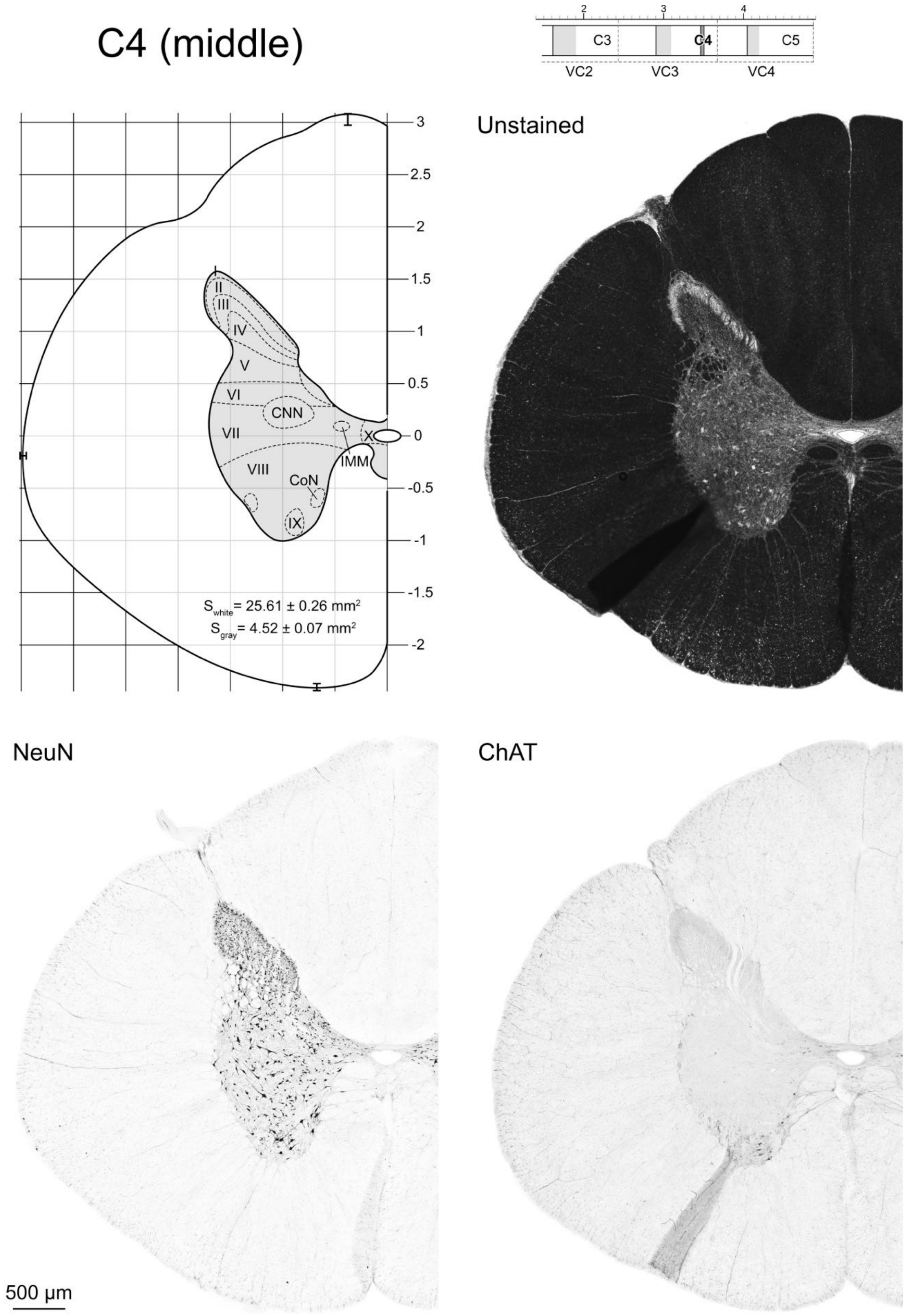

Supplementary Figure 11. Middle part of C4 segment of the cat spinal cord.

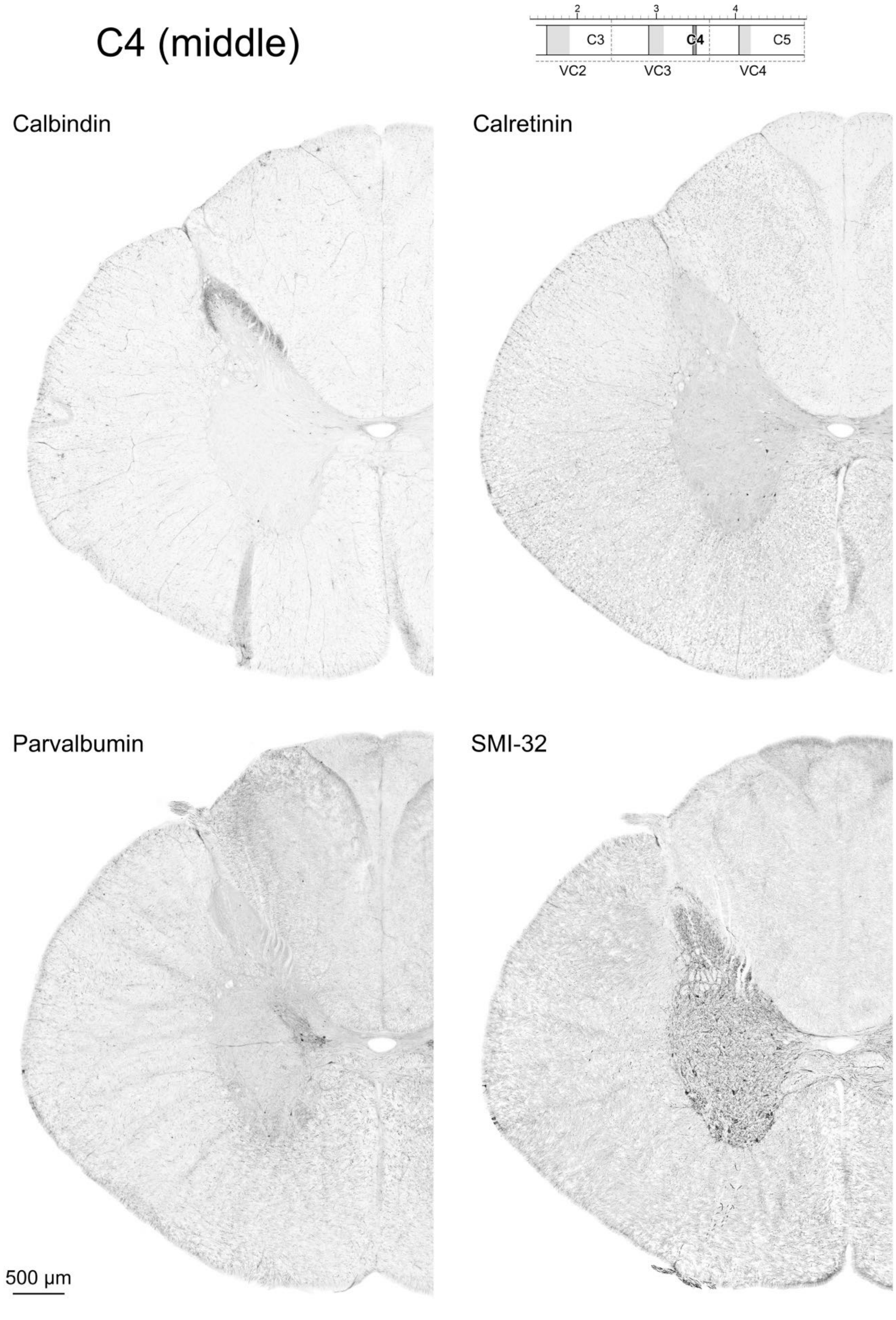

Supplementary Figure 11. Continued.

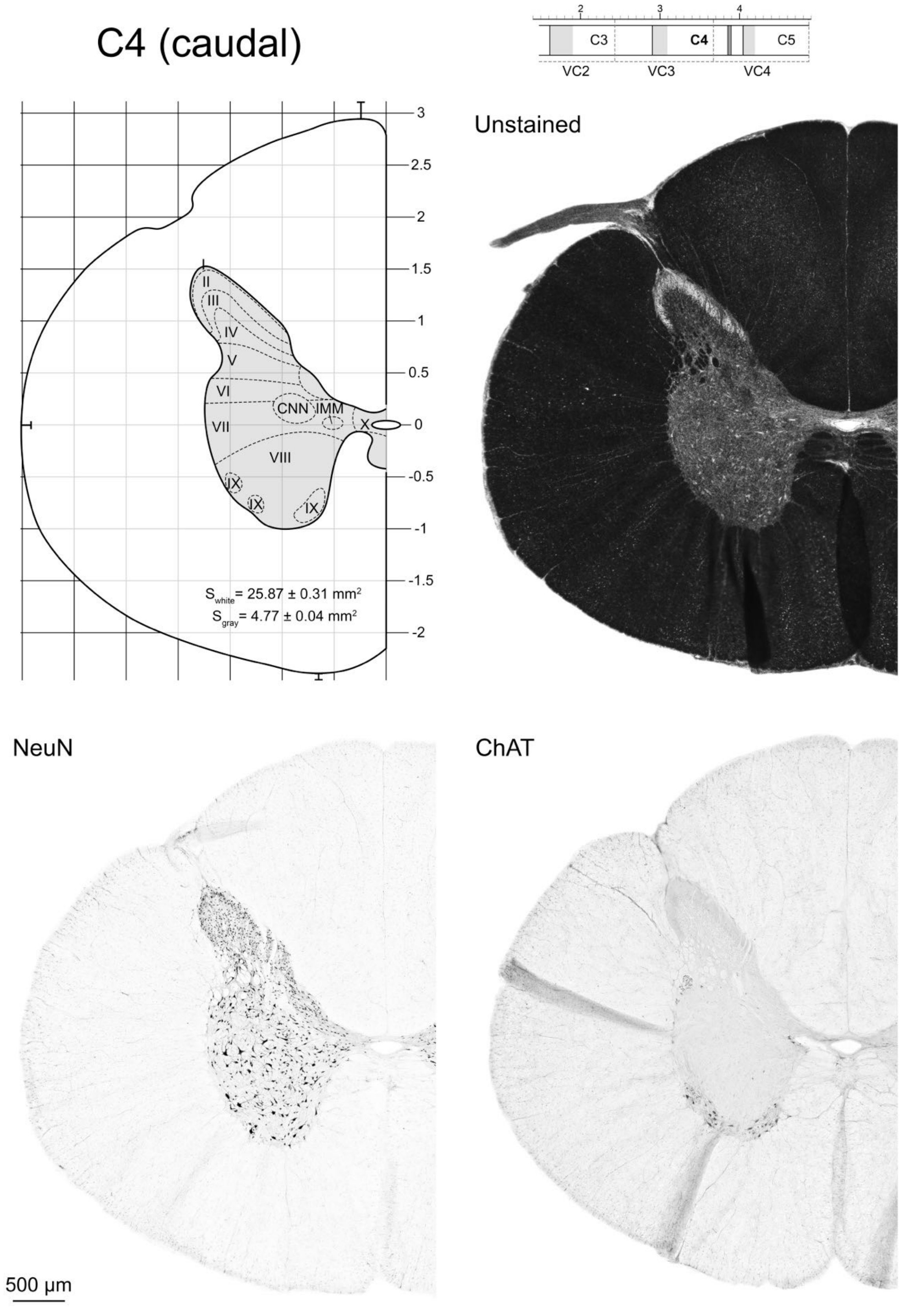

Supplementary Figure 12. Caudal part of C4 segment of the cat spinal cord.

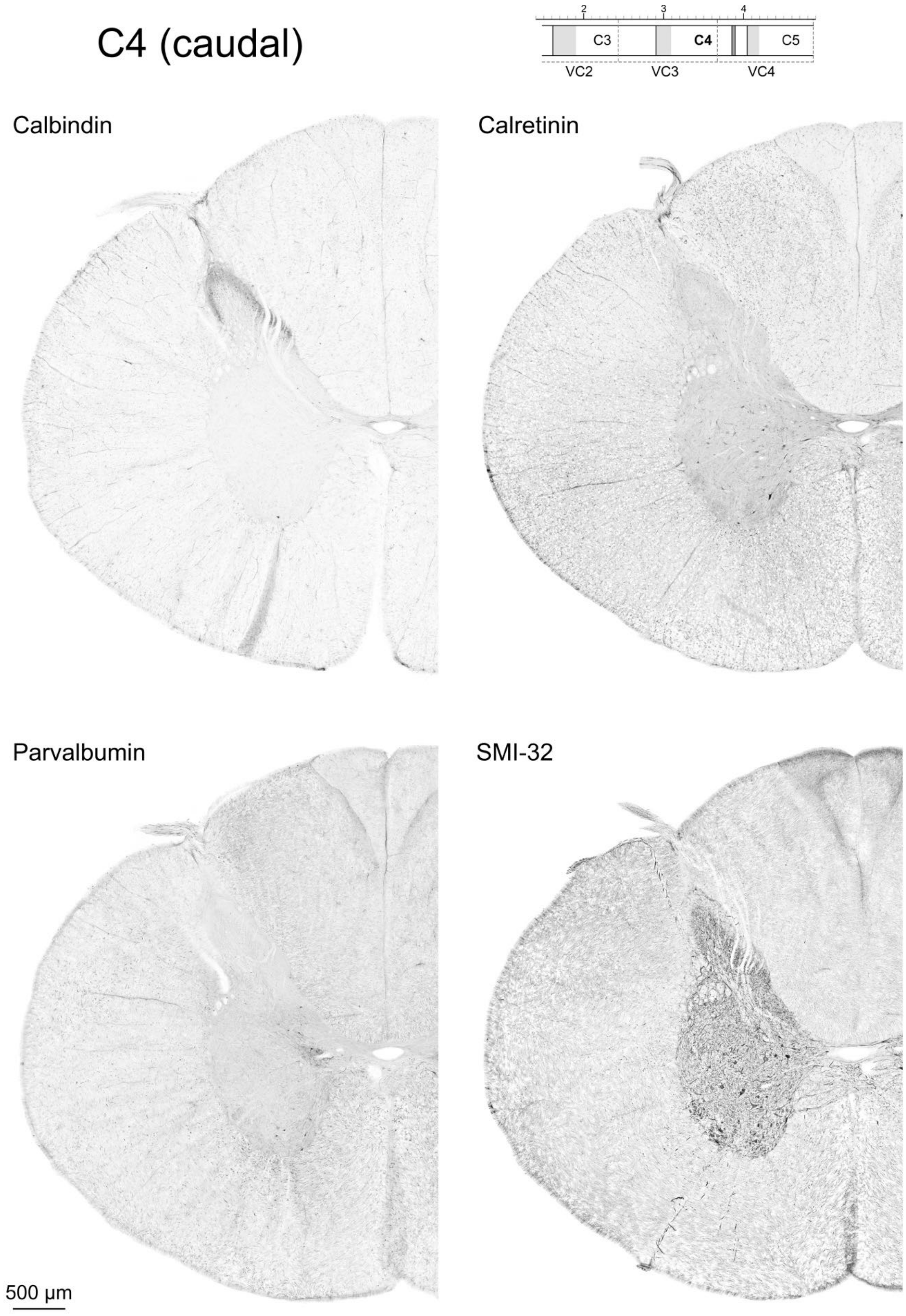

Supplementary Figure 12. Continued.

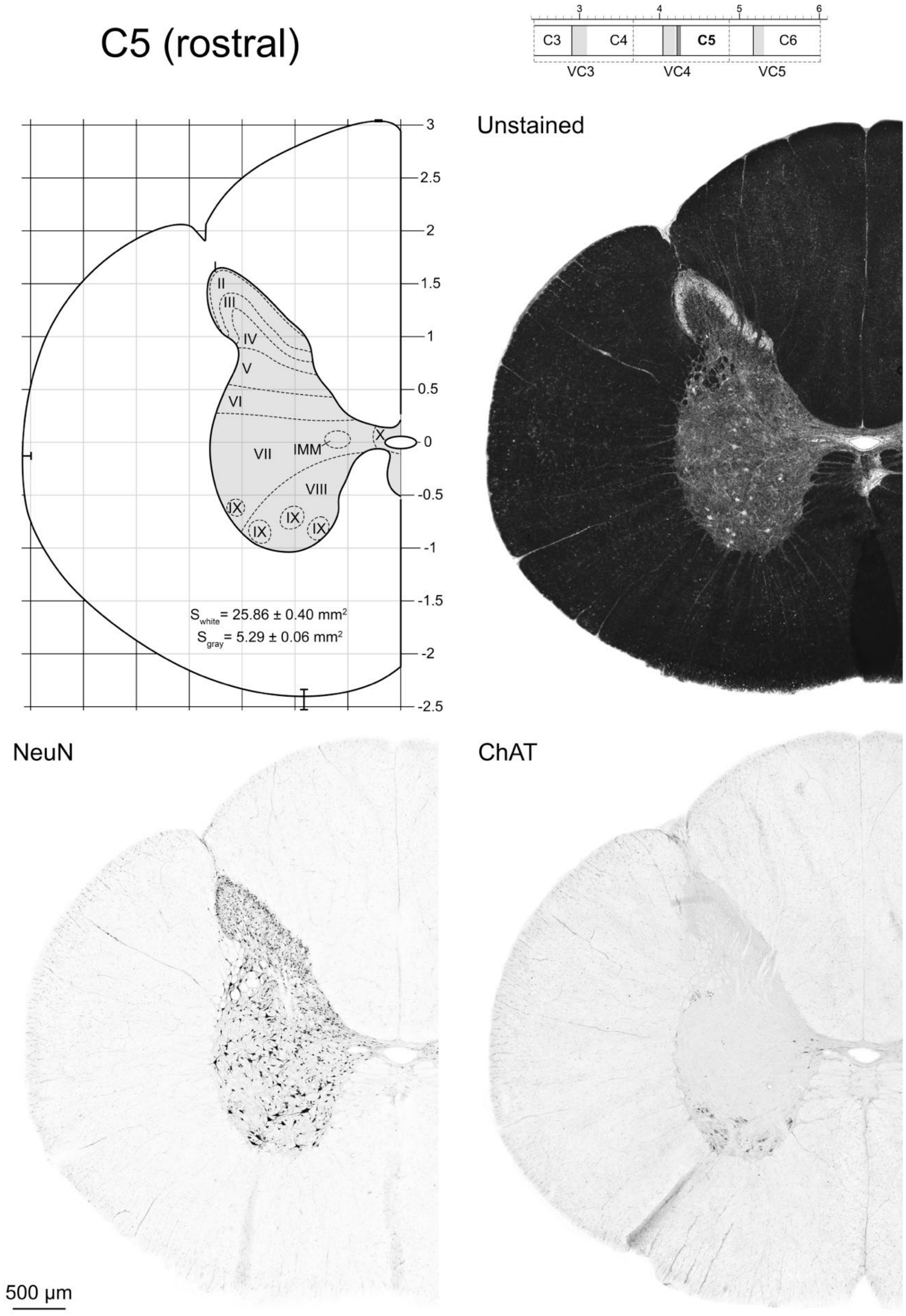

Supplementary Figure 13. Rostral part of C5 segment of the cat spinal cord.

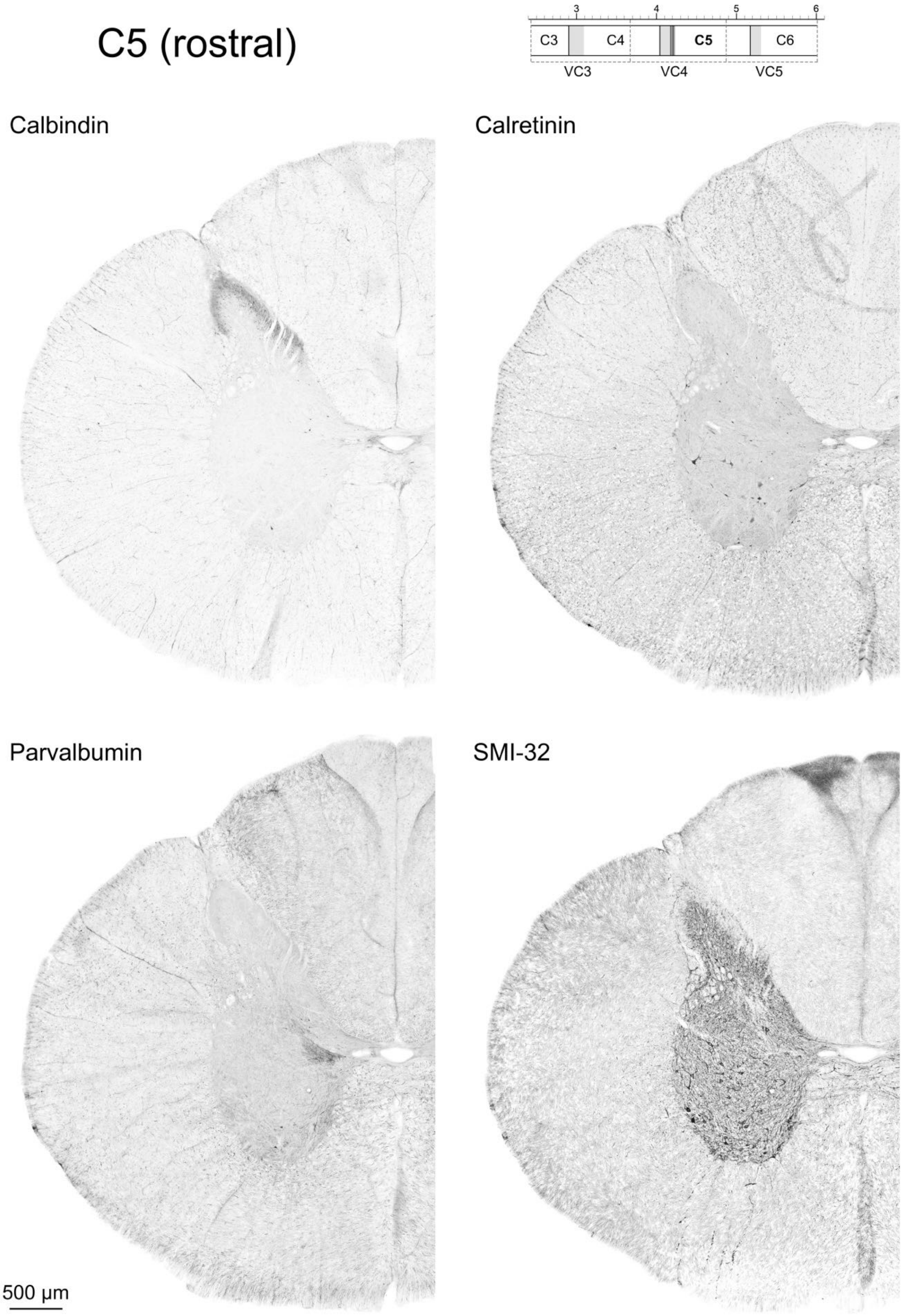

Supplementary Figure 13. Continued.

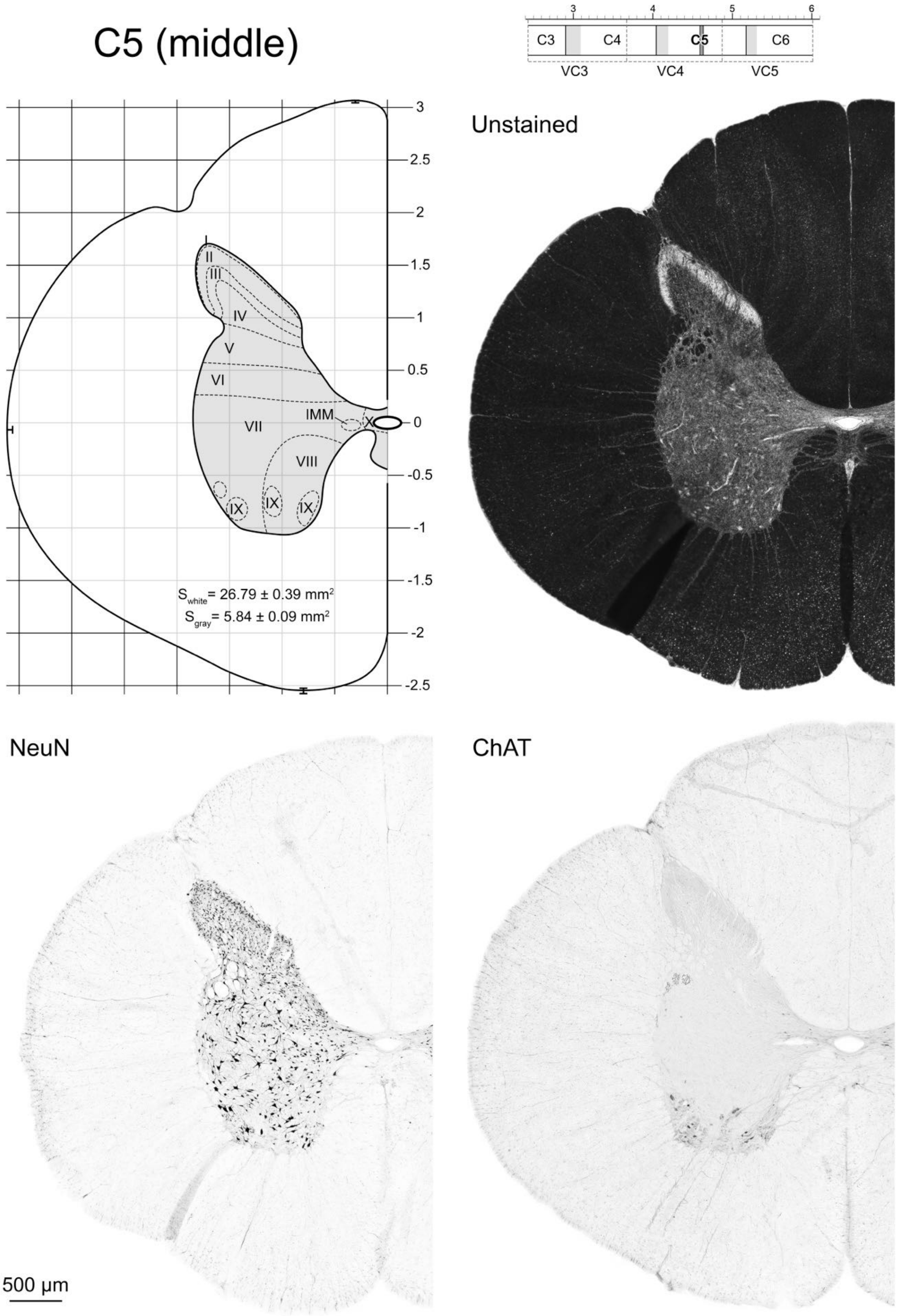

Supplementary Figure 14. Middle part of C5 segment of the cat spinal cord.

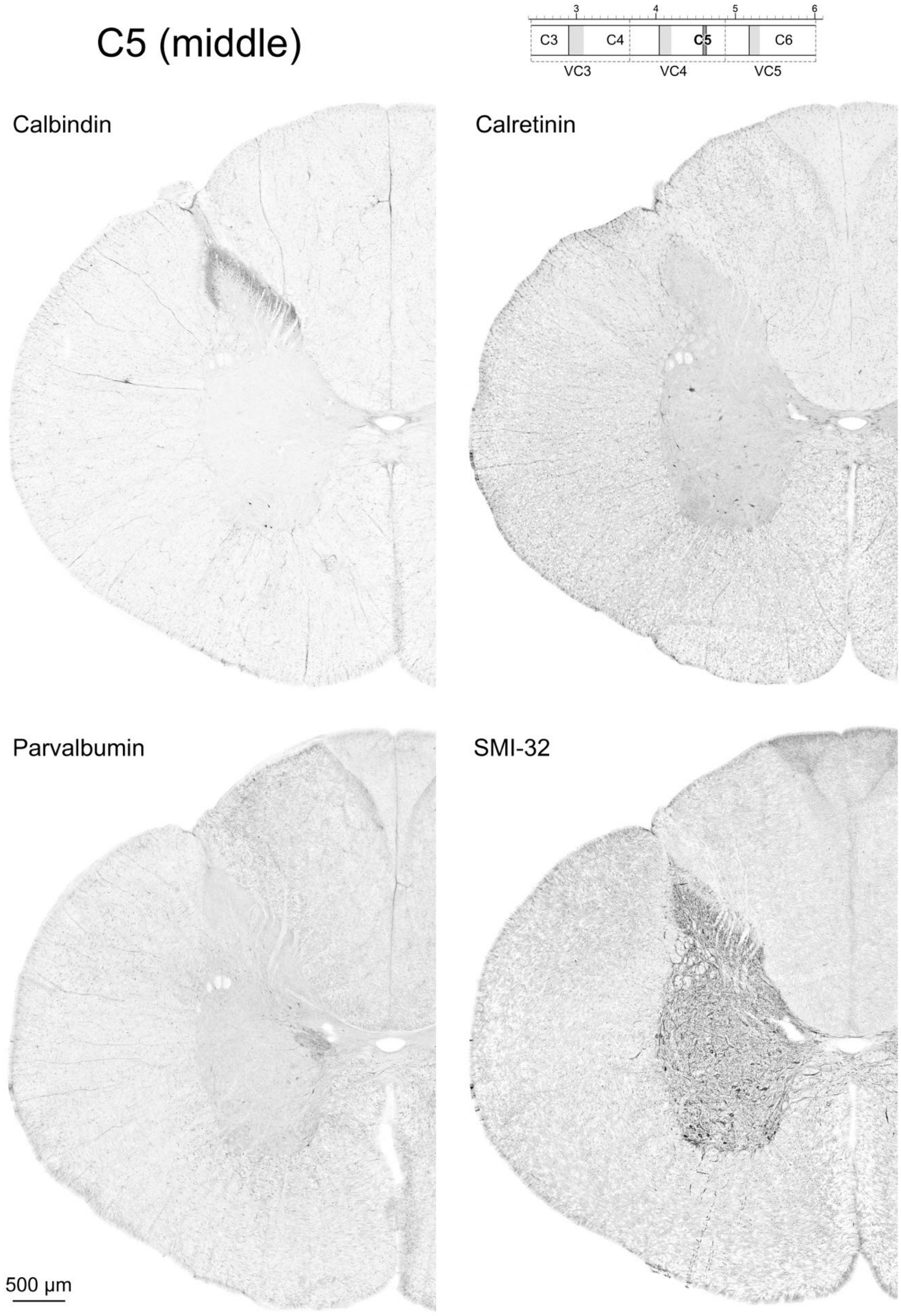

Supplementary Figure 14. Continued.

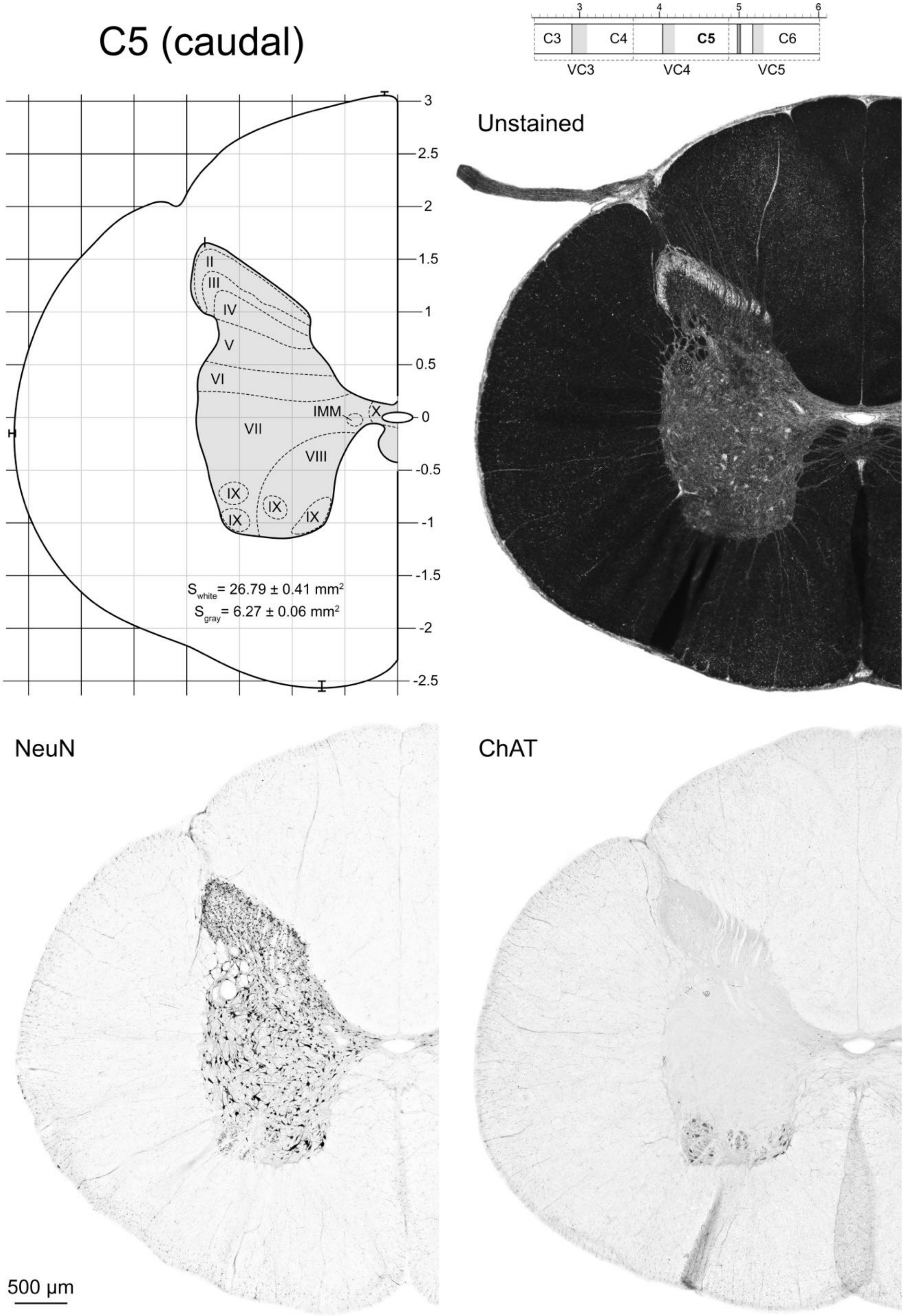

Supplementary Figure 15. Caudal part of C5 segment of the cat spinal cord.

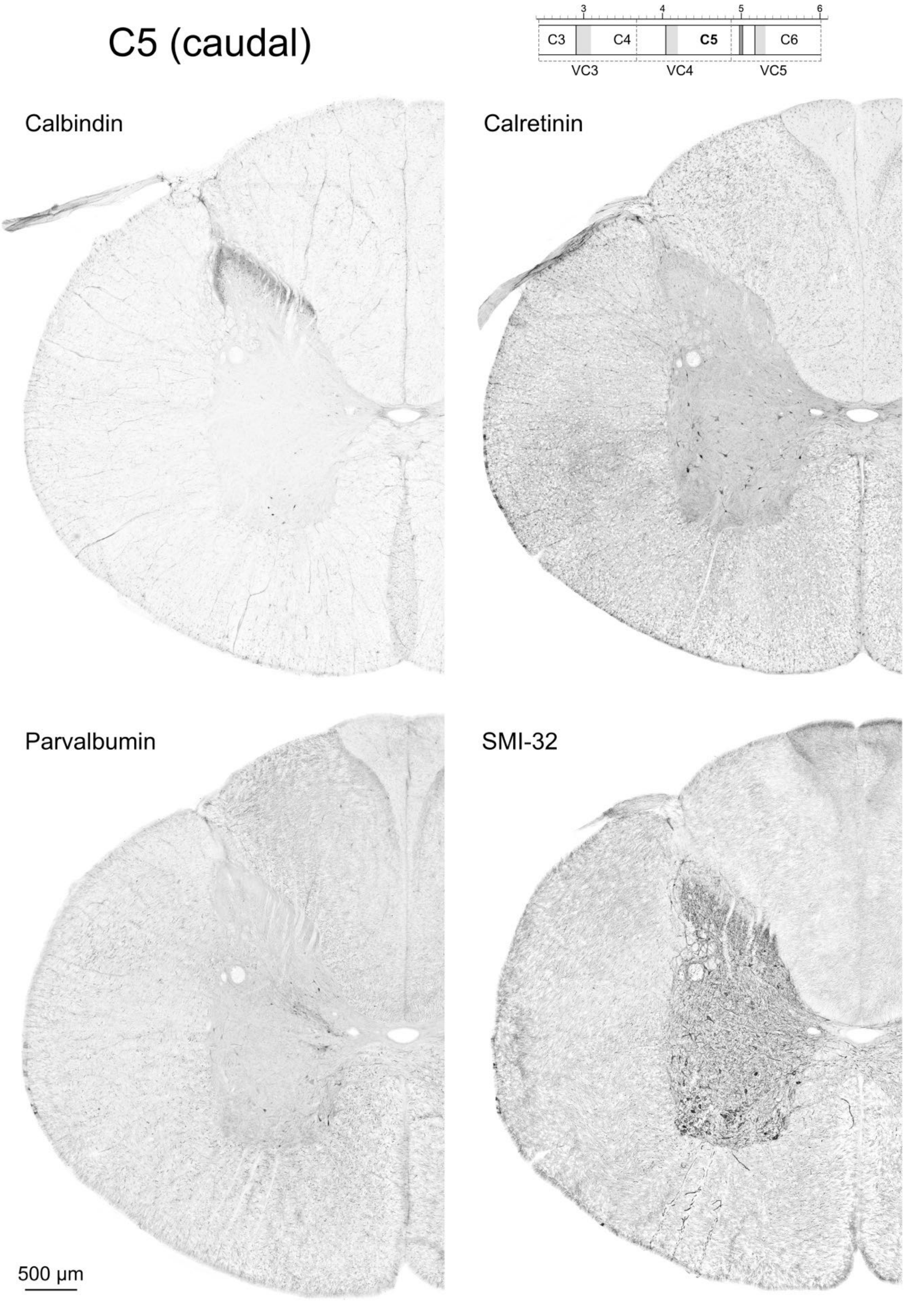

Supplementary Figure 15. Continued.

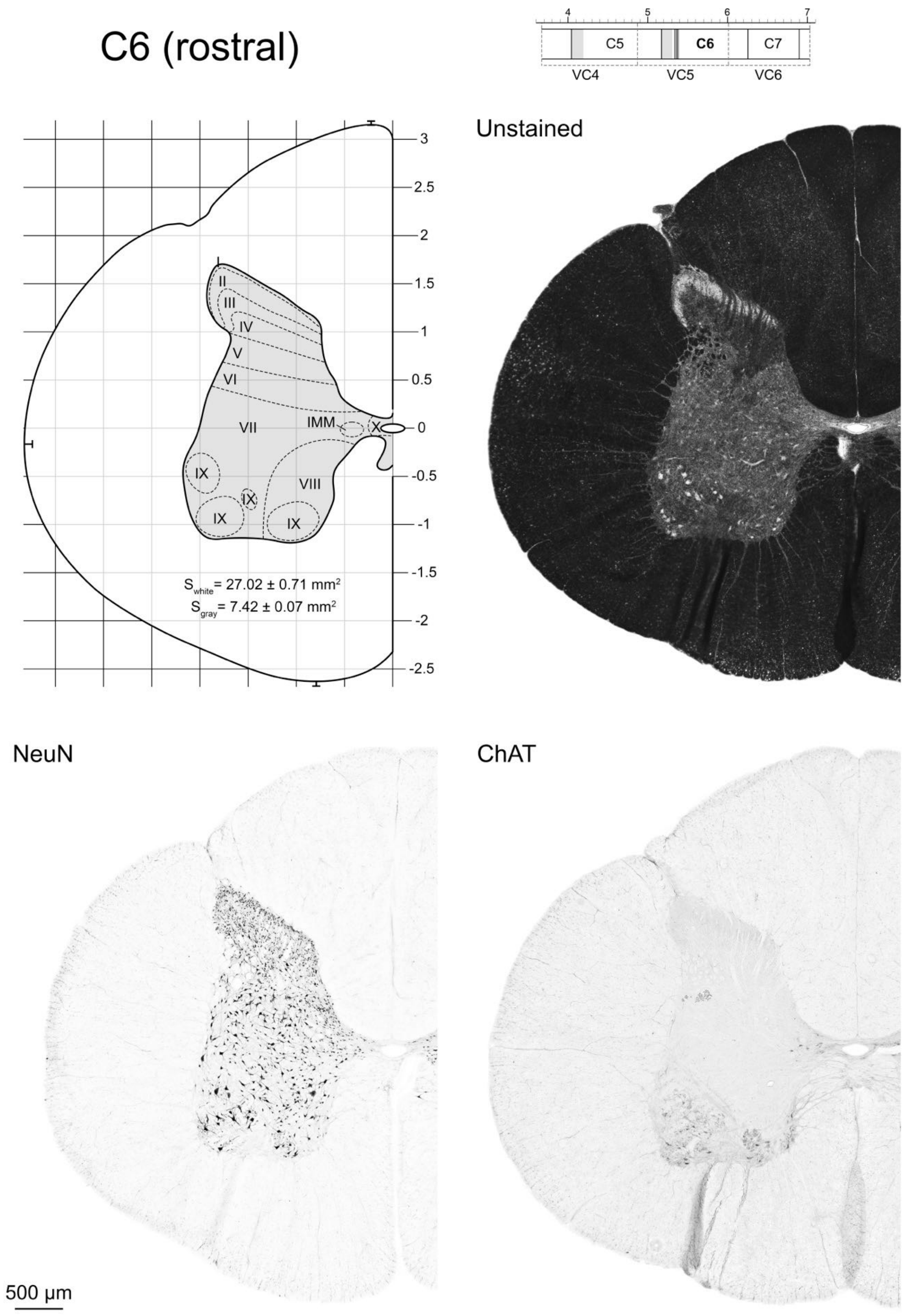

Supplementary Figure 16. Rostral part of C6 segment of the cat spinal cord.

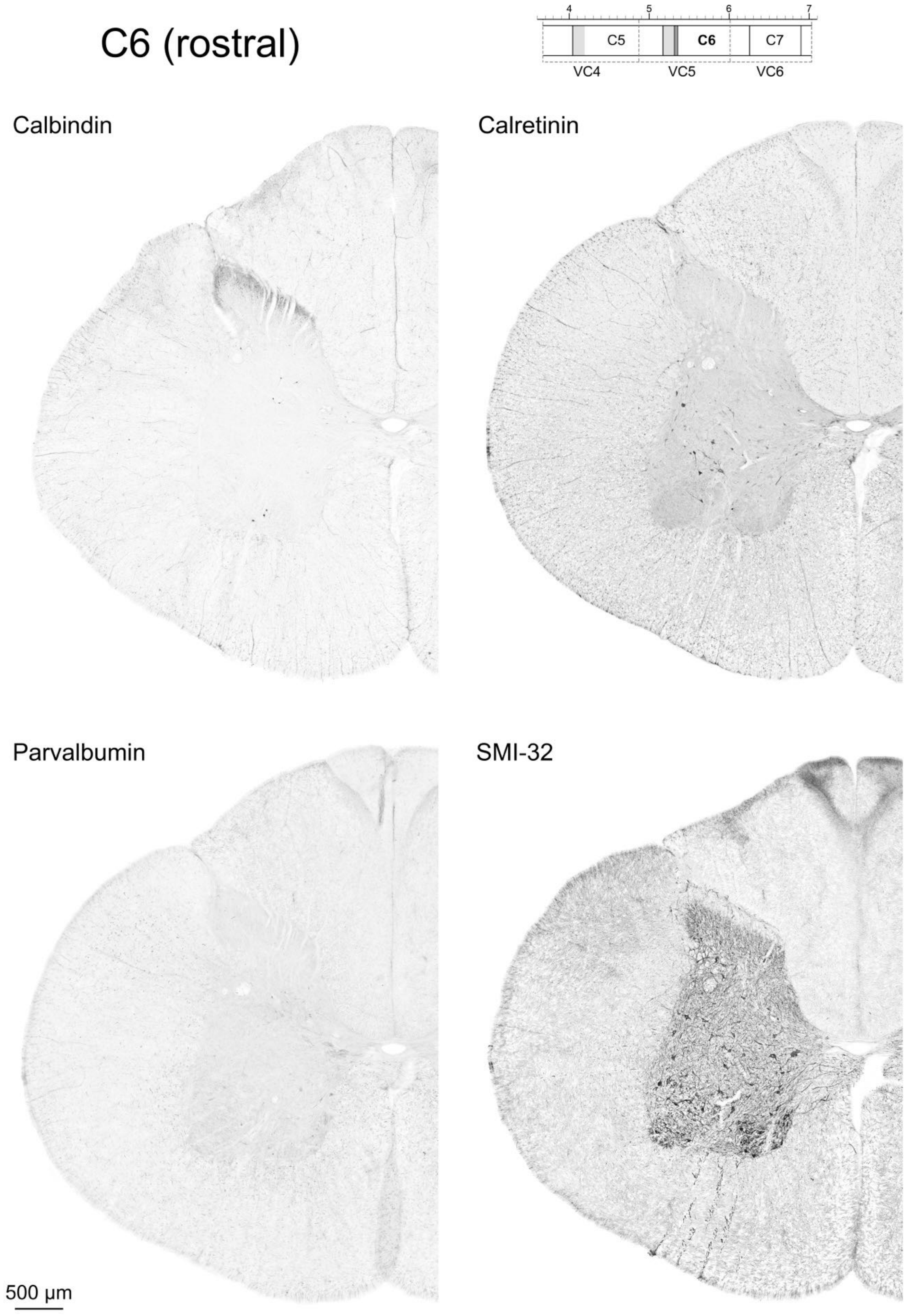

Supplementary Figure 16. Continued.

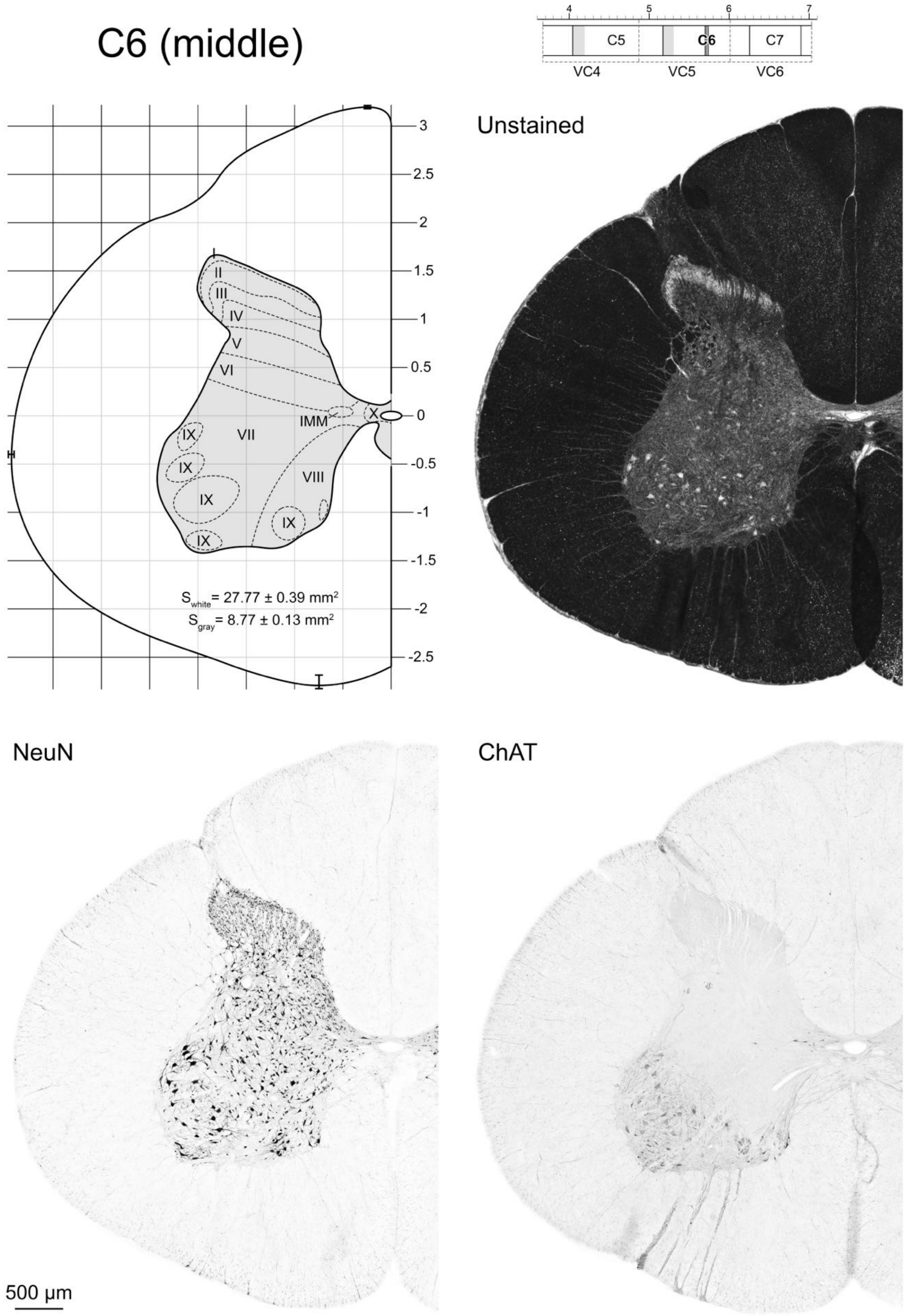

Supplementary Figure 17. Middle part of C6 segment of the cat spinal cord.

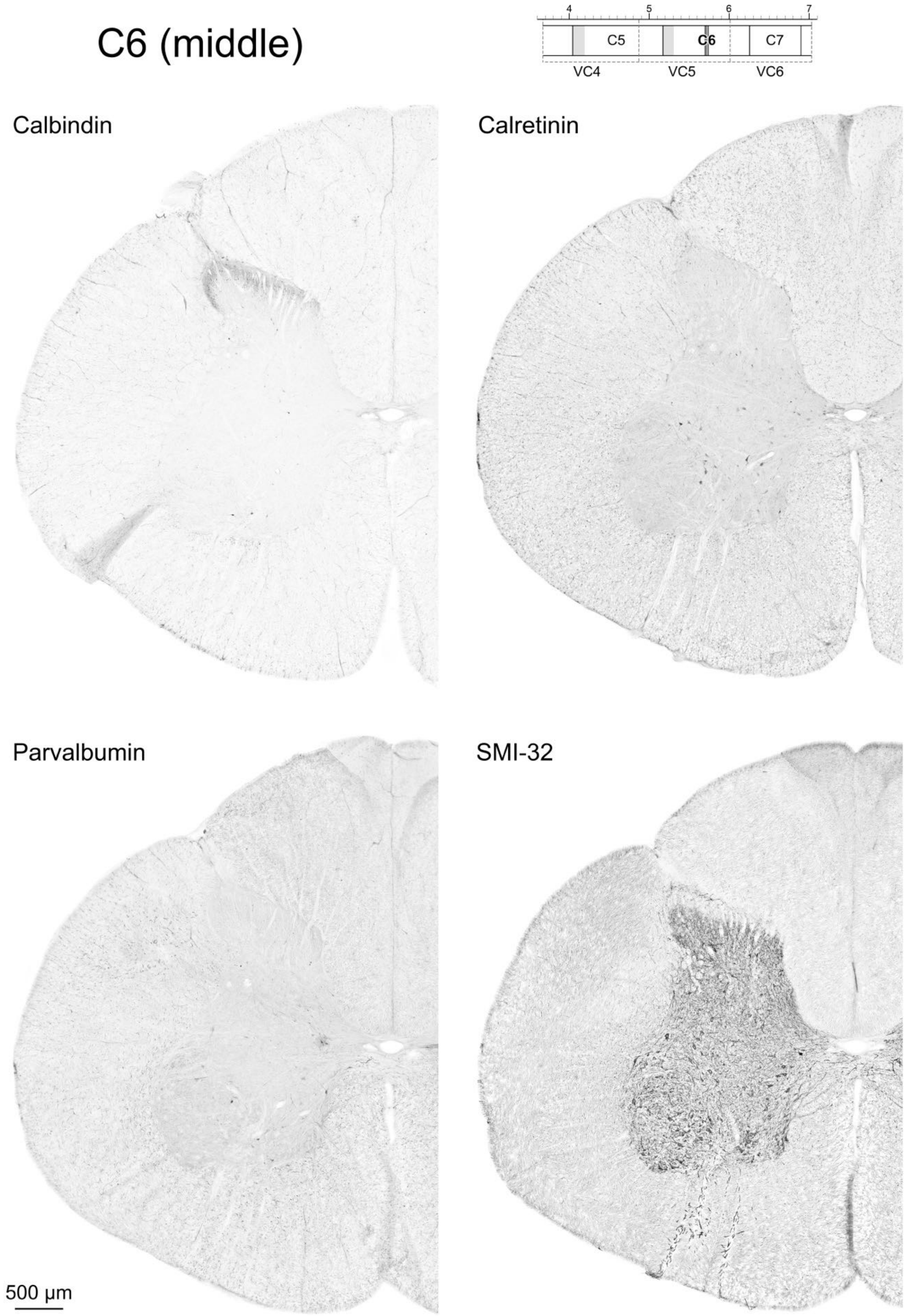

Supplementary Figure 17. Continued.

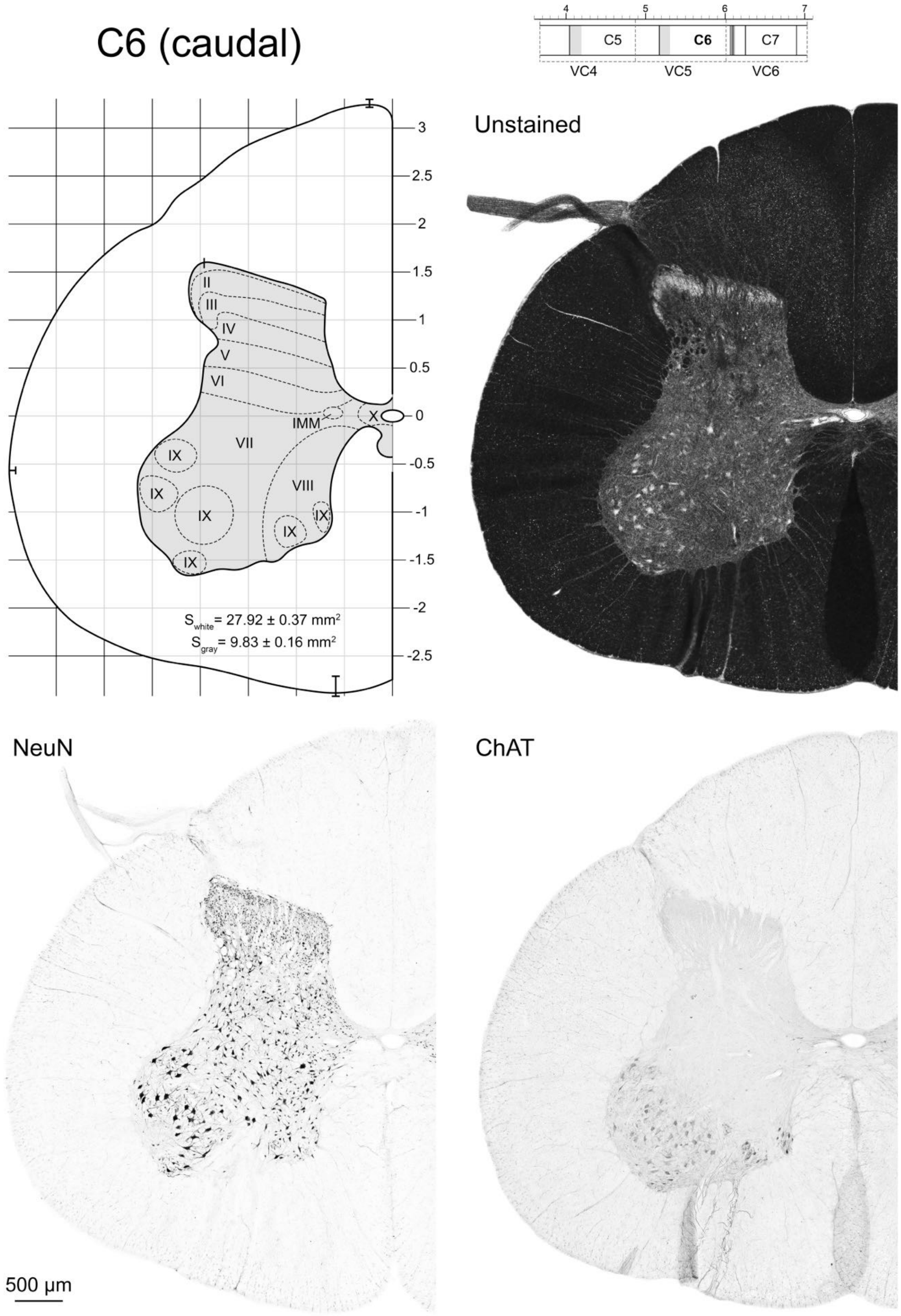

Supplementary Figure 18. Caudal part of C6 segment of the cat spinal cord.

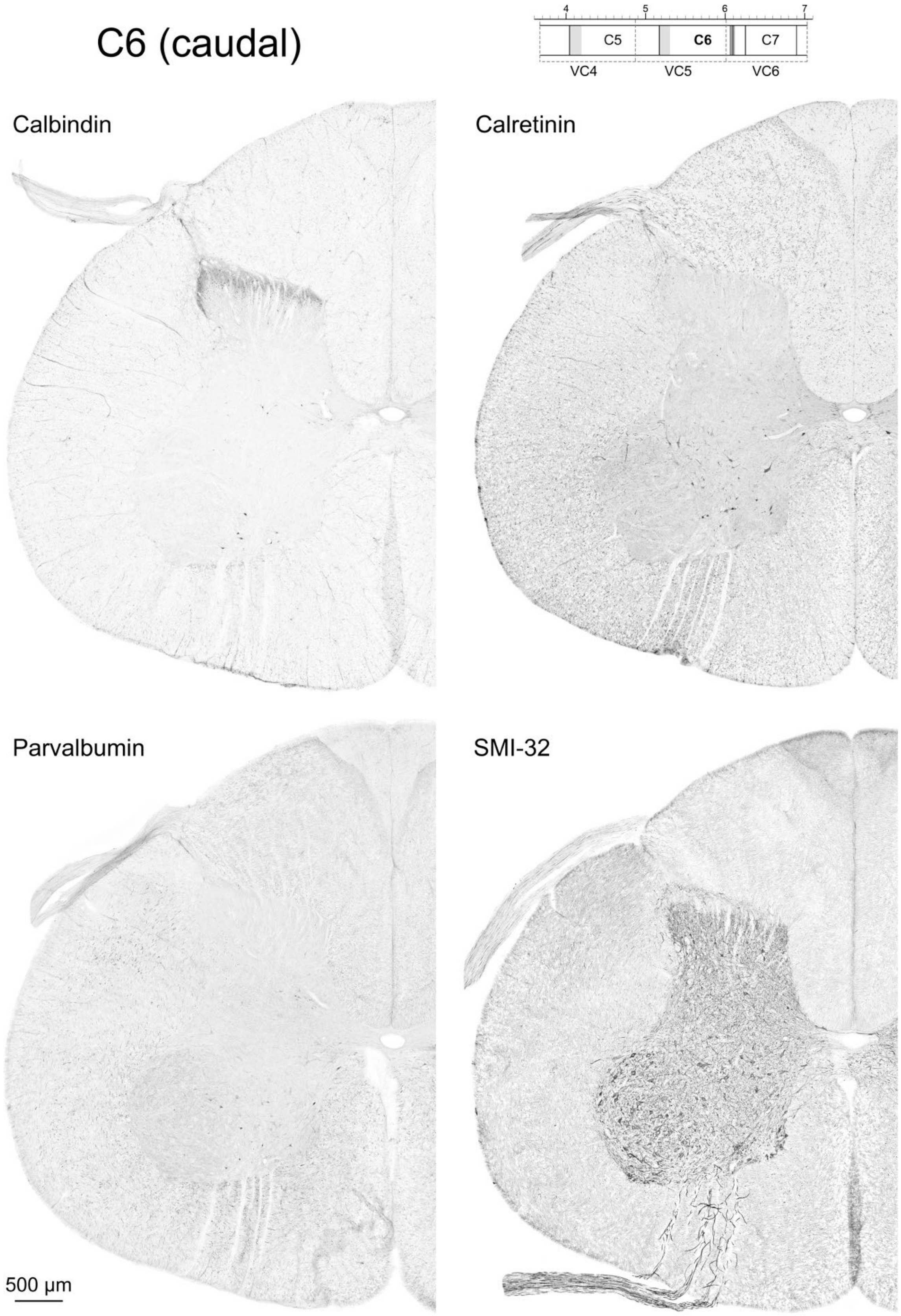

Supplementary Figure 18. Continued.

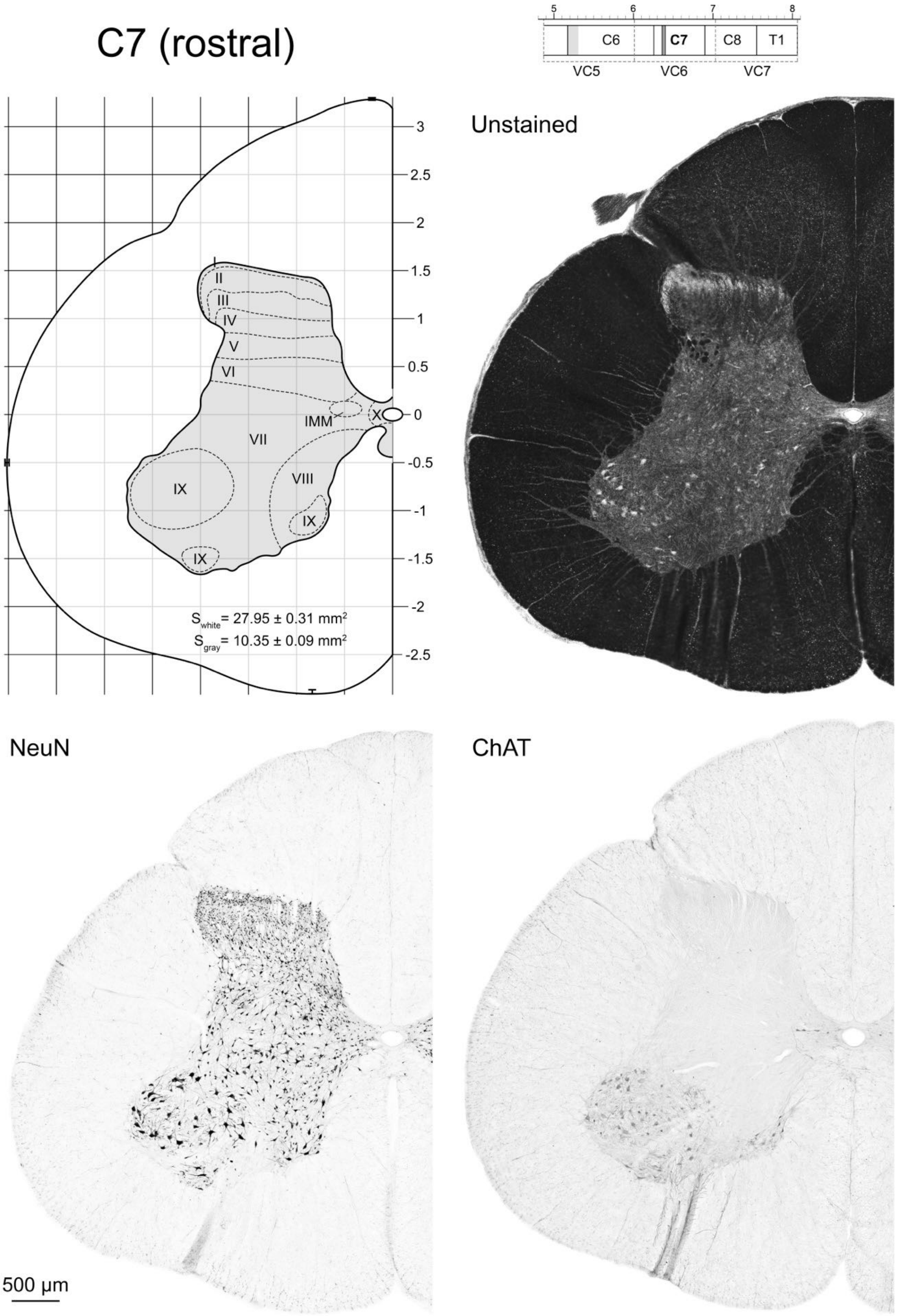

Supplementary Figure 19. Rostral part of C7 segment of the cat spinal cord.

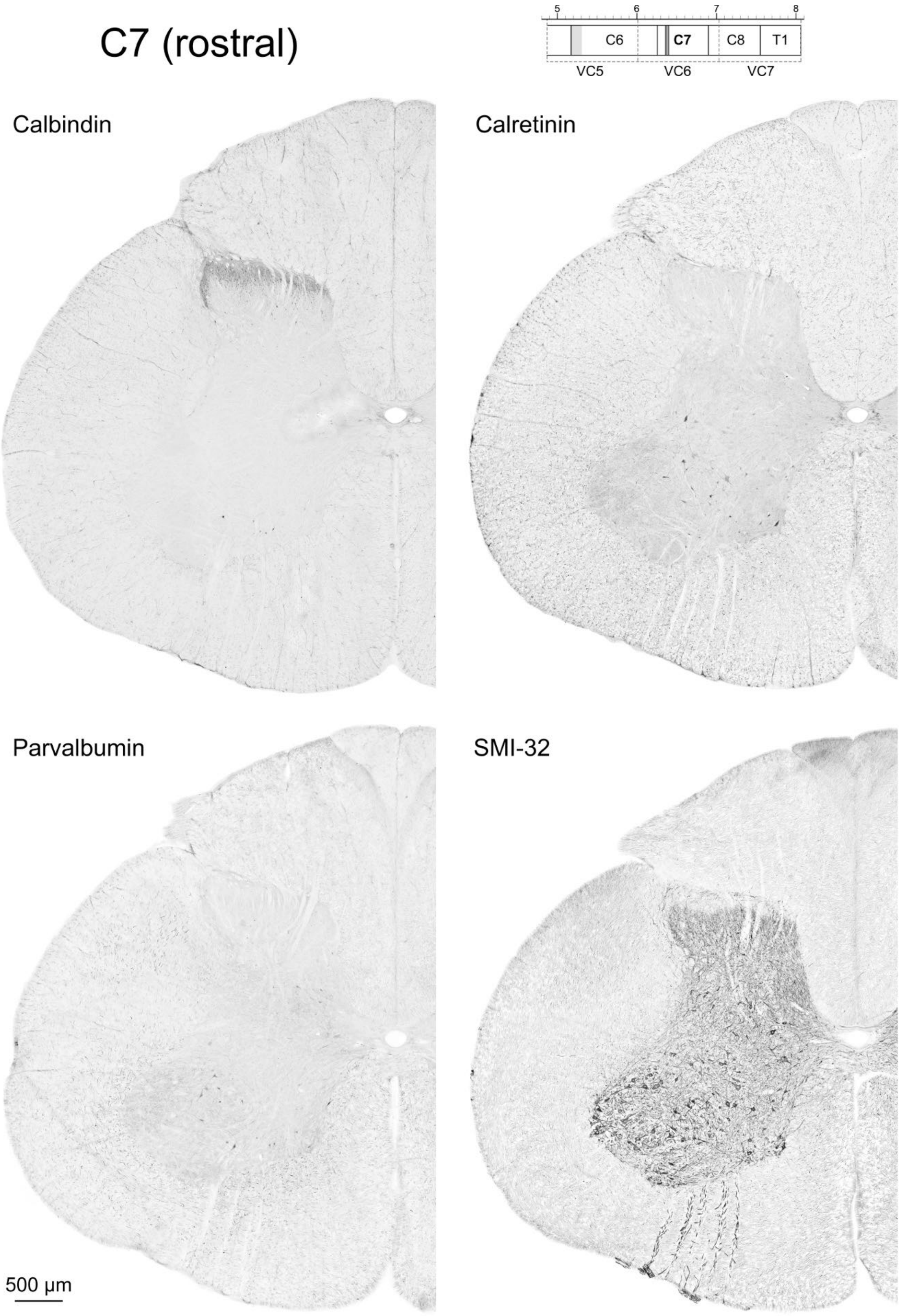

Supplementary Figure 19. Continued.

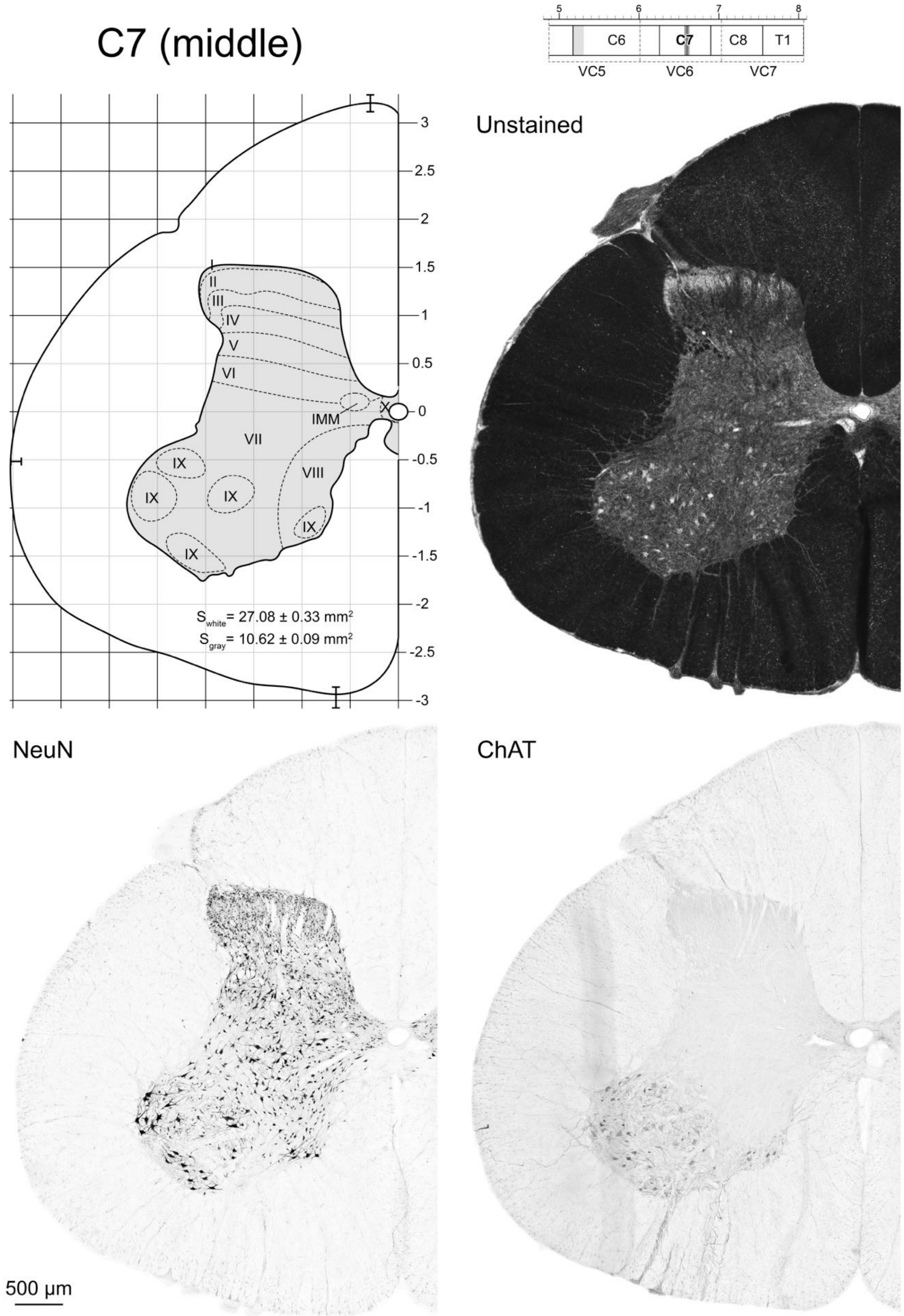

Supplementary Figure 20. Middle part of C7 segment of the cat spinal cord.

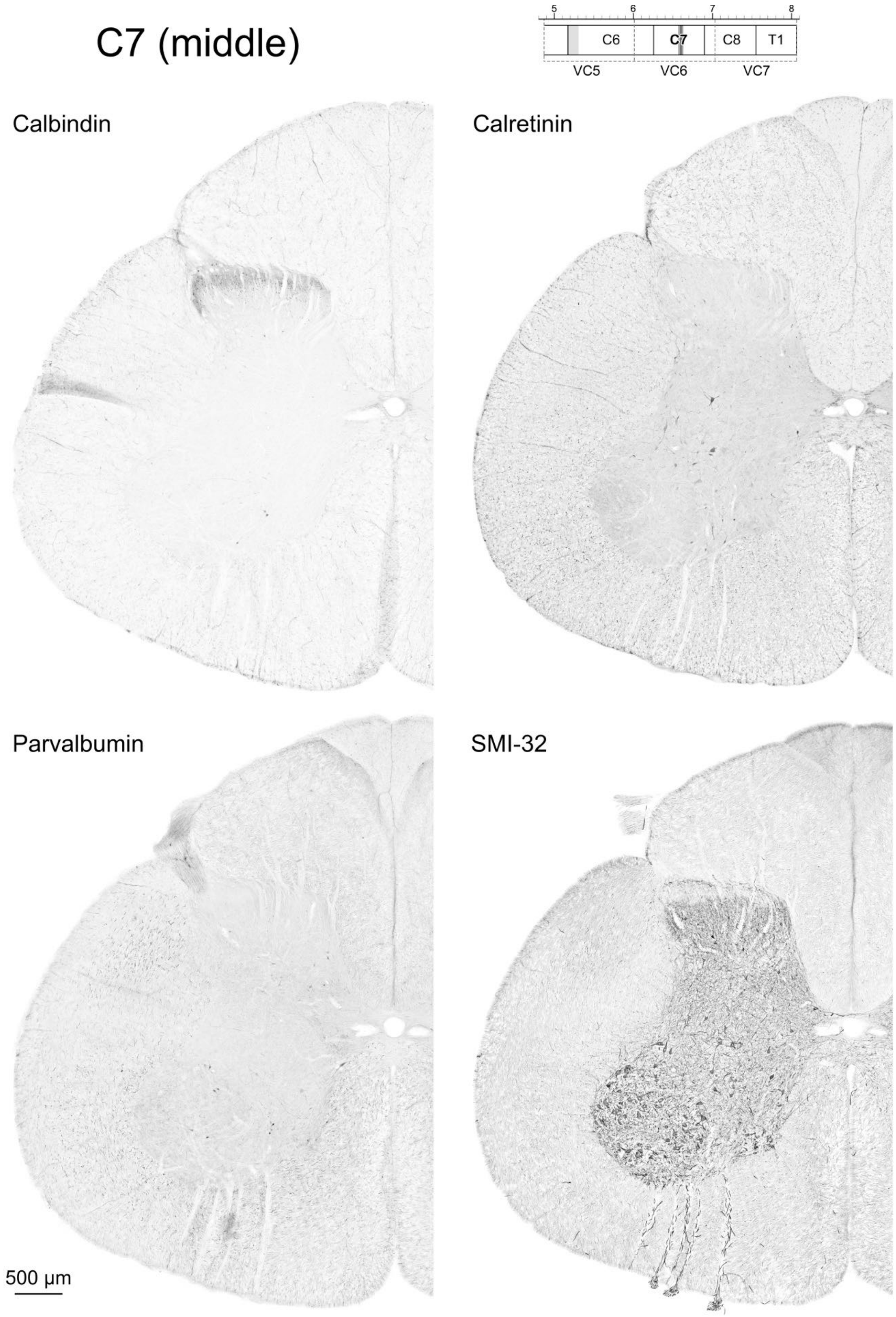

Supplementary Figure 20. Continued.

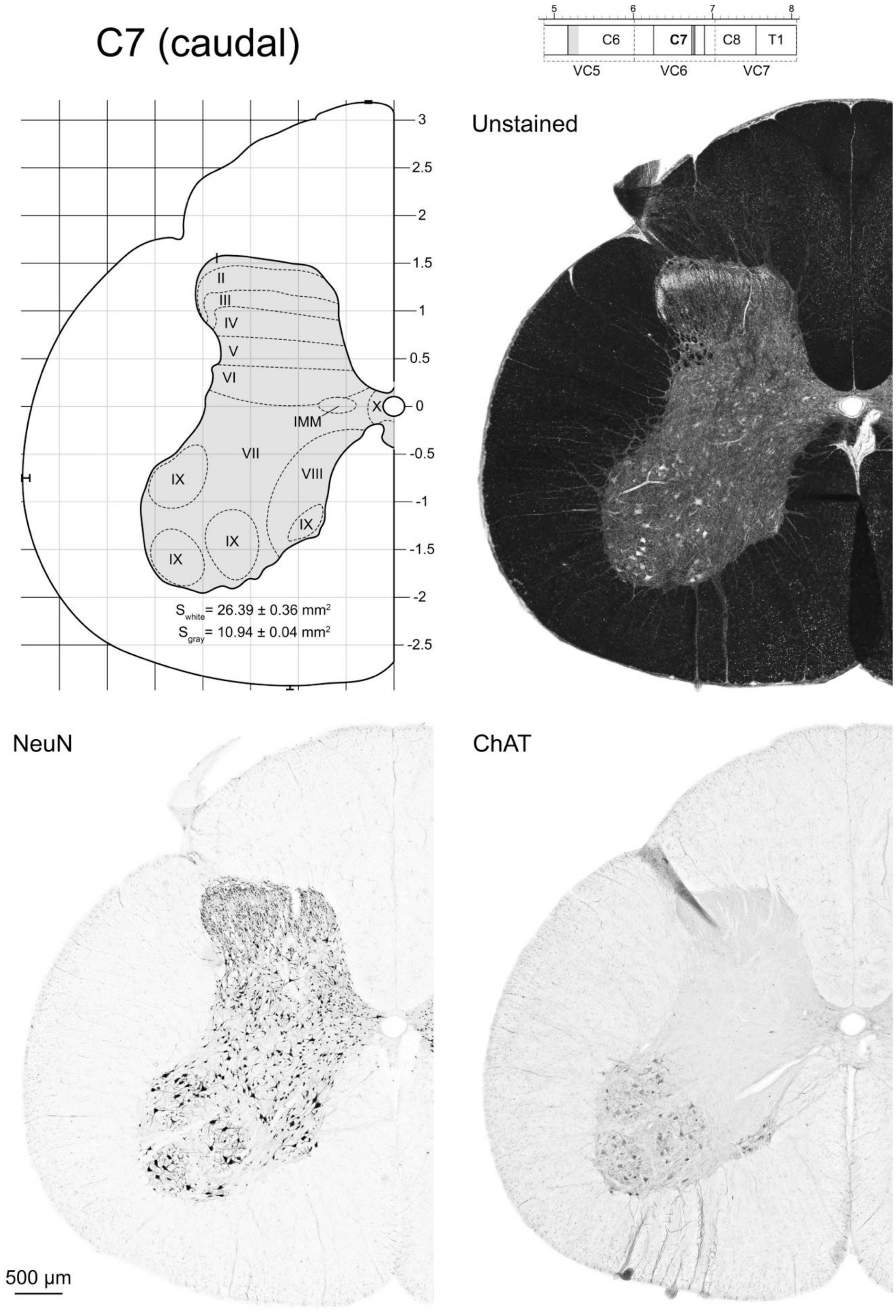

Supplementary Figure 21. Caudal part of C7 segment of the cat spinal cord.

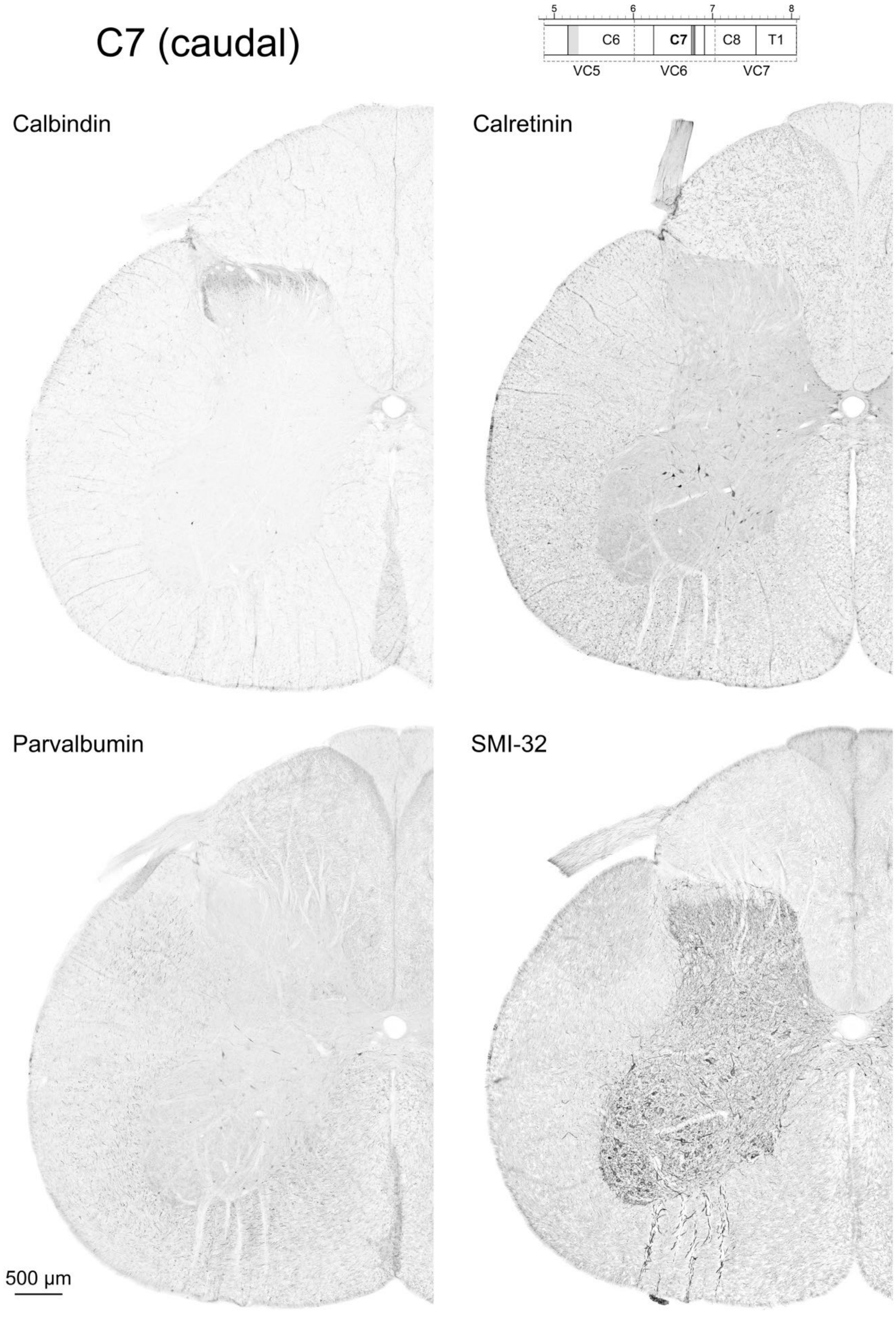

Supplementary Figure 21. Continued.

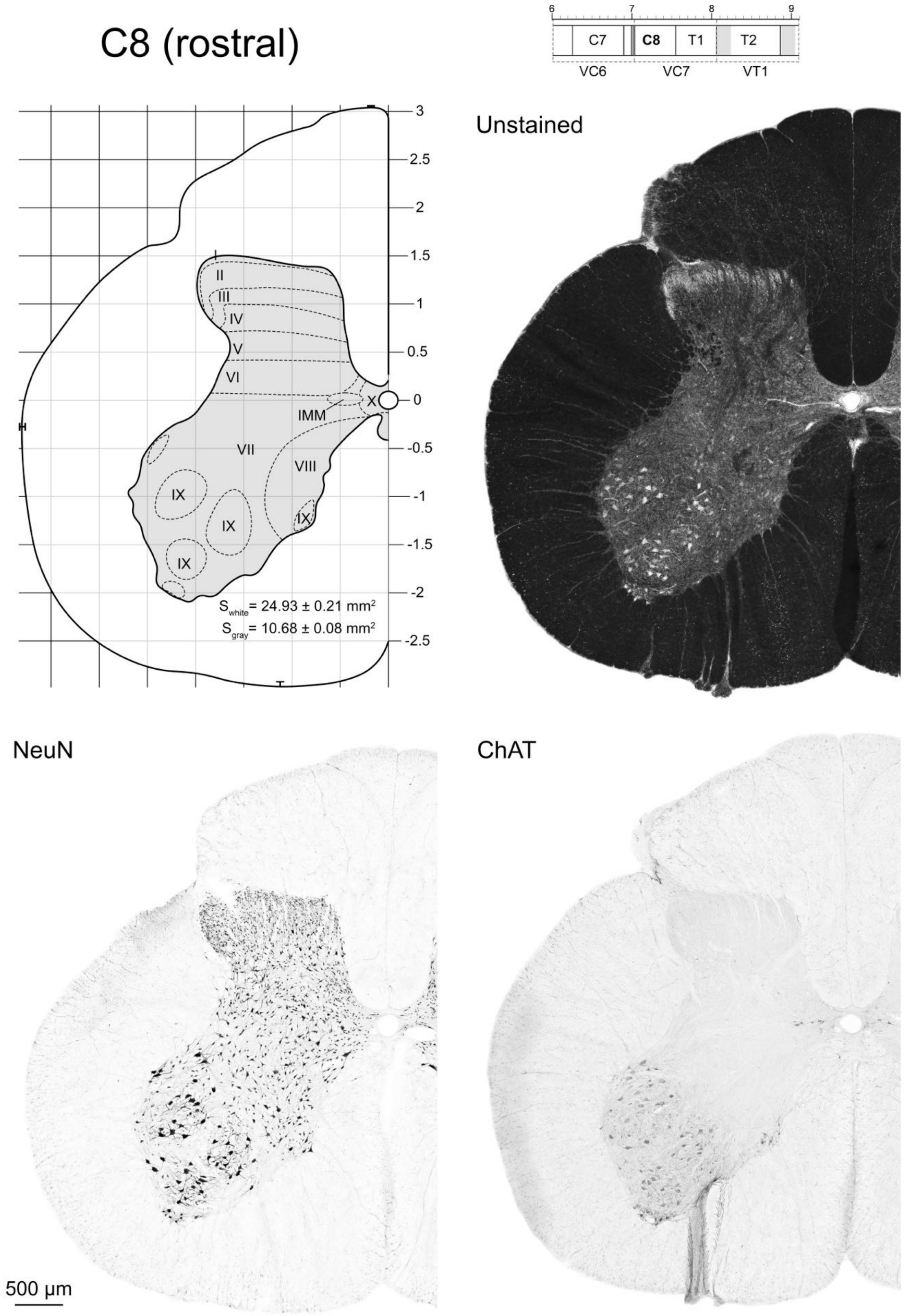

Supplementary Figure 22. Rostral part of C8 segment of the cat spinal cord.

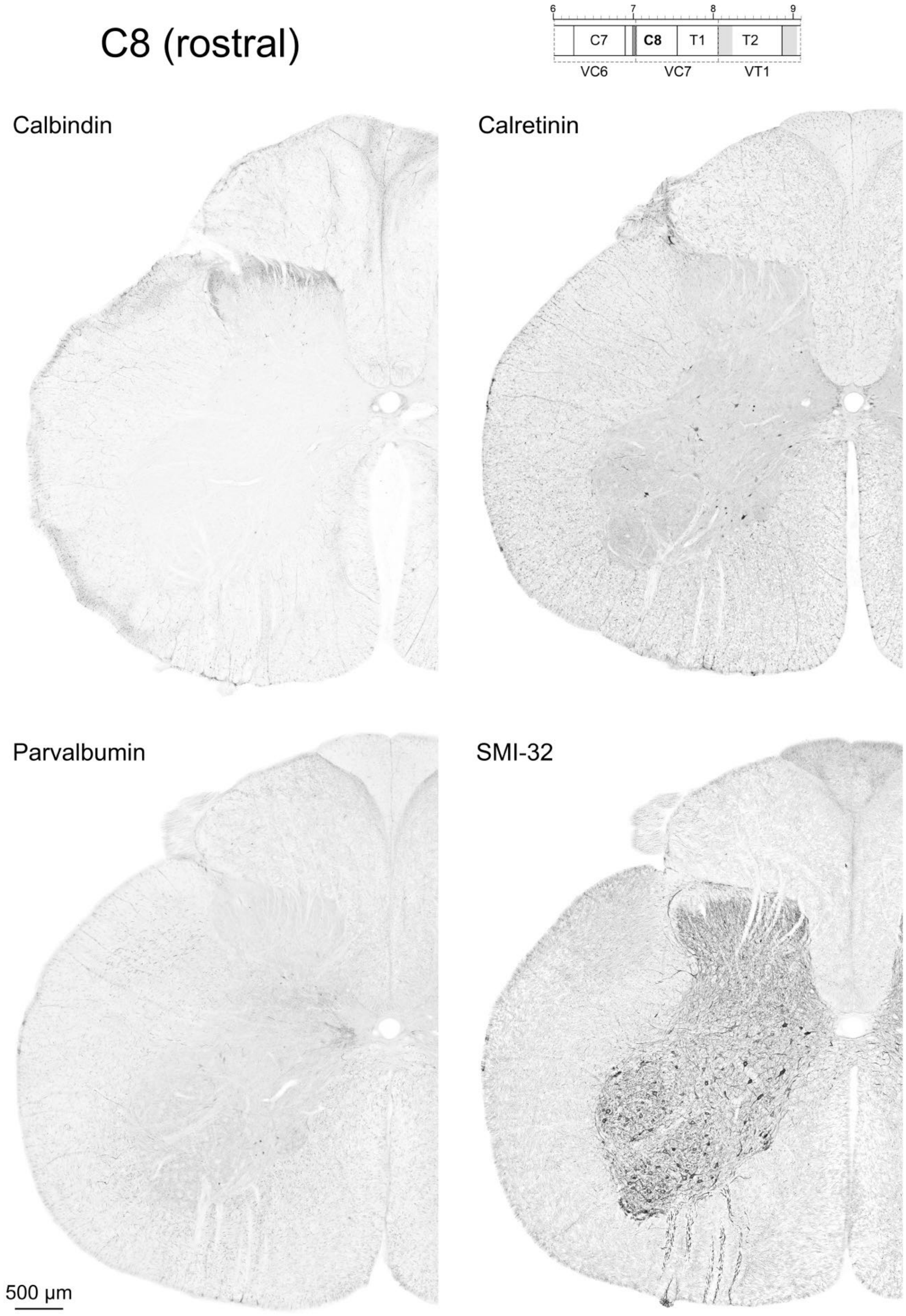

Supplementary Figure 22. Continued.

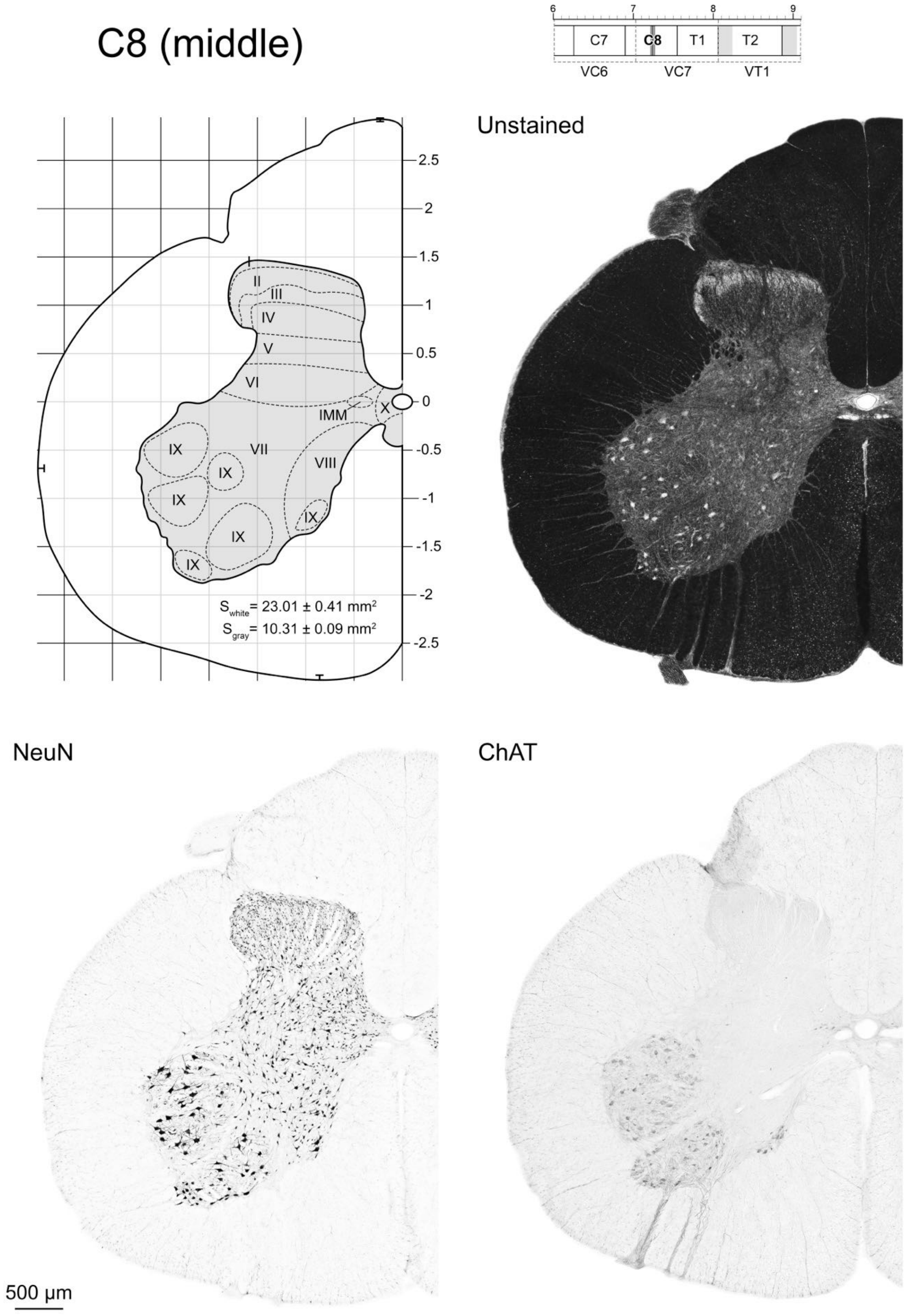

Supplementary Figure 23. Middle part of C8 segment of the cat spinal cord.

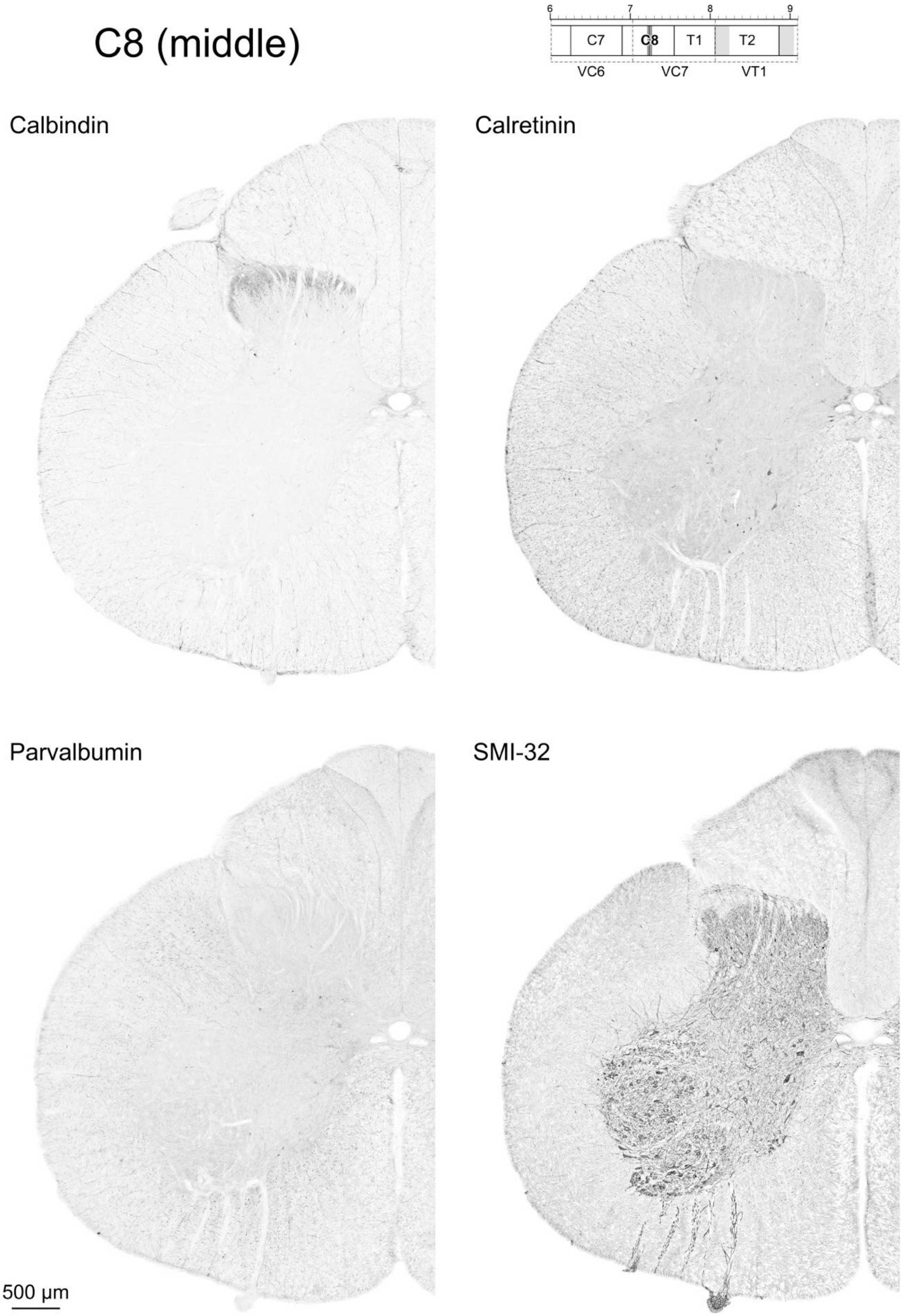

Supplementary Figure 23. Continued.

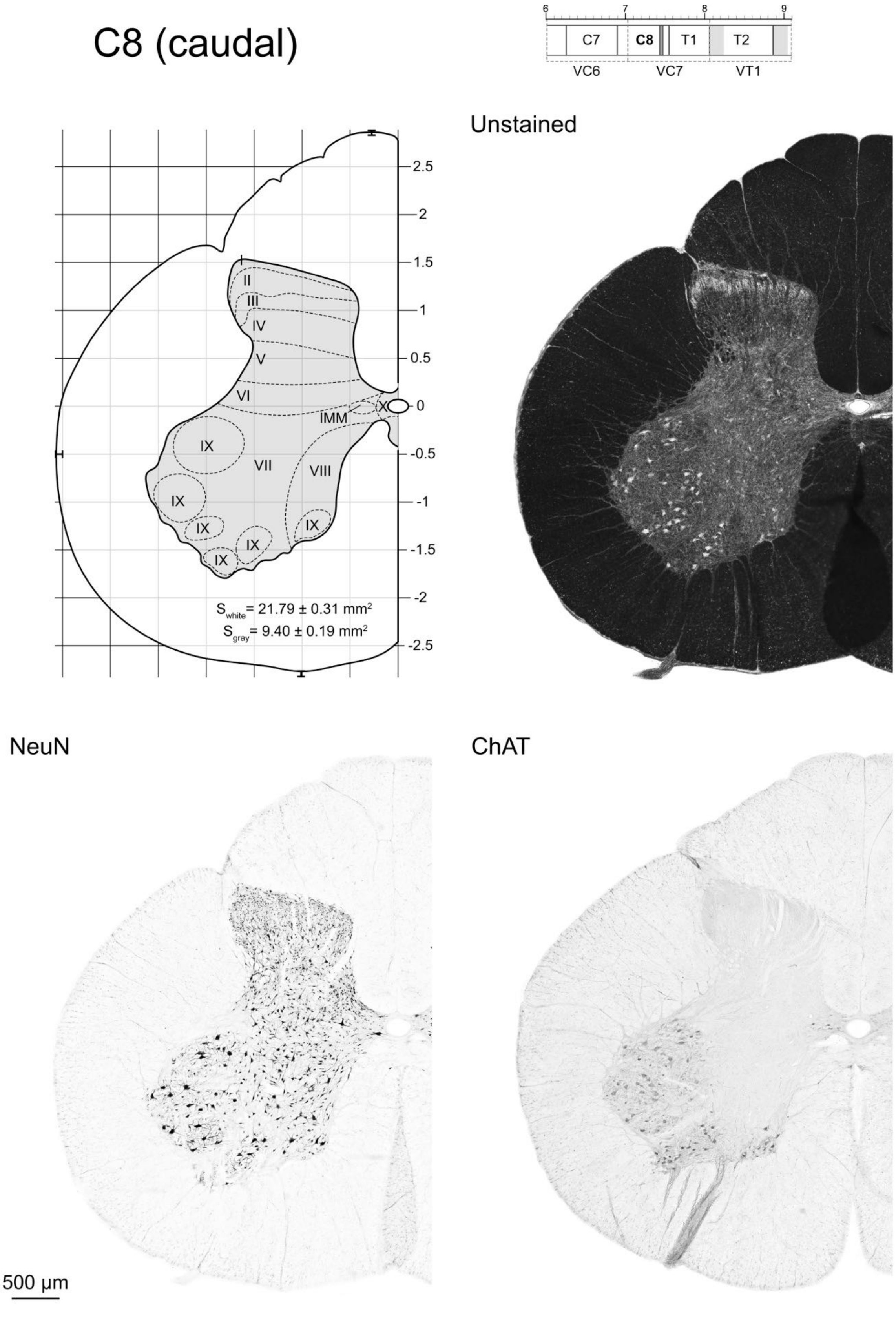

Supplementary Figure 24. Caudal part of C8 segment of the cat spinal cord.

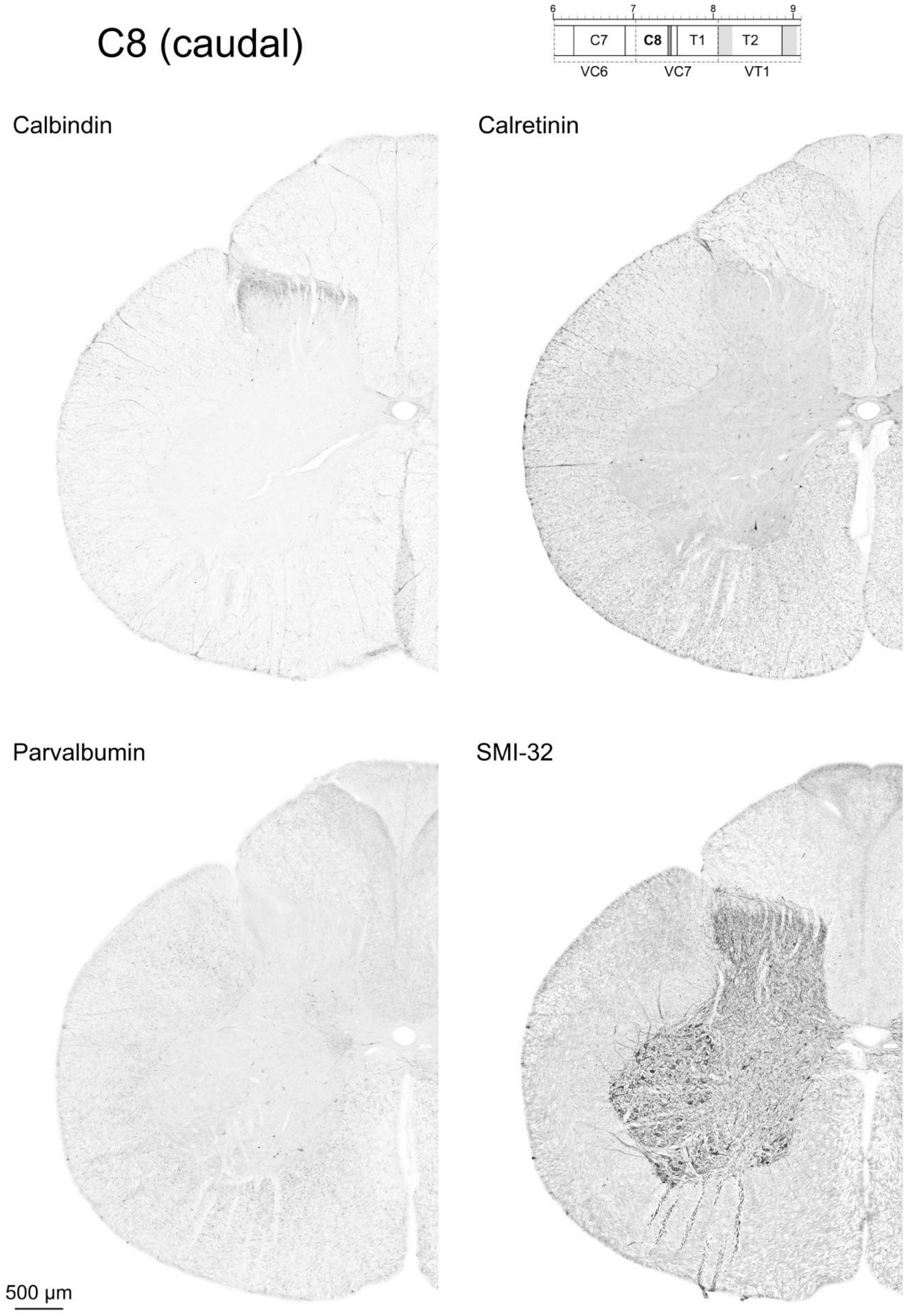

Supplementary Figure 24. Continued.
