# Supplementary material for: Neurochemical atlas of the cat spinal cord
Source: Front Neuroanat. 2022 Oct 19;16:1034395. doi: 10.3389/fnana.2022.1034395 (PMC9627295; doi:10.3389/fnana.2022.1034395)
Supplement: Supplementary file 2 [file Data_Sheet_2.PDF]

Supplementary Material

1 Abbreviations

I – lamina I

II – lamina II

III – lamina III

IV – lamina IV

V – lamina V

VI – lamina VI

VII – lamina VII

VIII – lamina VIII

IX – lamina IX

X – lamina X

CN – Clarke's Nucleus

IC – Intercalated Nucleus

IMM – Intermediomedial Nucleus

IML – Intermediolateral Nucleus

S_{white} – area of the white matter

S_{gray} – area of the gray matter

2 Supplementary Figures

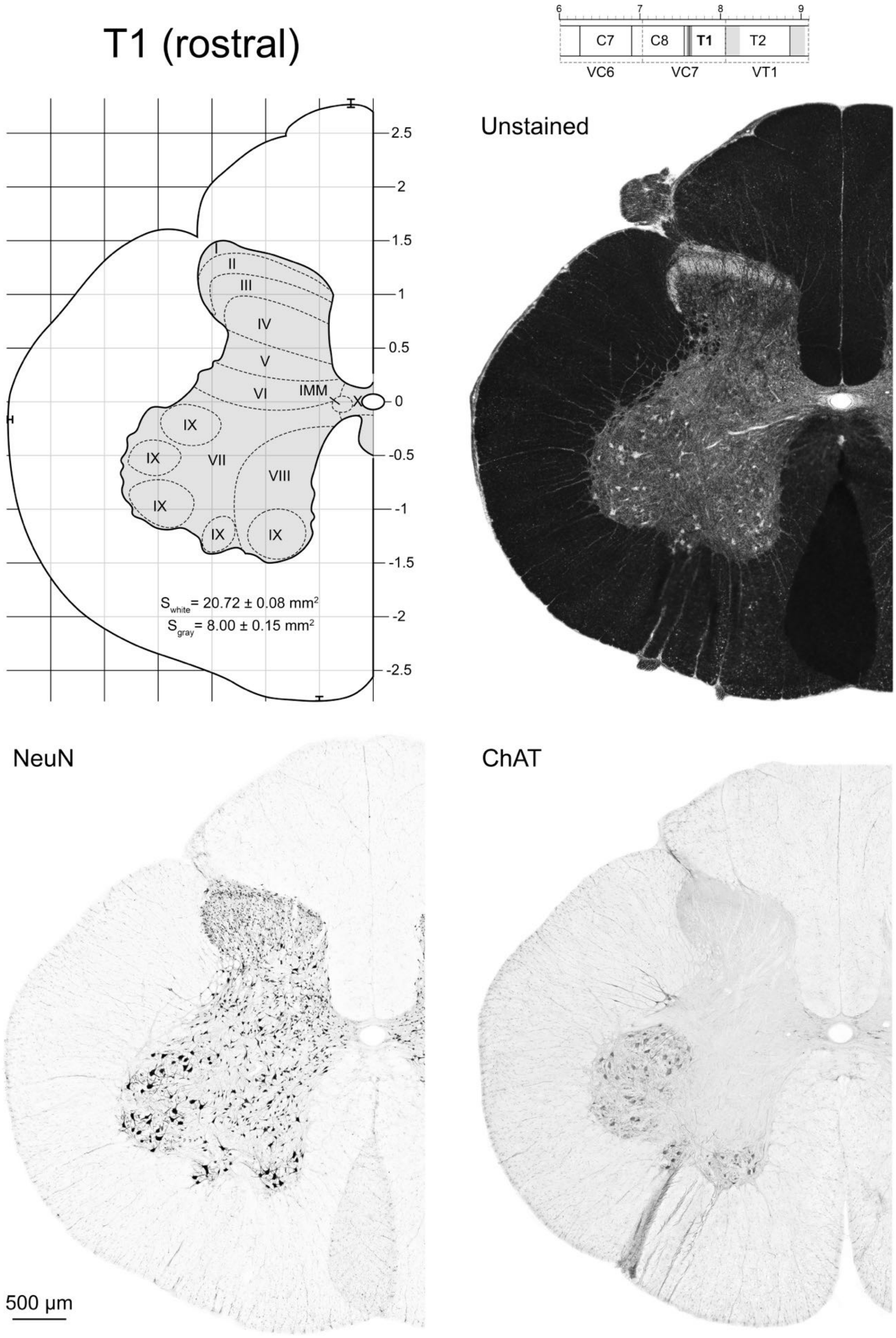

Supplementary Figure 1. Rostral part of T1 segment of the cat spinal cord.

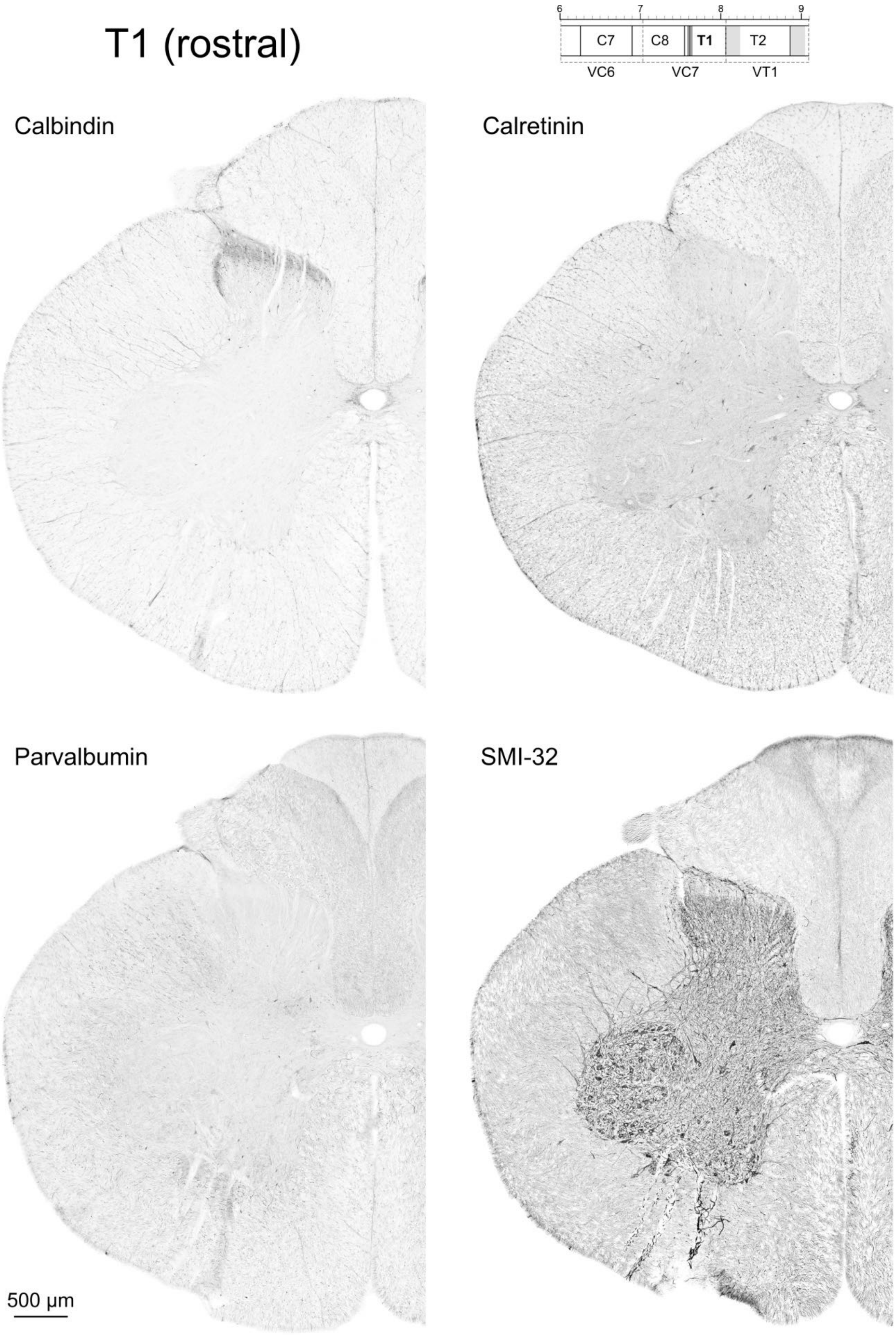

Supplementary Figure 1. Continued.

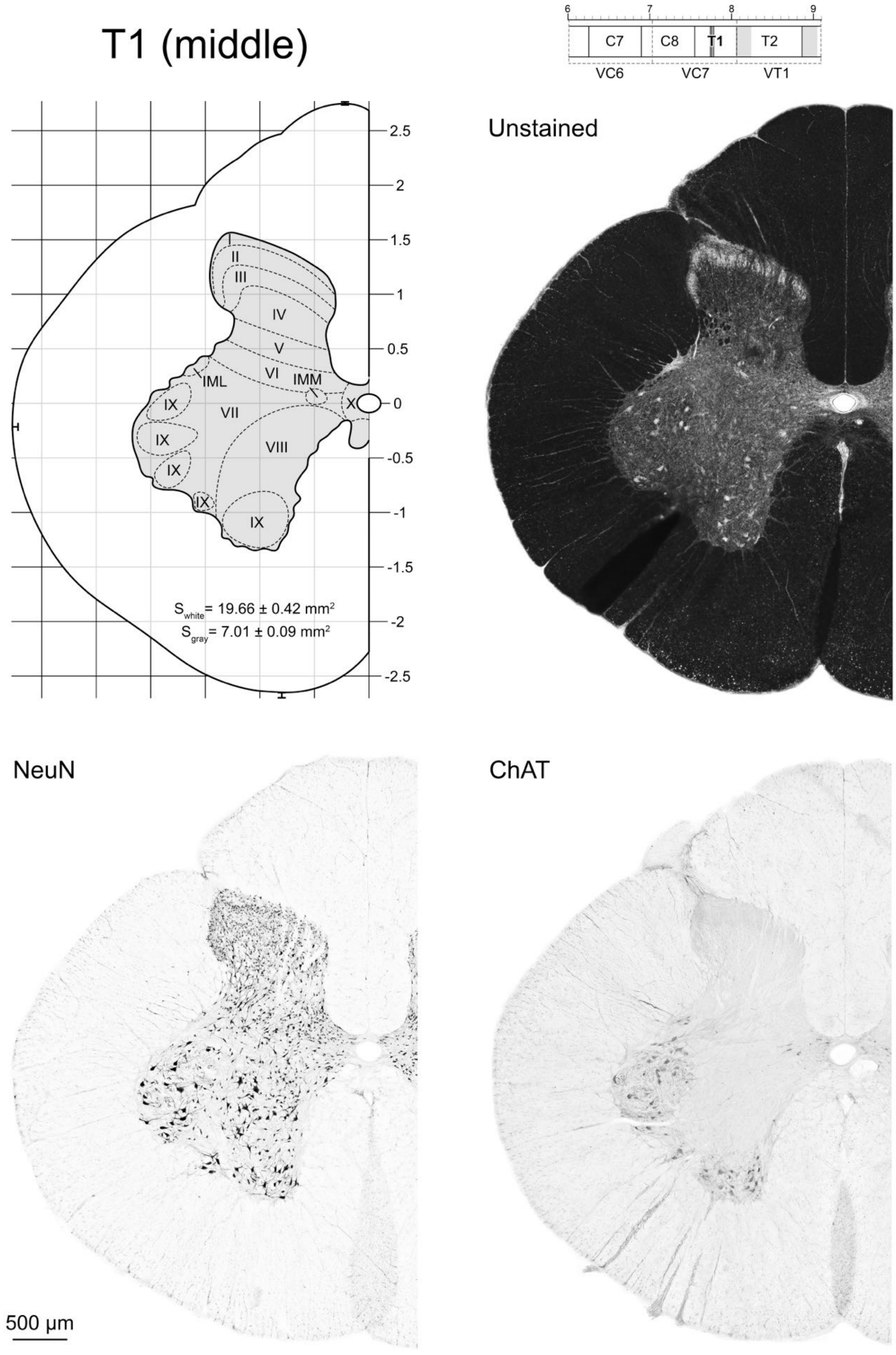

Supplementary Figure 2. Middle part of T1 segment of the cat spinal cord.

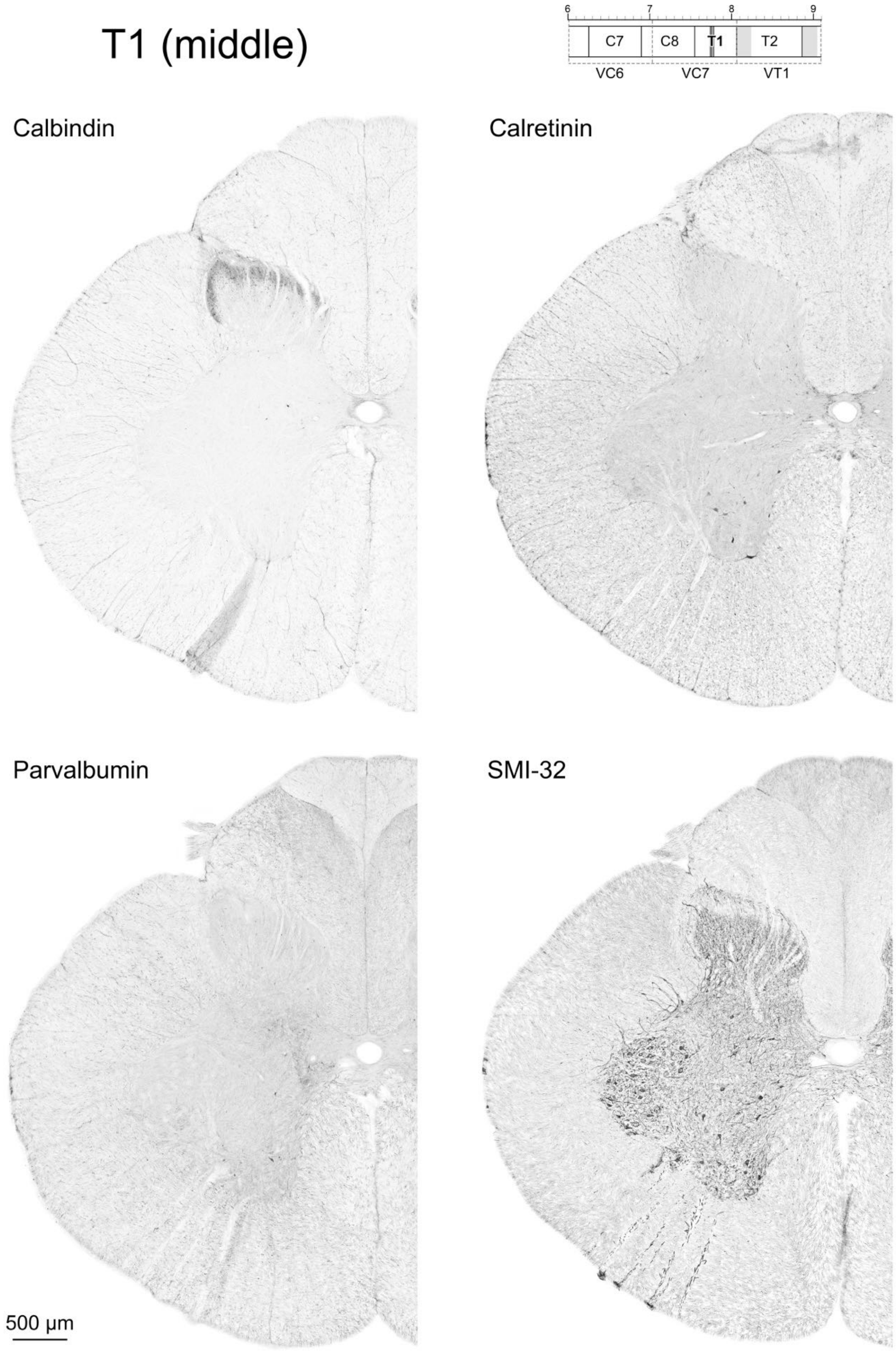

Supplementary Figure 2. Continued.

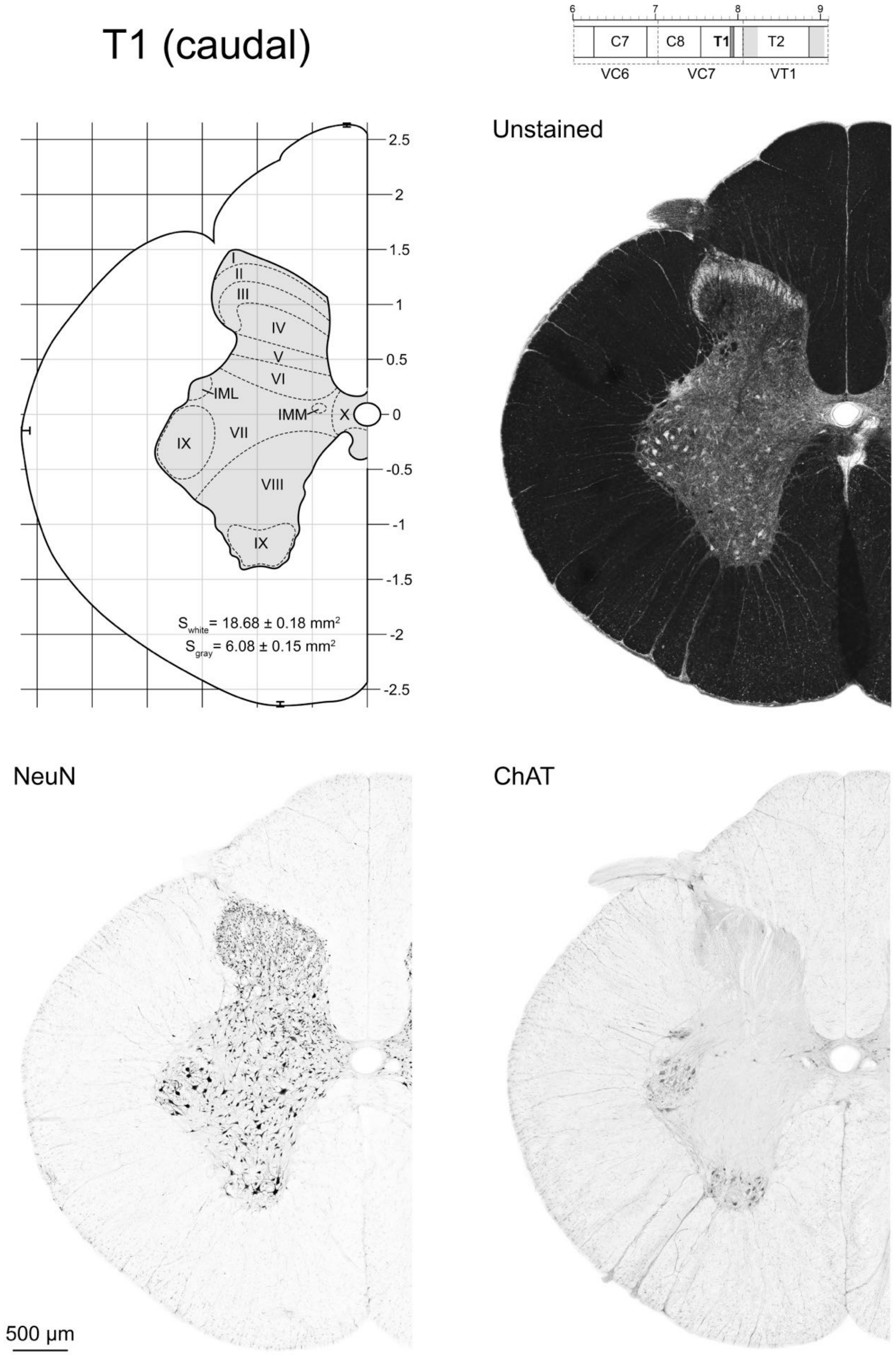

Supplementary Figure 3. Caudal part of T1 segment of the cat spinal cord.

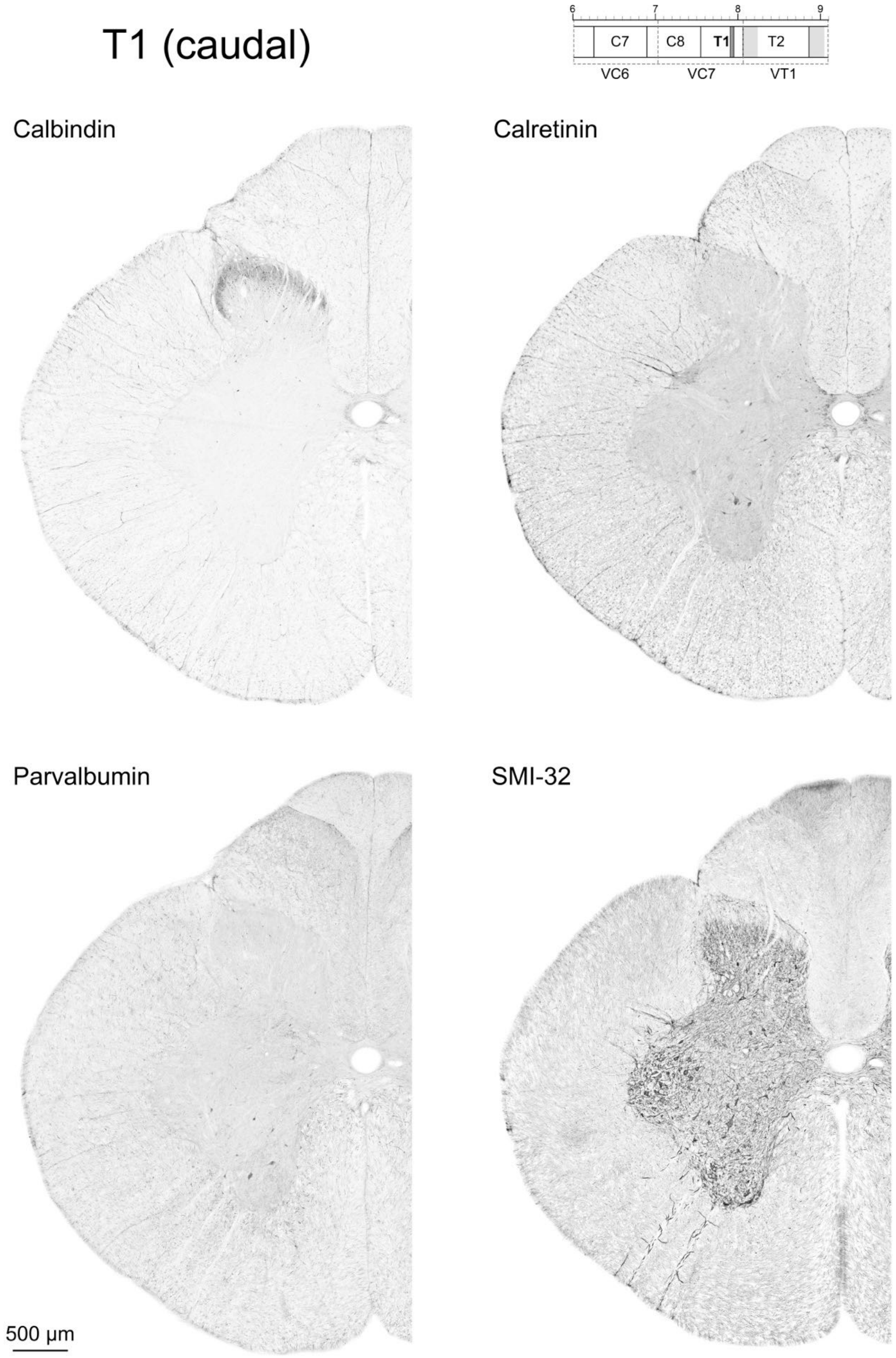

Supplementary Figure 3. Continued.

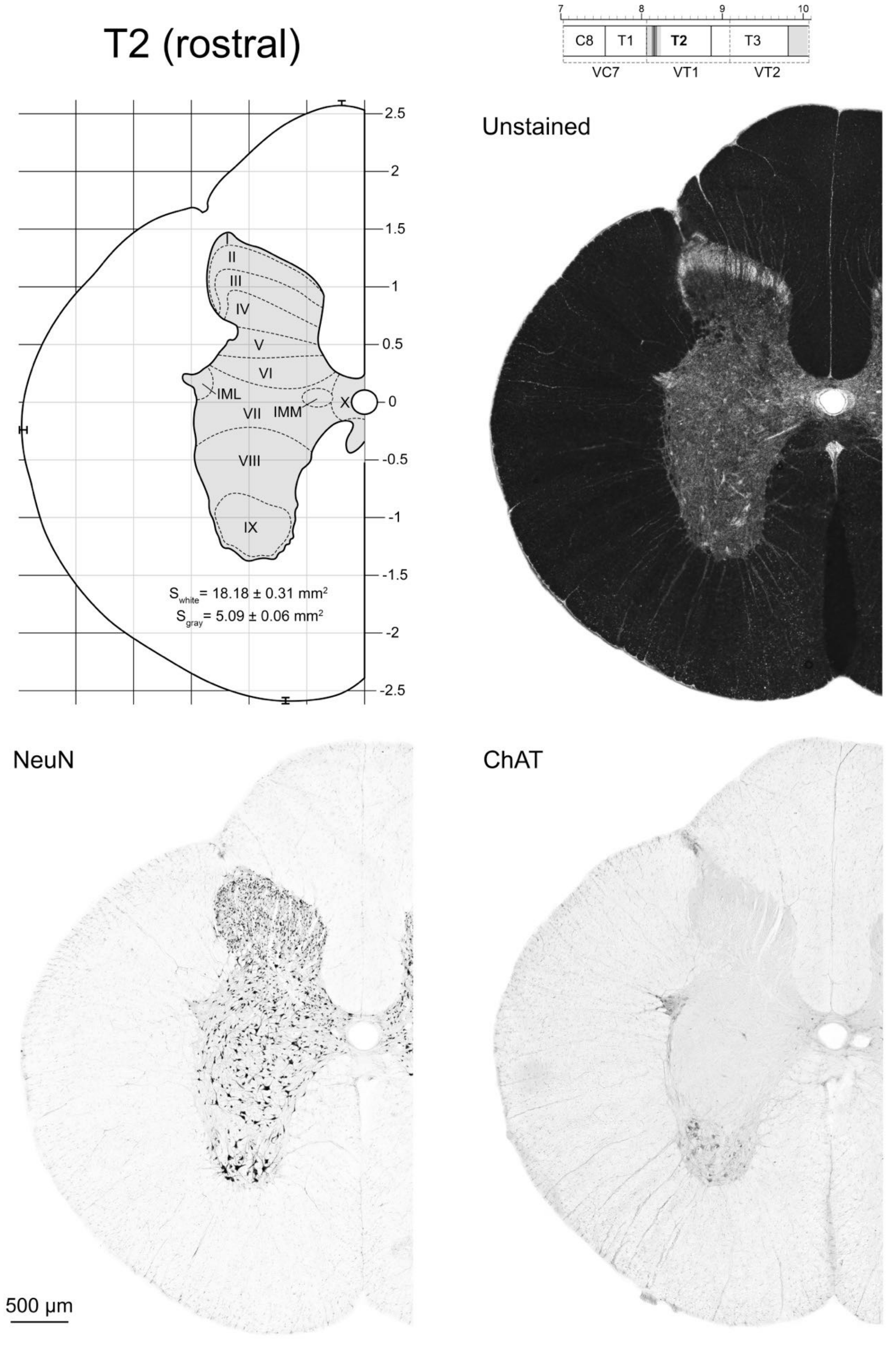

Supplementary Figure 4. Rostral part of T2 segment of the cat spinal cord.

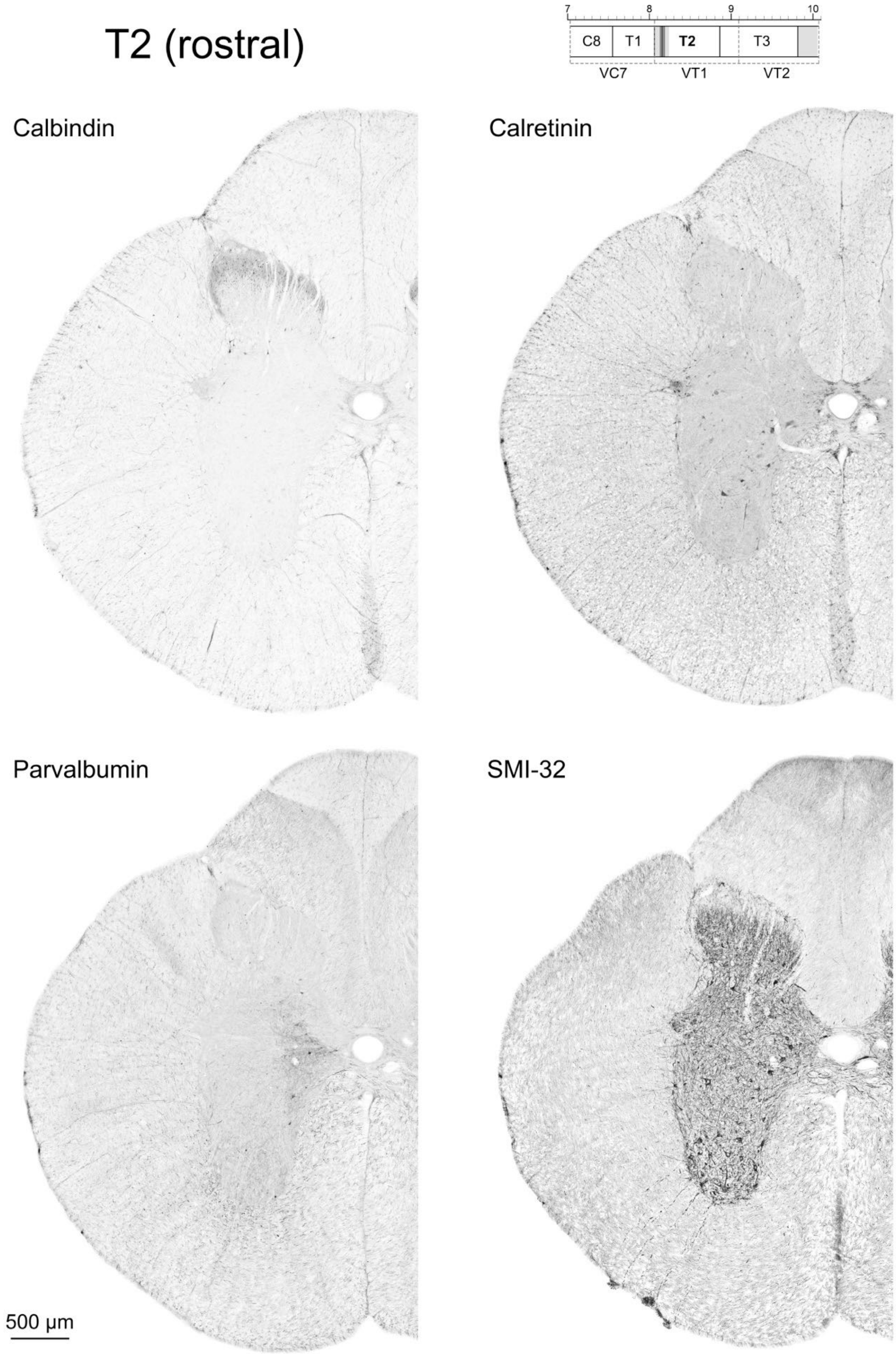

Supplementary Figure 4. Continued.

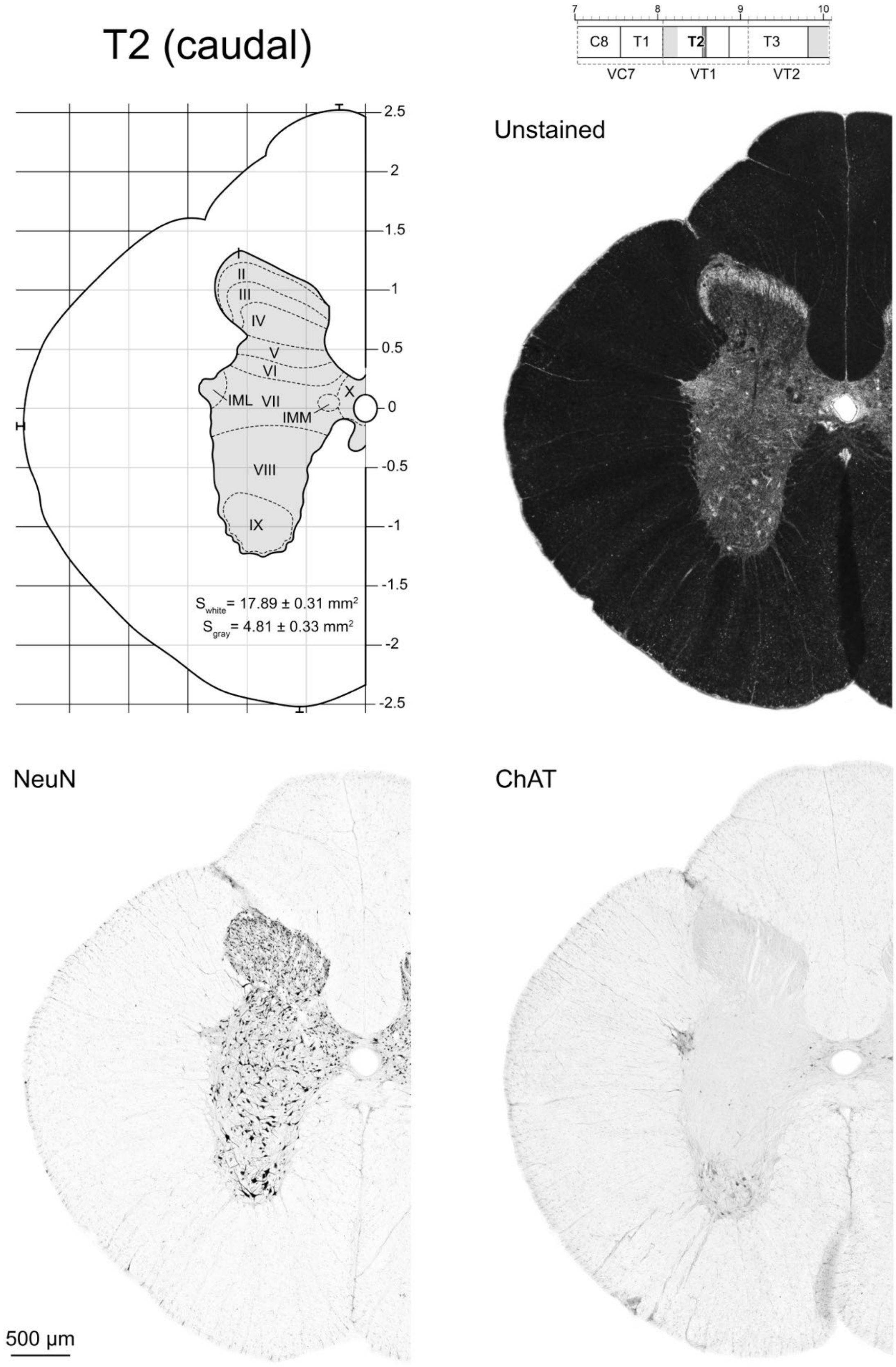

Supplementary Figure 5. Caudal part of T2 segment of the cat spinal cord.

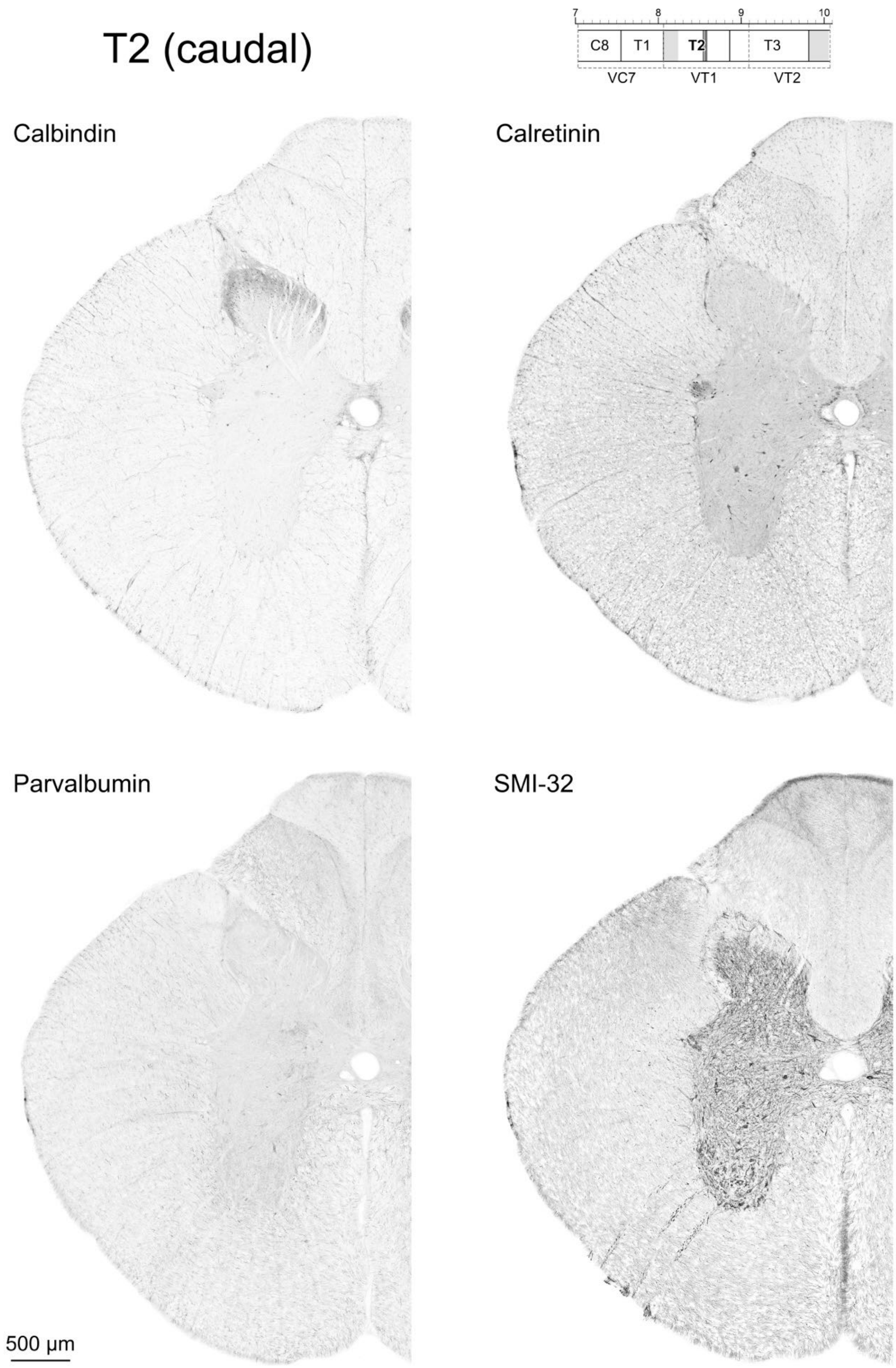

Supplementary Figure 5. Continued.

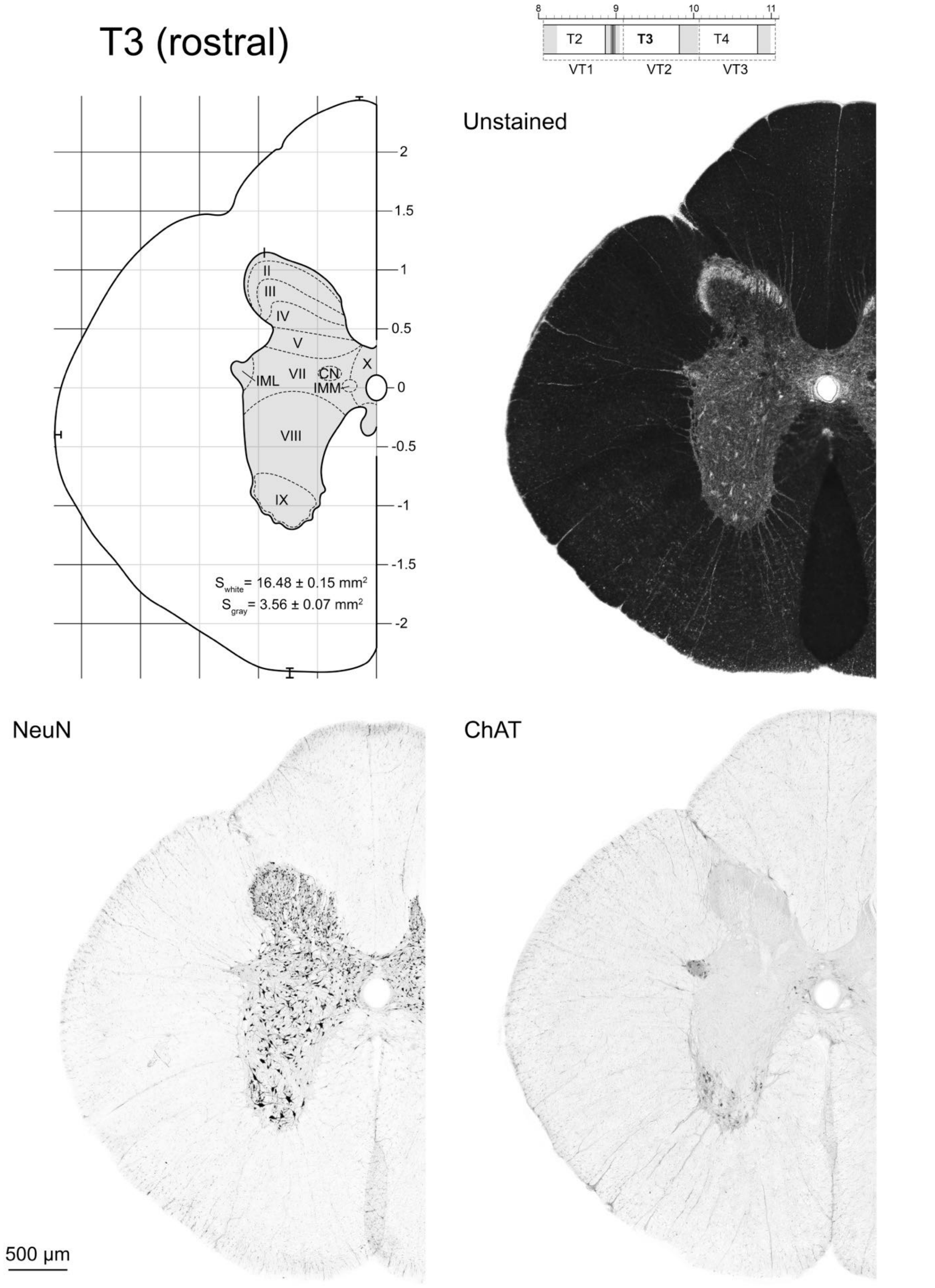

Supplementary Figure 6. Rostral part of T3 segment of the cat spinal cord.

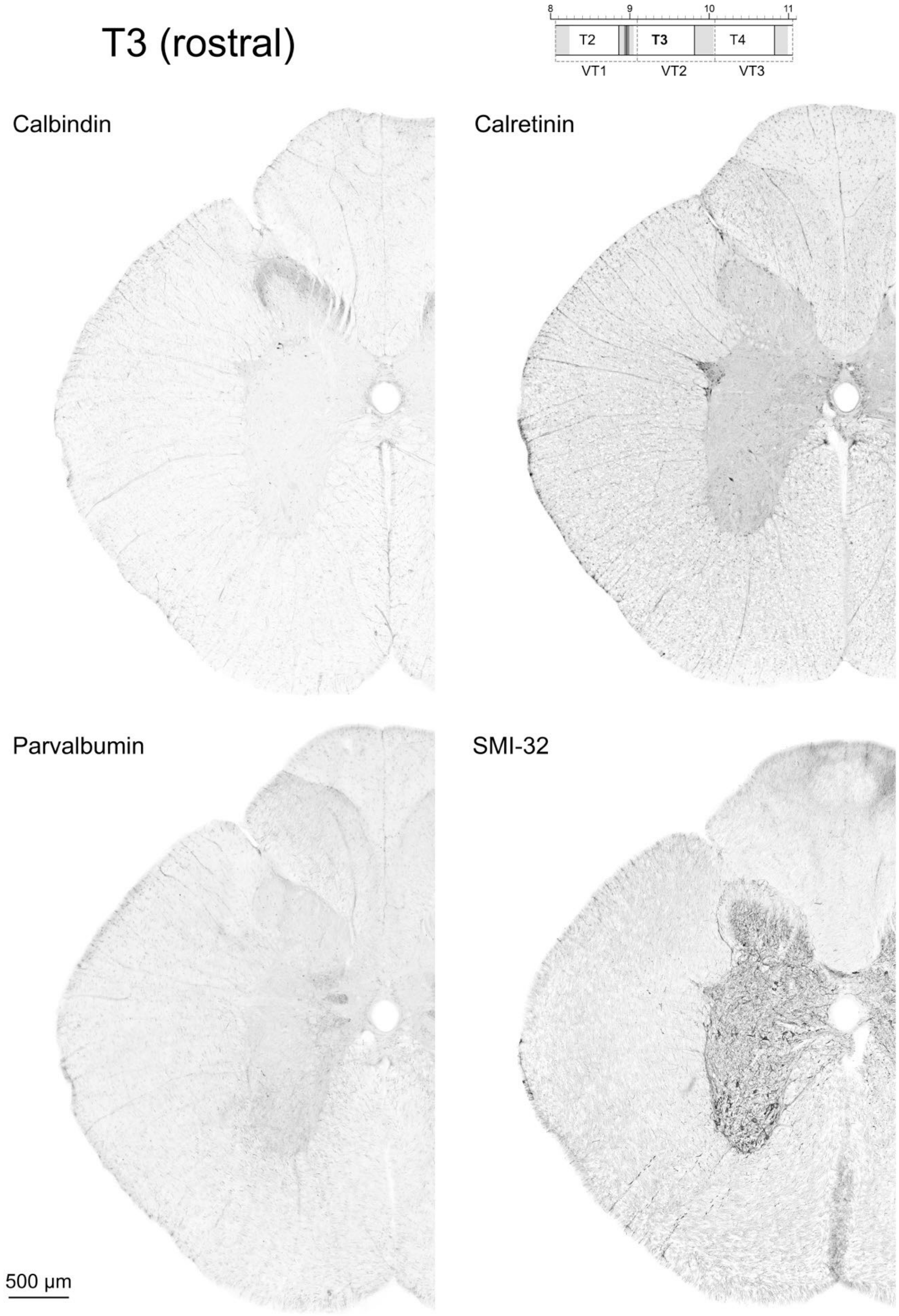

Supplementary Figure 6. Continued.

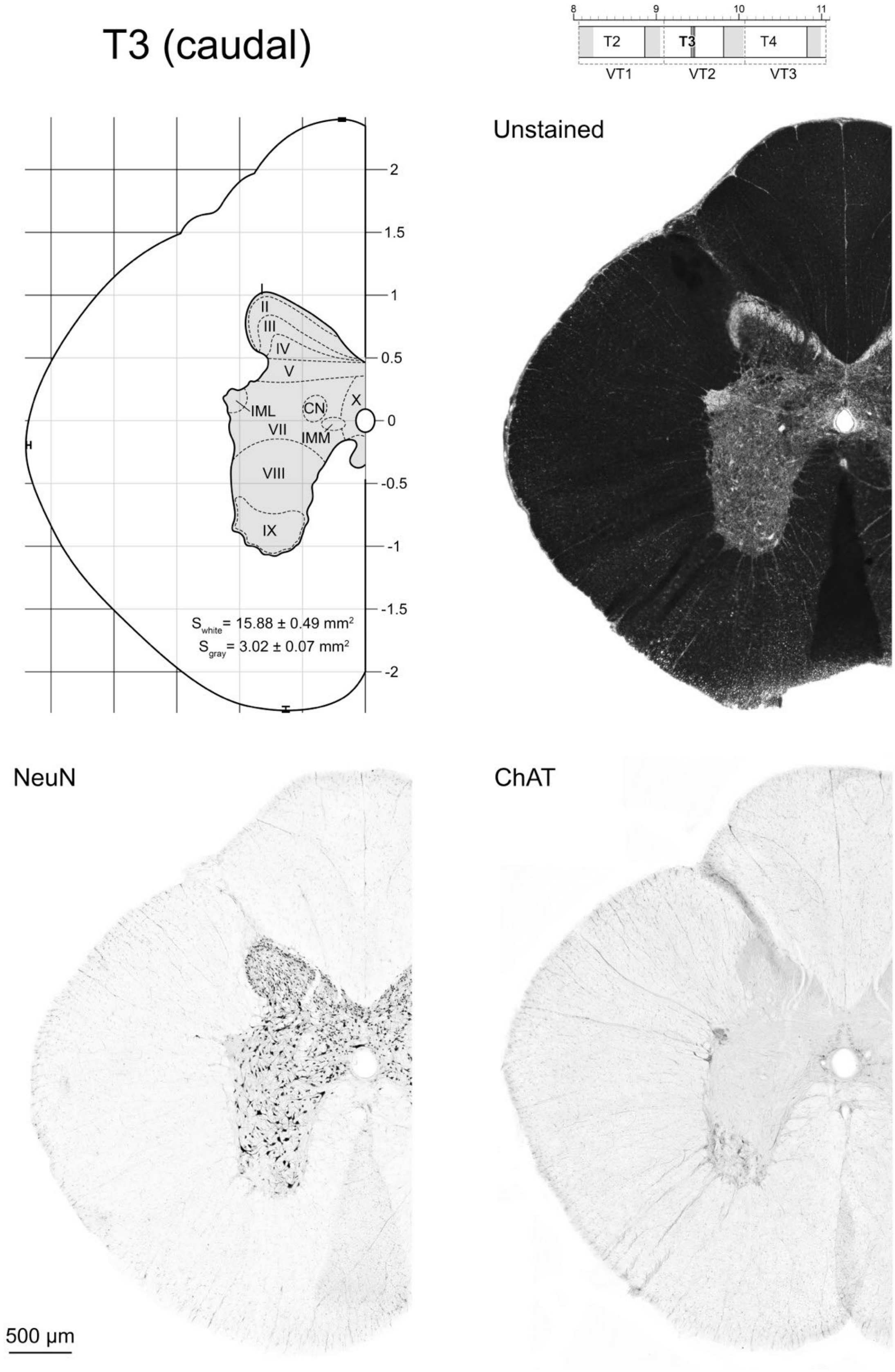

Supplementary Figure 7. Caudal part of T3 segment of the cat spinal cord.

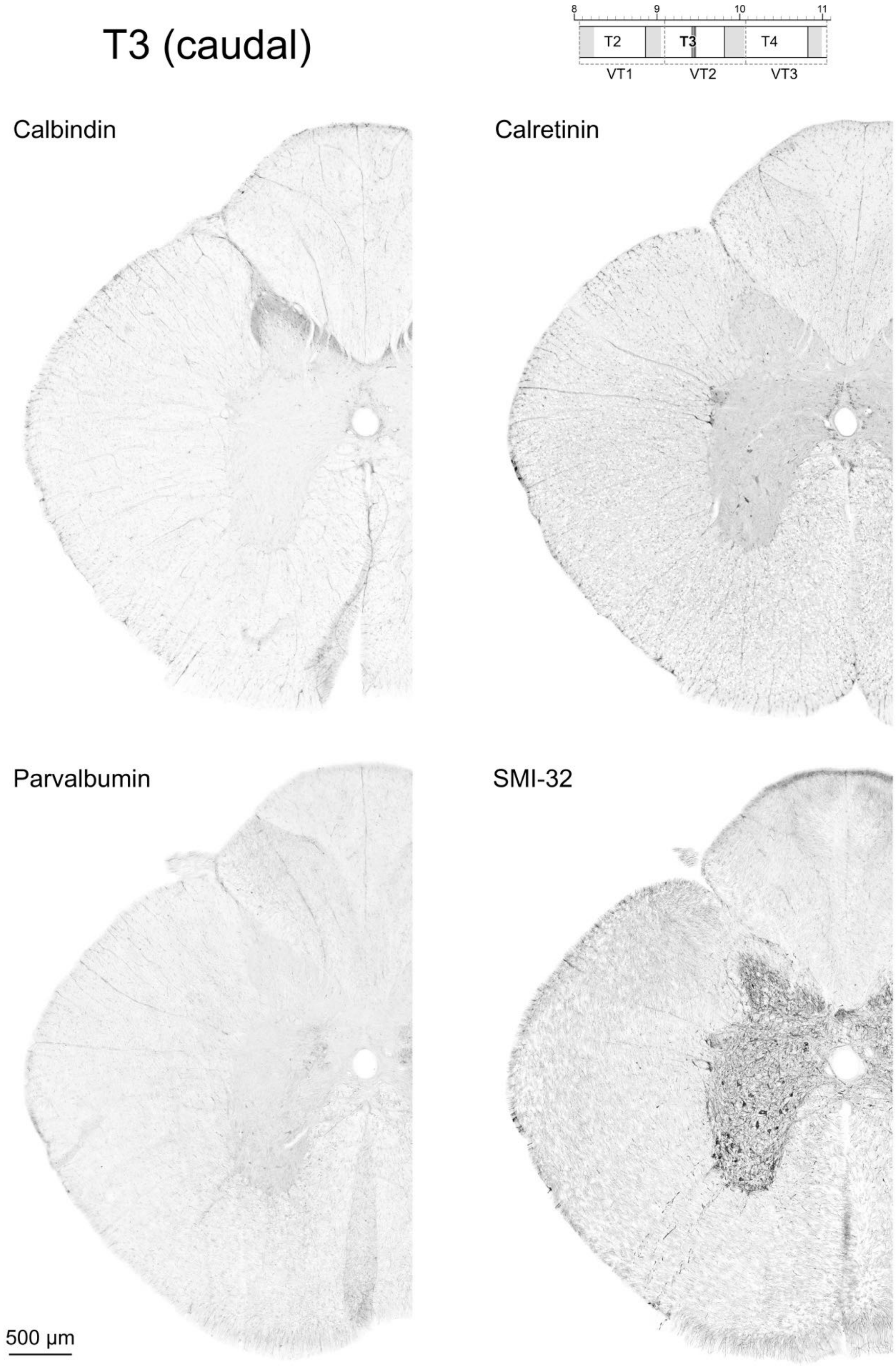

Supplementary Figure 7. Continued.

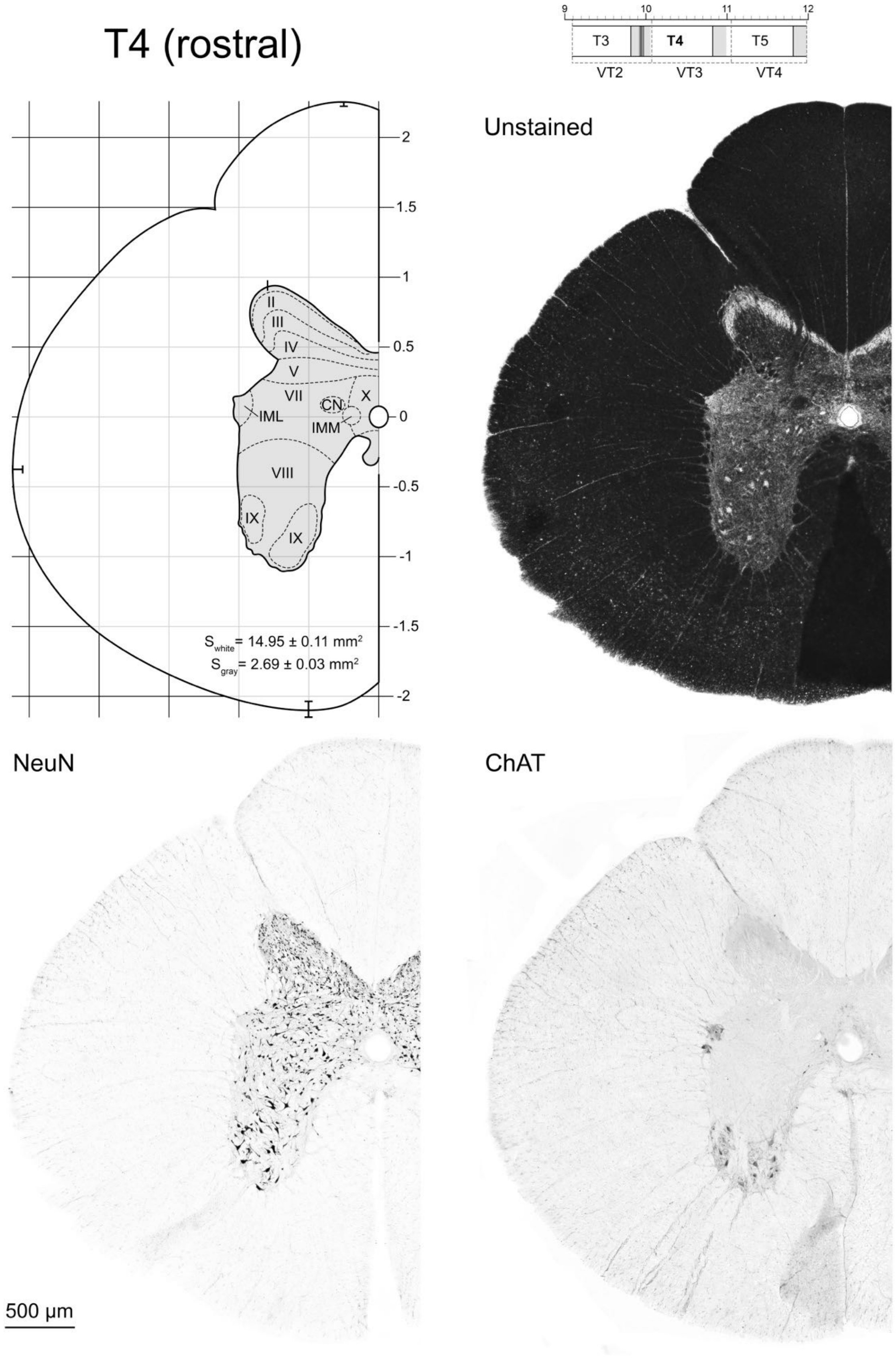

Supplementary Figure 8. Rostral part of T4 segment of the cat spinal cord.

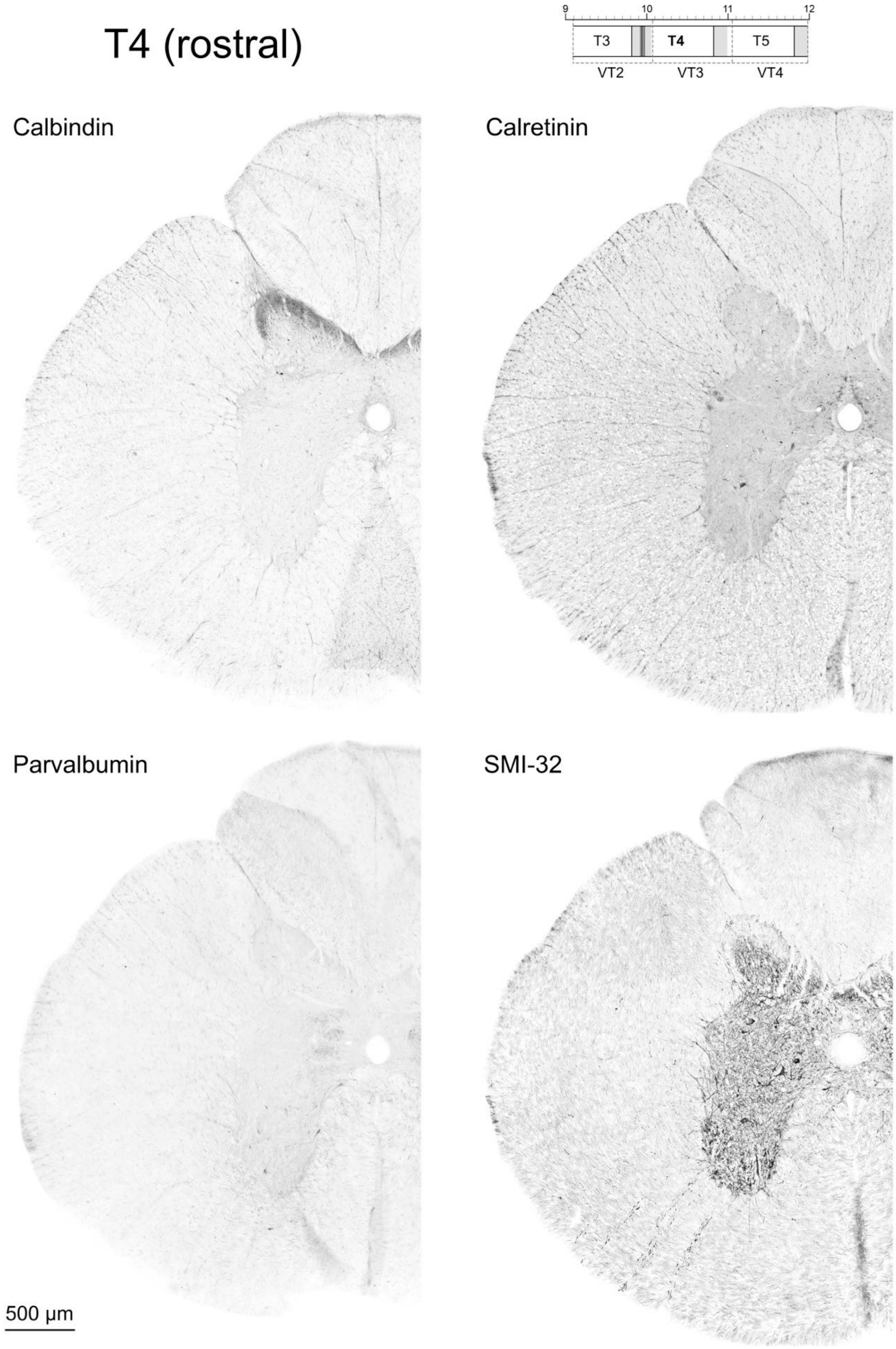

Supplementary Figure 8. Continued.

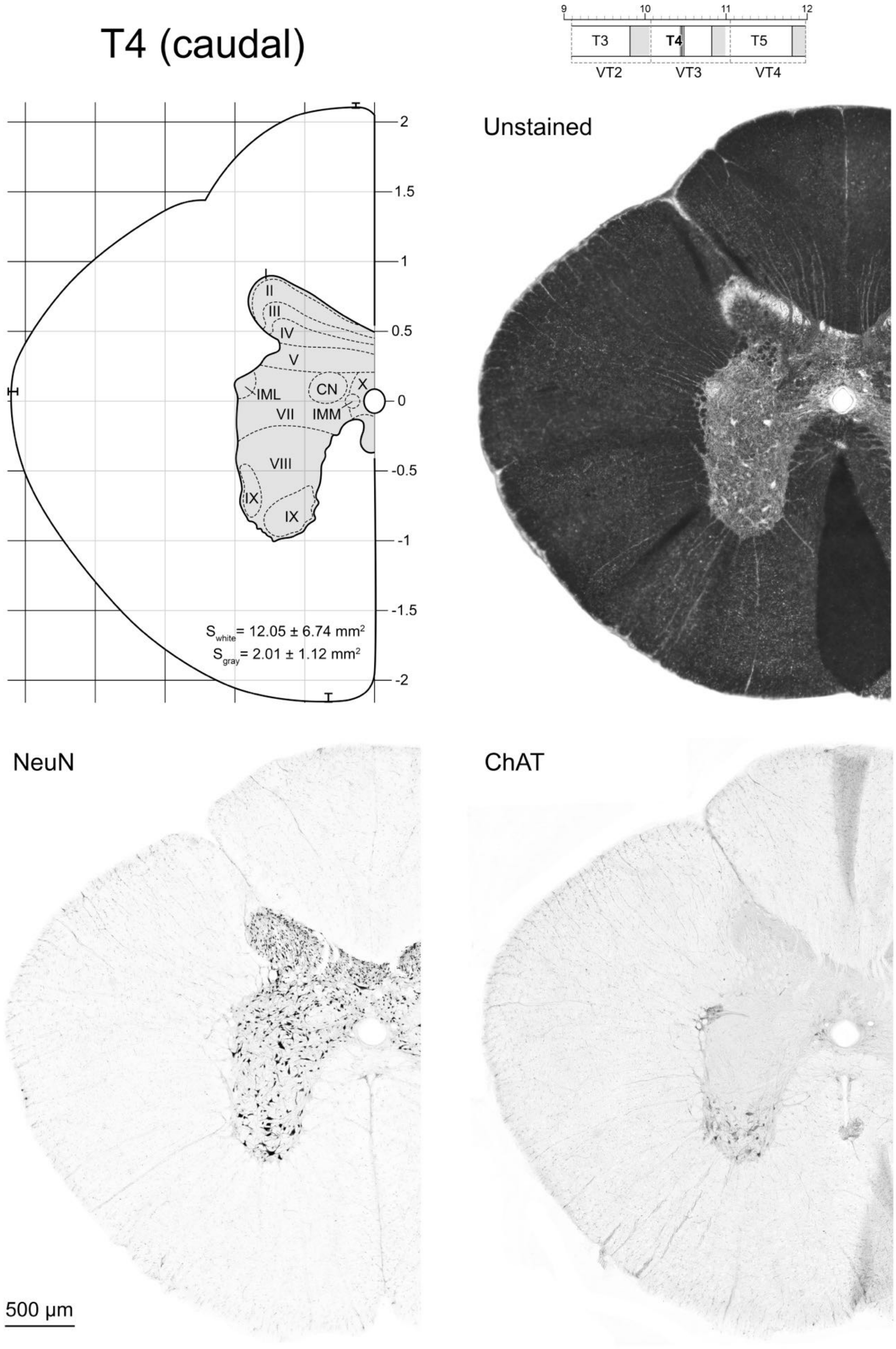

Supplementary Figure 9. Caudal part of T4 segment of the cat spinal cord.

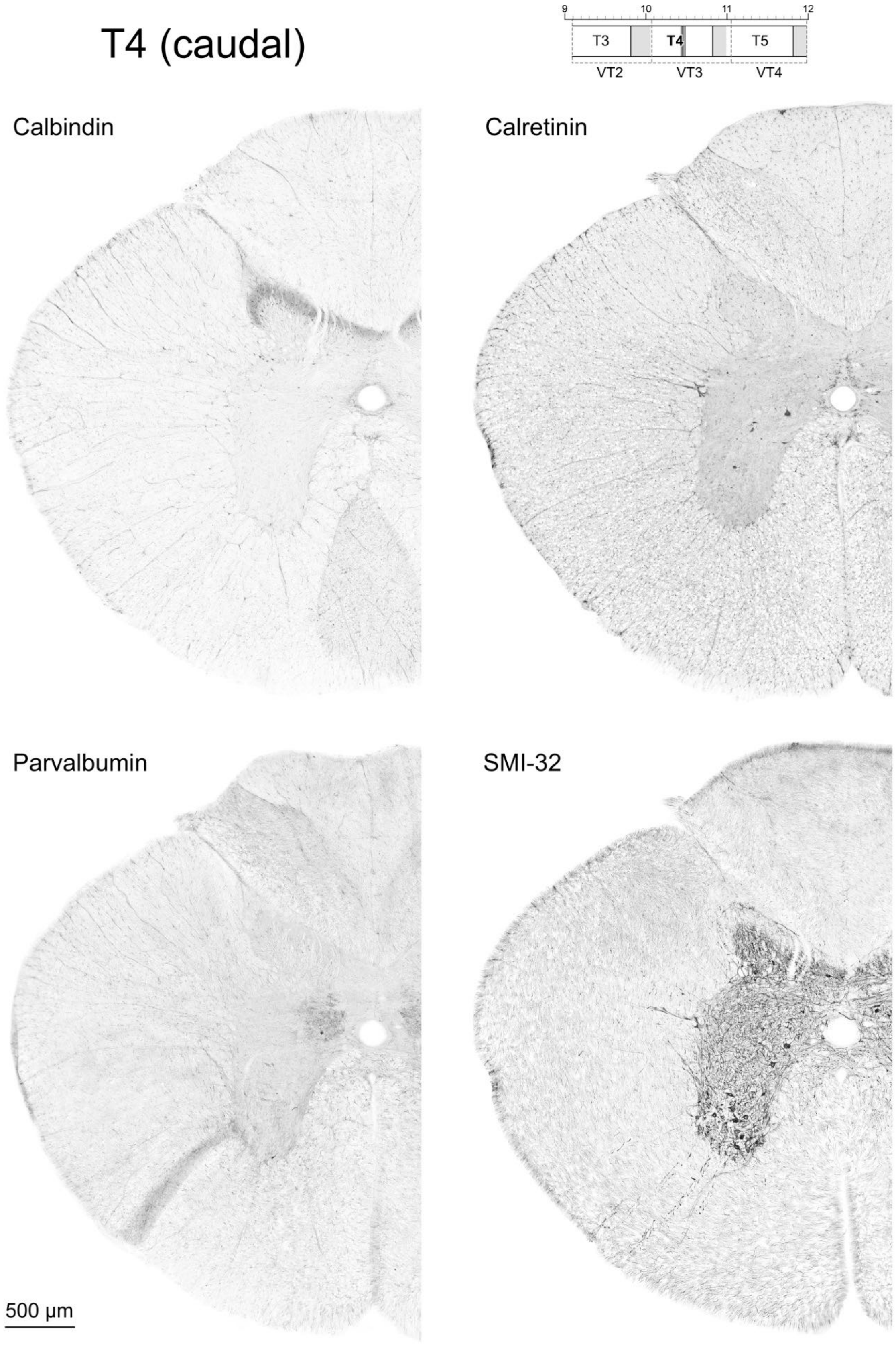

Supplementary Figure 9. Continued.

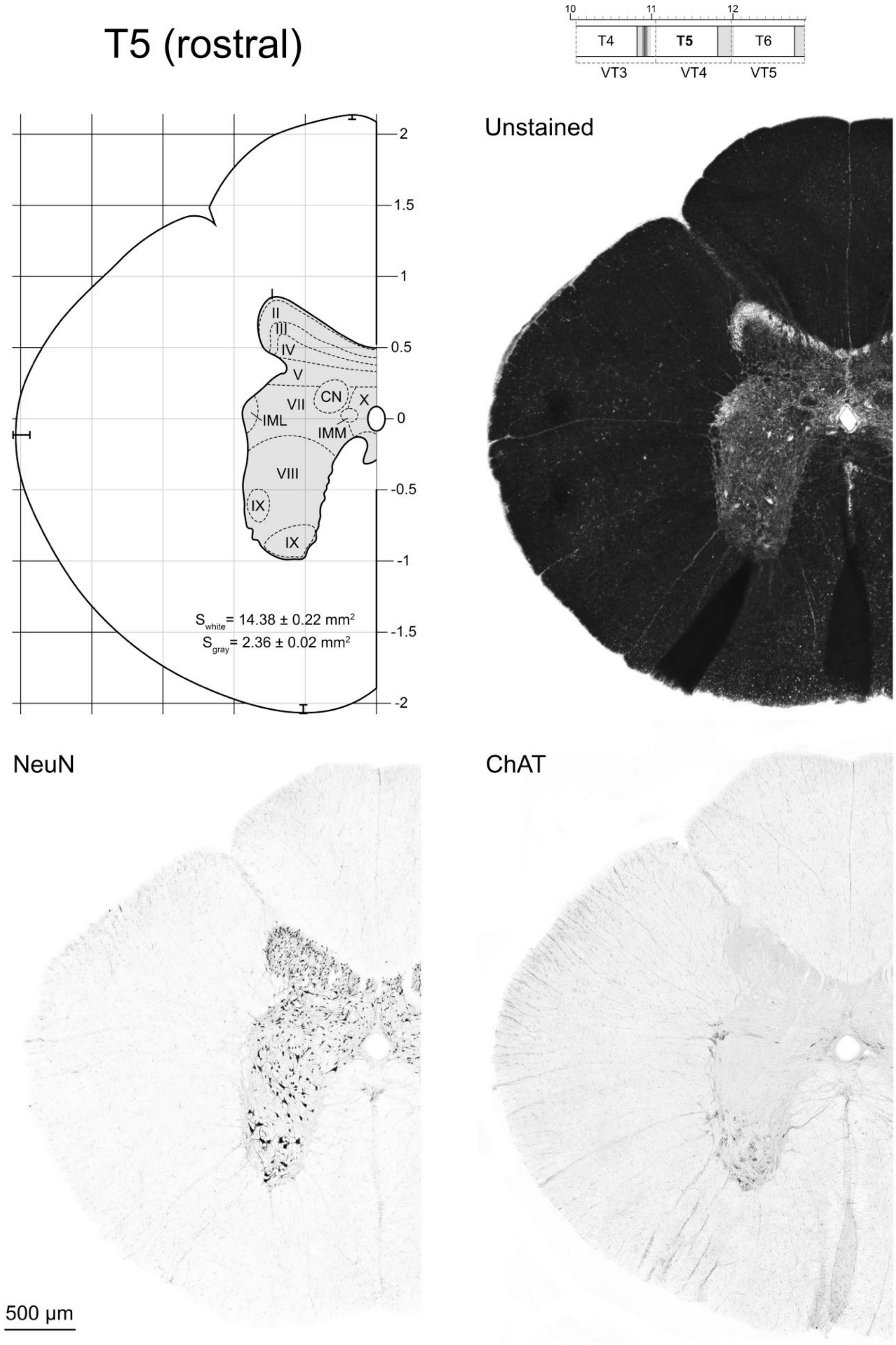

Supplementary Figure 10. Rostral part of T5 segment of the cat spinal cord.

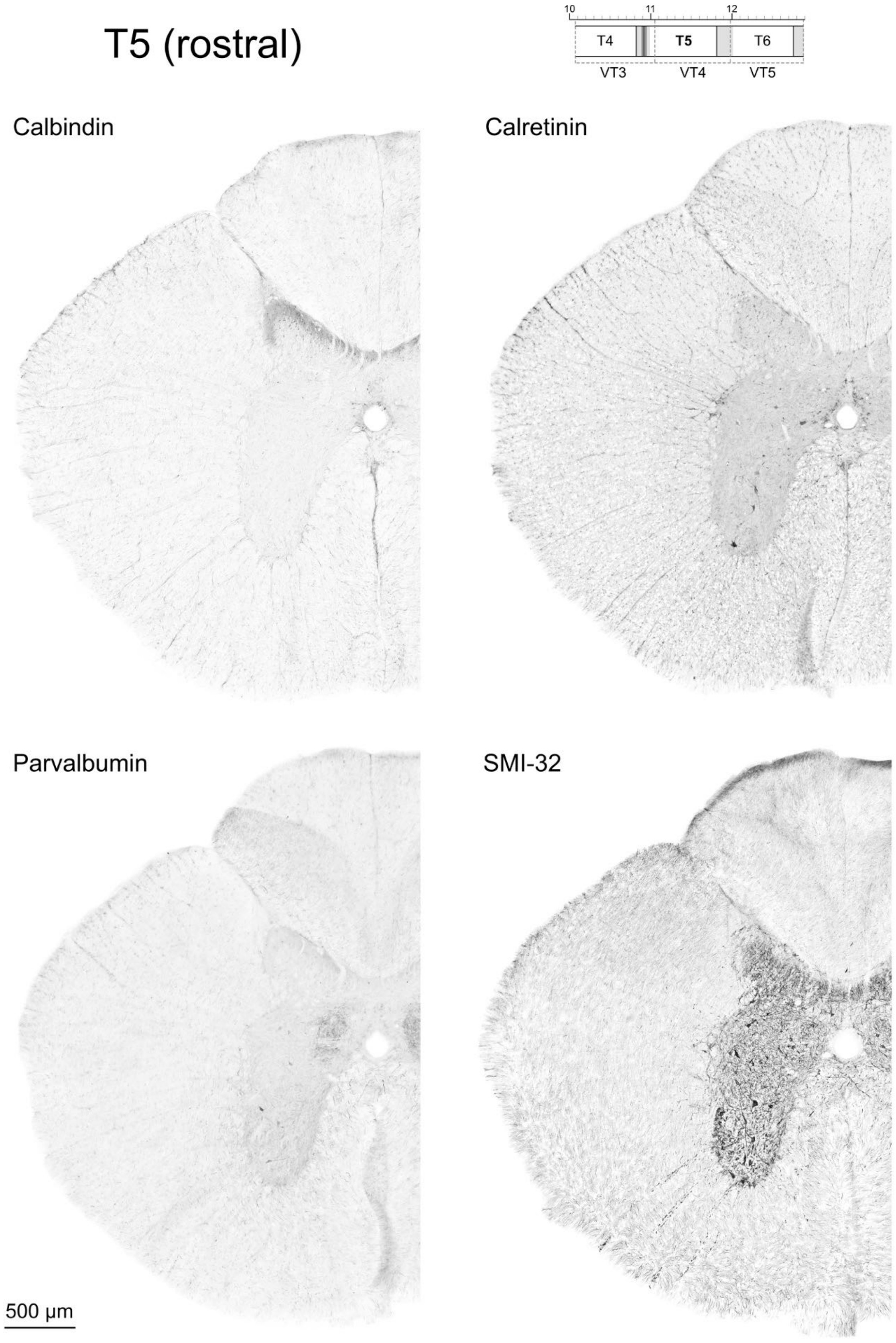

Supplementary Figure 10. Continued.

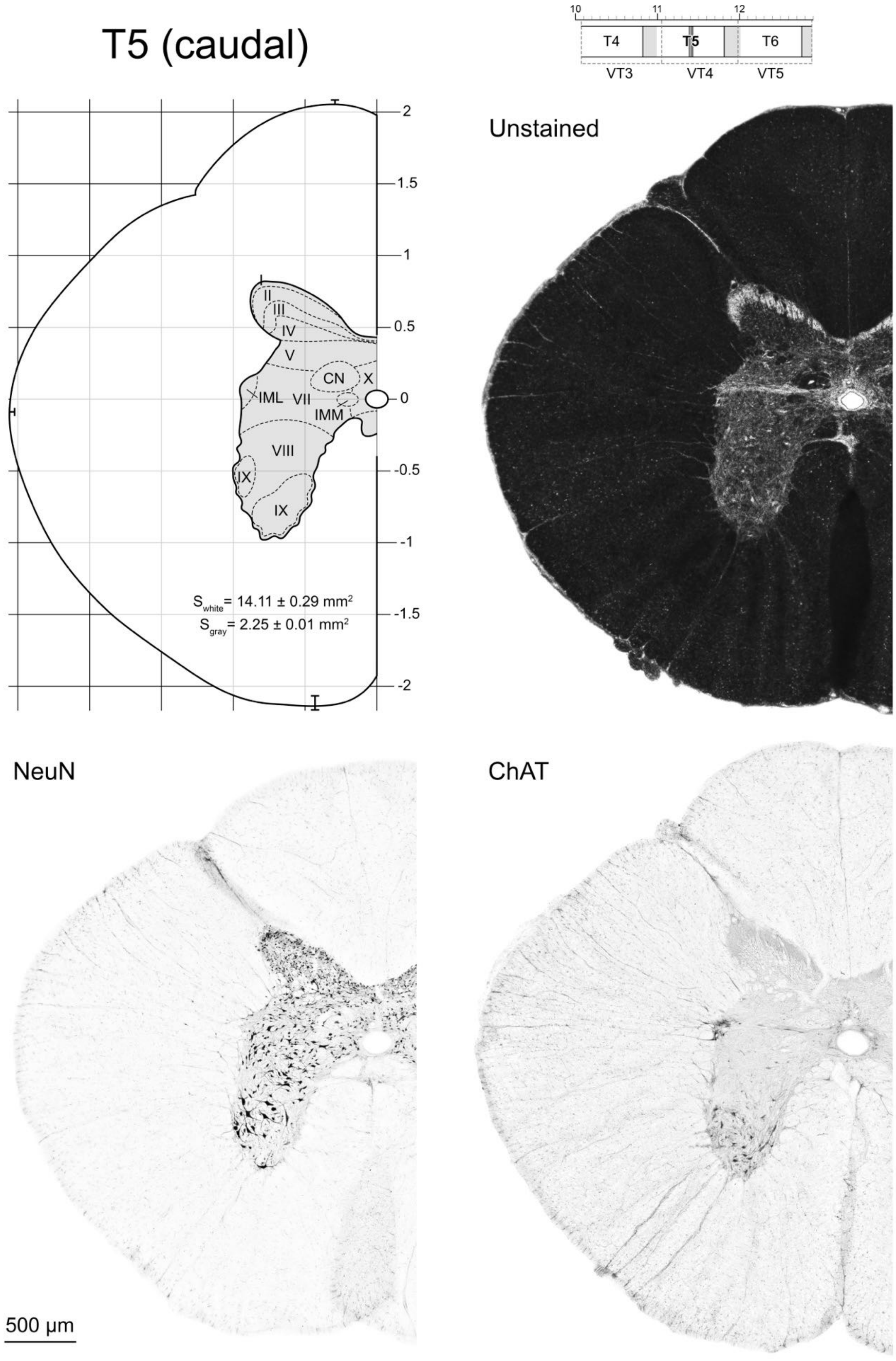

Supplementary Figure 11. Caudal part of T5 segment of the cat spinal cord.

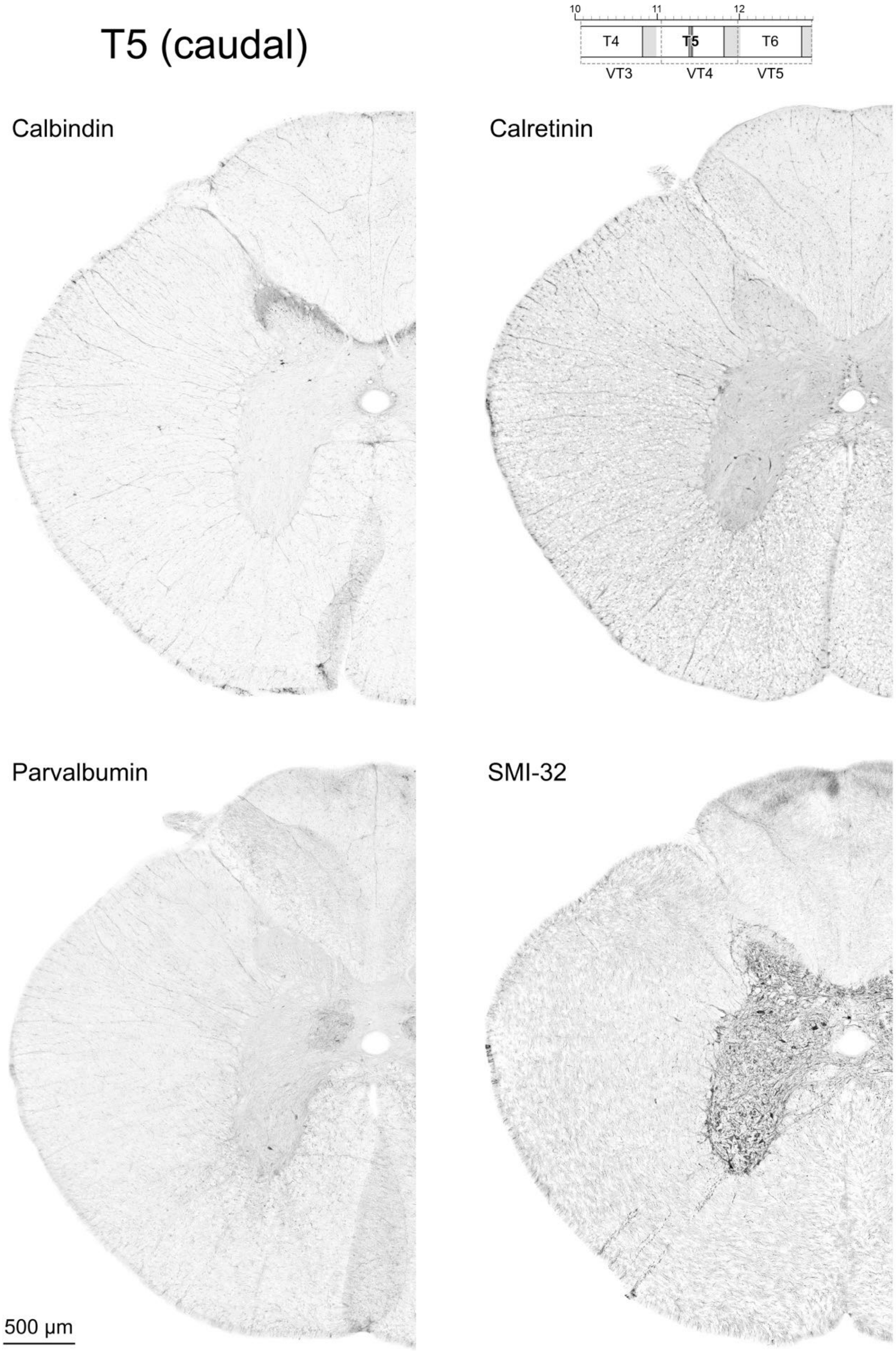

Supplementary Figure 11. Continued.

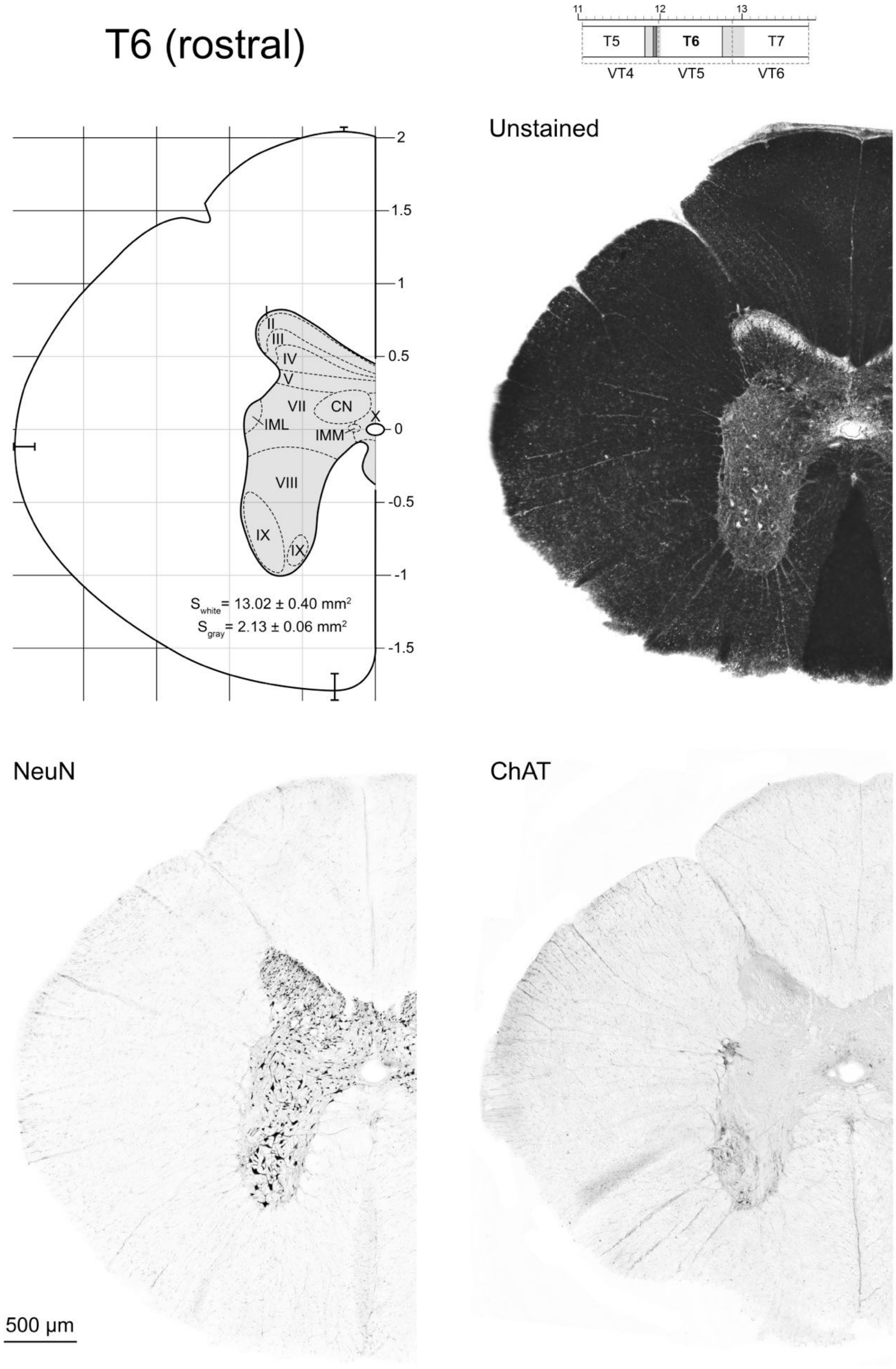

Supplementary Figure 12. Rostral part of T6 segment of the cat spinal cord.

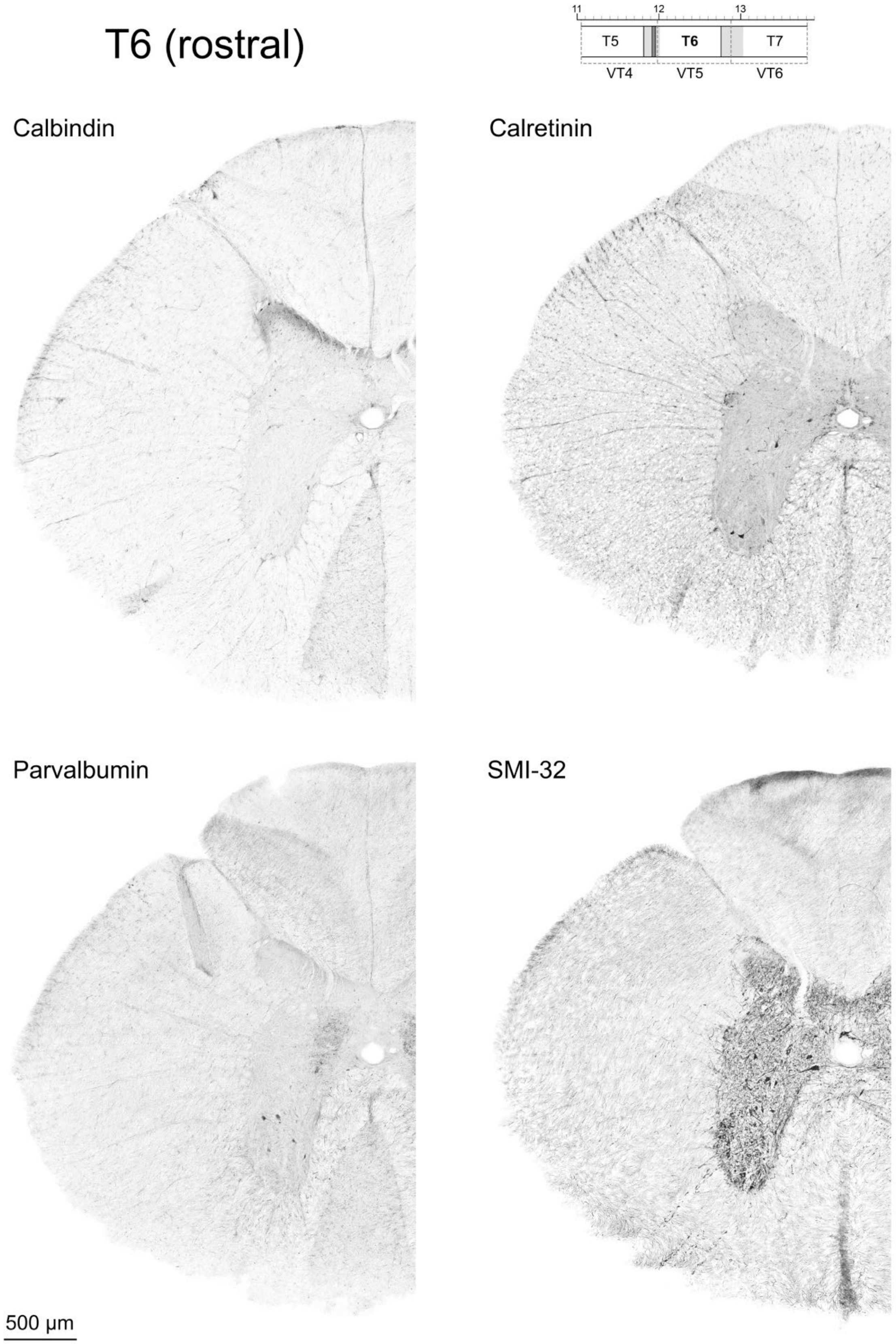

Supplementary Figure 12. Continued.

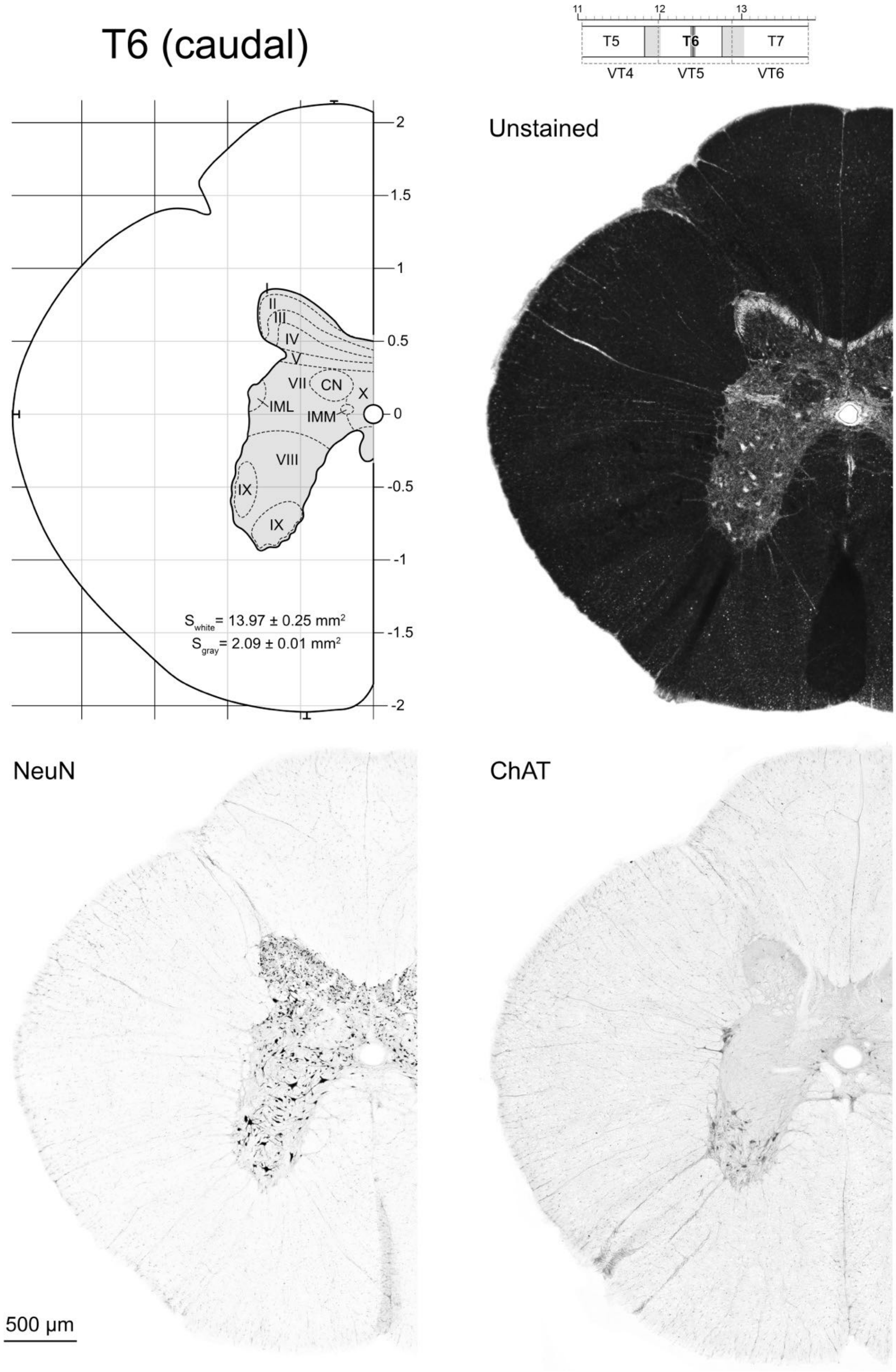

Supplementary Figure 13. Caudal part of T6 segment of the cat spinal cord.

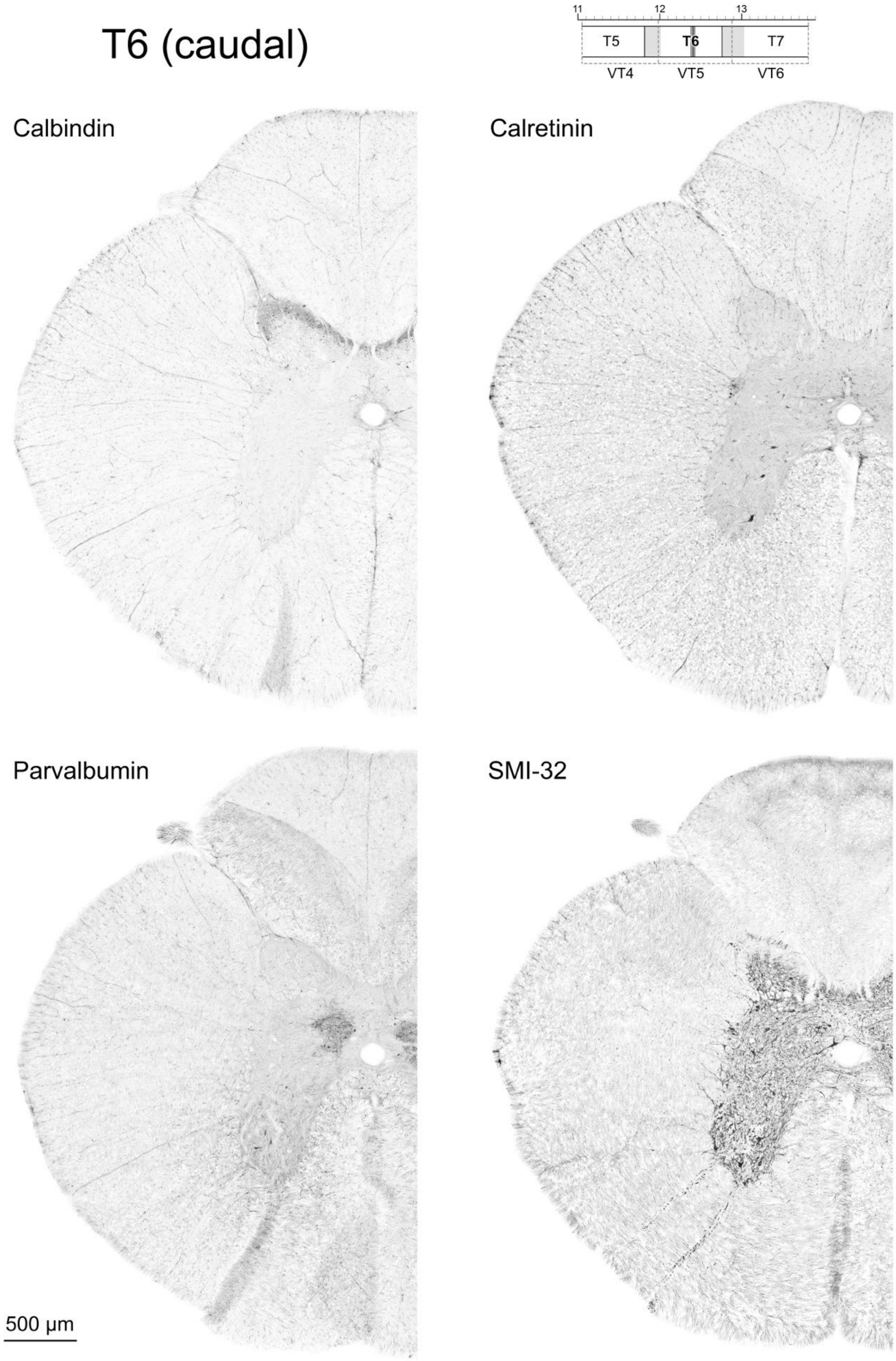

Supplementary Figure 13. Continued.

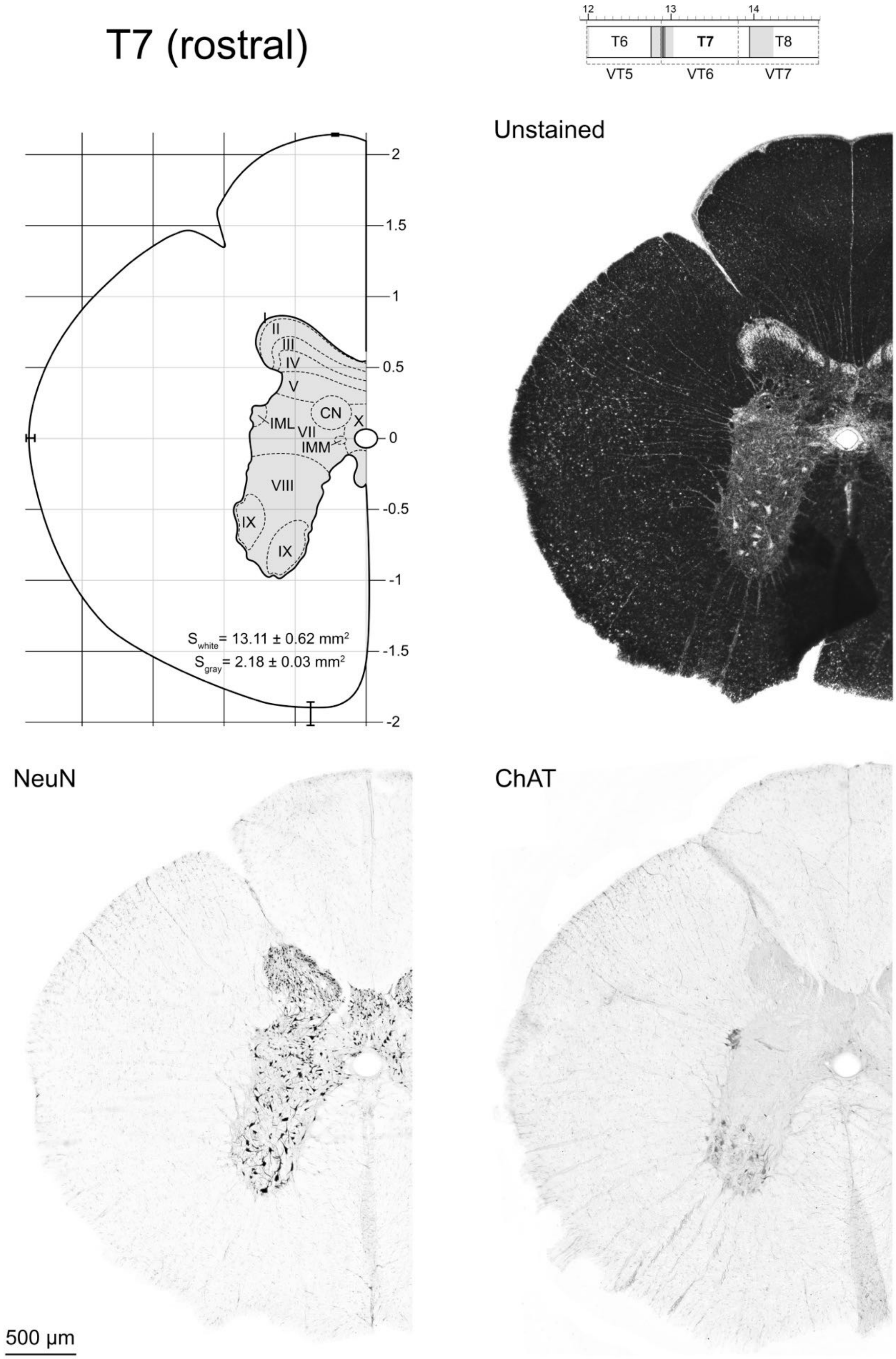

Supplementary Figure 14. Rostral part of T7 segment of the cat spinal cord.

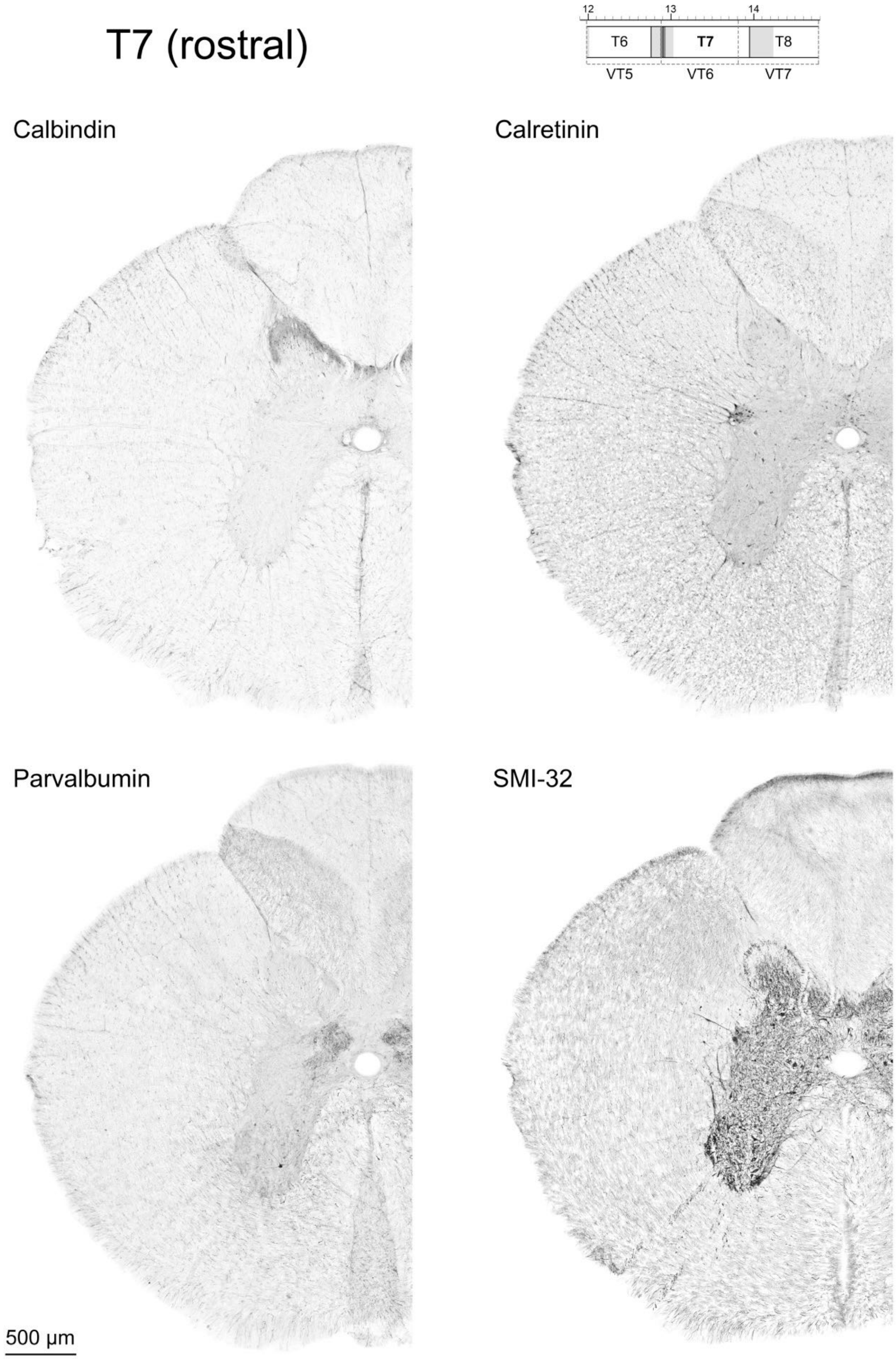

Supplementary Figure 14. Continued.

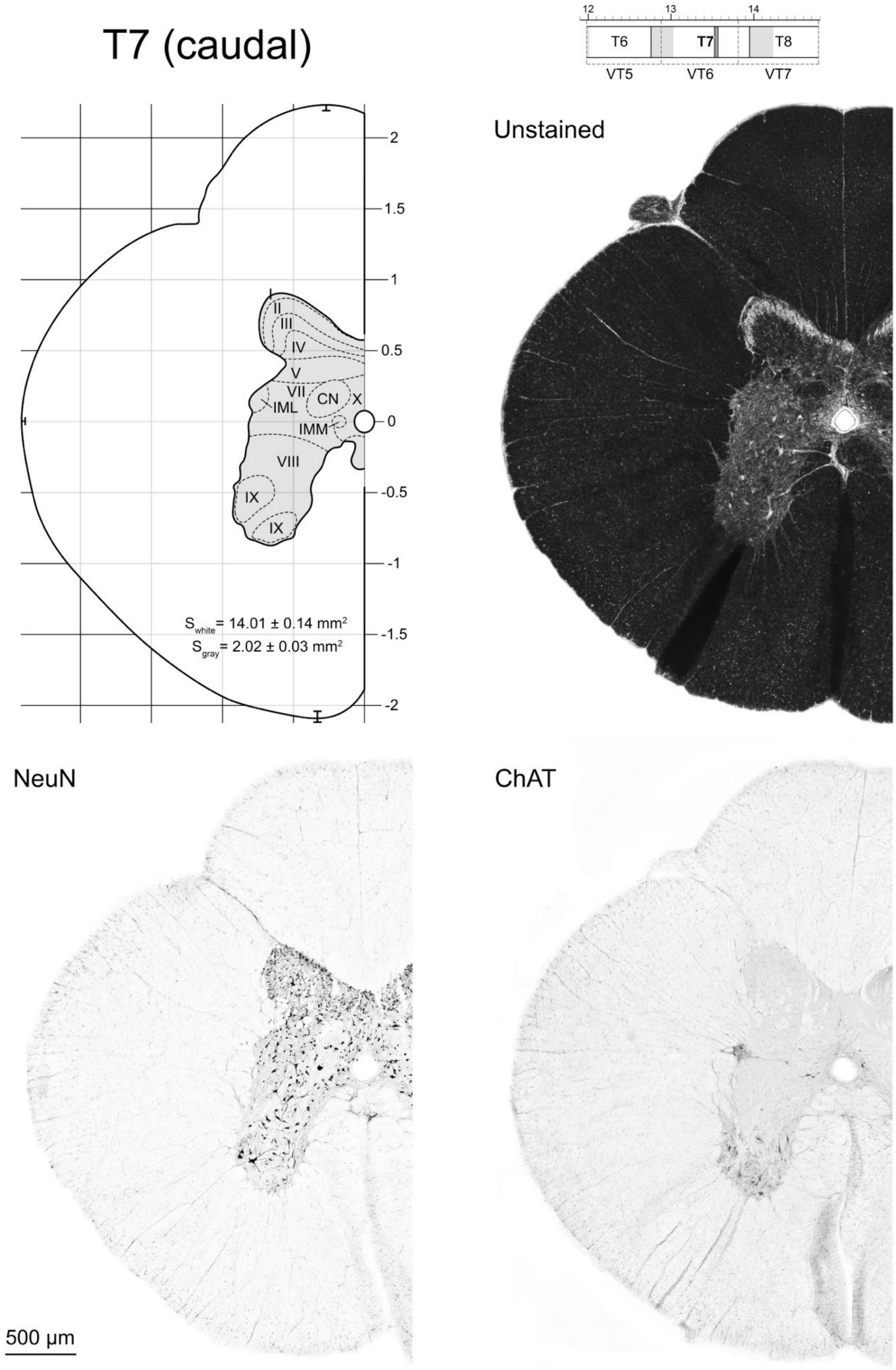

Supplementary Figure 15. Caudal part of T7 segment of the cat spinal cord.

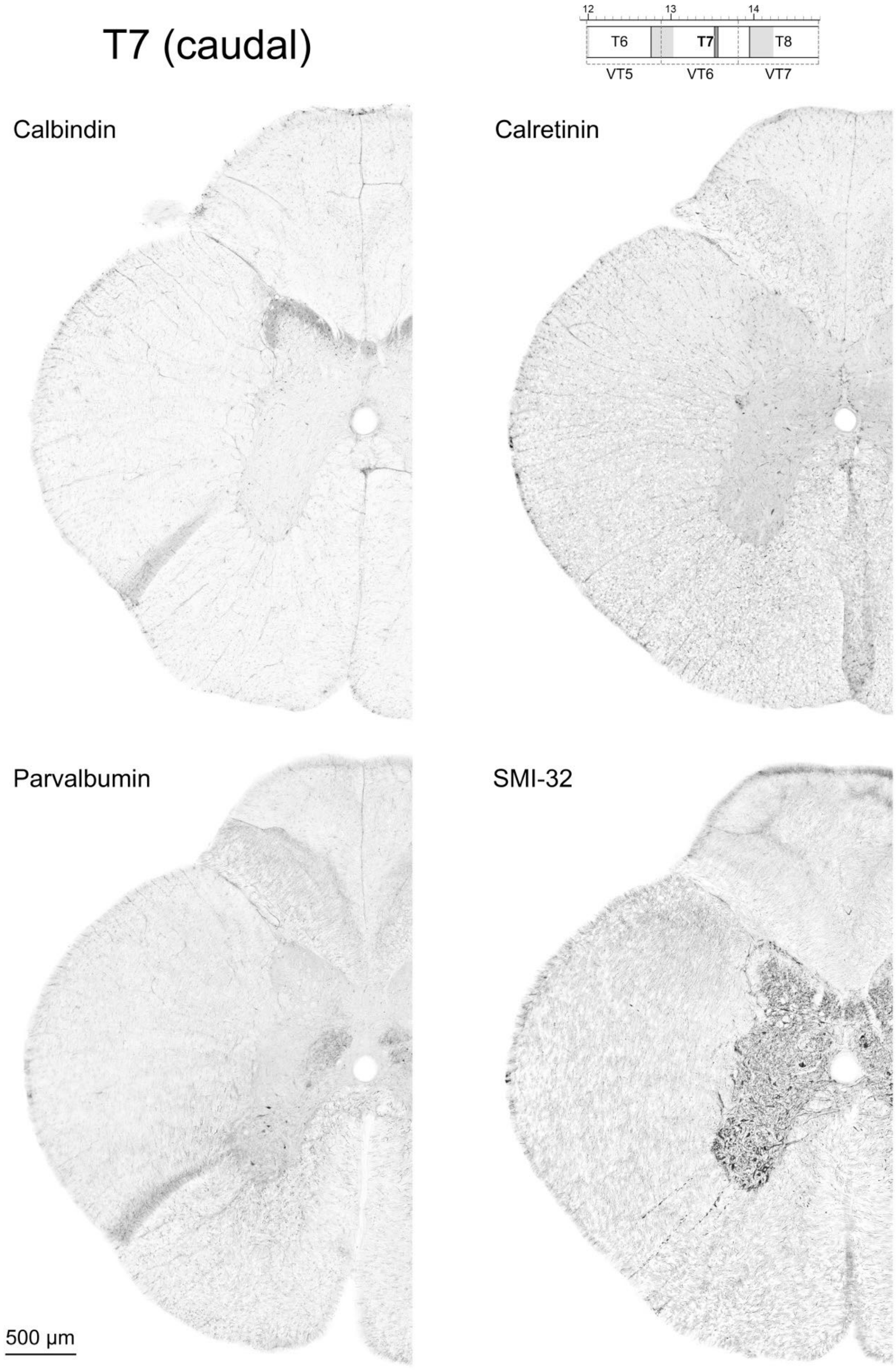

Supplementary Figure 15. Continued.

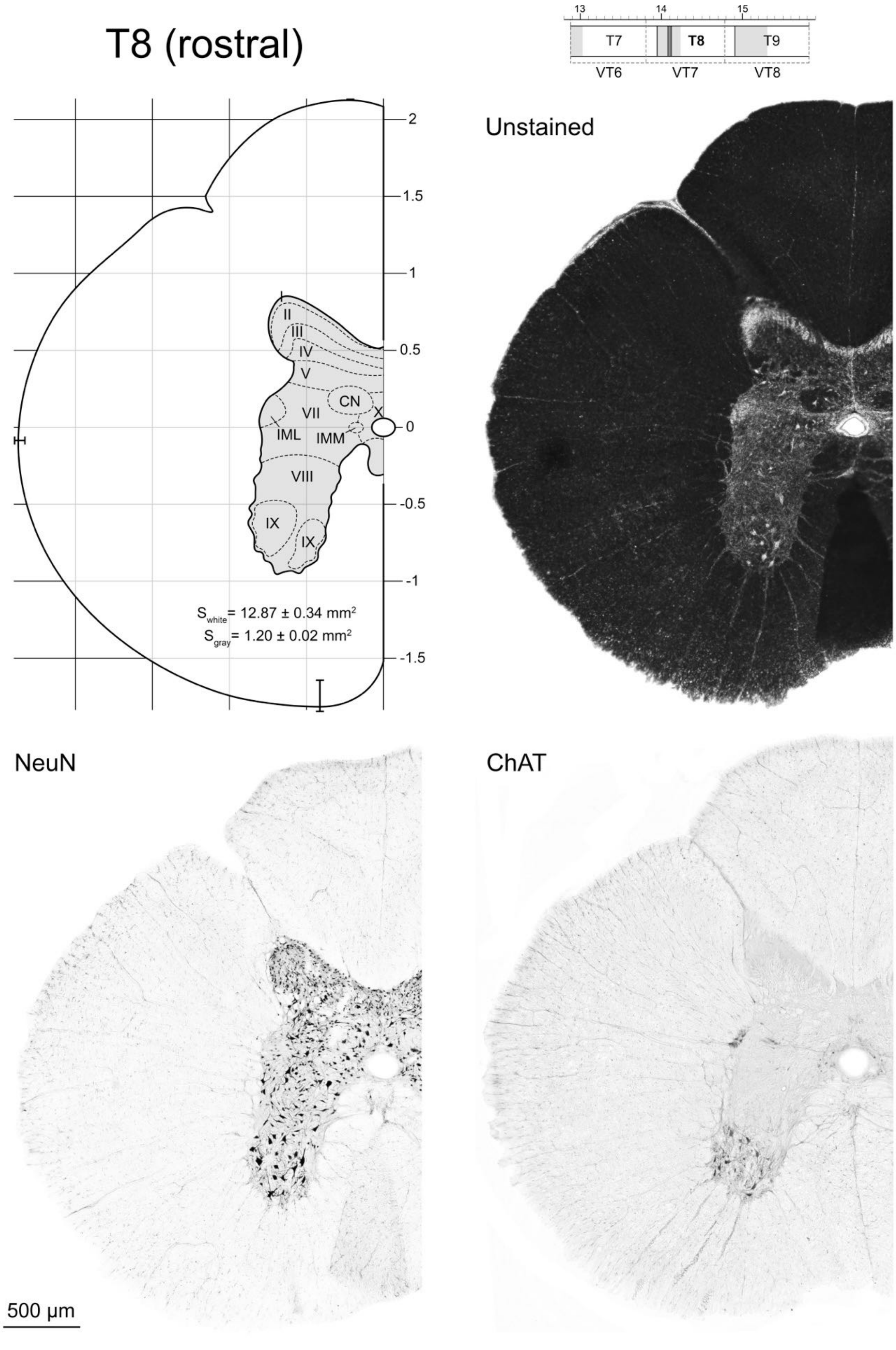

Supplementary Figure 16. Rostral part of T8 segment of the cat spinal cord.

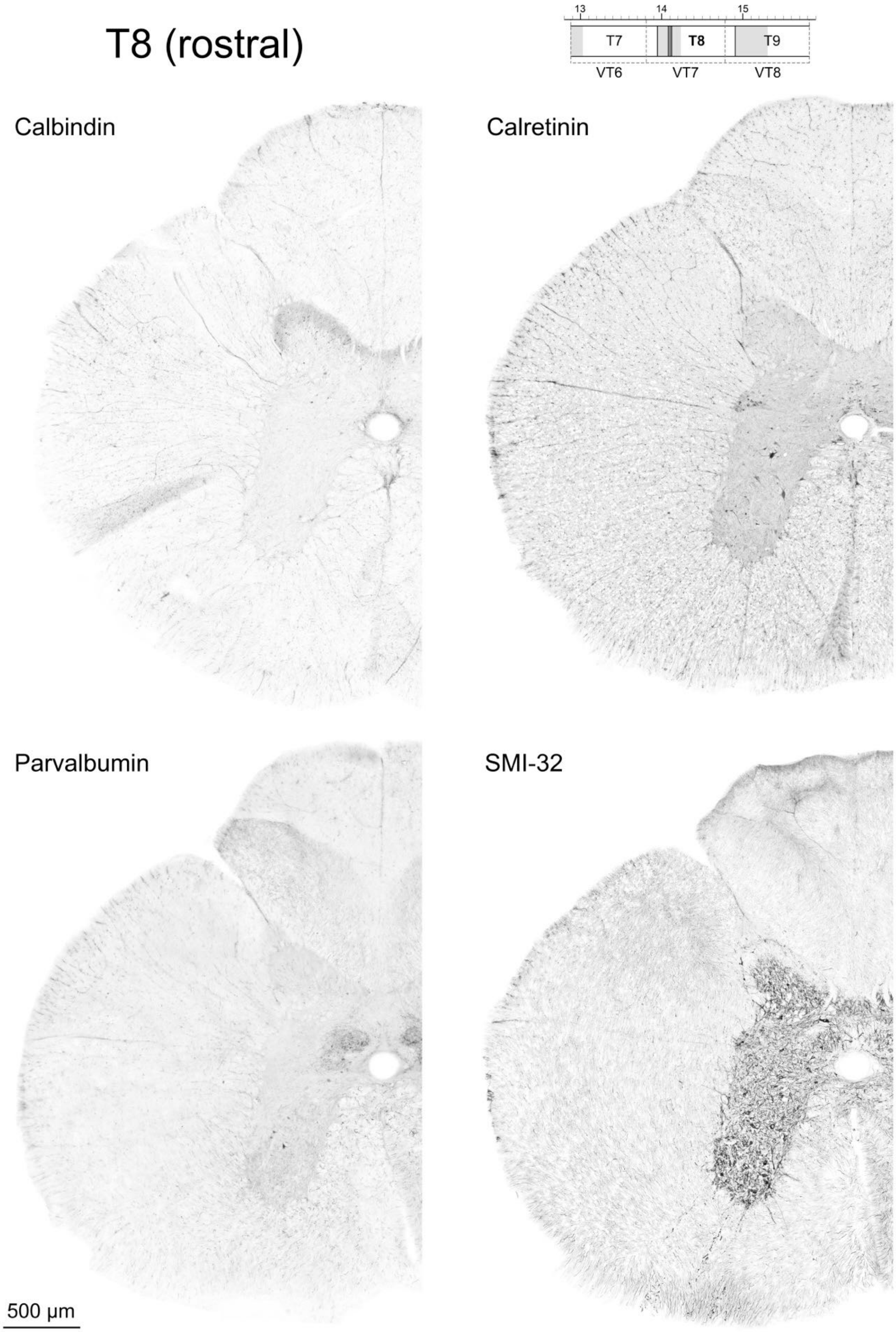

Supplementary Figure 16. Continued.

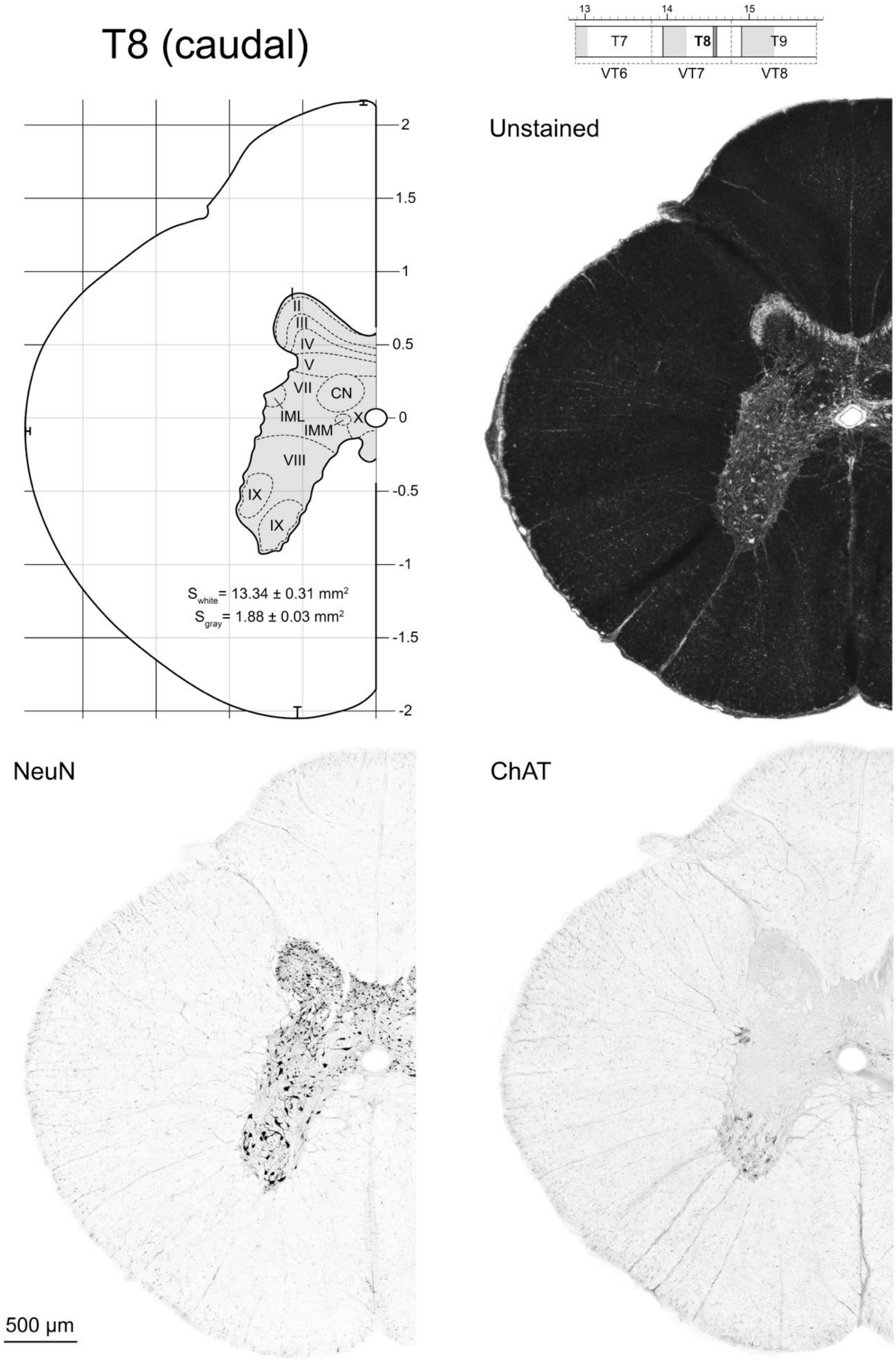

Supplementary Figure 17. Caudal part of T8 segment of the cat spinal cord.

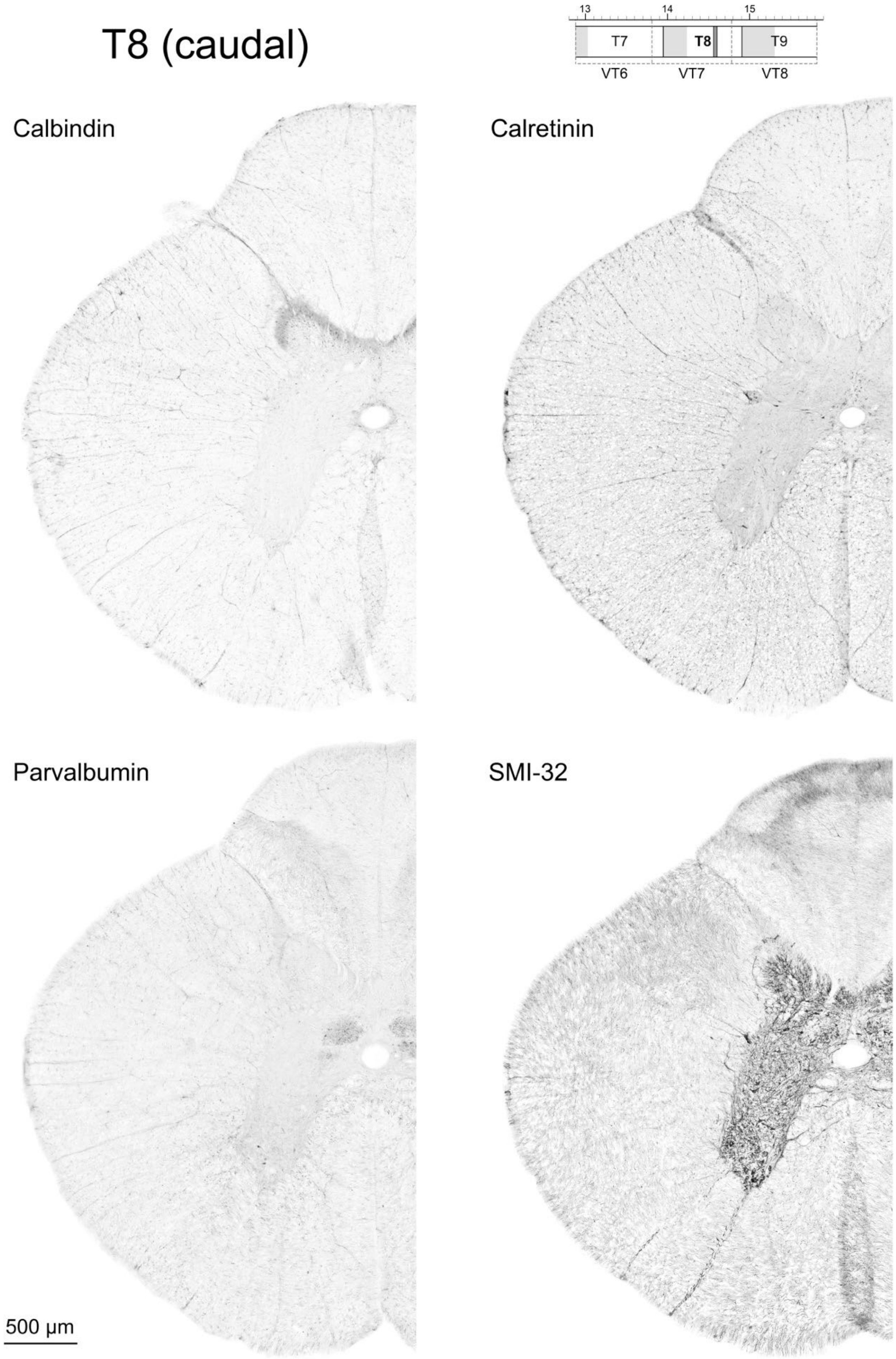

Supplementary Figure 17. Continued.

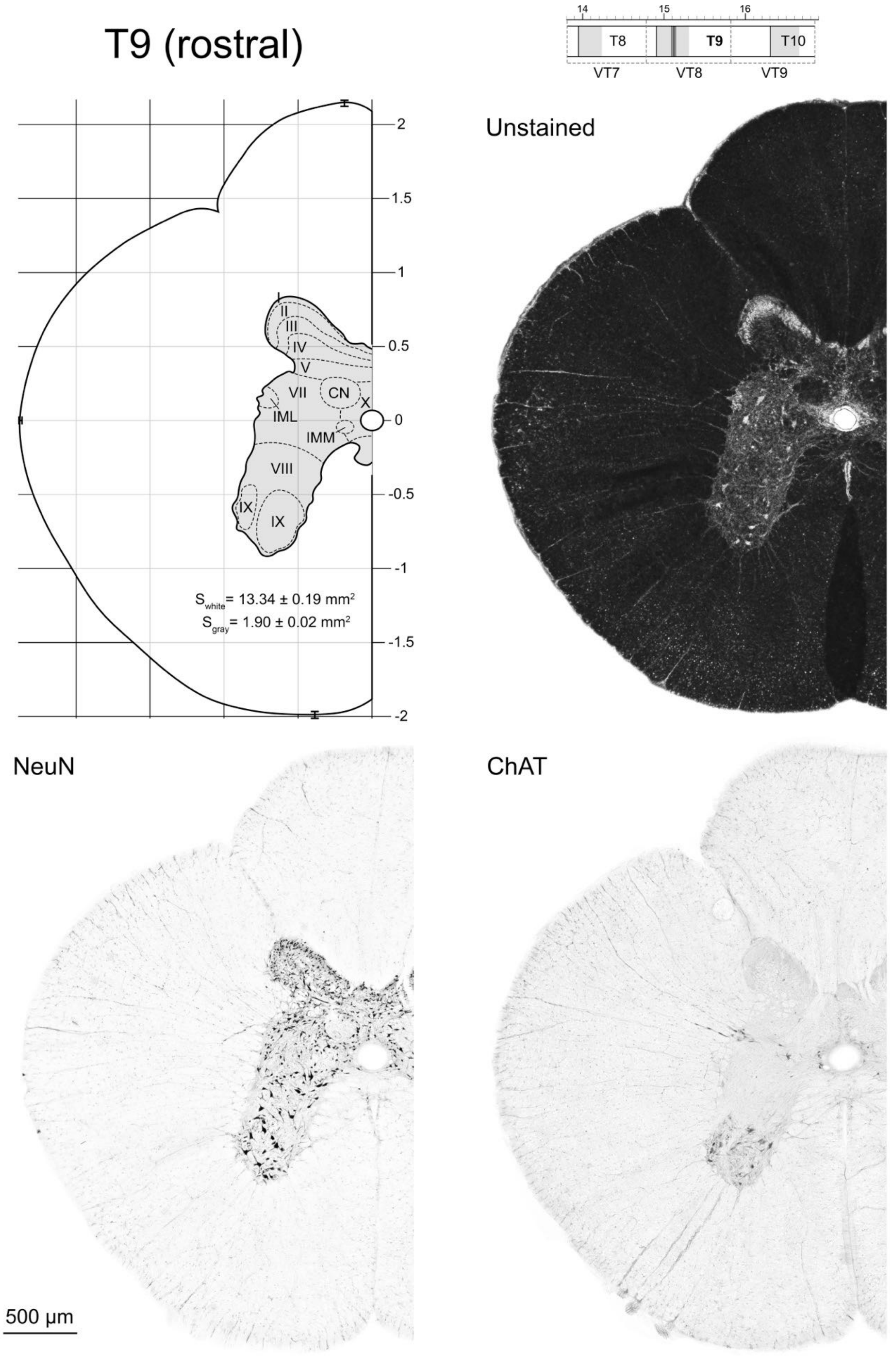

Supplementary Figure 18. Rostral part of T9 segment of the cat spinal cord.

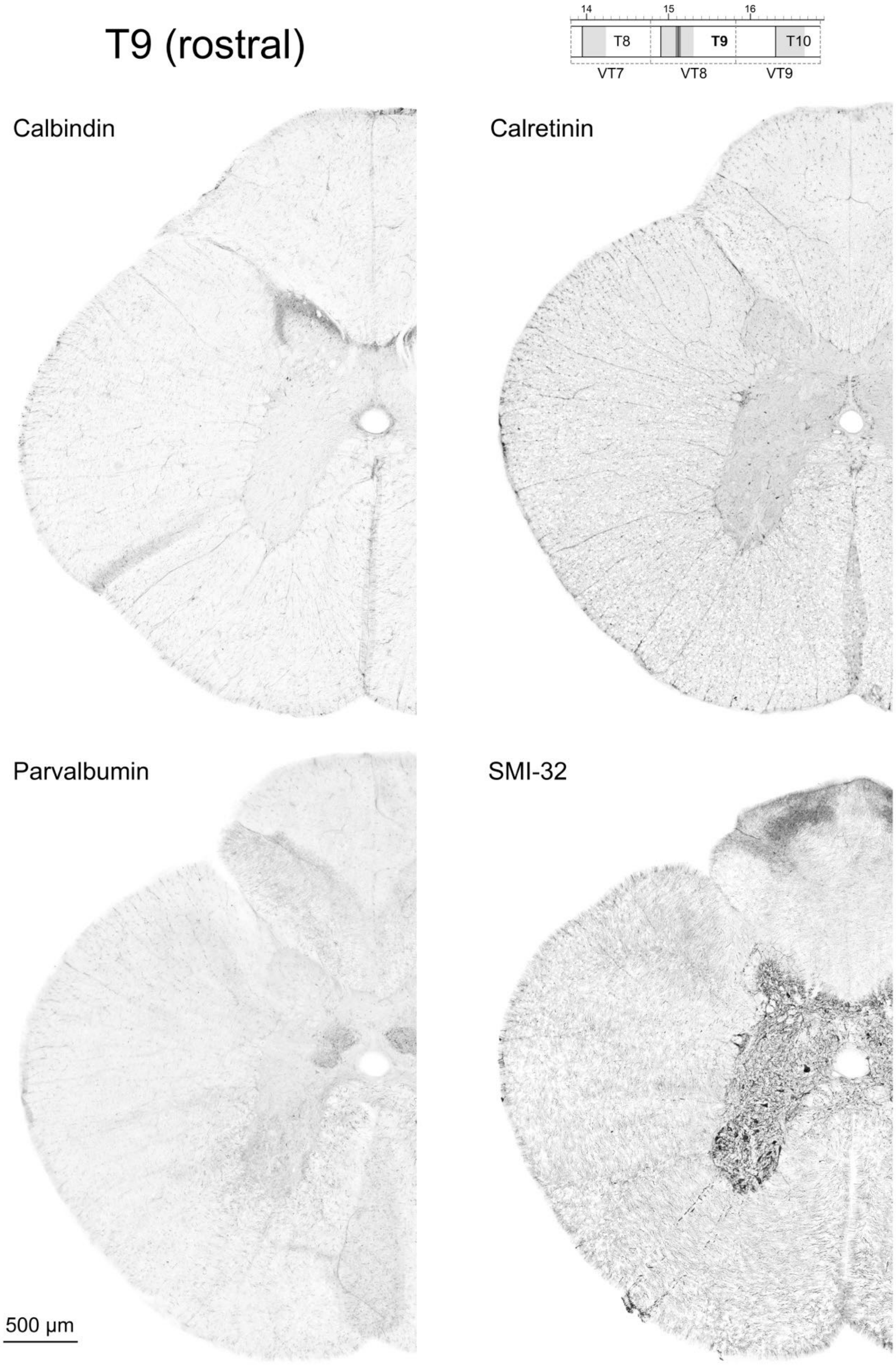

Supplementary Figure 18. Continued.

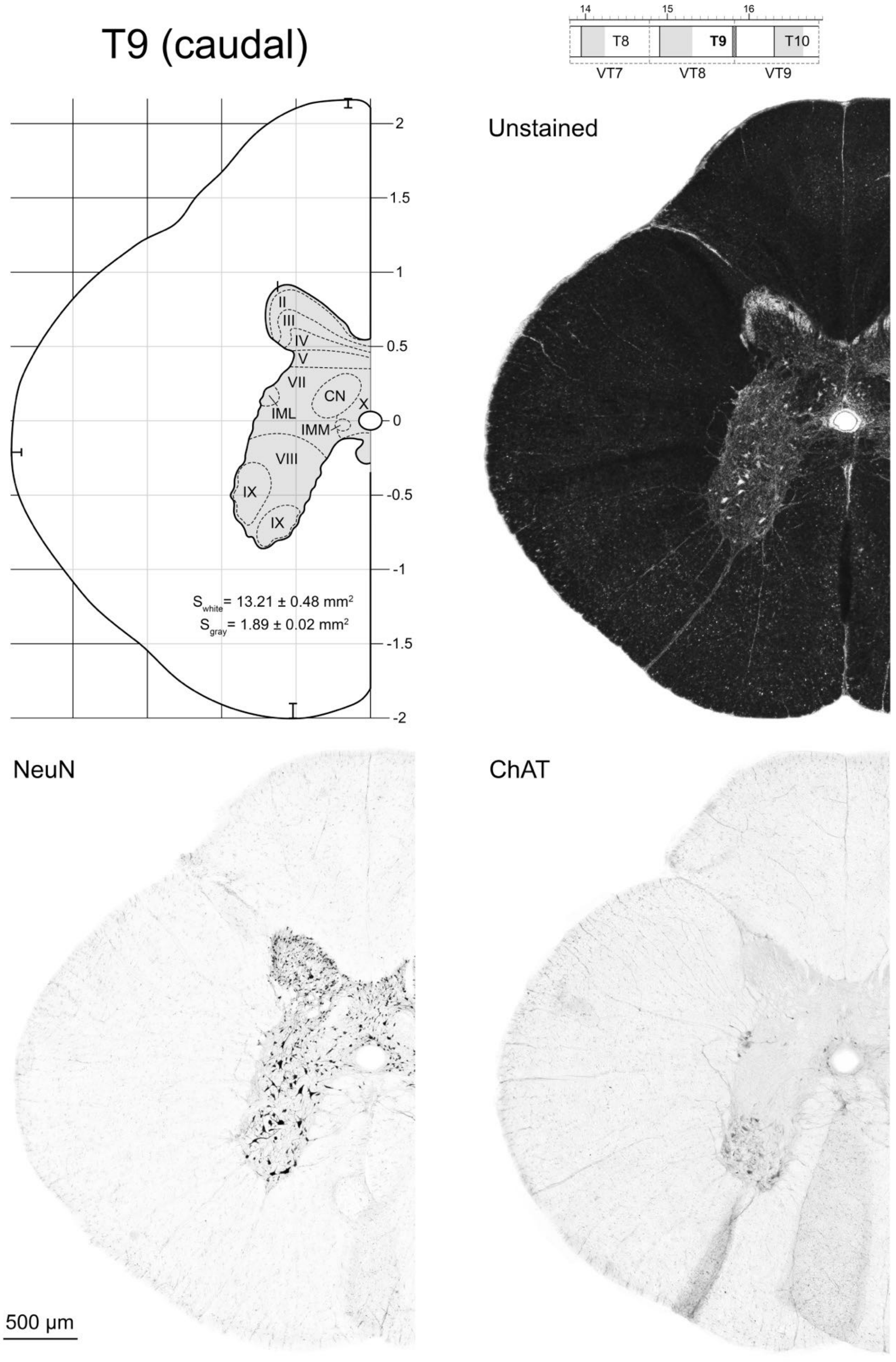

Supplementary Figure 19. Caudal part of T9 segment of the cat spinal cord.

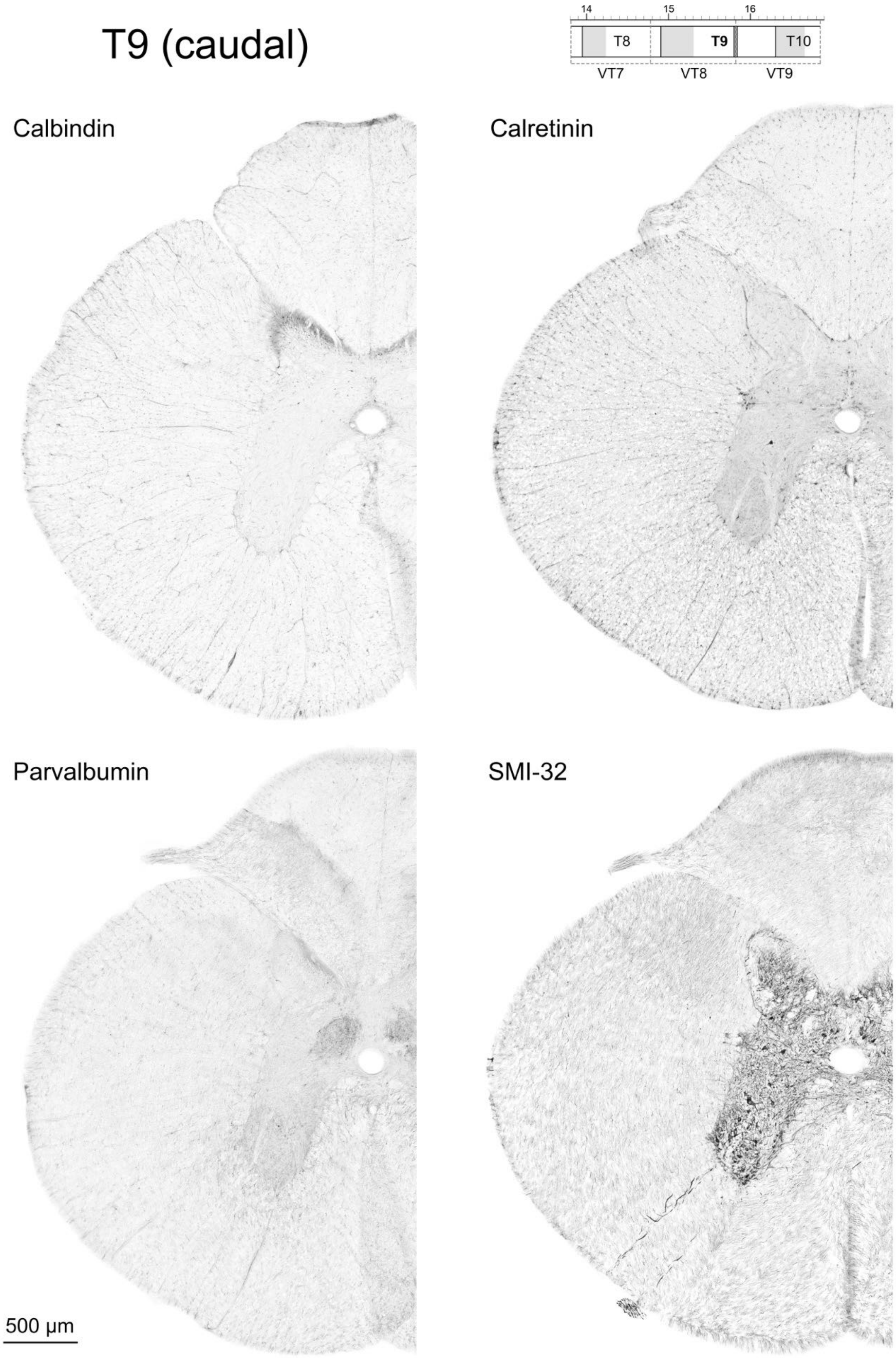

Supplementary Figure 19. Continued.

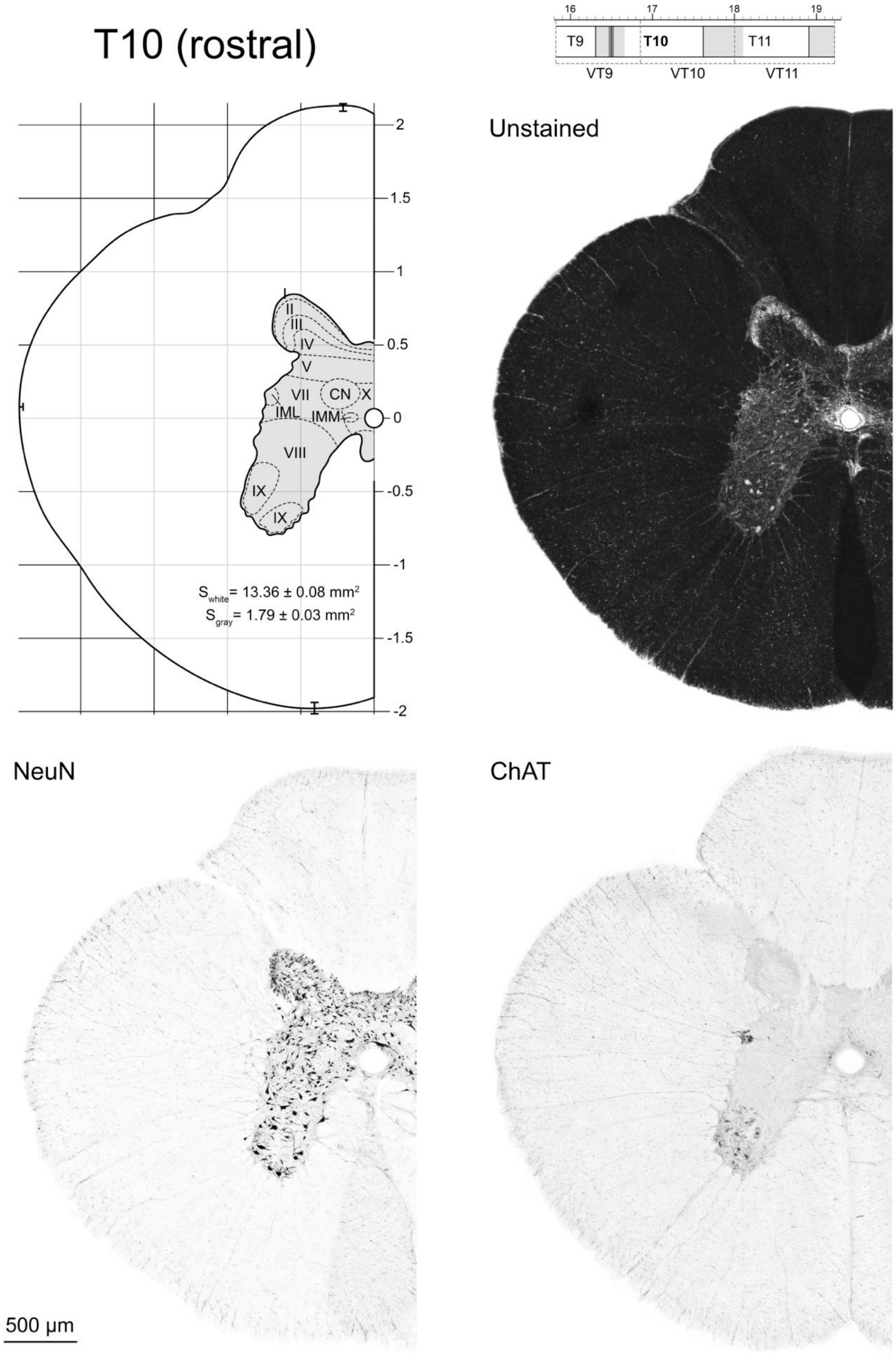

Supplementary Figure 20. Rostral part of T10 segment of the cat spinal cord.

T10 (rostral)

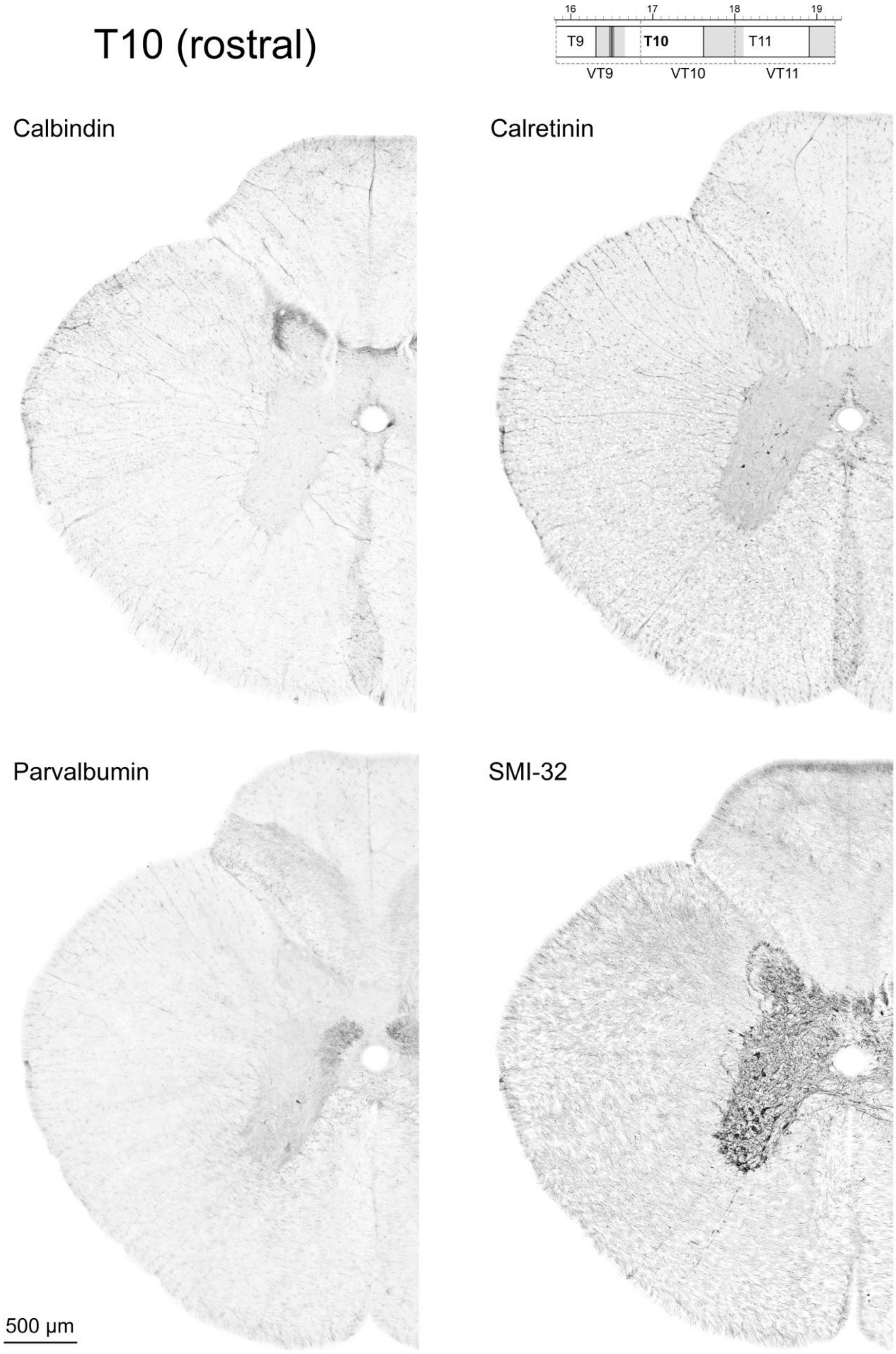

Supplementary Figure 20. Continued.

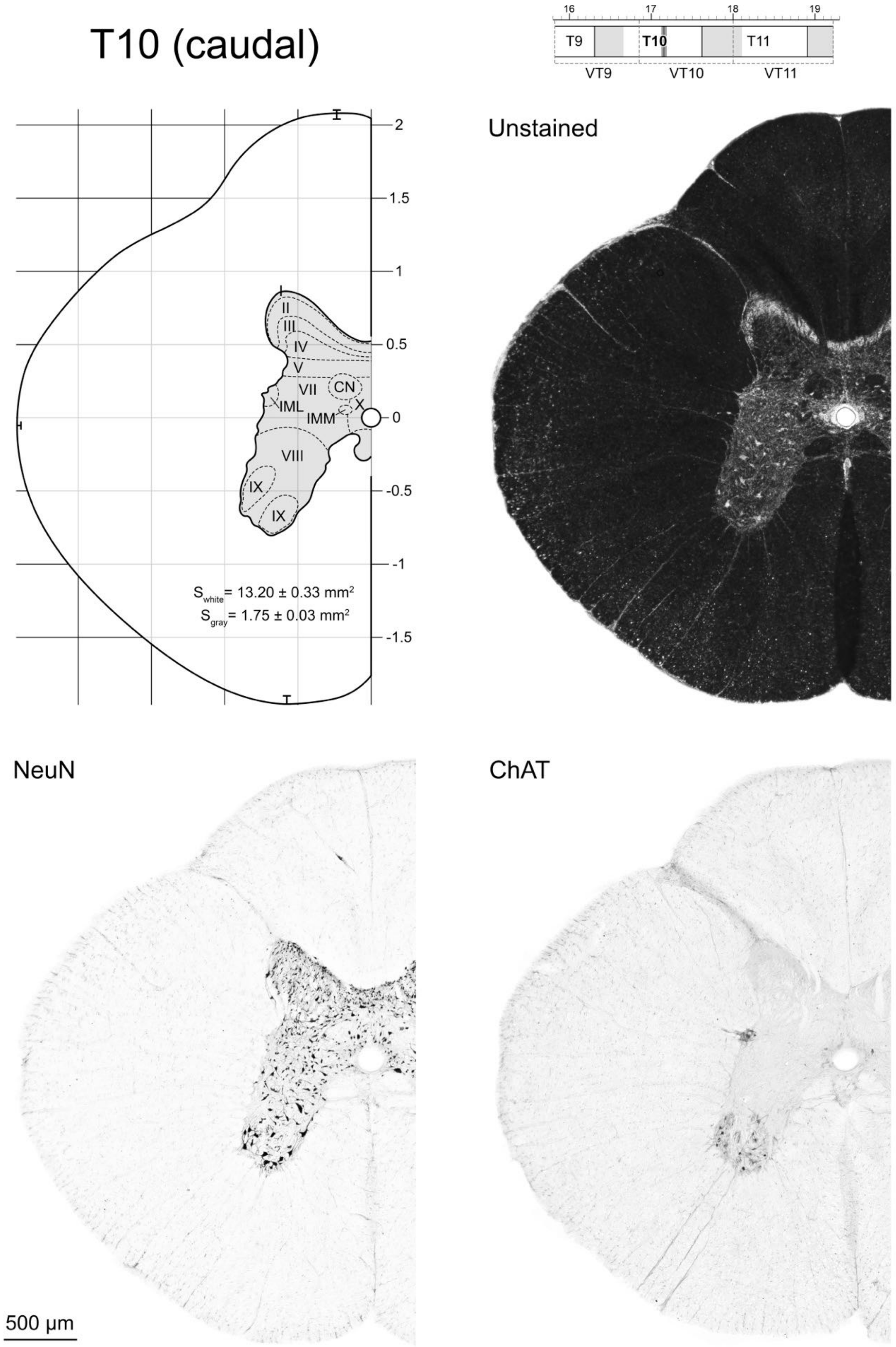

Supplementary Figure 21. Caudal part of T10 segment of the cat spinal cord.

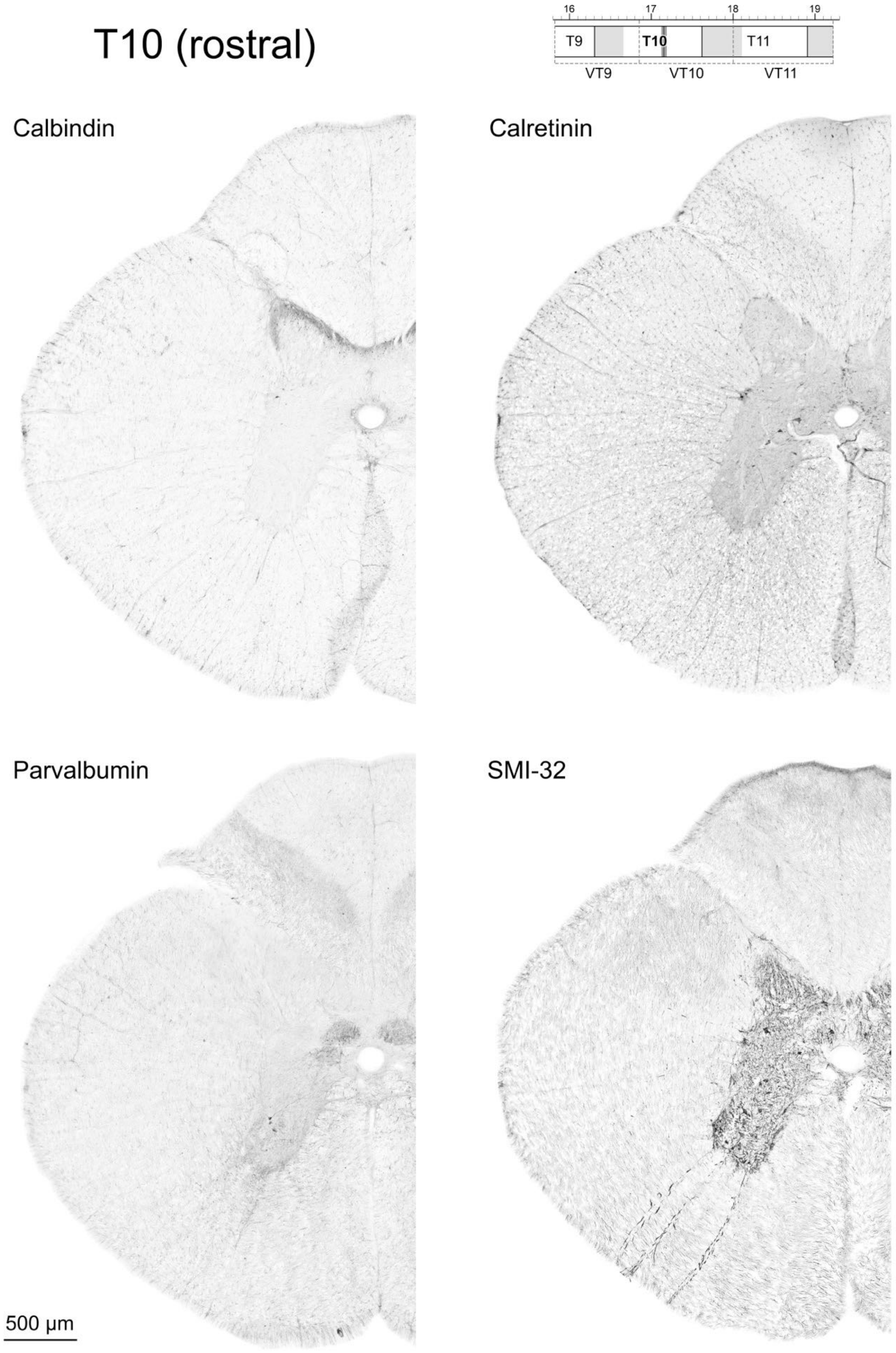

Supplementary Figure 21. Continued.

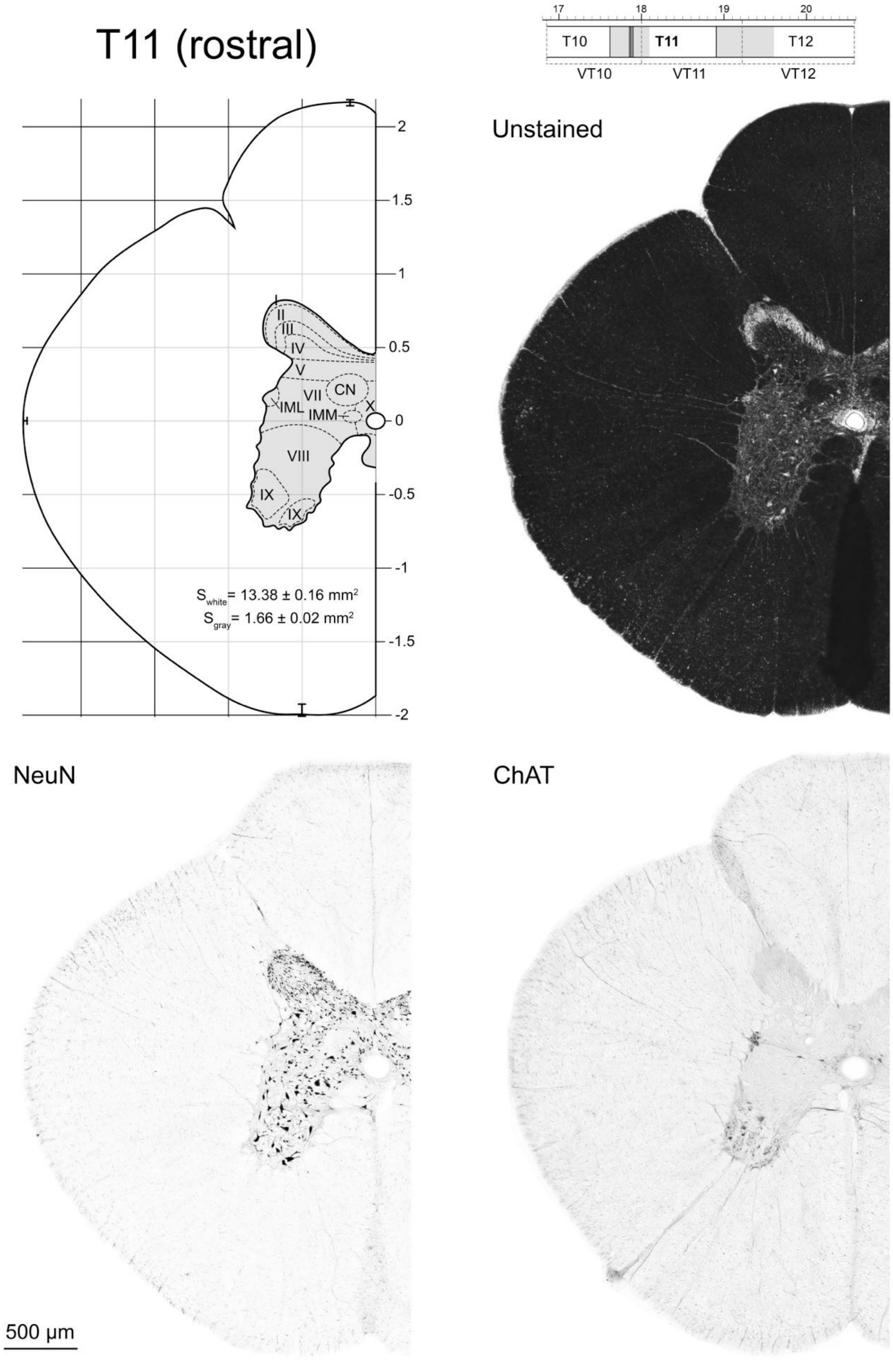

Supplementary Figure 22. Rostral part of T11 segment of the cat spinal cord.

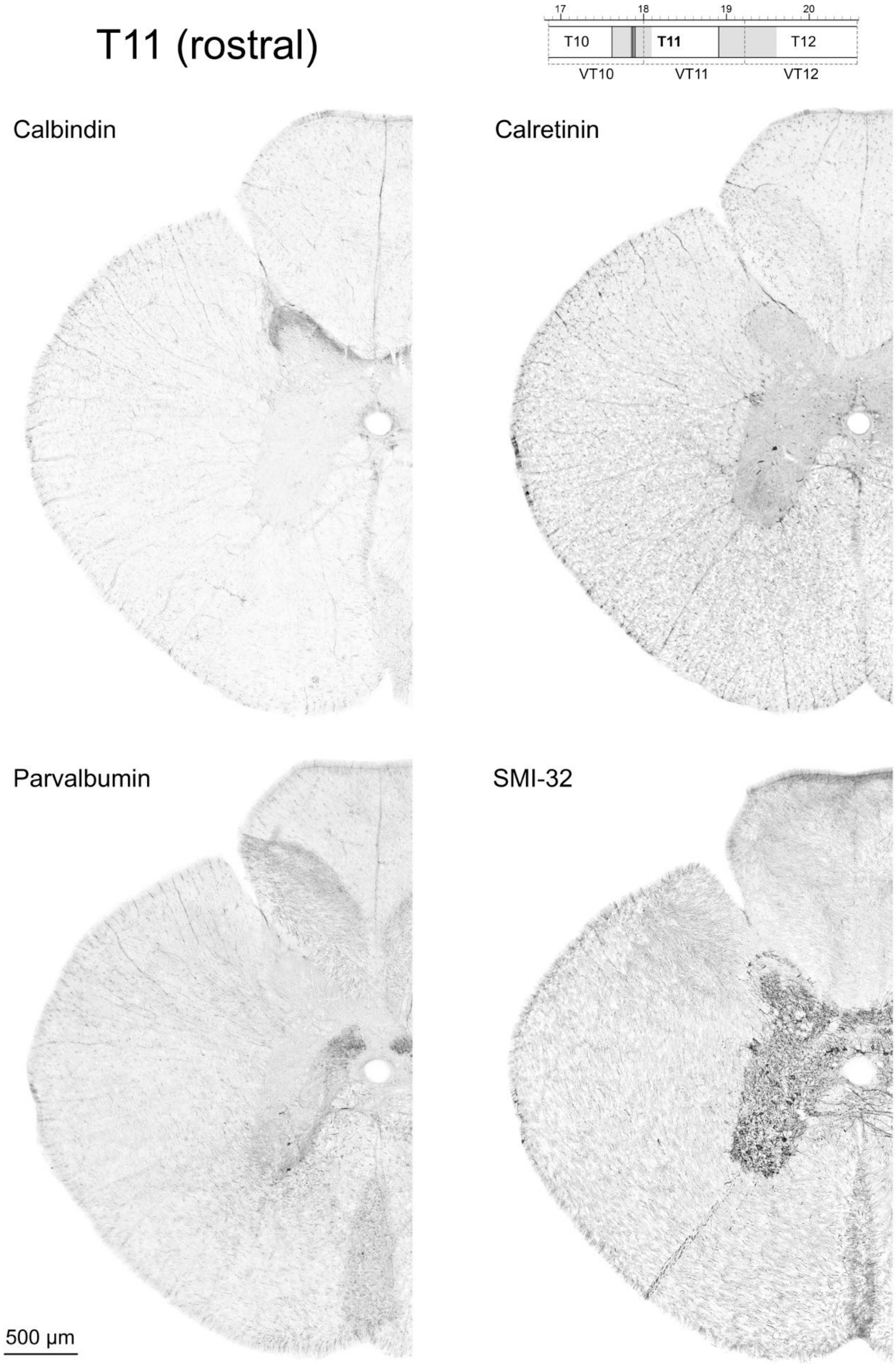

Supplementary Figure 22. Continued.

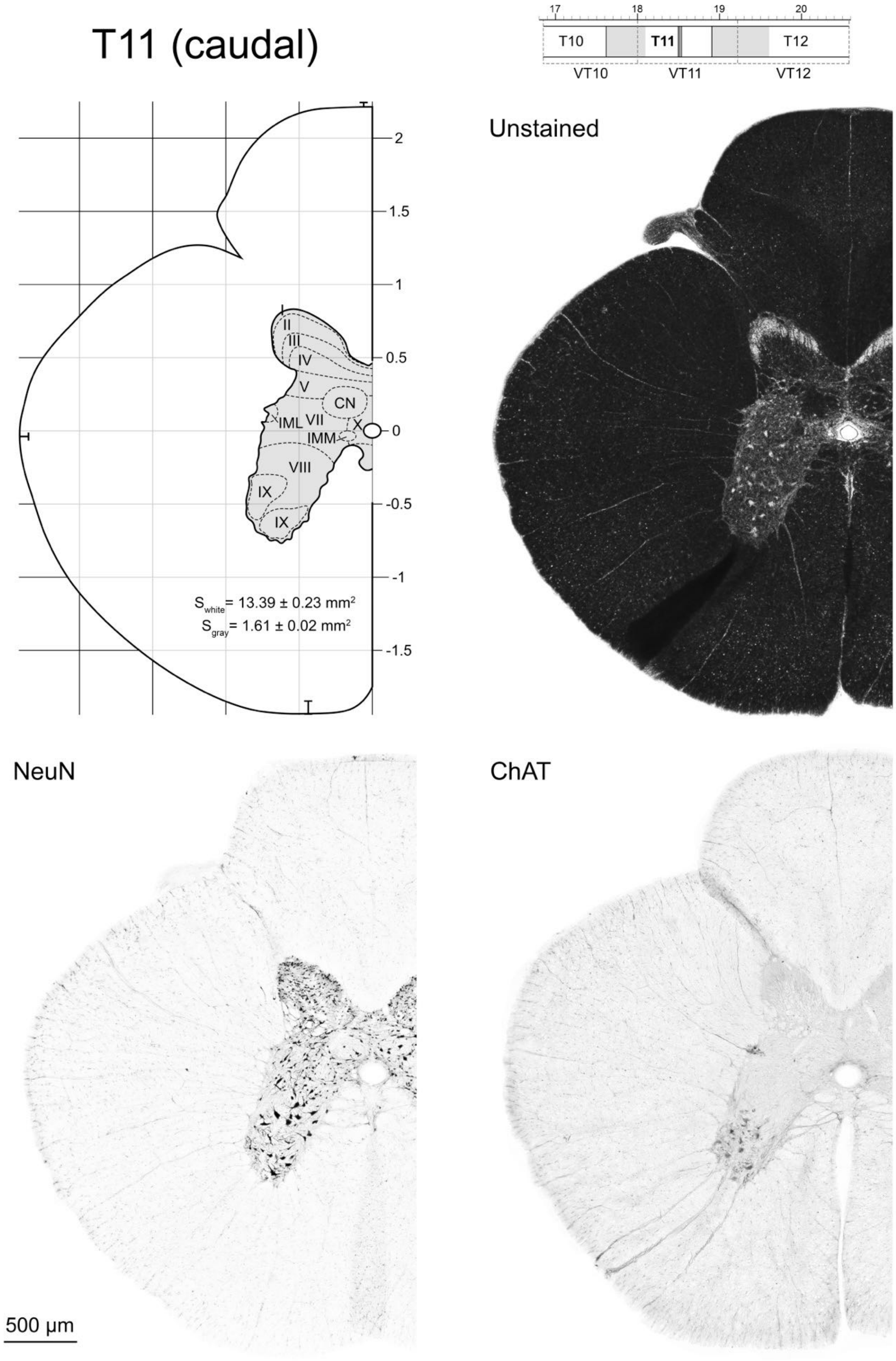

Supplementary Figure 23. Caudal part of T11 segment of the cat spinal cord.

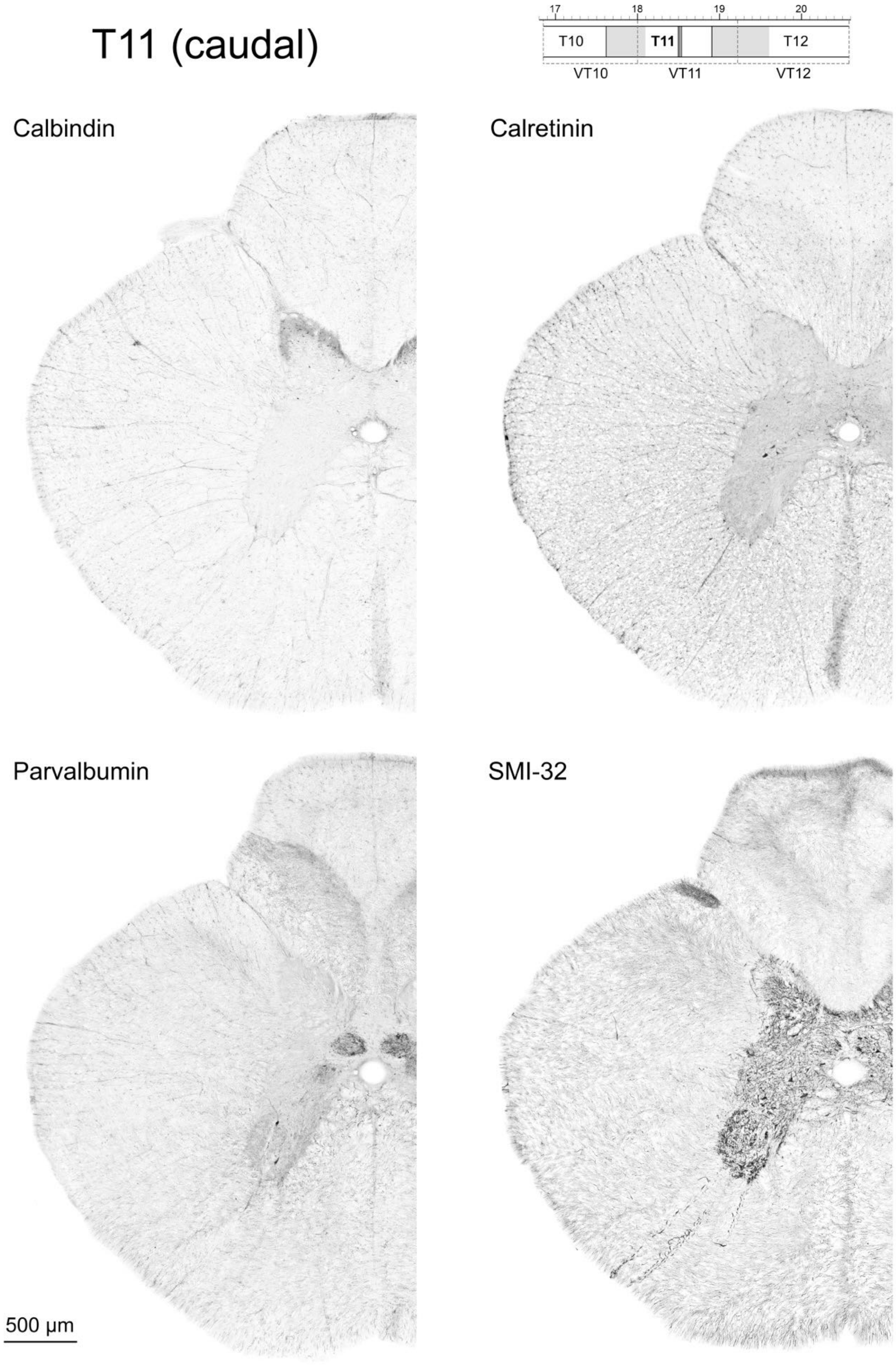

Supplementary Figure 23. Continued.

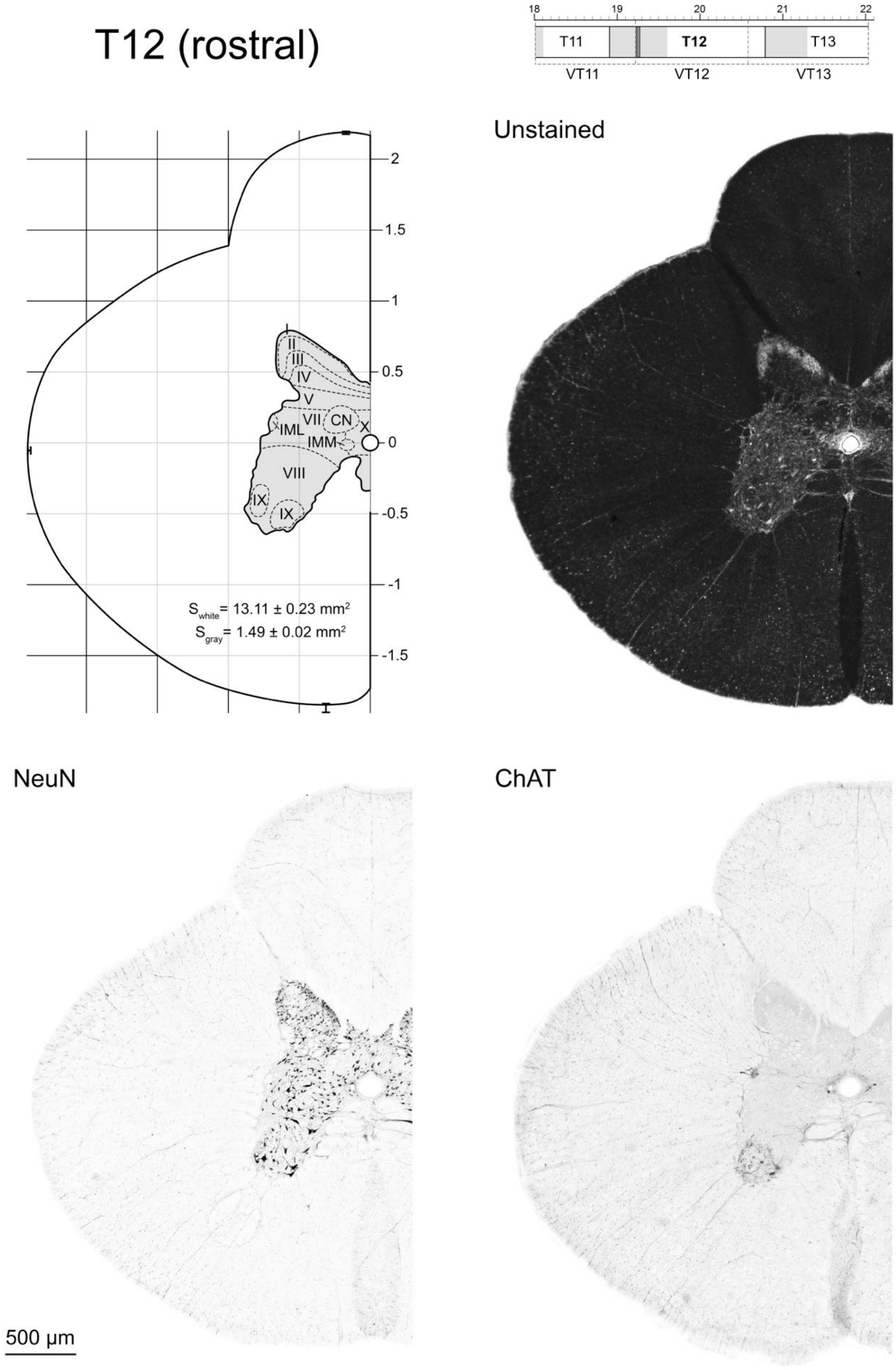

Supplementary Figure 24. Rostral part of T12 segment of the cat spinal cord.

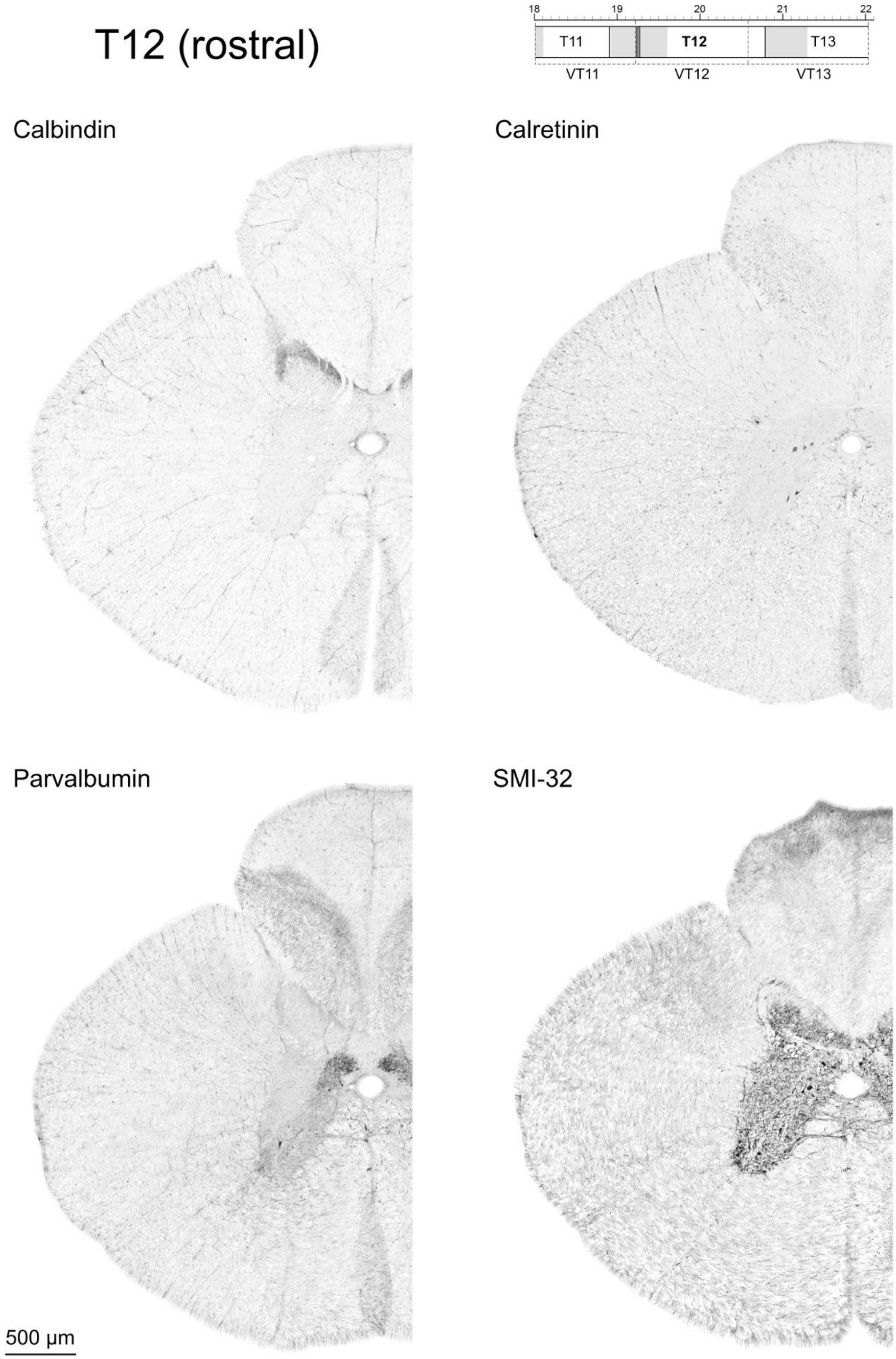

Supplementary Figure 24. Continued.

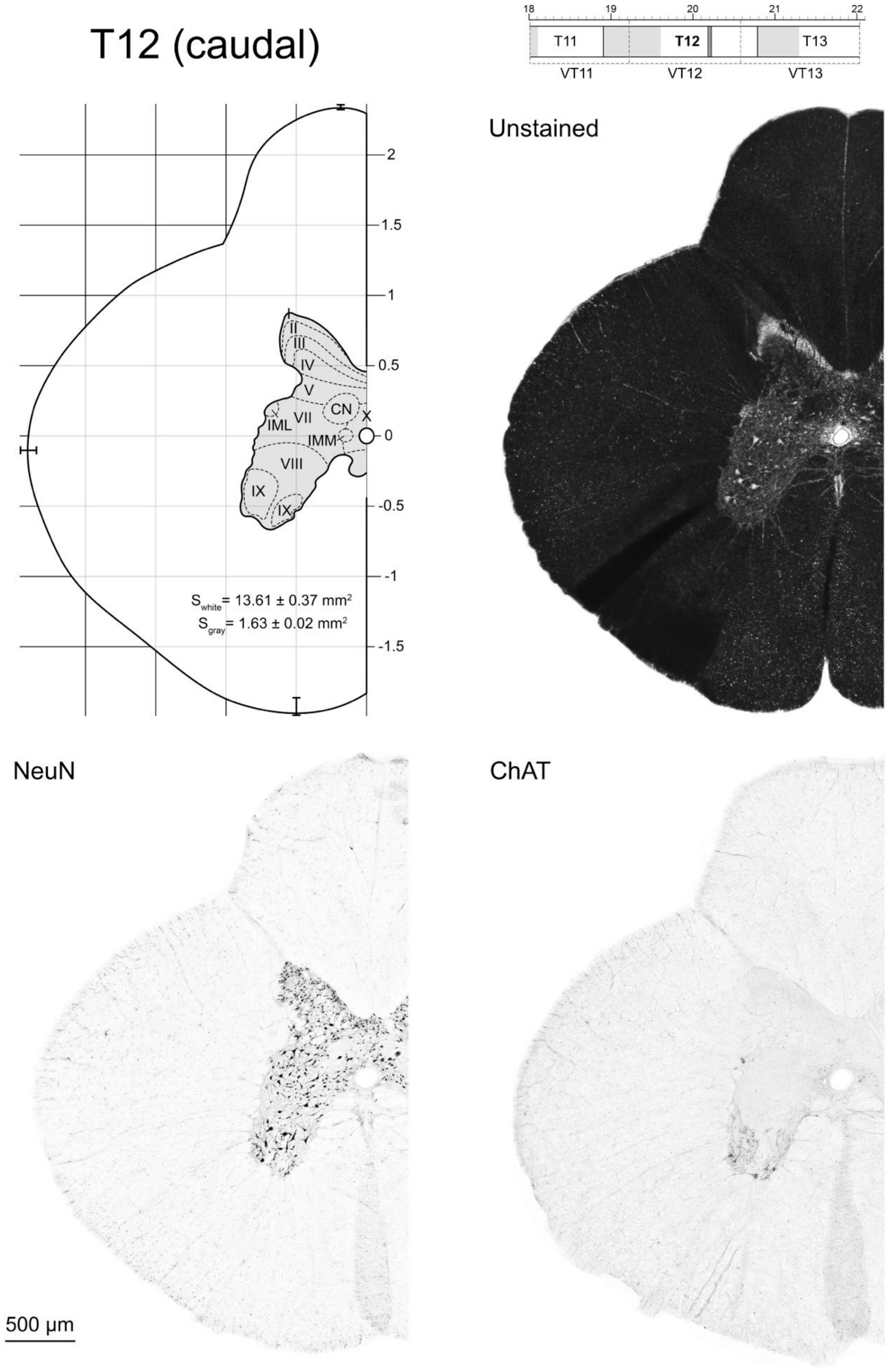

Supplementary Figure 25. Caudal part of T12 segment of the cat spinal cord.

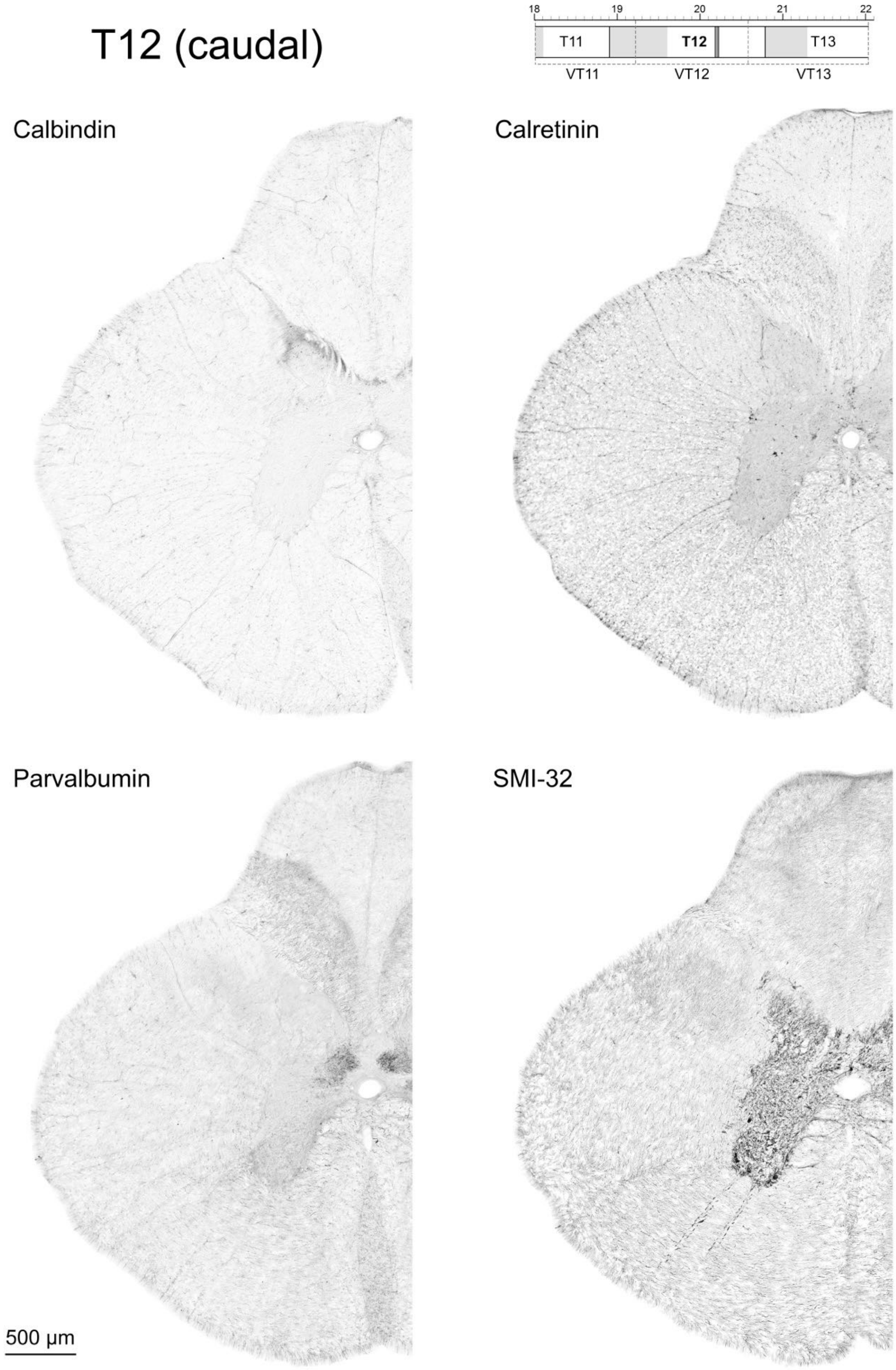

Supplementary Figure 25. Continued.

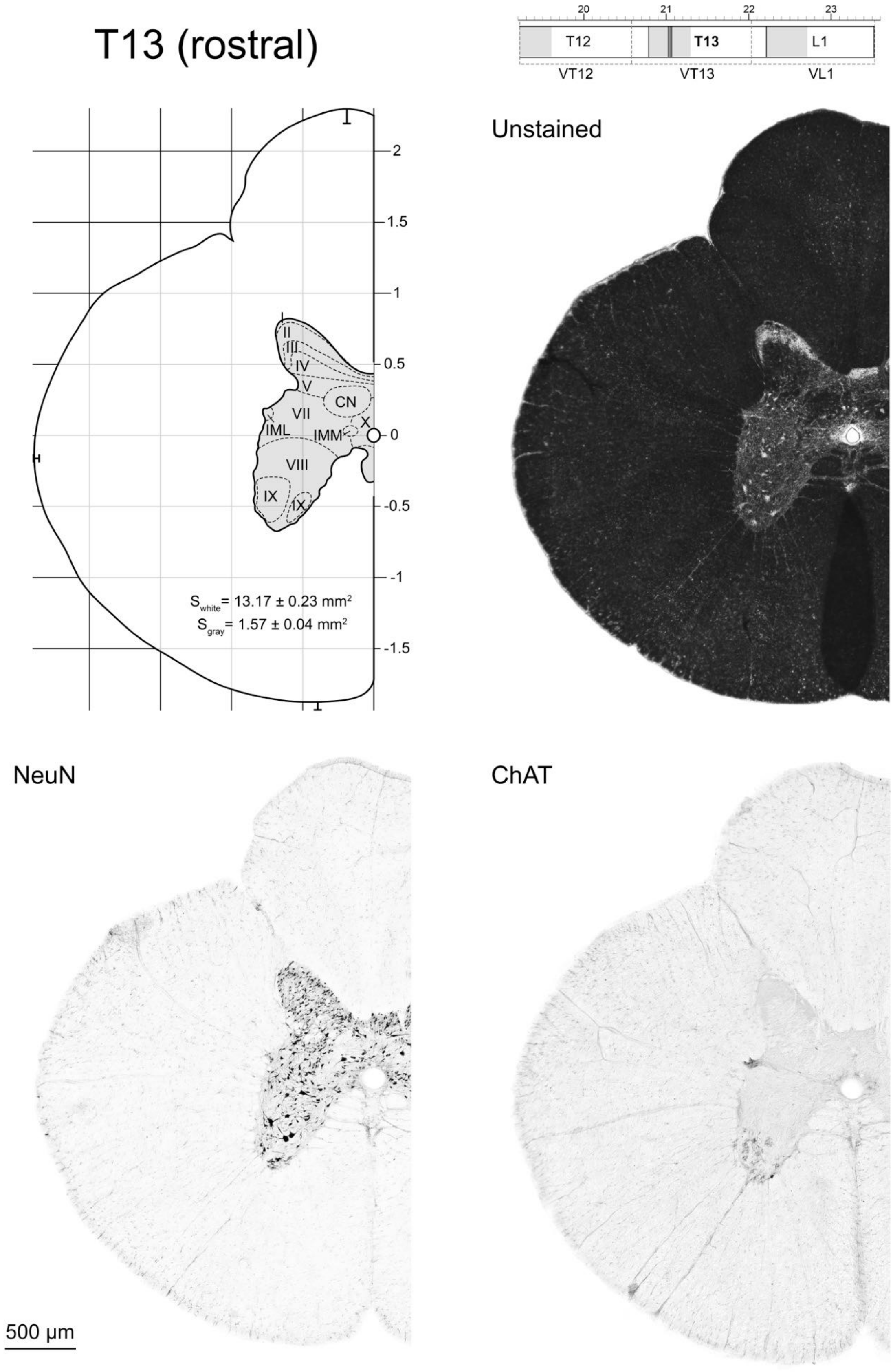

Supplementary Figure 26. Rostral part of T13 segment of the cat spinal cord.

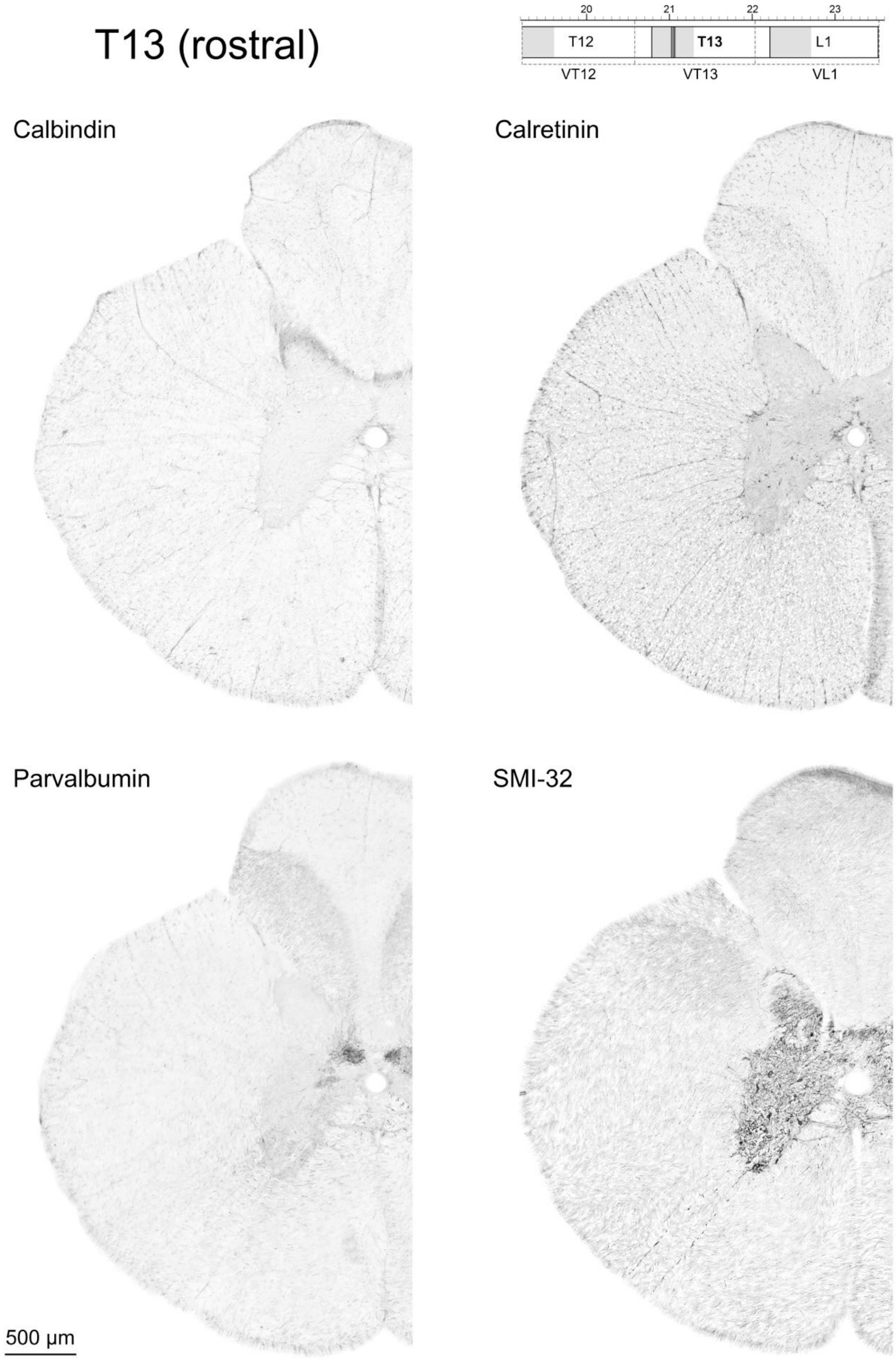

Supplementary Figure 26. Continued.

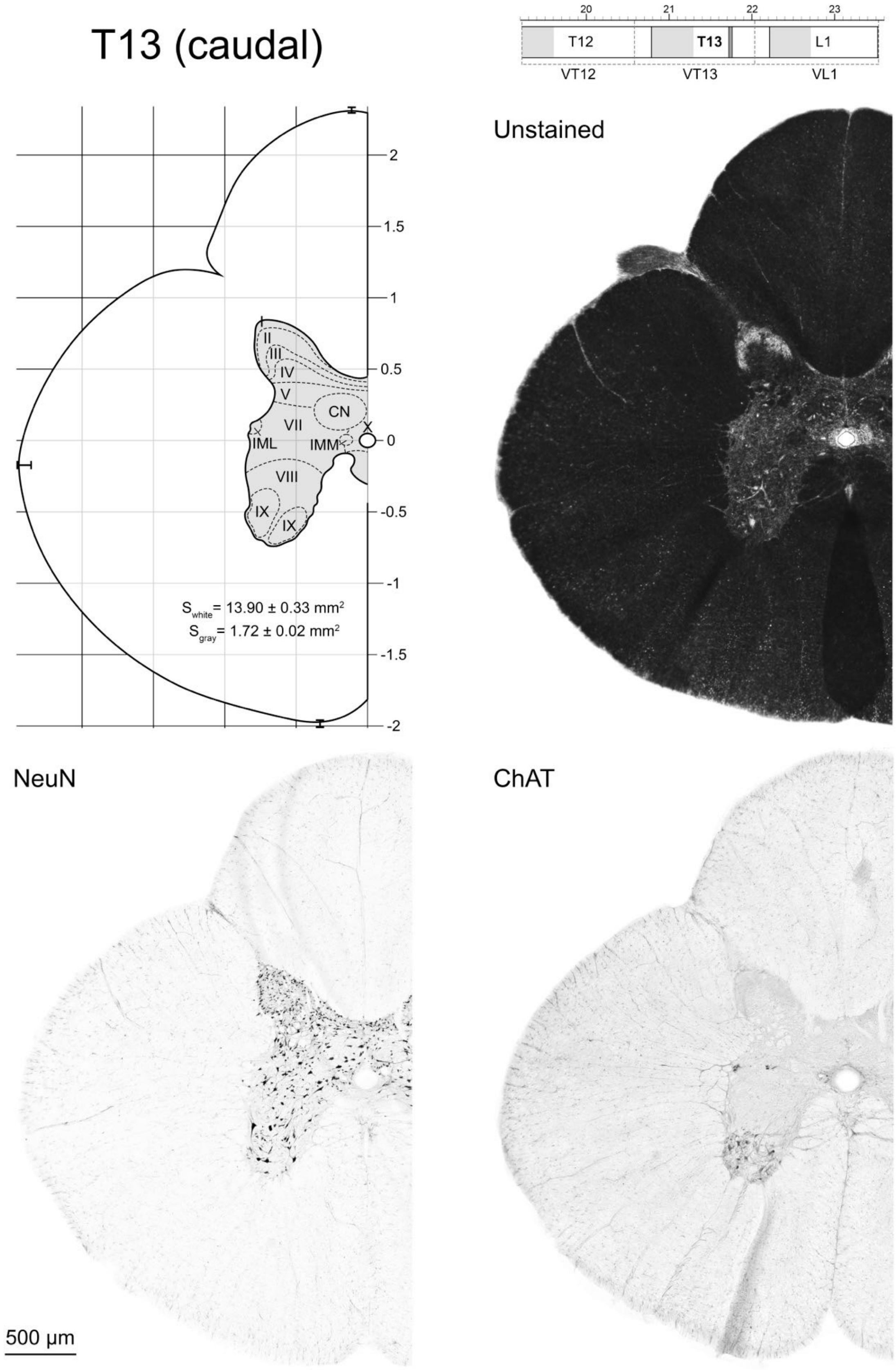

Supplementary Figure 27. Caudal part of T13 segment of the cat spinal cord.

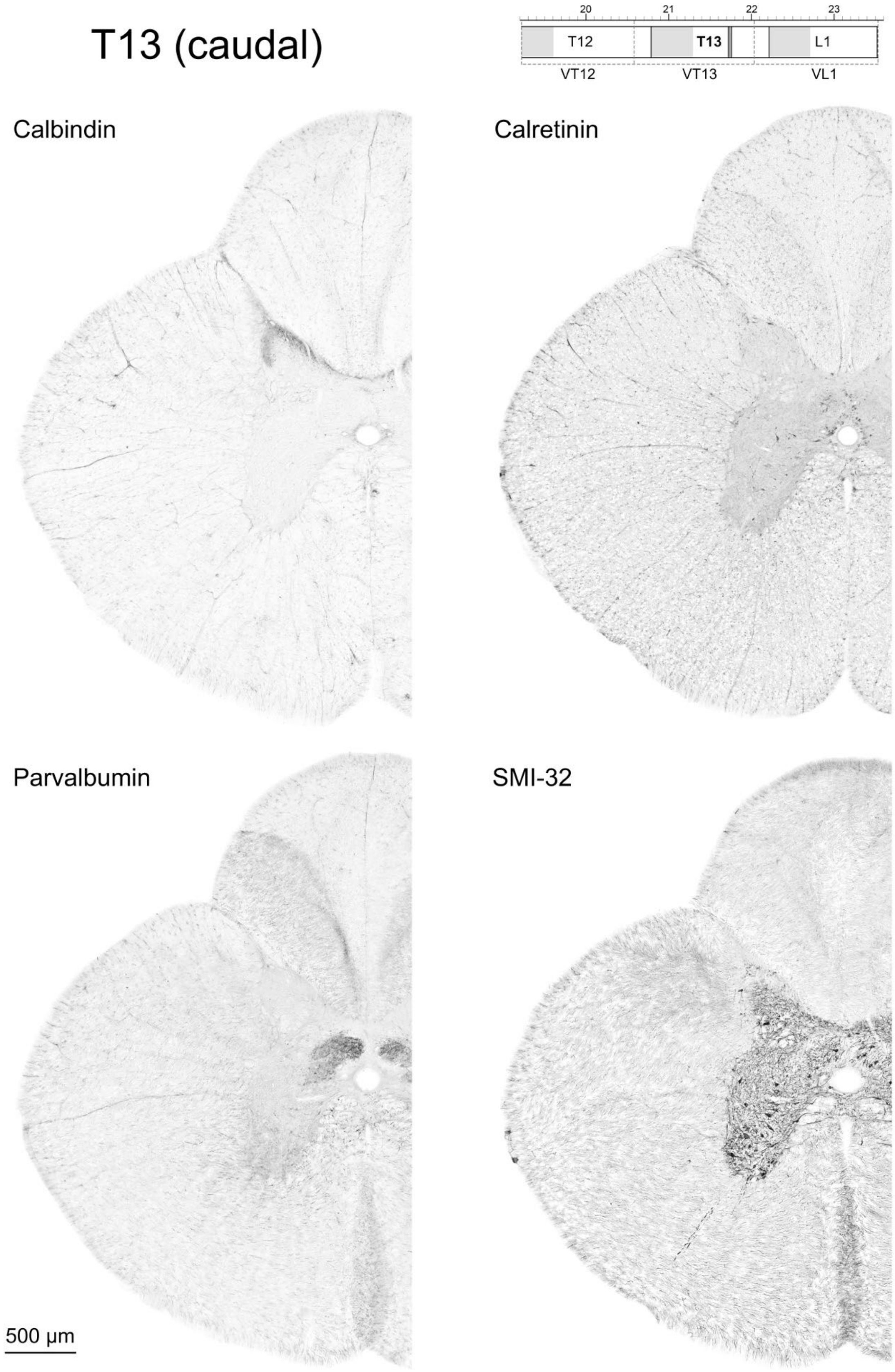

Supplementary Figure 27. Continued.
