# Supplementary material for: Neurochemical atlas of the cat spinal cord
Source: Front Neuroanat. 2022 Oct 19;16:1034395. doi: 10.3389/fnana.2022.1034395 (PMC9627295; doi:10.3389/fnana.2022.1034395)
Supplement: Supplementary file 3 [file Data_Sheet_3.PDF]

Supplementary Material

1 Abbreviations

I – lamina I

II – lamina II

III – lamina III

IV – lamina IV

V – lamina V

VI – lamina VI

VII – lamina VII

VIII – lamina VIII

IX – lamina IX

X – lamina X

CN – Clarke's Nucleus

DGC – Dorsal Gray Commissure

IC – Intercalated Nucleus

IMM – Intermediomedial Nucleus

IML – Intermediolateral Nucleus

S_{white} – area of the white matter

S_{gray} – area of the gray matter

2 Supplementary Figures

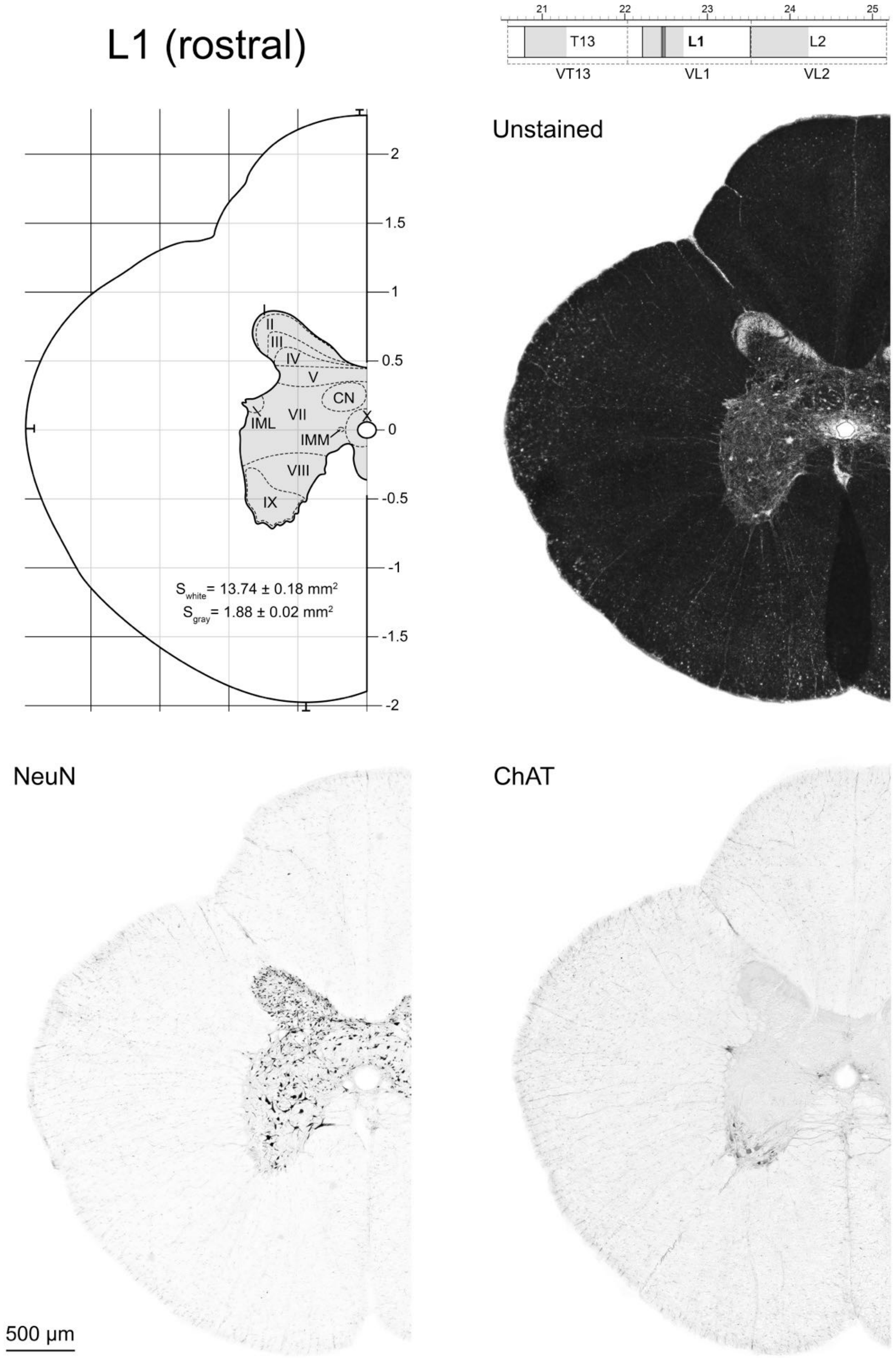

Supplementary Figure 1. Rostral part of L1 segment of the cat spinal cord.

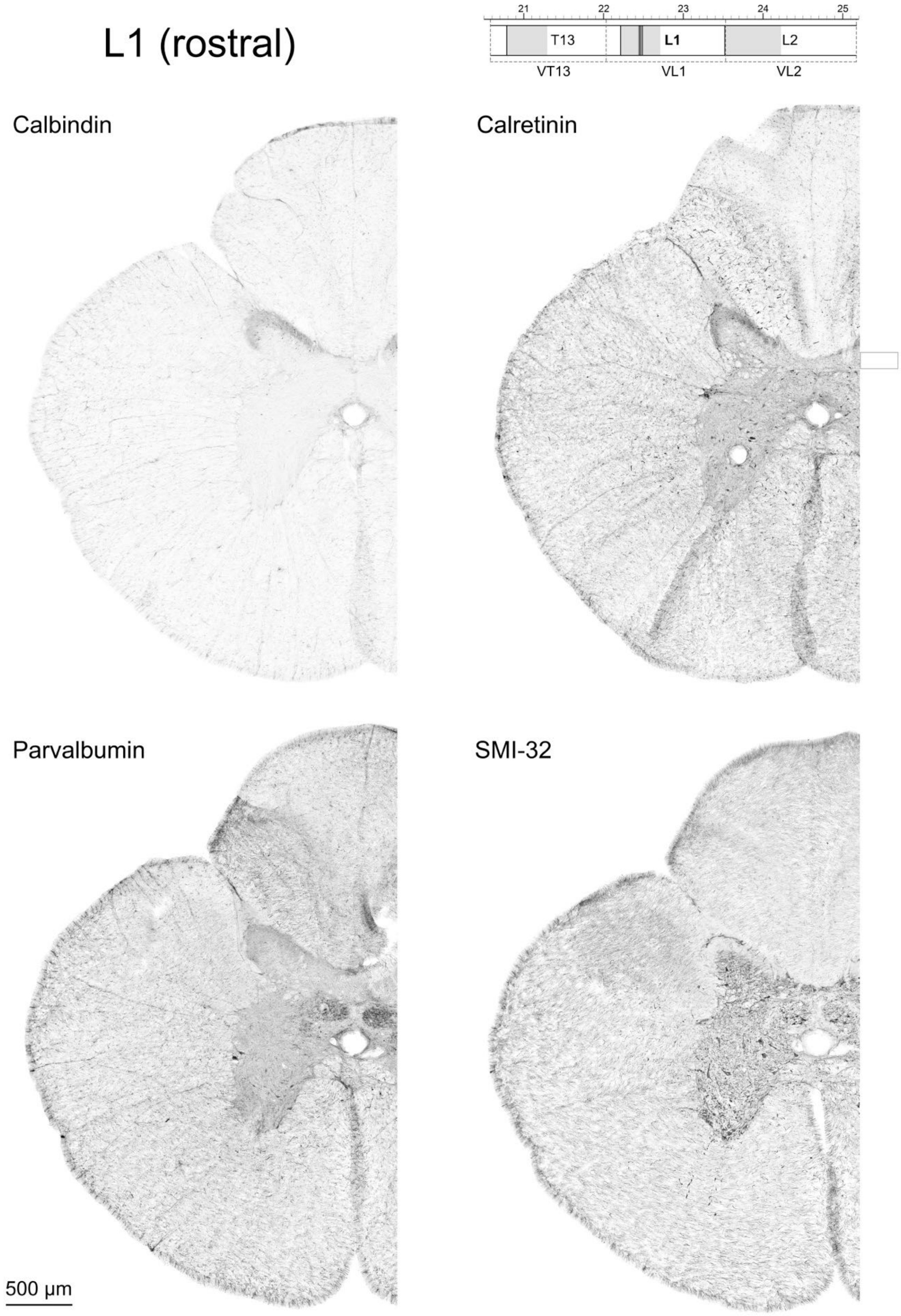

Supplementary Figure 1. Continued.

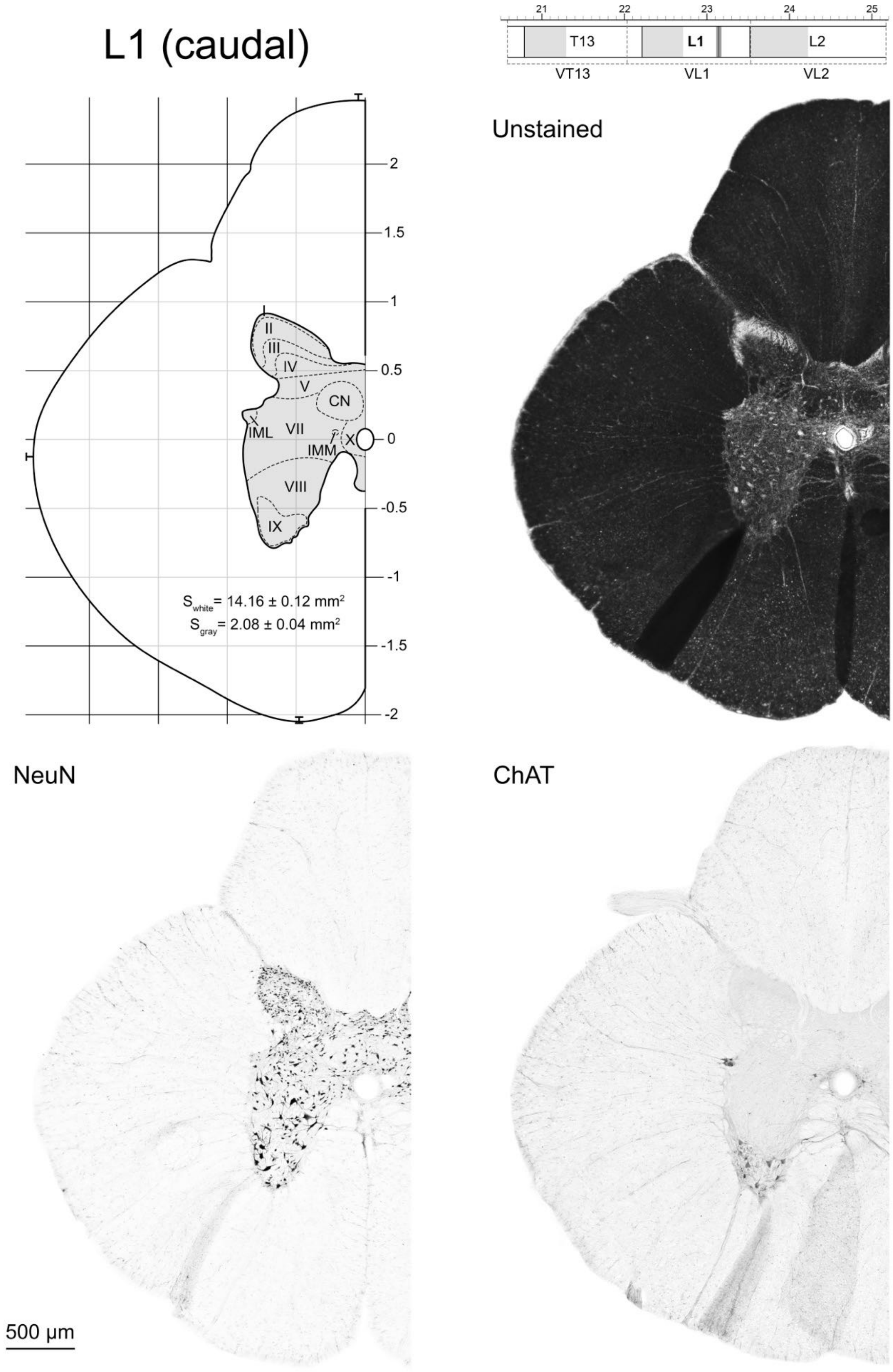

Supplementary Figure 2. Caudal part of L1 segment of the cat spinal cord.

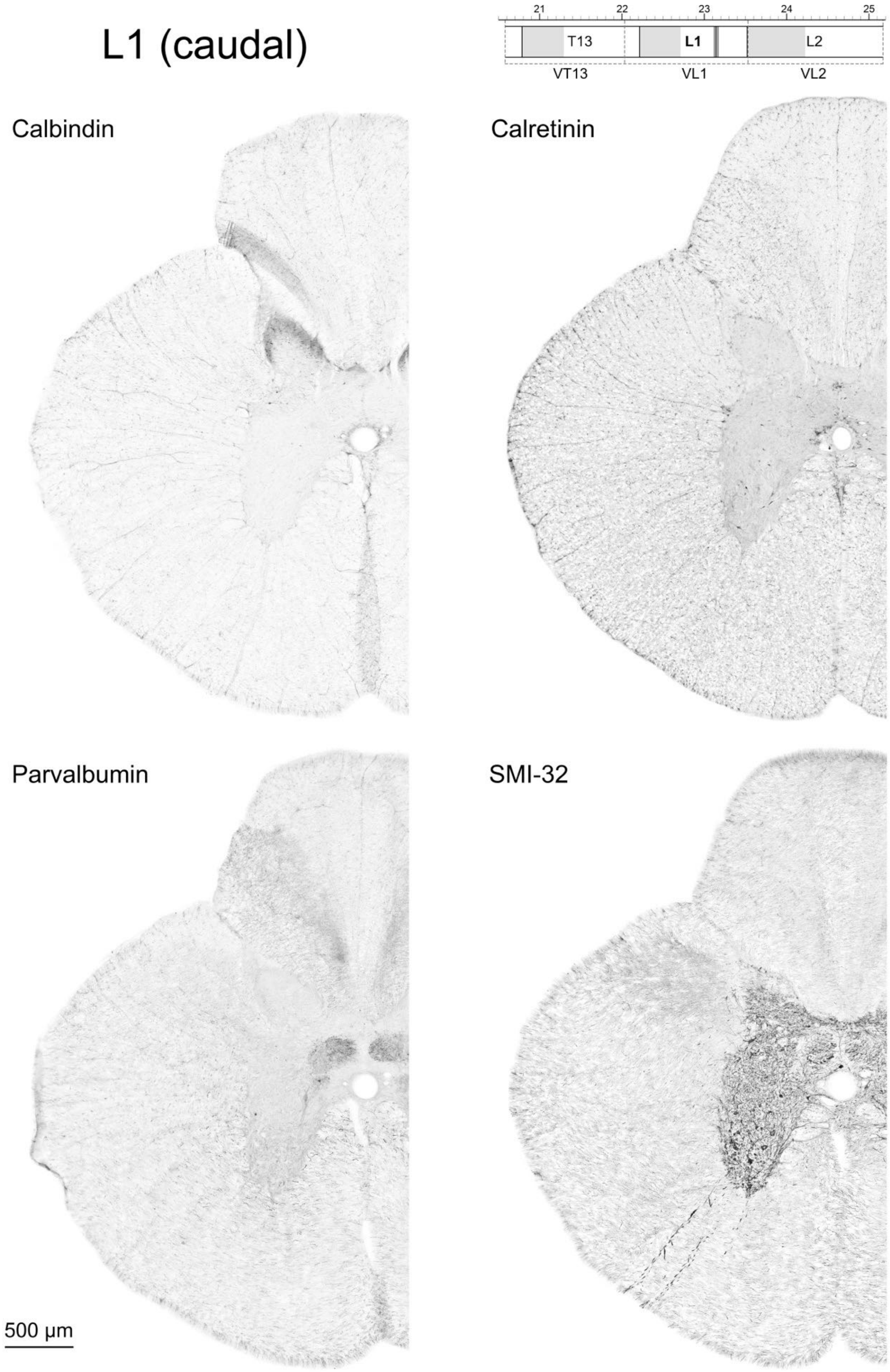

Supplementary Figure 2. Continued.

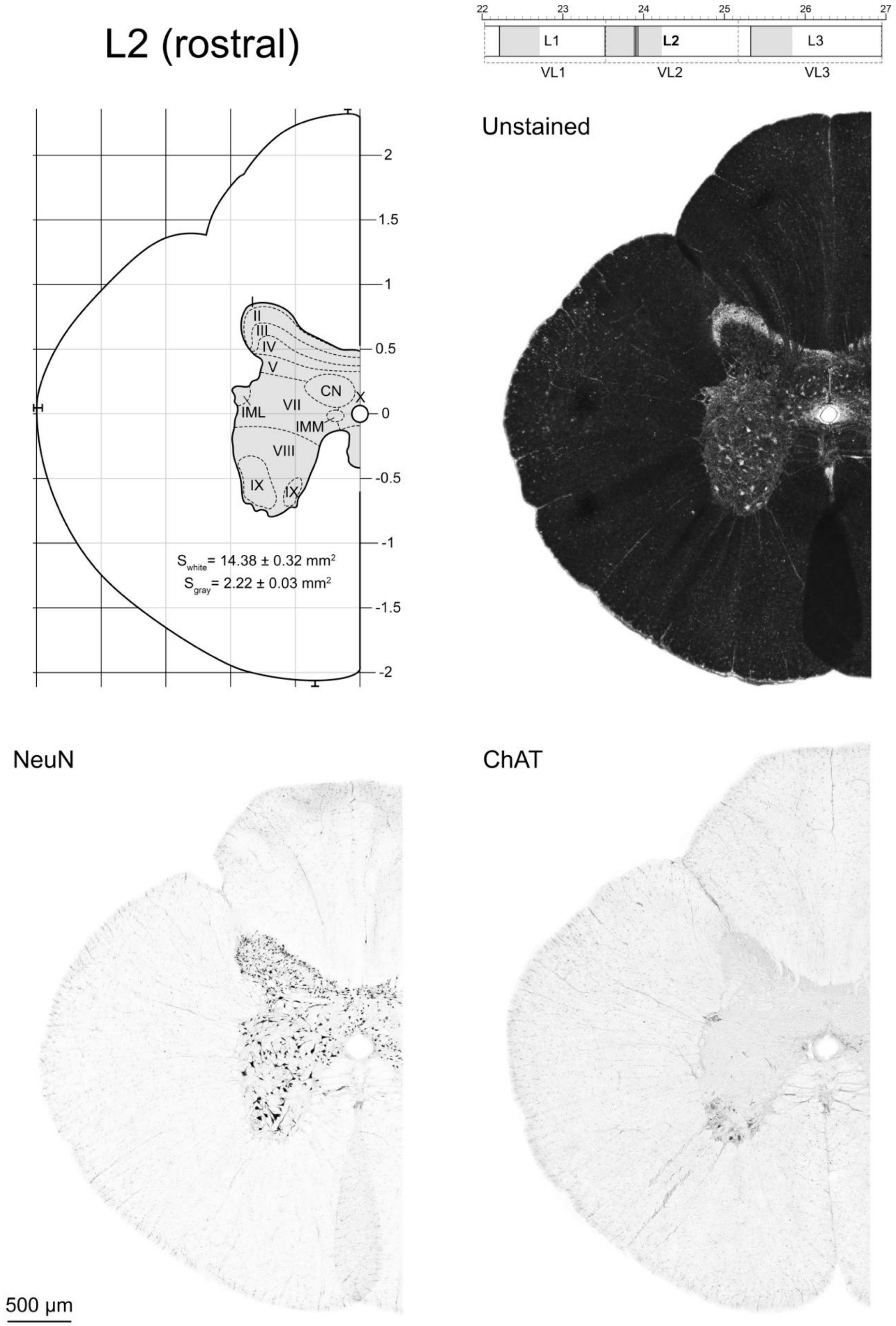

Supplementary Figure 3. Rostral part of L2 segment of the cat spinal cord.

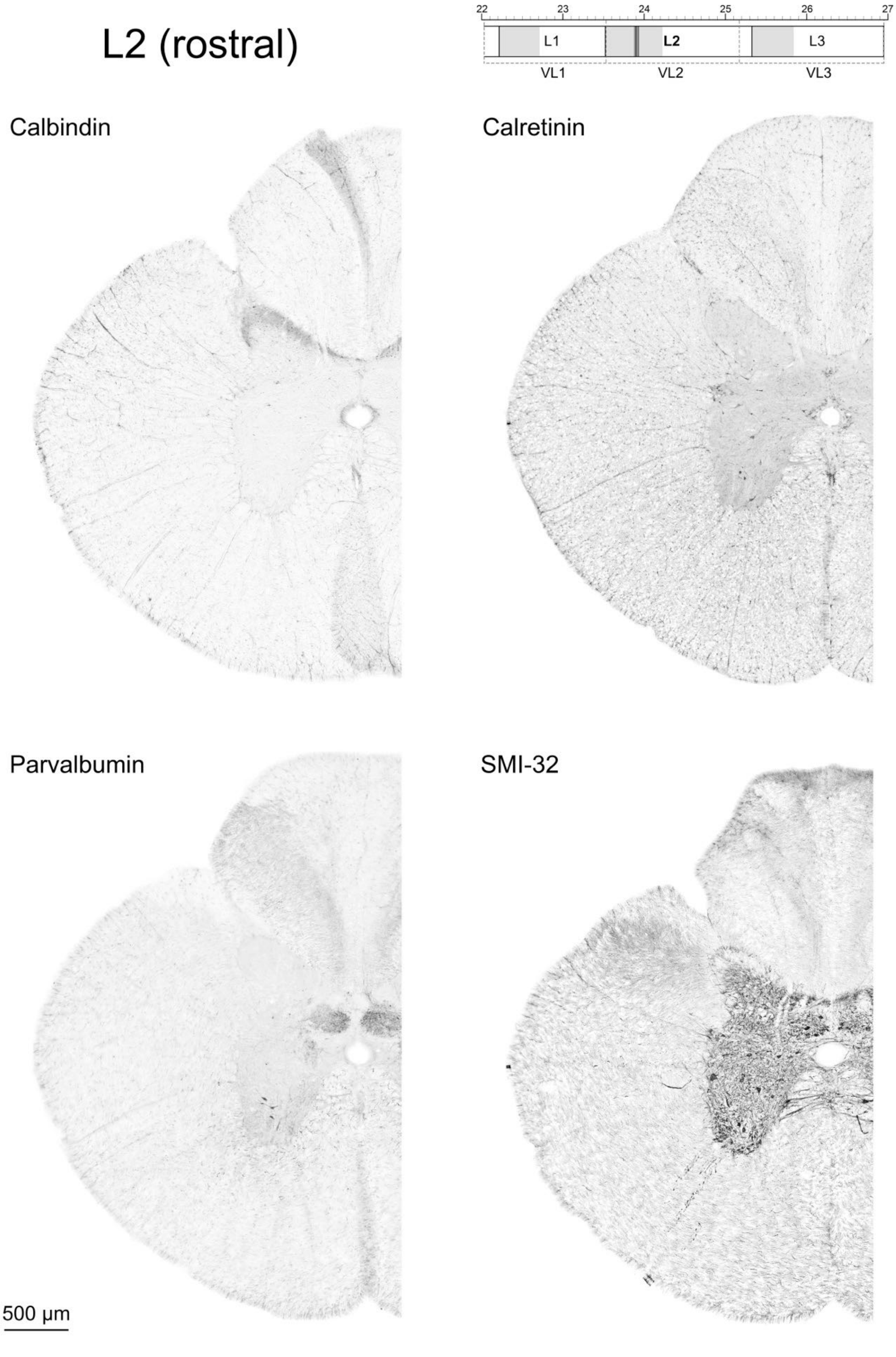

Supplementary Figure 3. Continued.

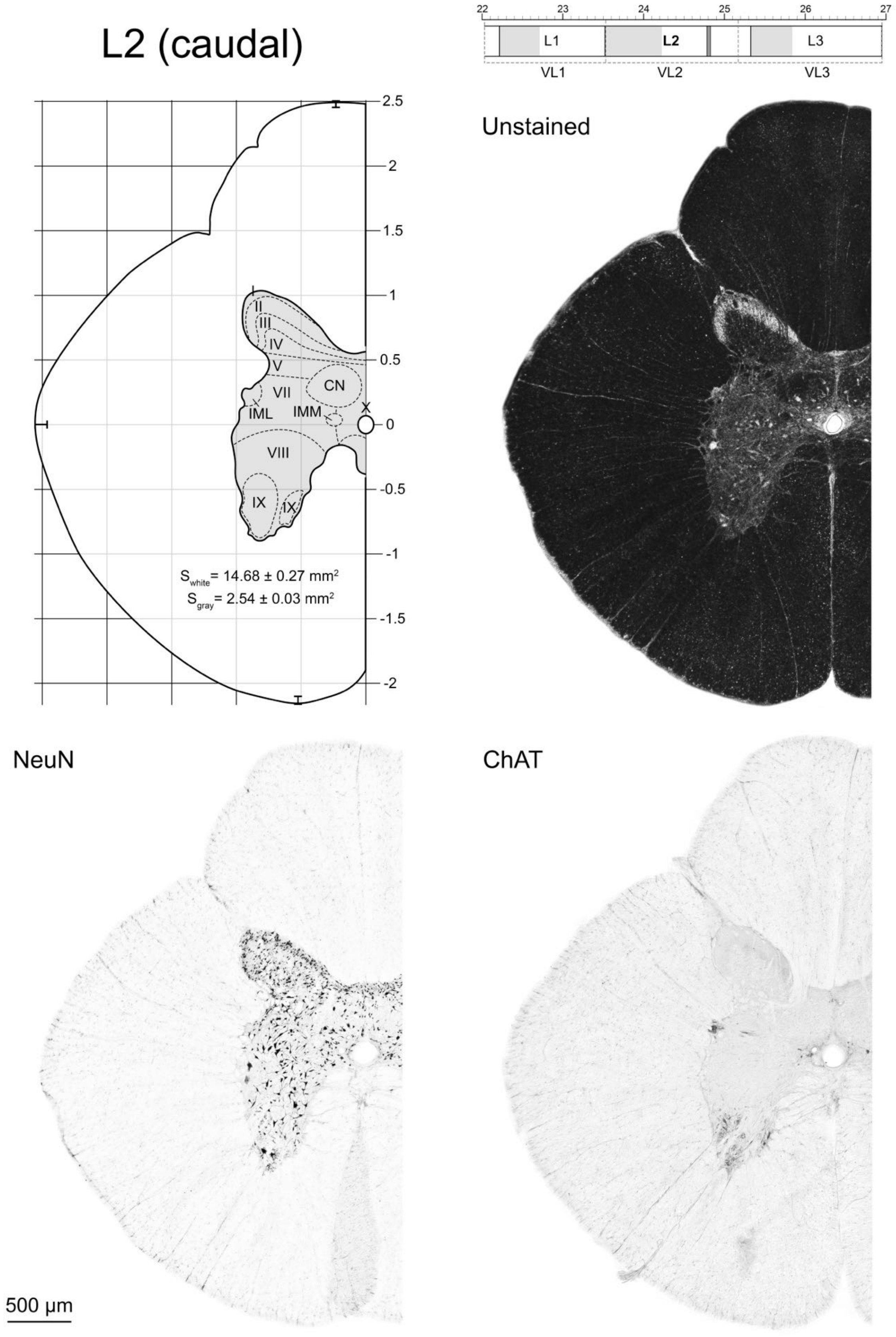

Supplementary Figure 4. Caudal part of L2 segment of the cat spinal cord.

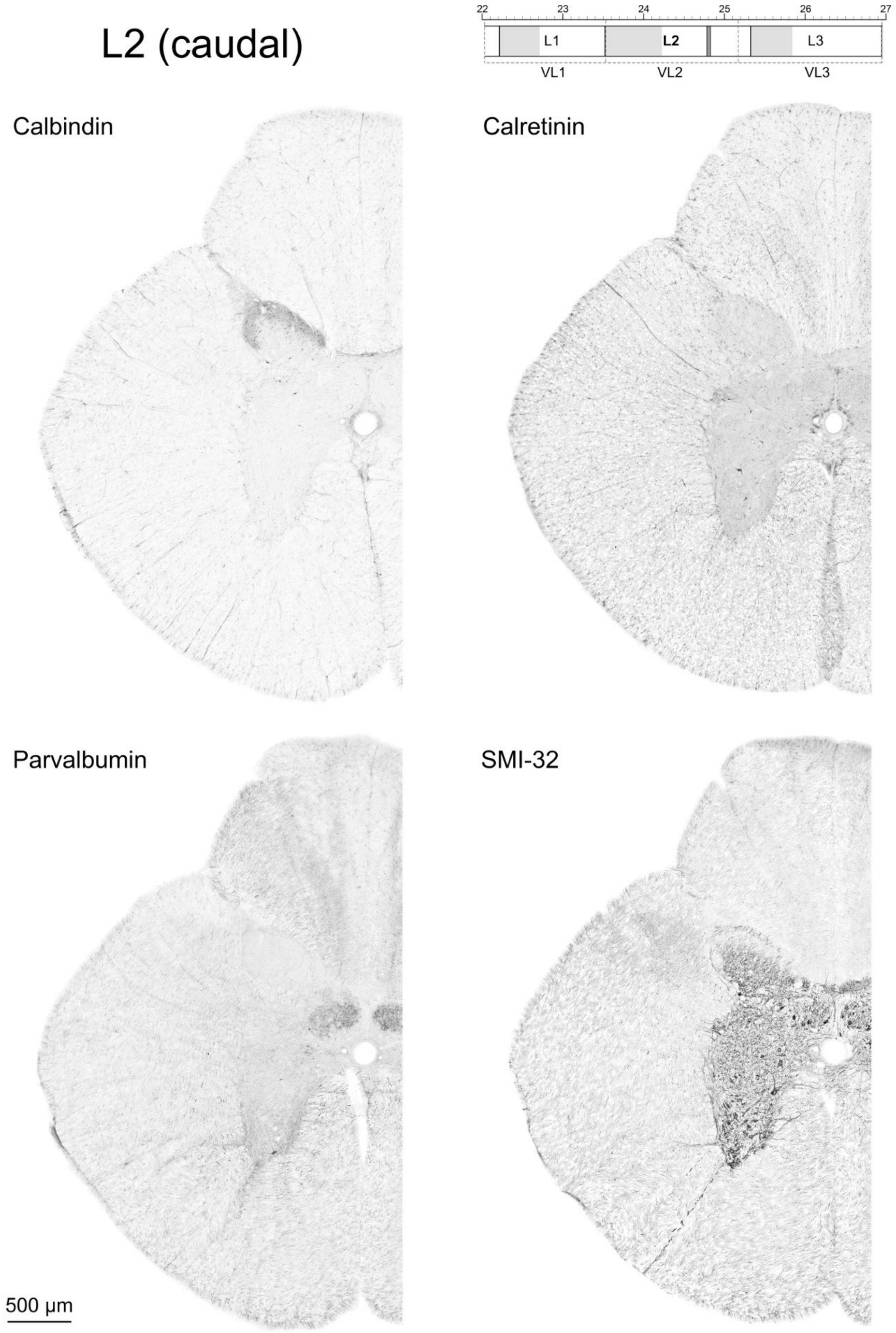

Supplementary Figure 4. Continued.

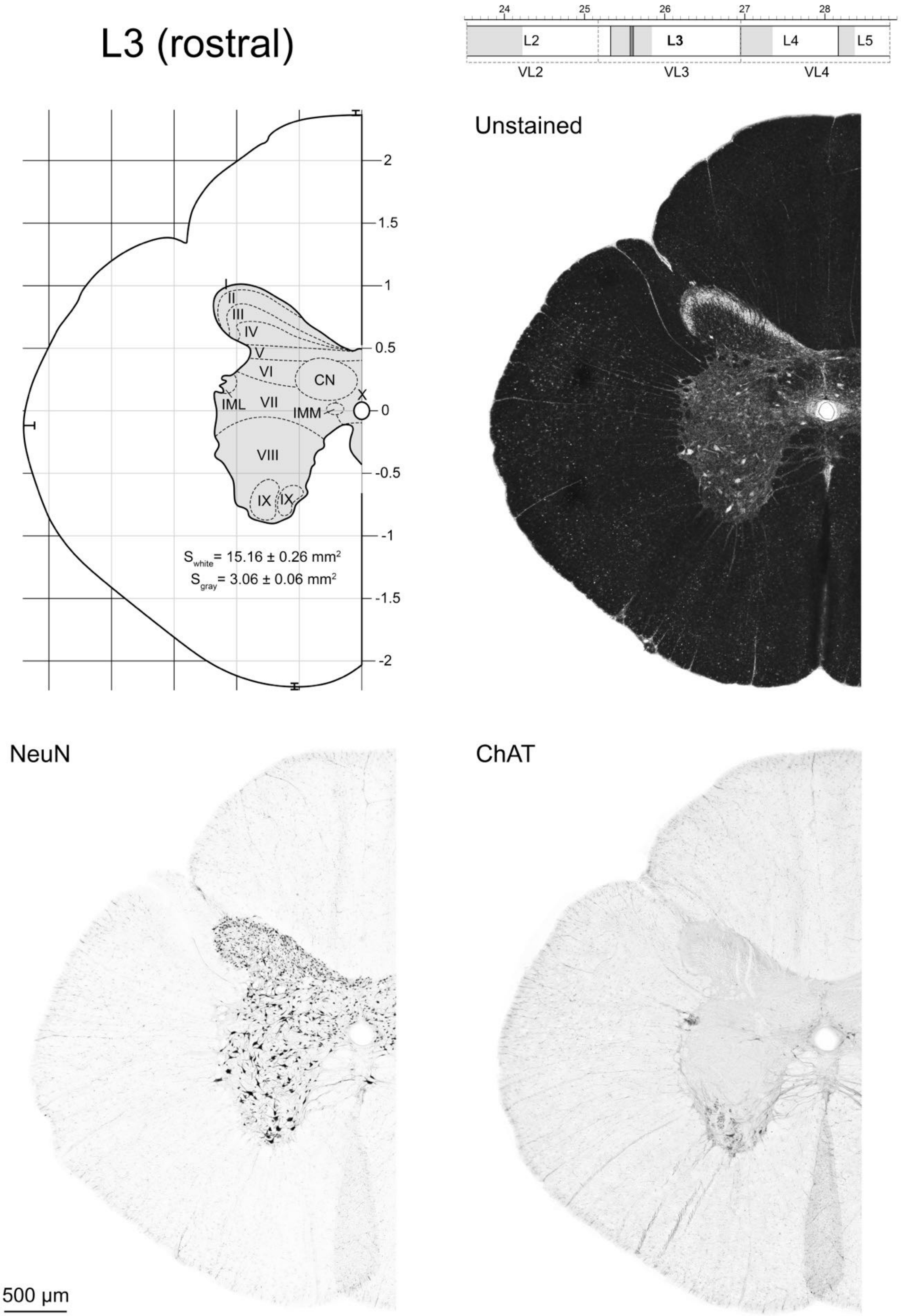

Supplementary Figure 5. Rostral part of L3 segment of the cat spinal cord.

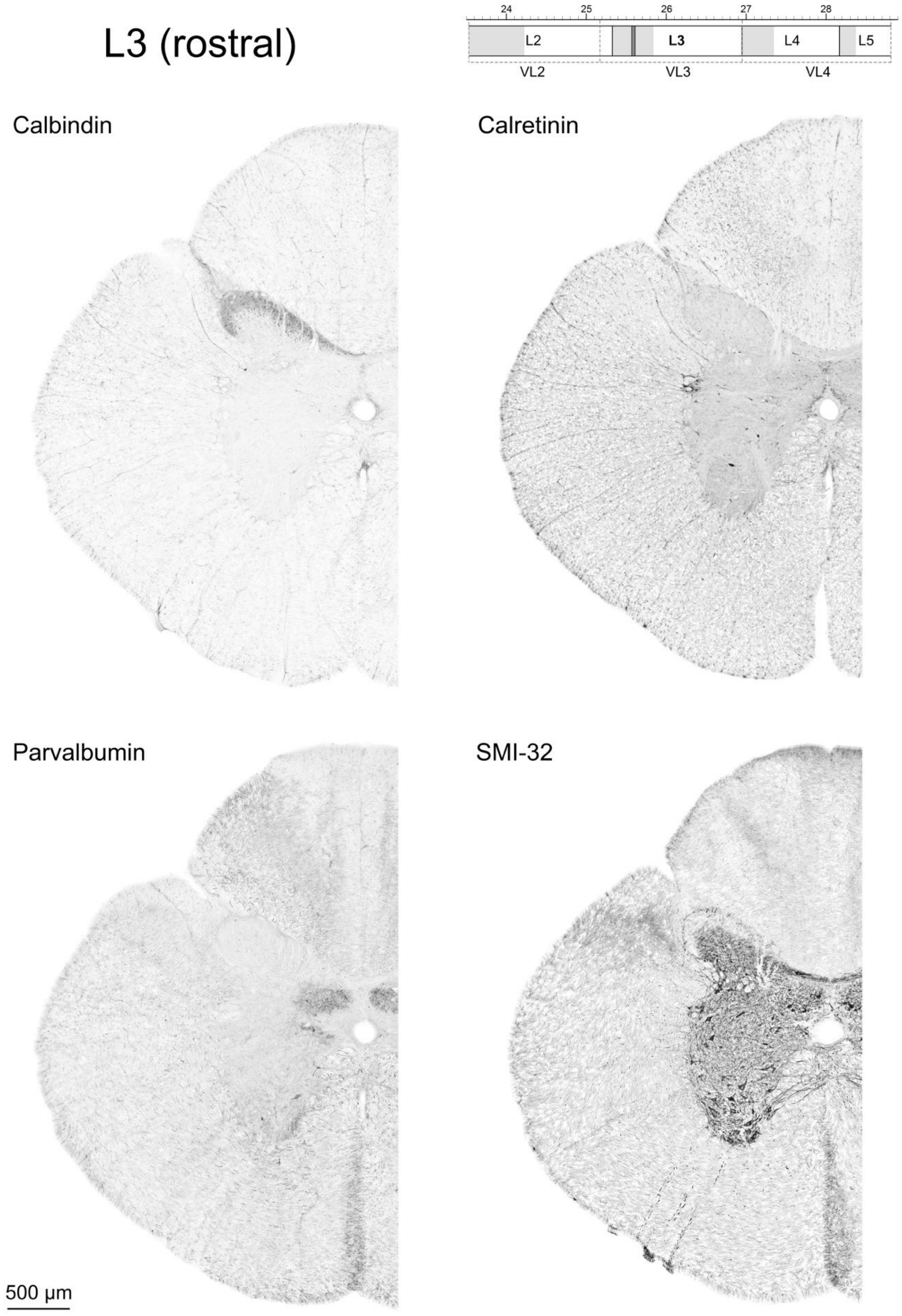

Supplementary Figure 5. Continued.

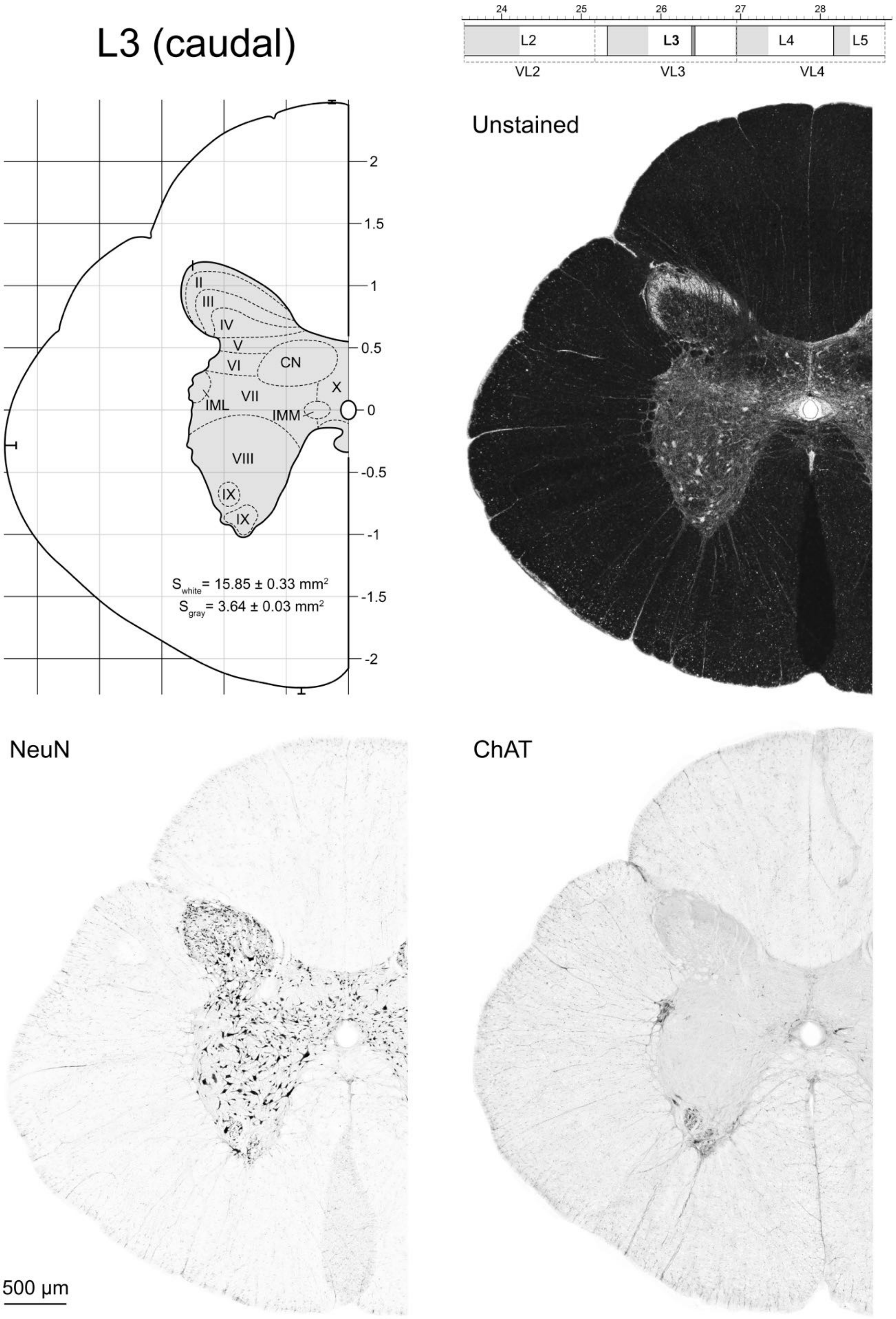

Supplementary Figure 6. Caudal part of L3 segment of the cat spinal cord.

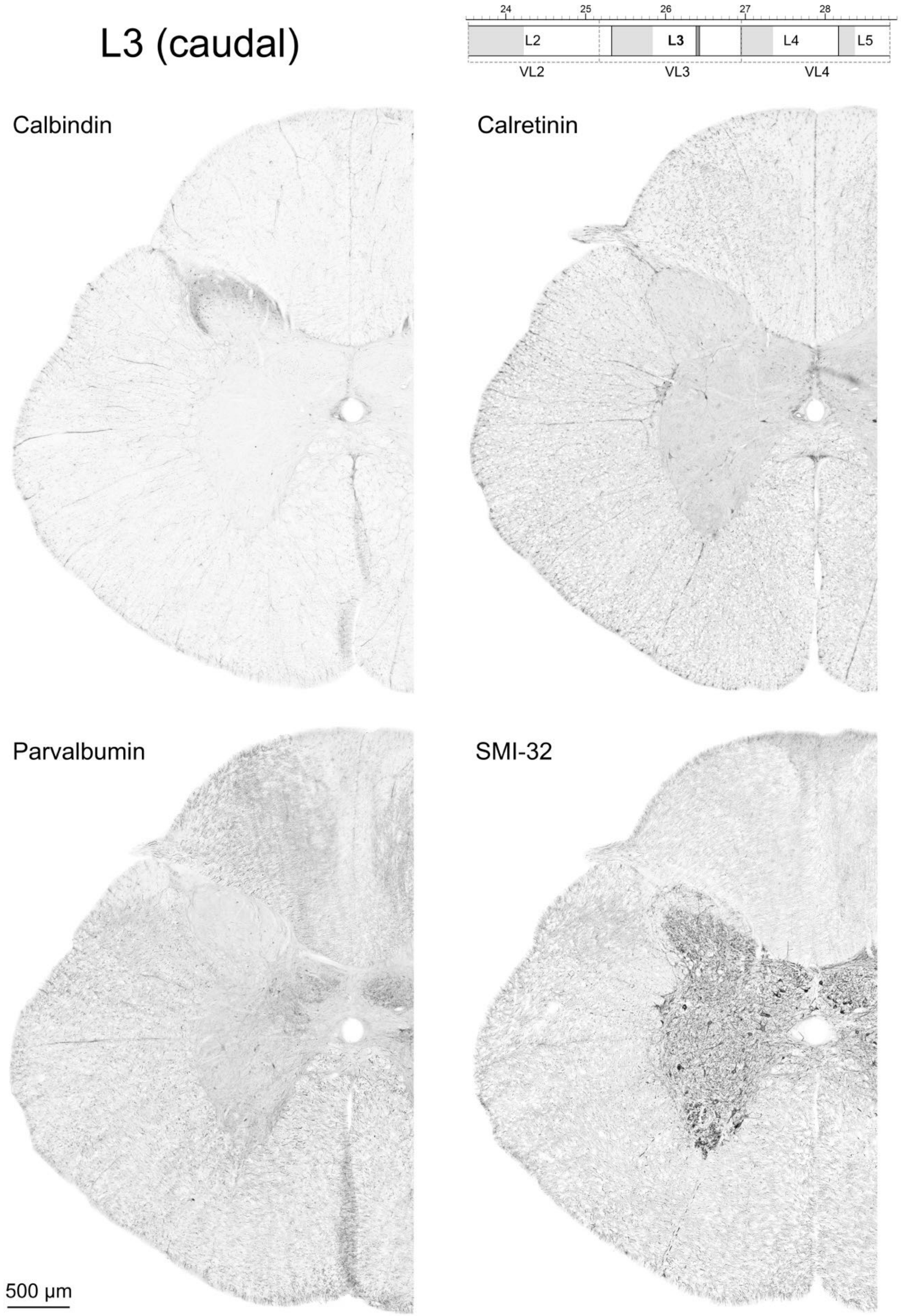

Supplementary Figure 6. Continued.

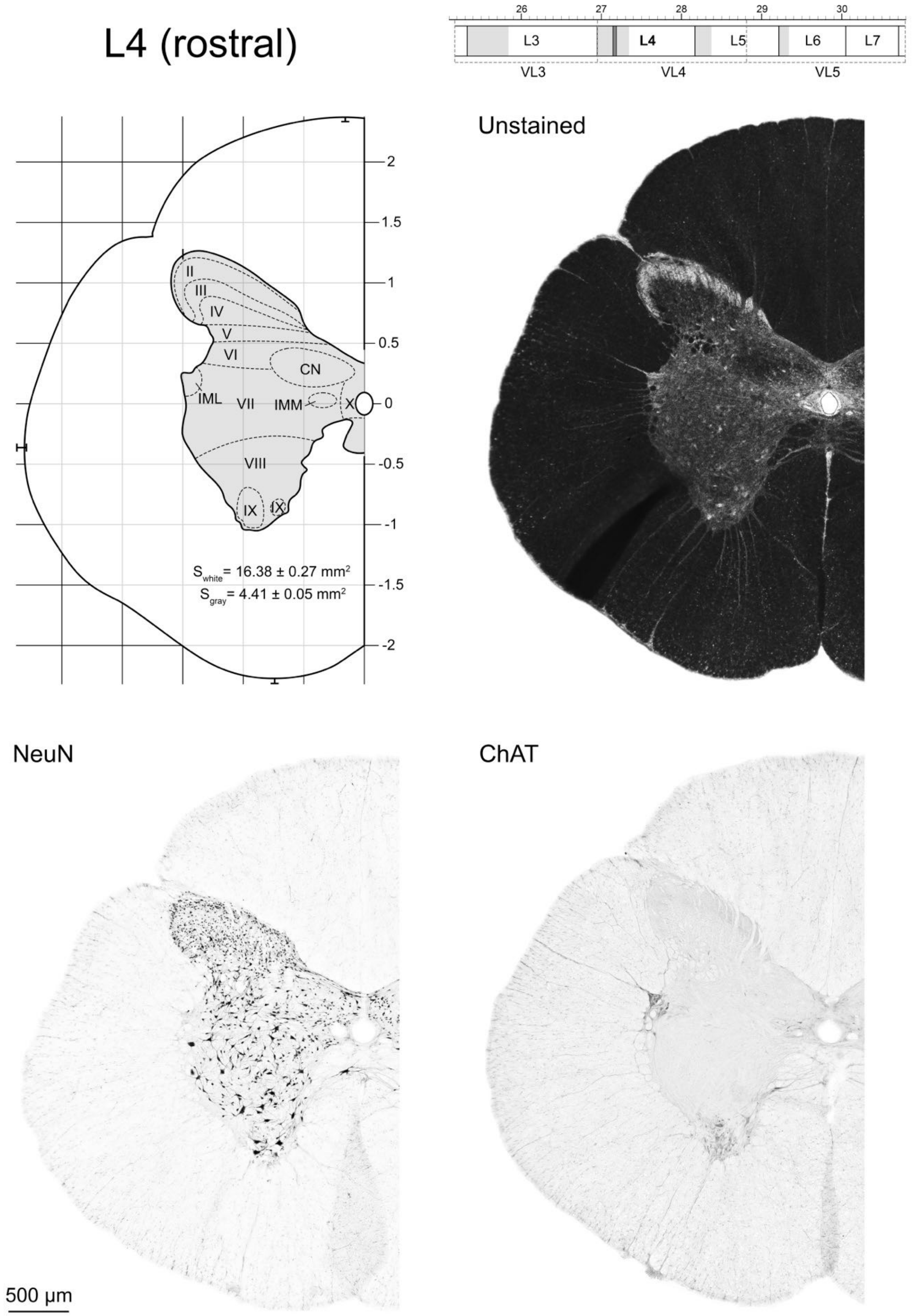

Supplementary Figure 7. Rostral part of L4 segment of the cat spinal cord.

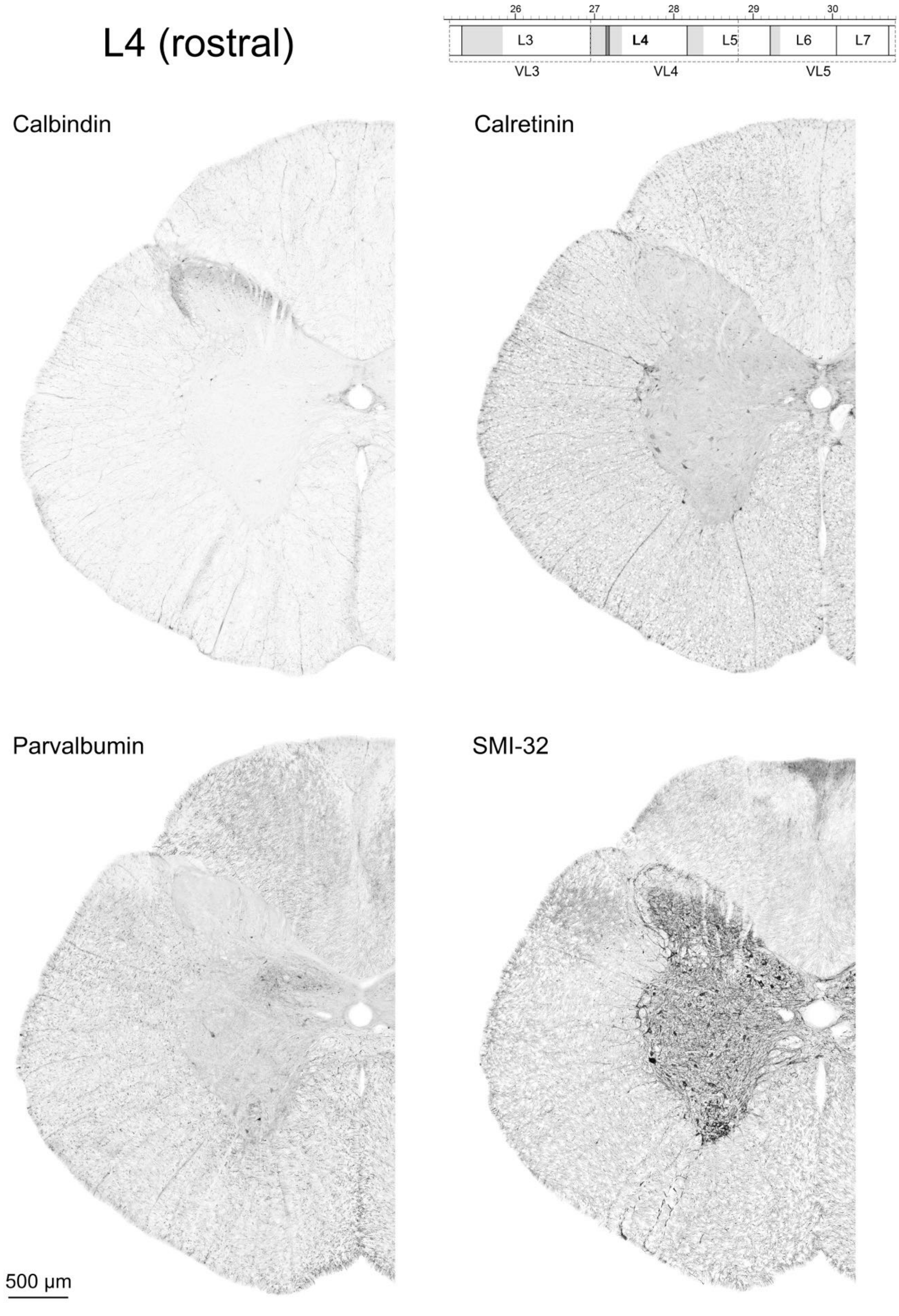

Supplementary Figure 7. Continued.

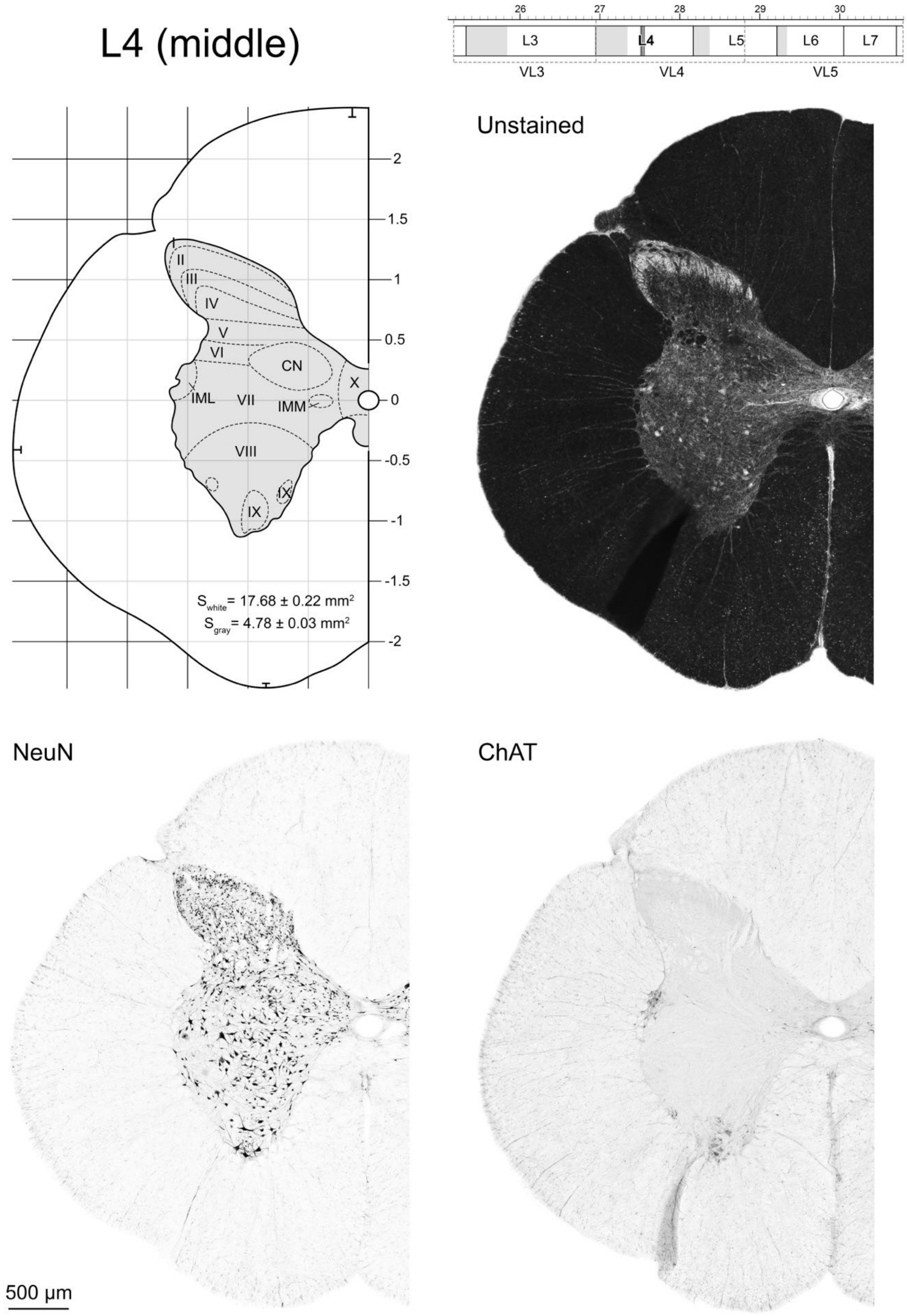

Supplementary Figure 8. Middle part of L4 segment of the cat spinal cord.

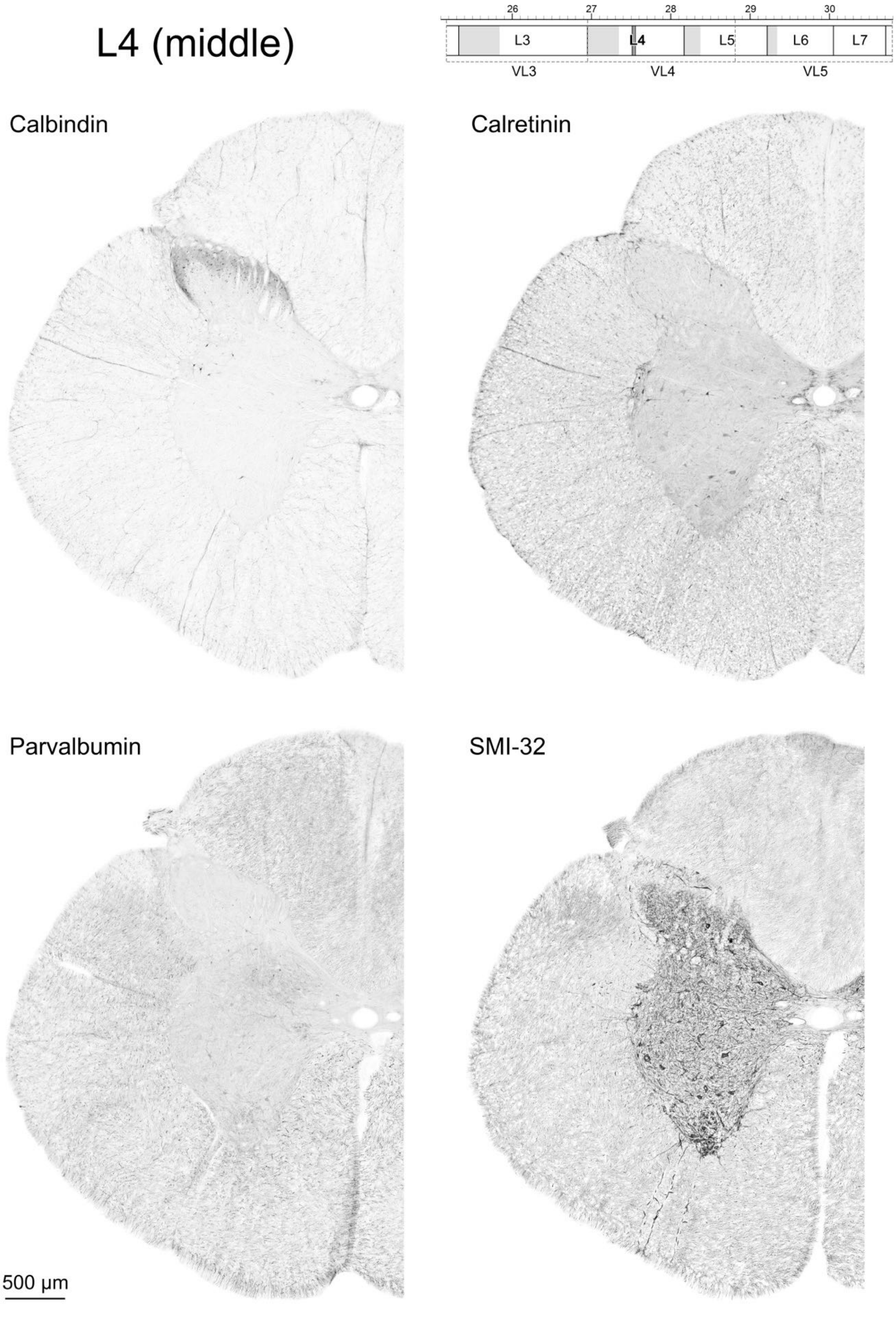

Supplementary Figure 8. Continued.

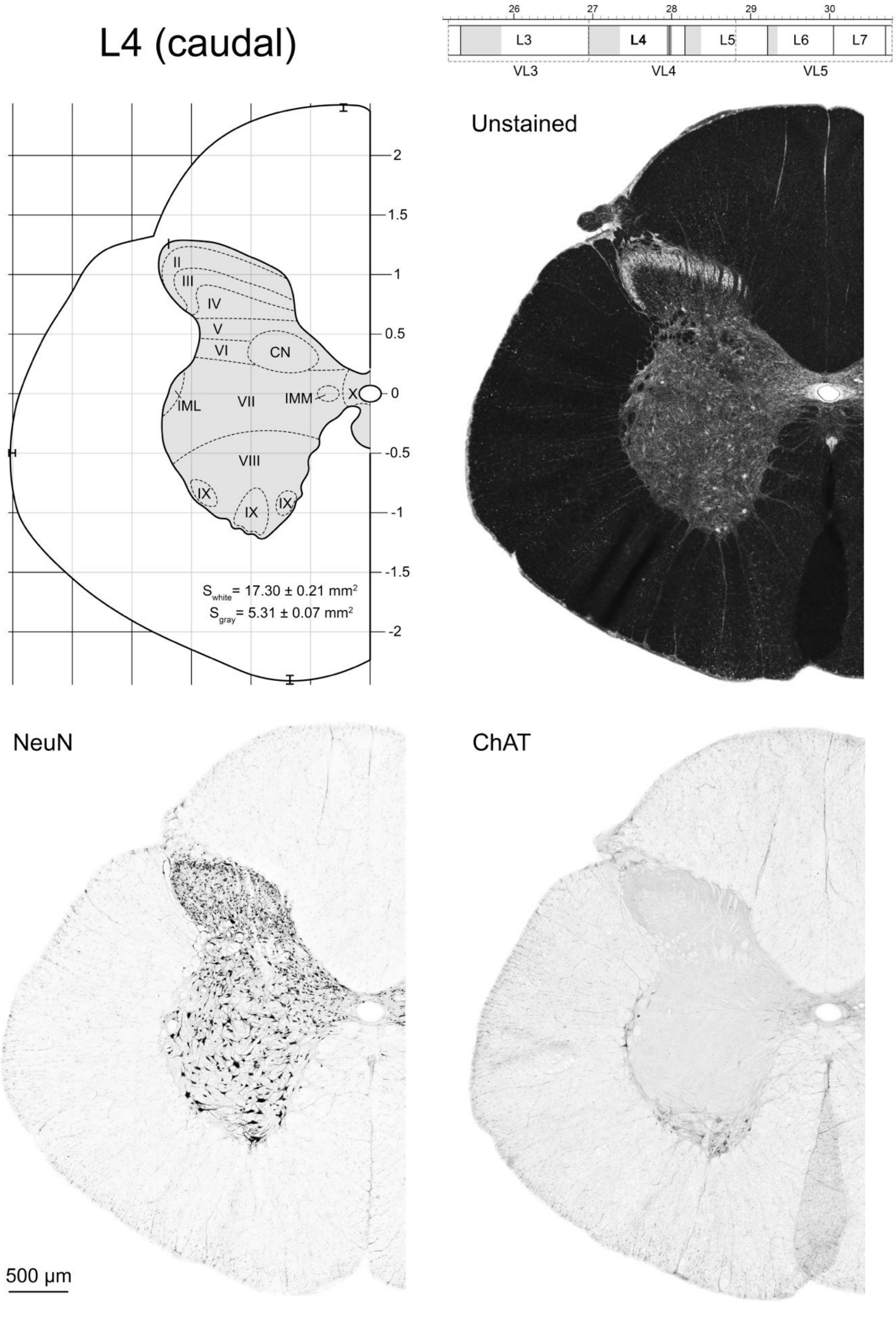

Supplementary Figure 9. Caudal part of L4 segment of the cat spinal cord.

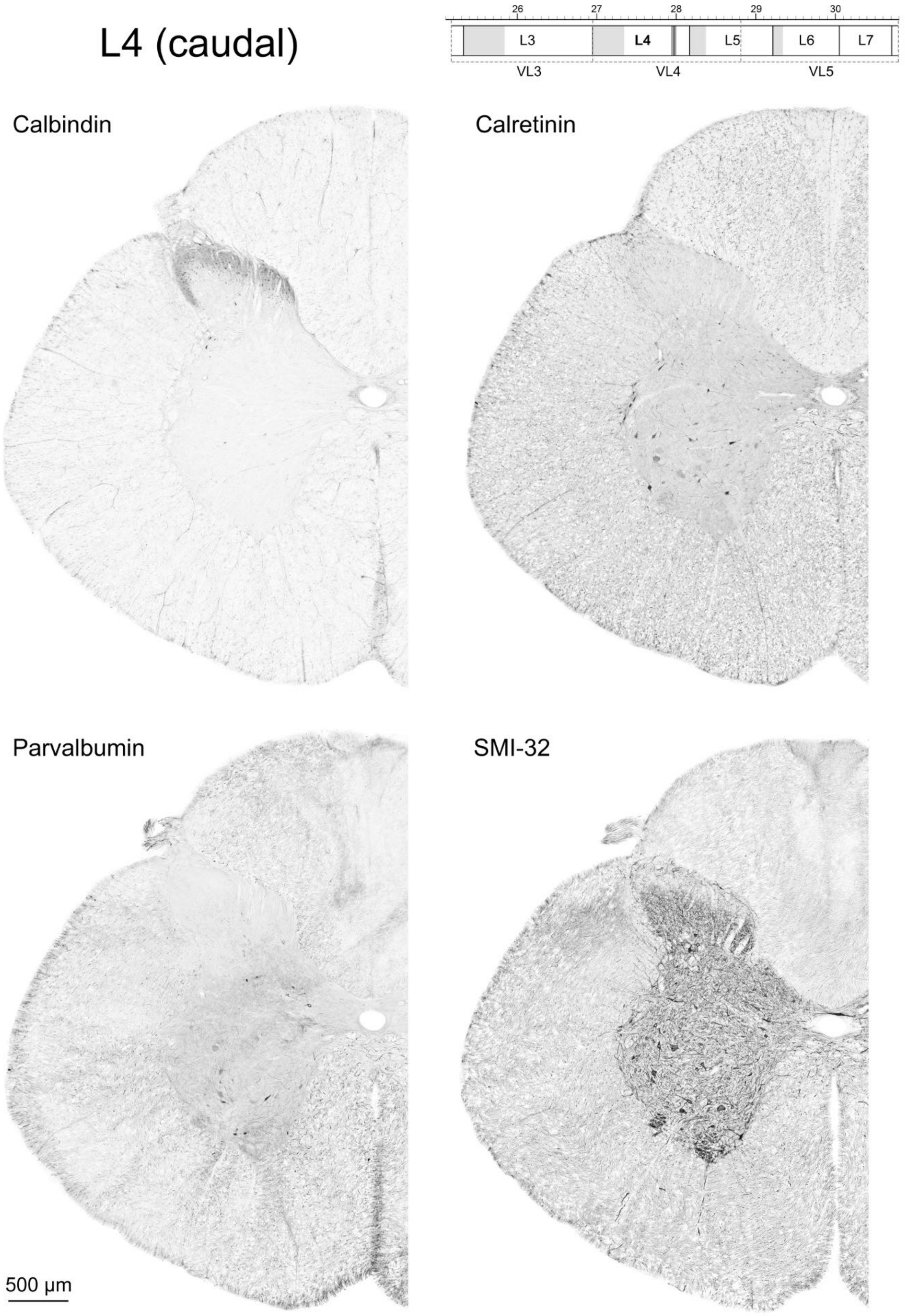

Supplementary Figure 9. Continued.

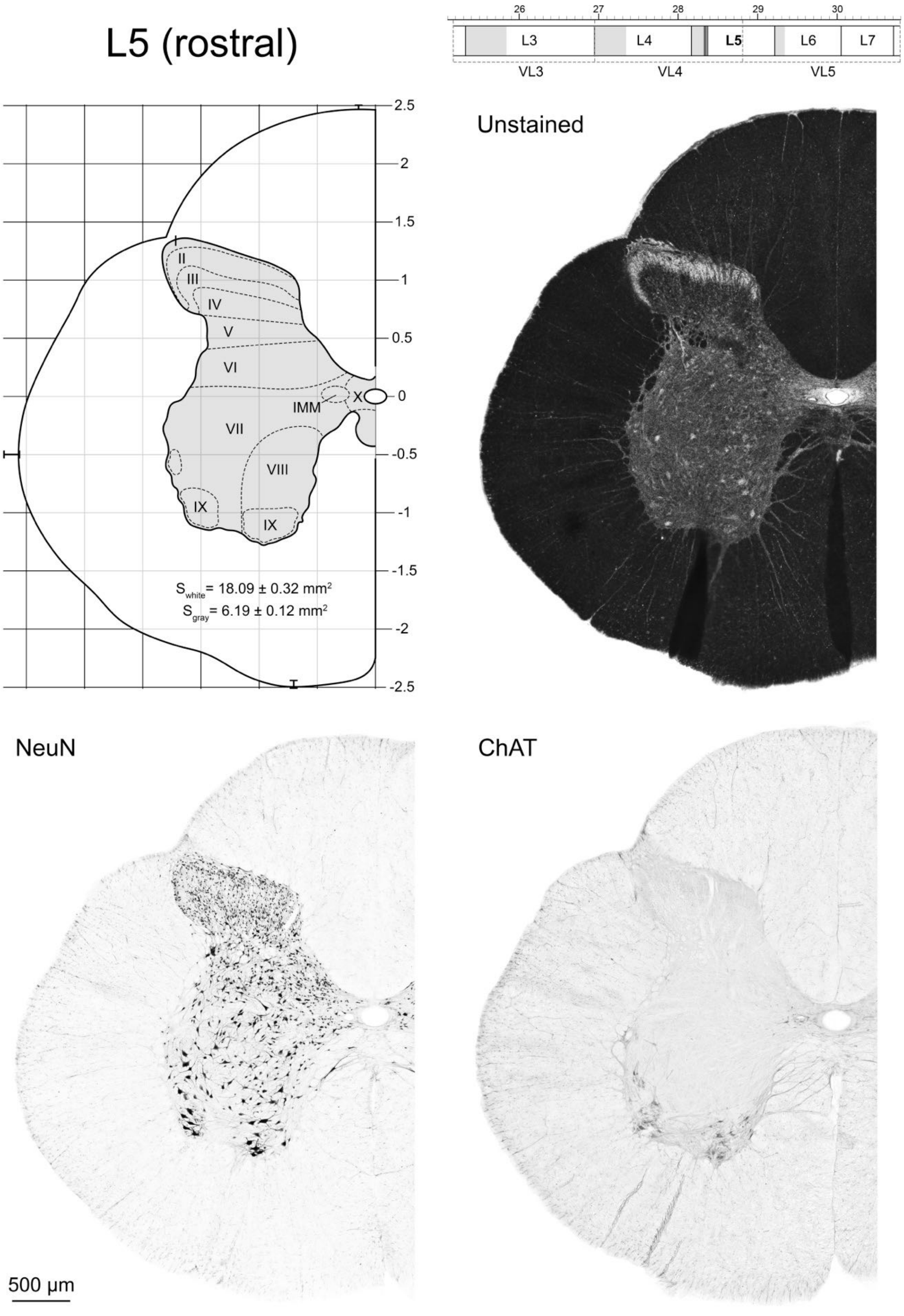

Supplementary Figure 10. Rostral part of L5 segment of the cat spinal cord.

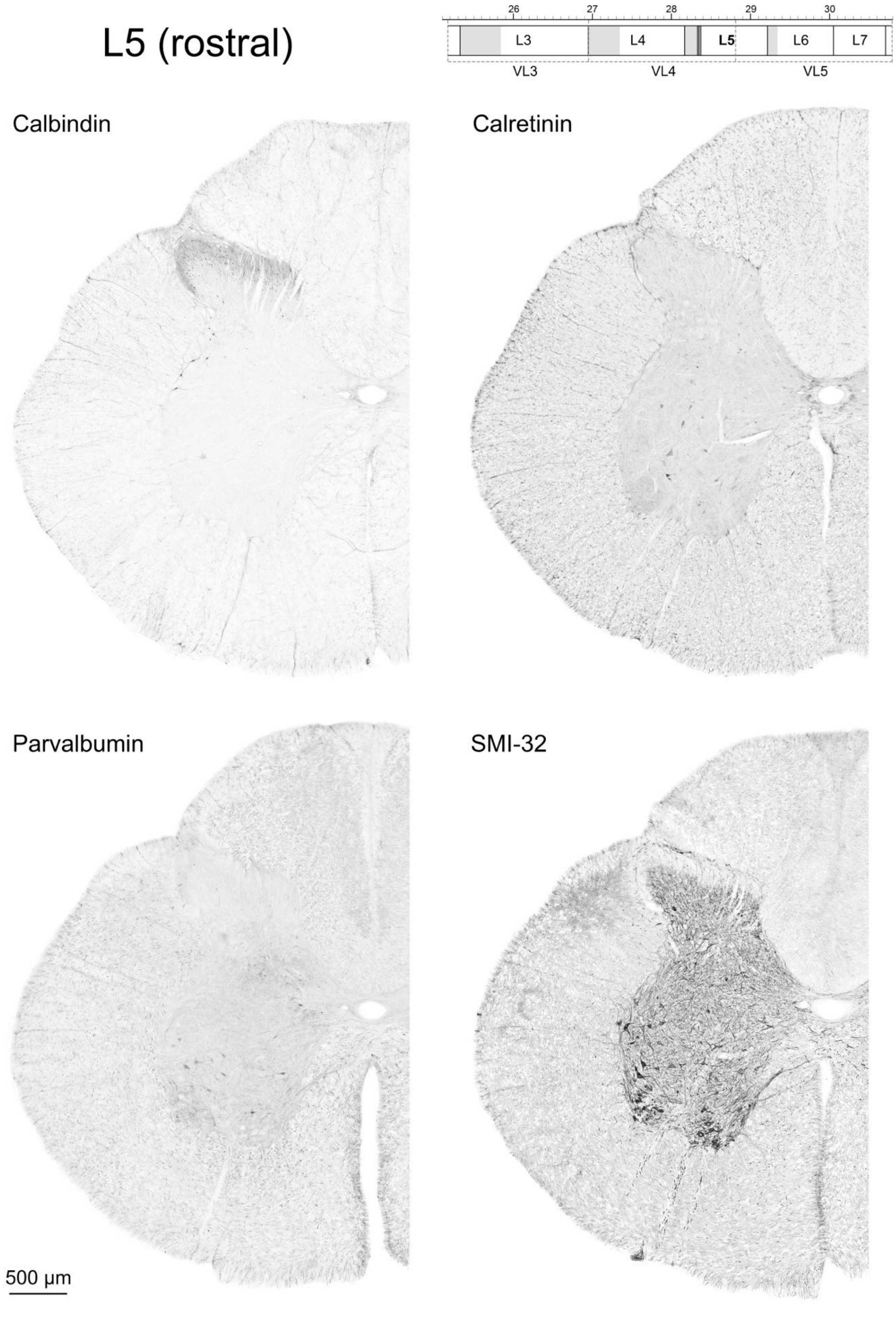

Supplementary Figure 10. Continued.

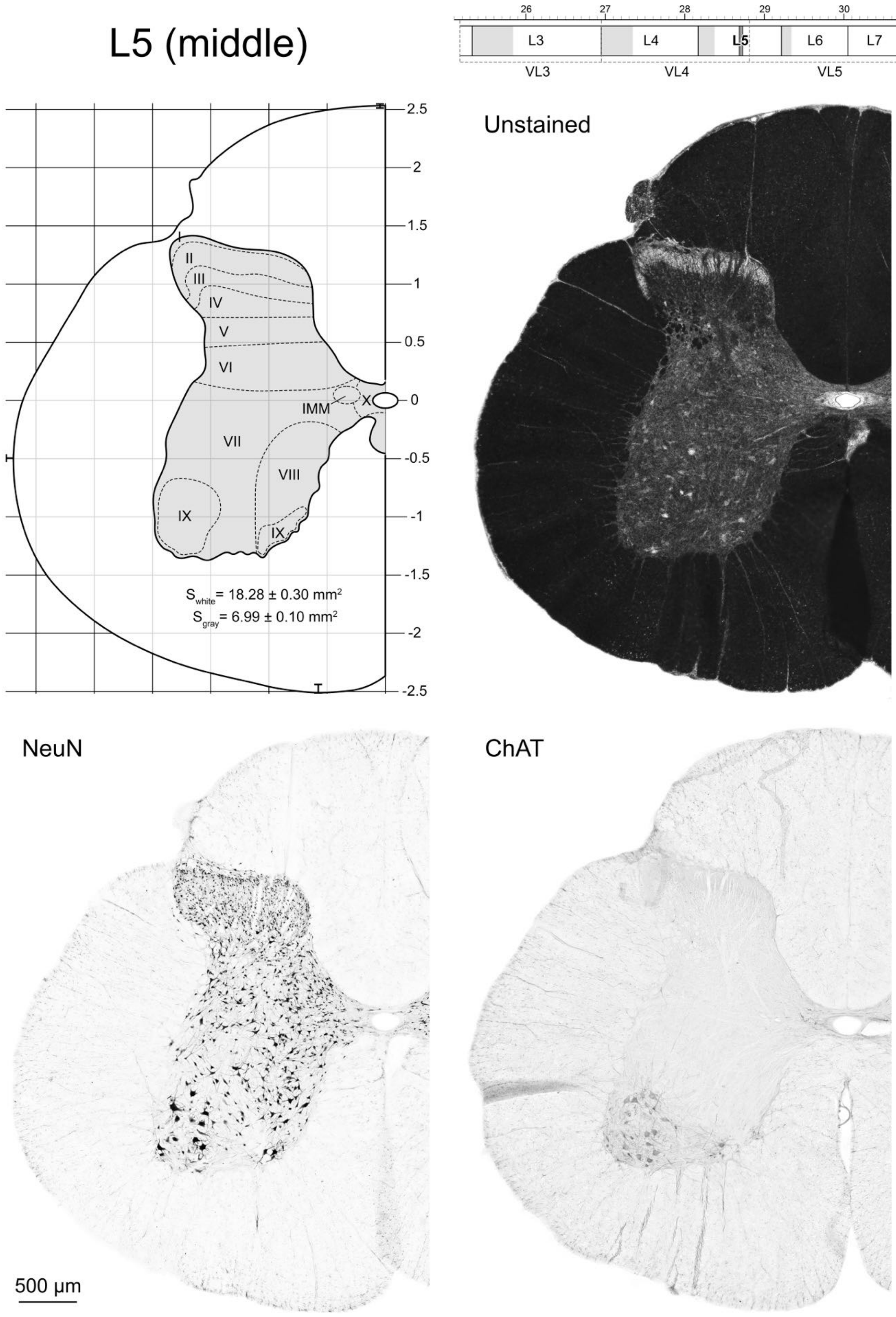

Supplementary Figure 11. Middle part of L5 segment of the cat spinal cord.

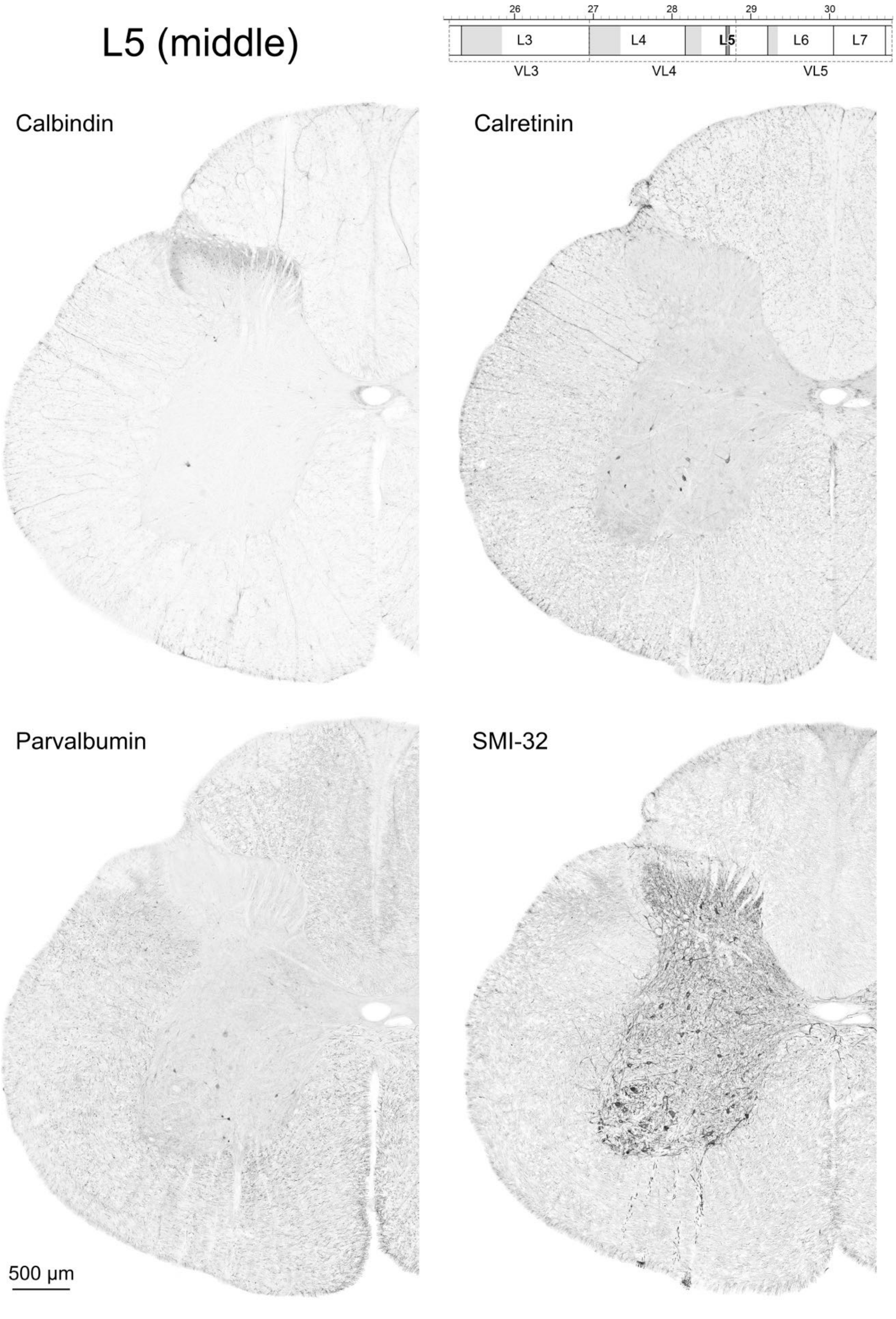

Supplementary Figure 11. Continued.

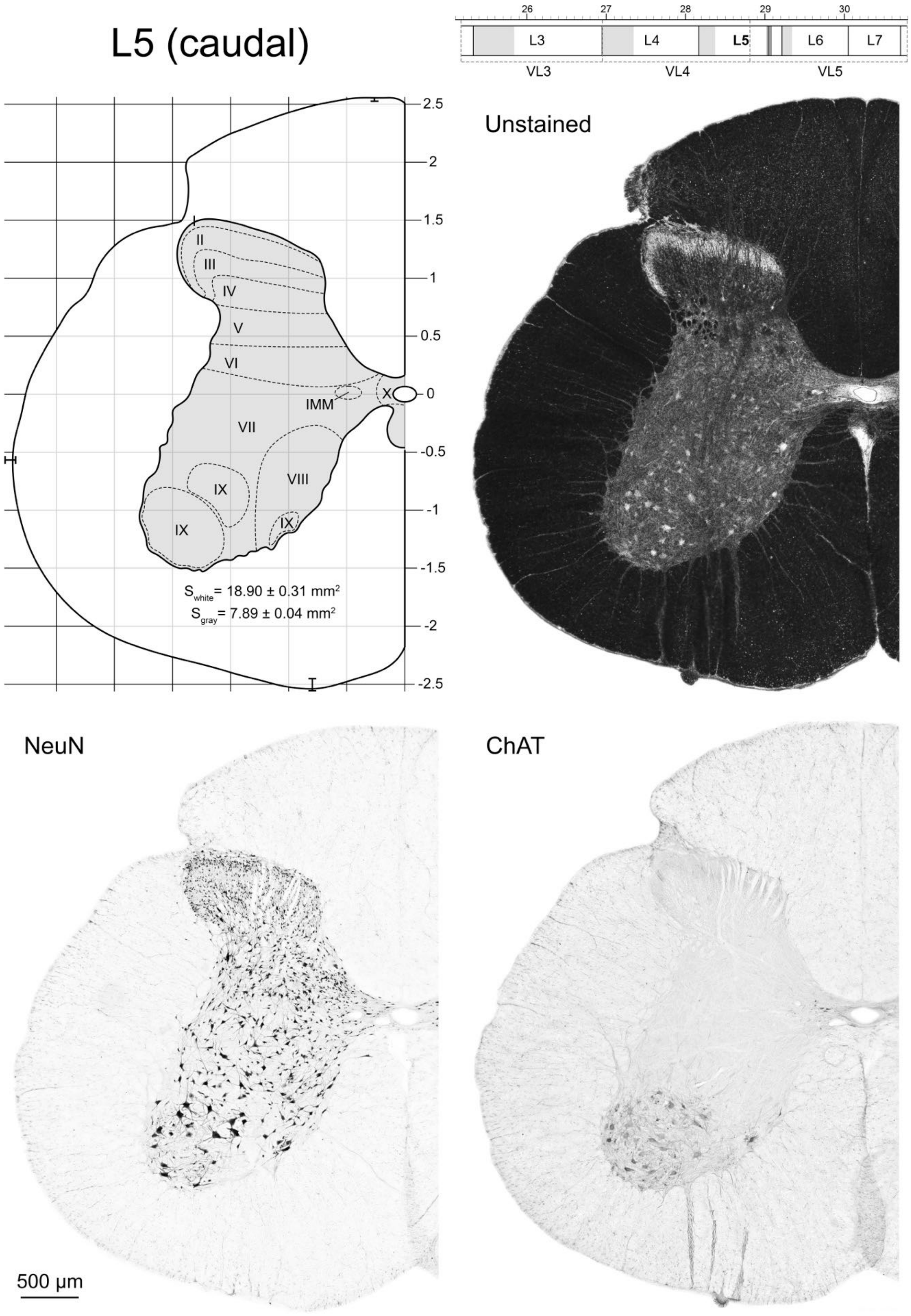

Supplementary Figure 12. Caudal part of L5 segment of the cat spinal cord.

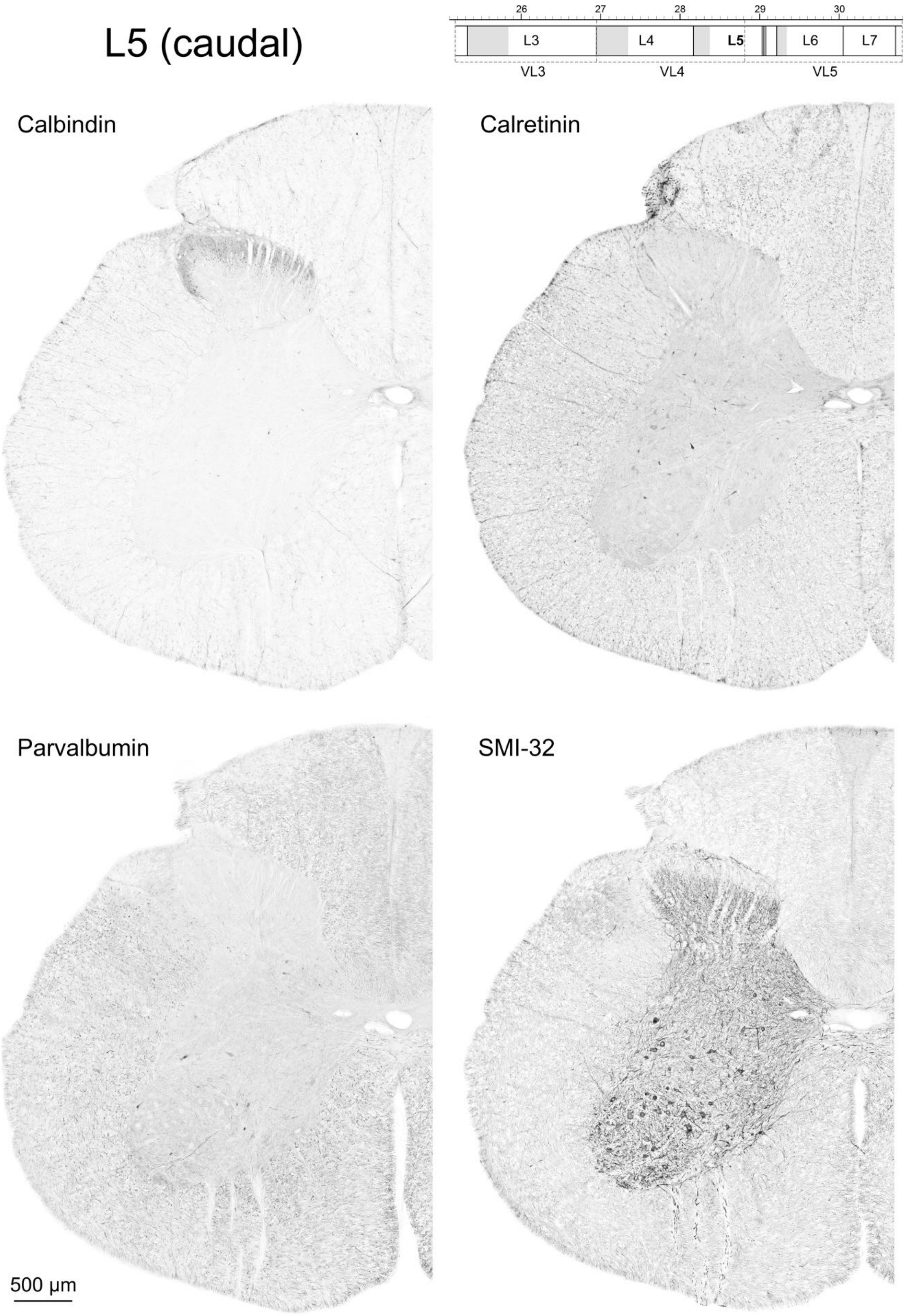

Supplementary Figure 12. Continued.

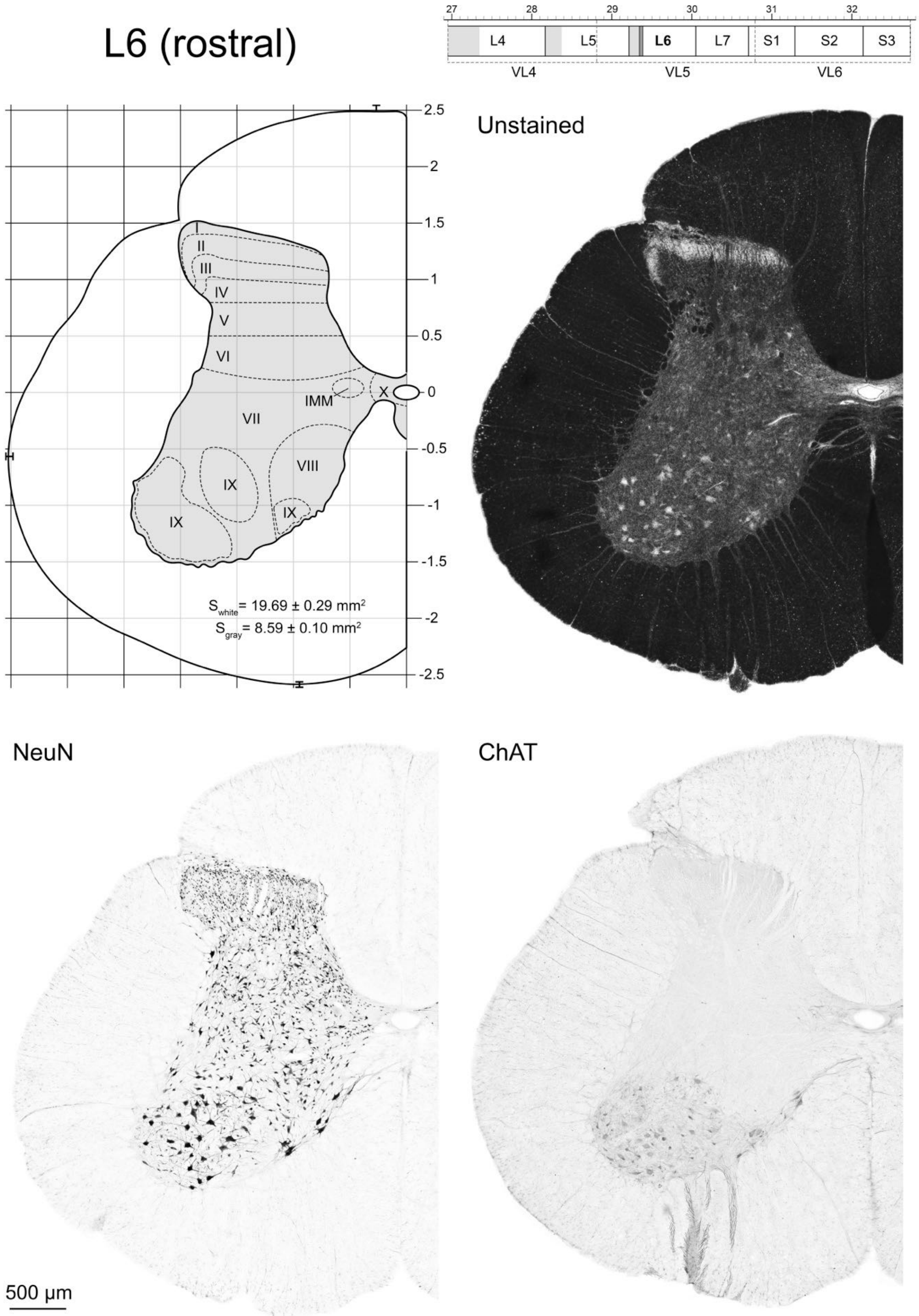

Supplementary Figure 13. Rostral part of L6 segment of the cat spinal cord.

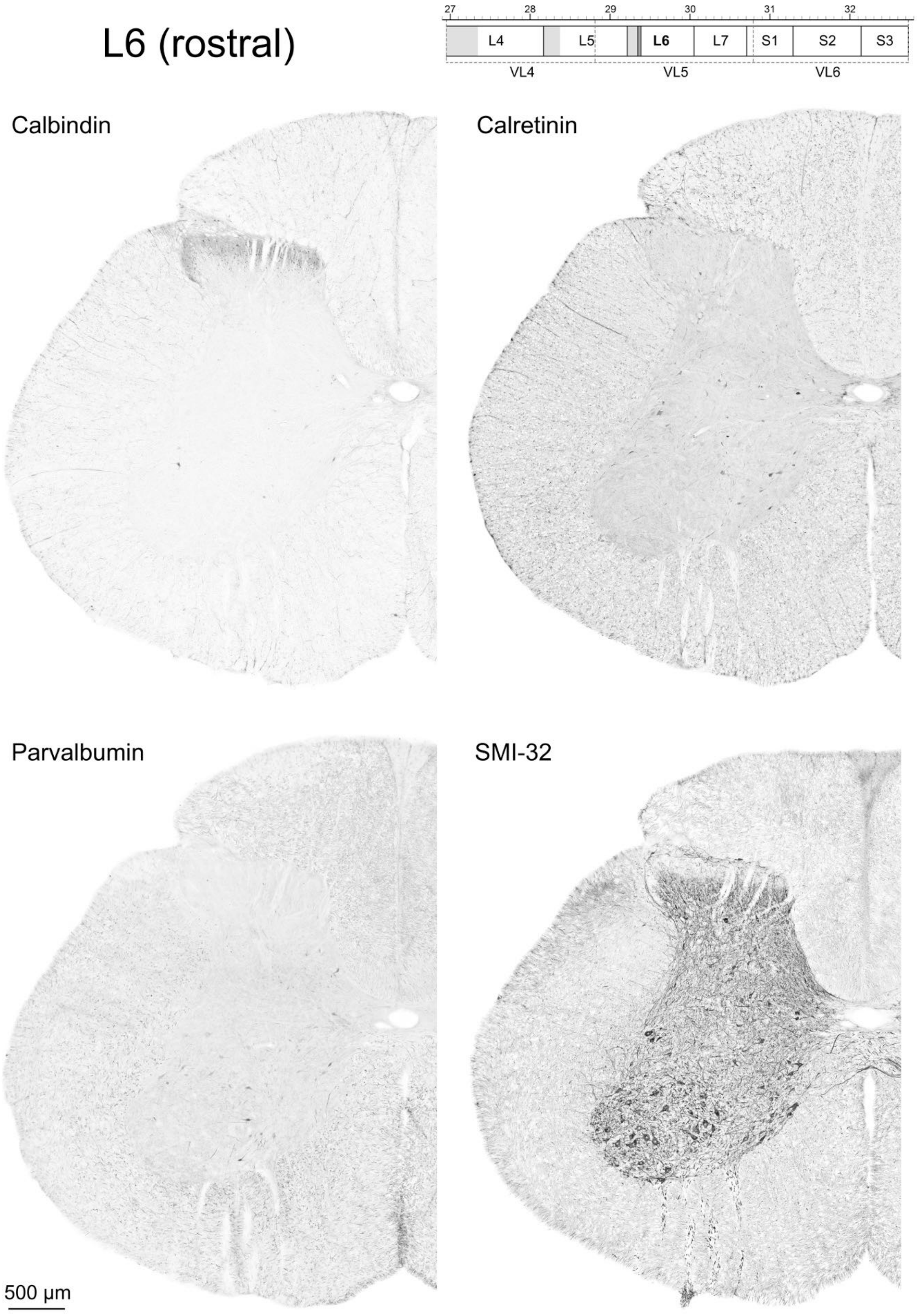

Supplementary Figure 13. Continued.

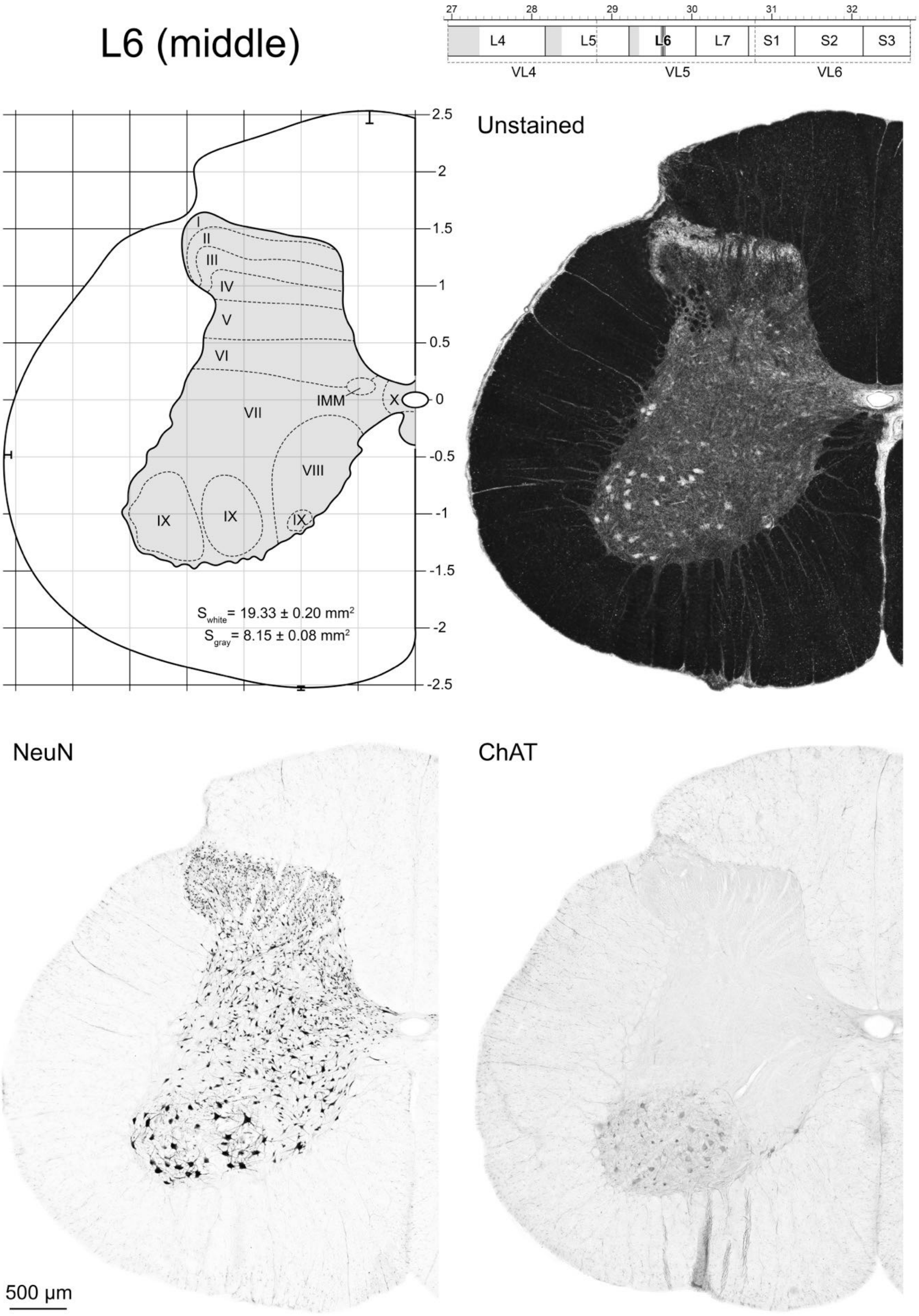

Supplementary Figure 14. Middle part of L6 segment of the cat spinal cord.

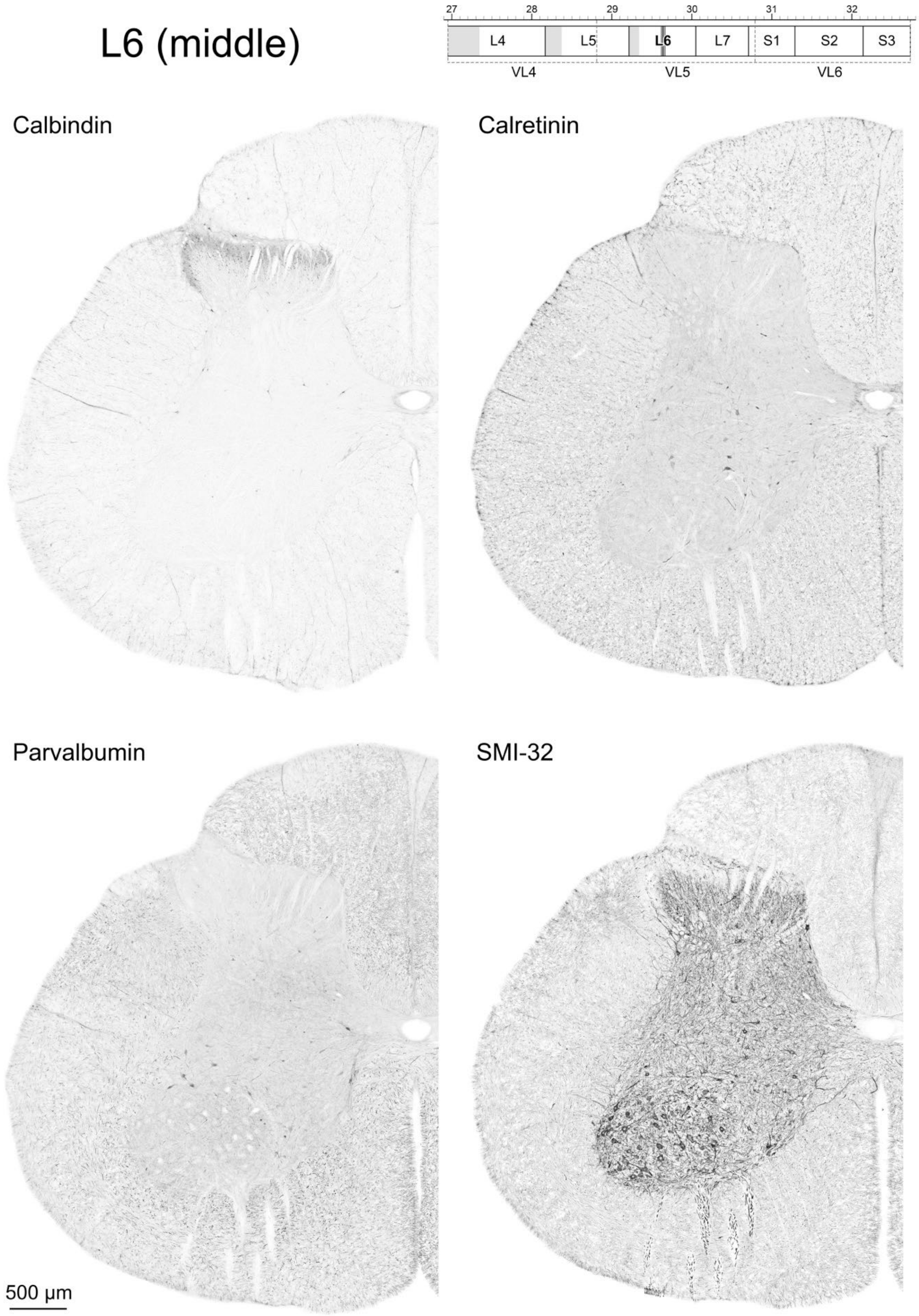

Supplementary Figure 14. Continued.

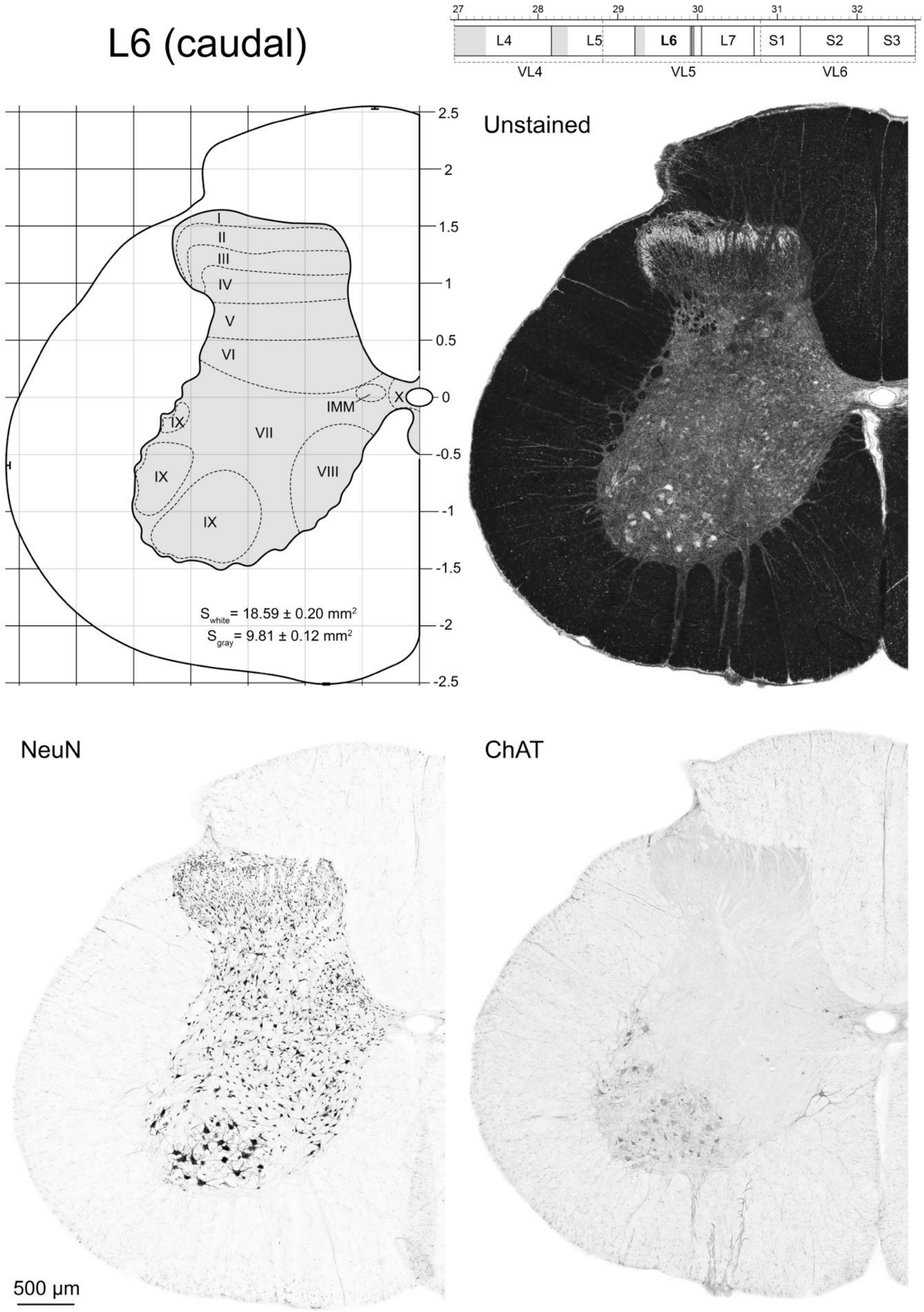

Supplementary Figure 15. Caudal part of L6 segment of the cat spinal cord.

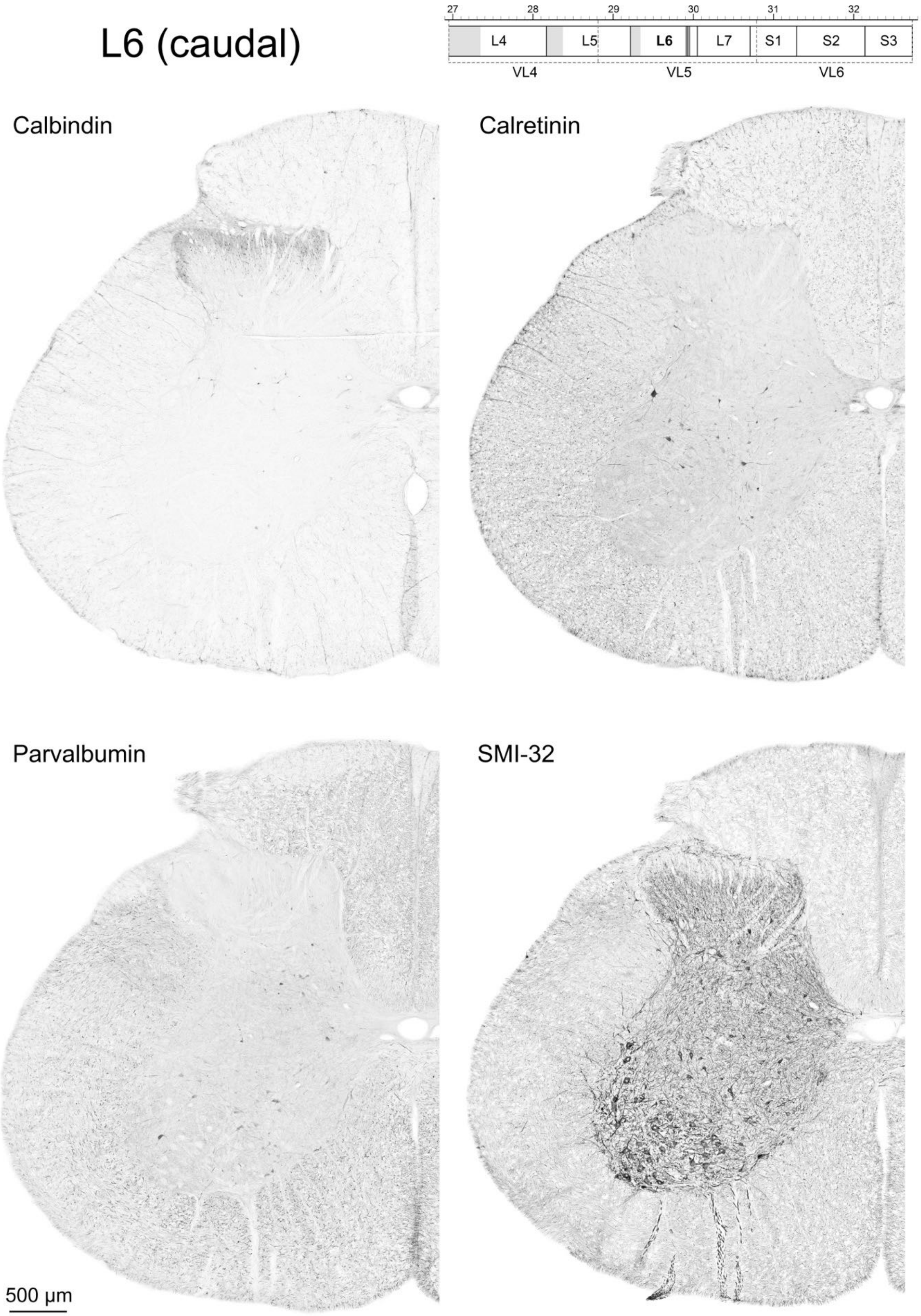

Supplementary Figure 15. Continued.

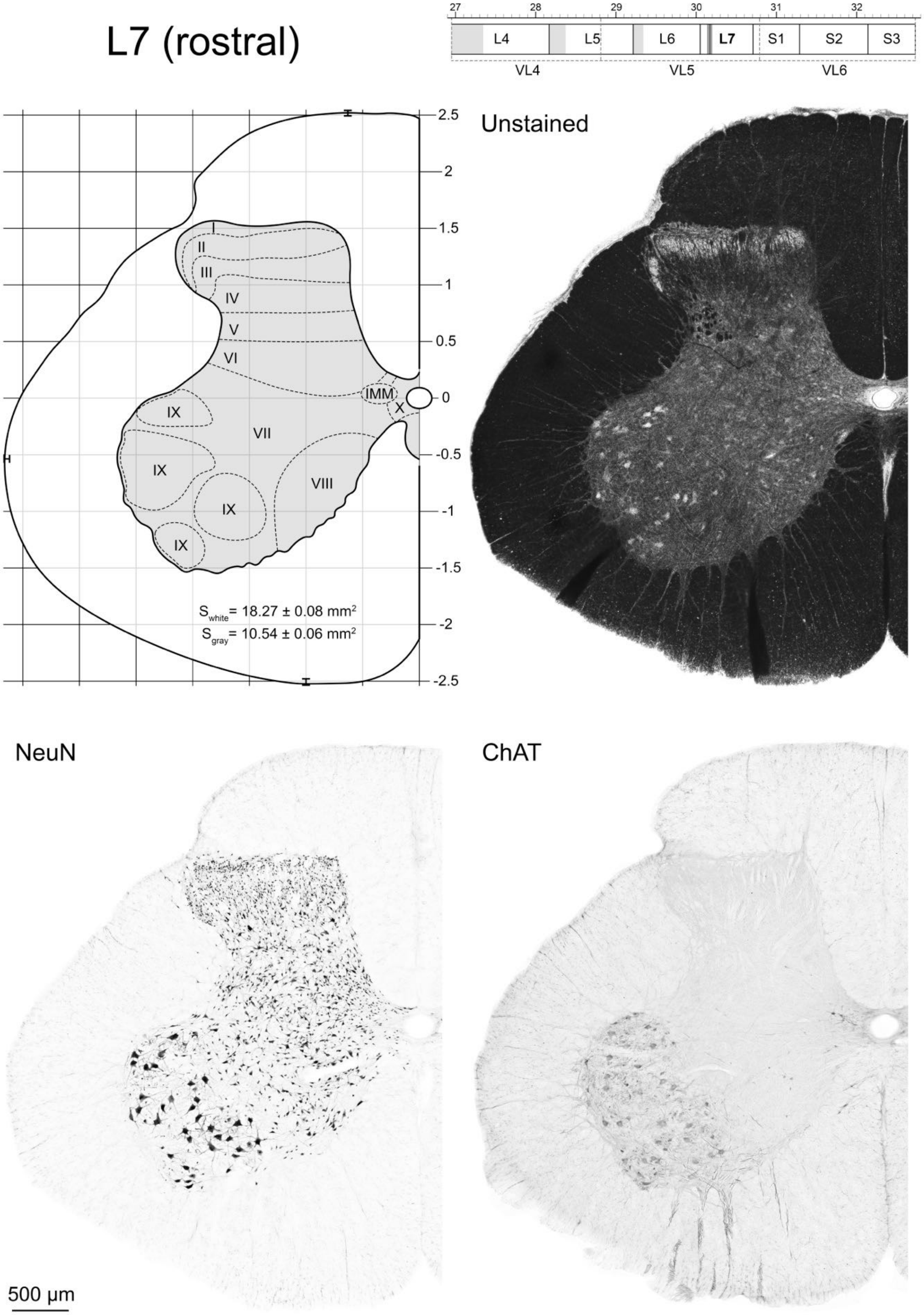

Supplementary Figure 16. Rostral part of L7 segment of the cat spinal cord.

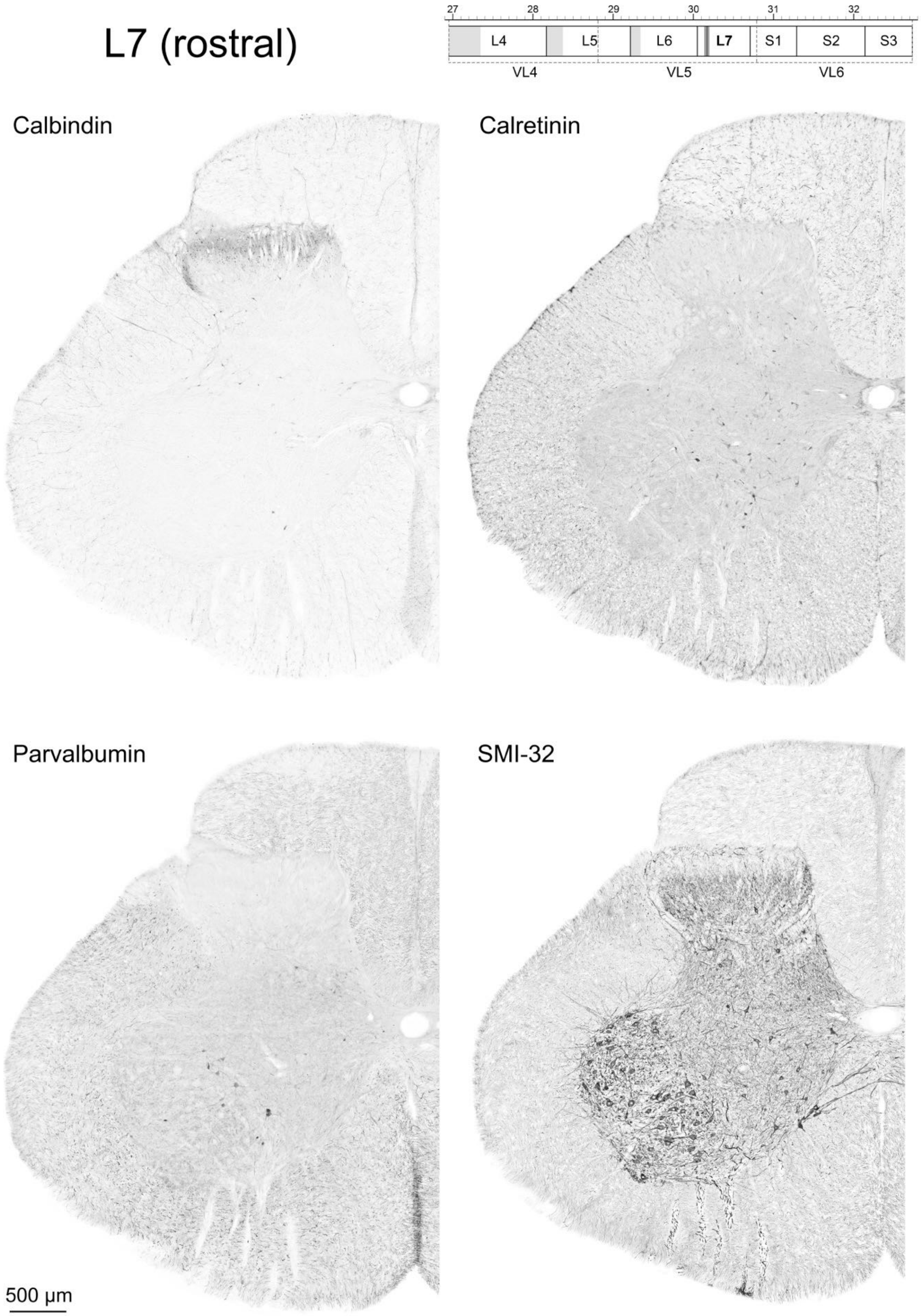

Supplementary Figure 16. Continued.

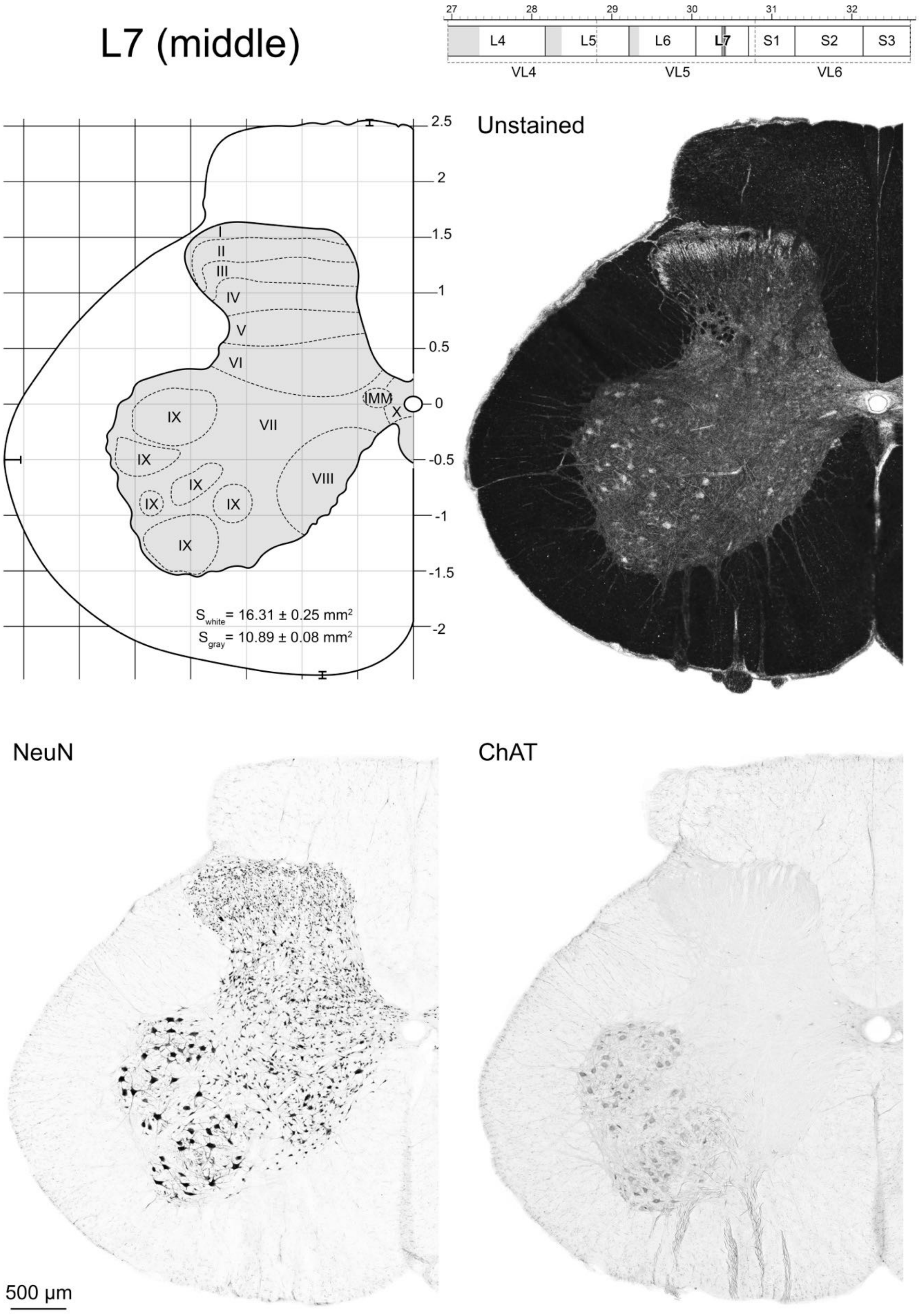

Supplementary Figure 17. Middle part of L7 segment of the cat spinal cord.

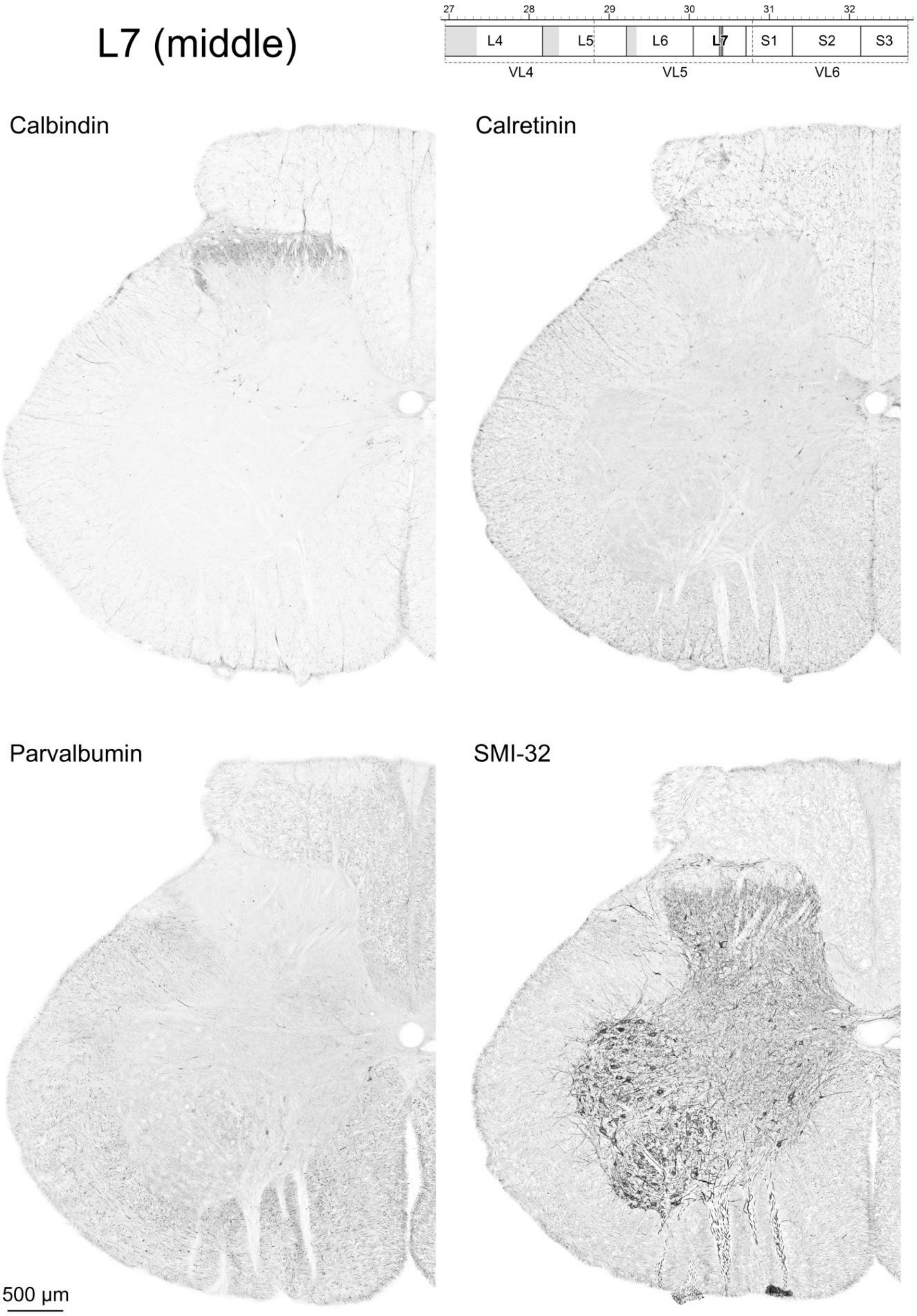

Supplementary Figure 17. Continued.

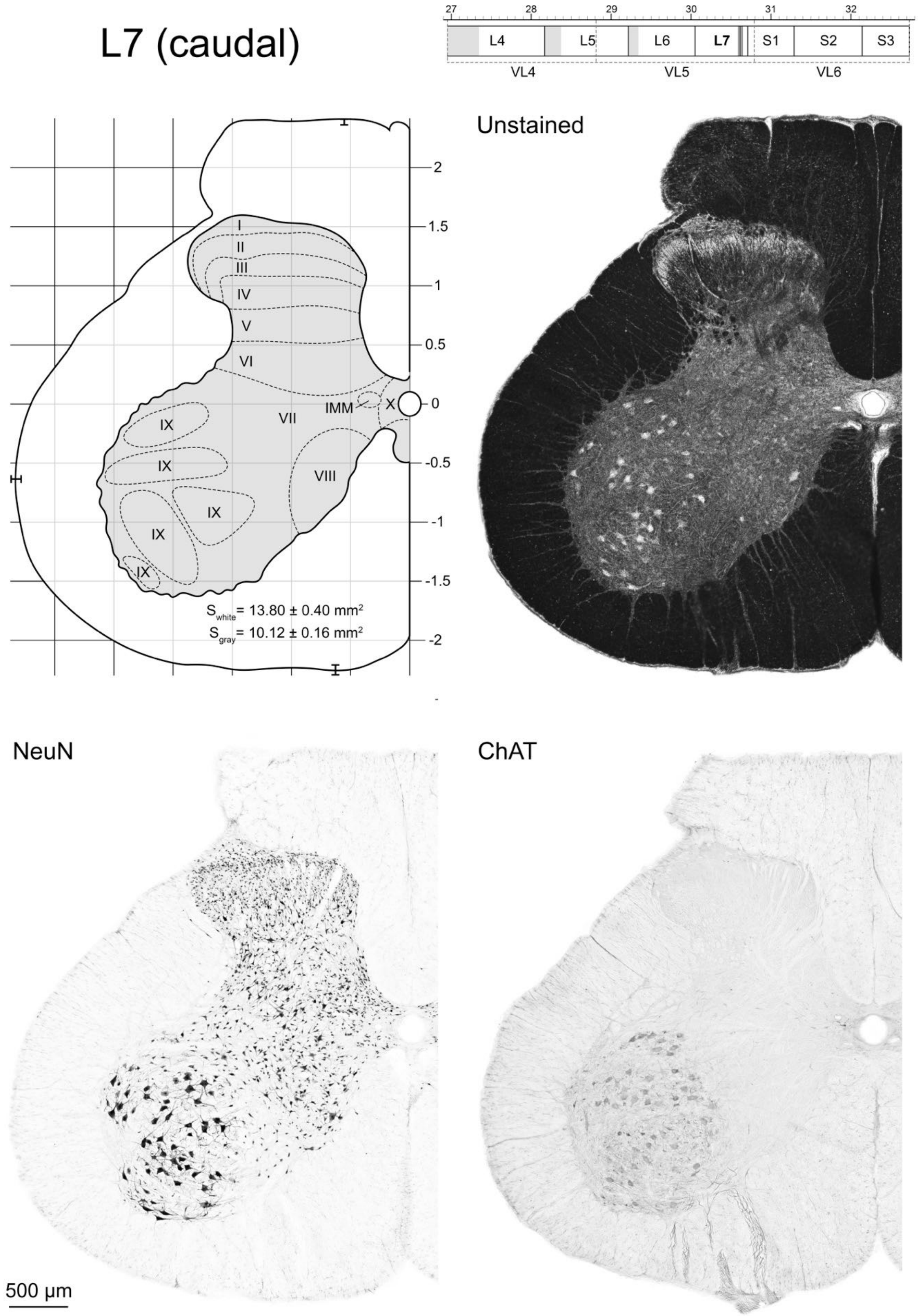

Supplementary Figure 18. Caudal part of L7 segment of the cat spinal cord.

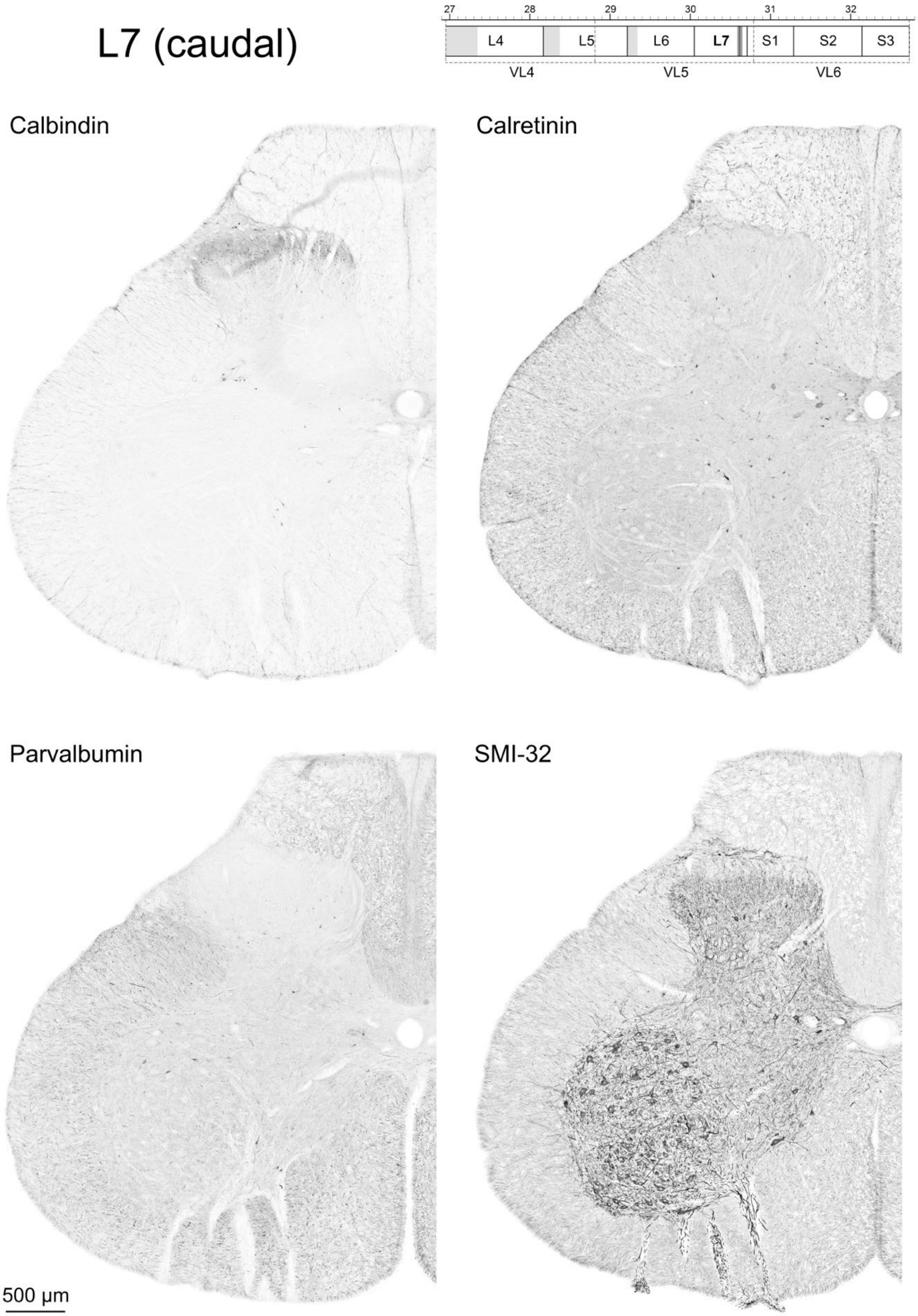

Supplementary Figure 18. Continued.
