# Supplementary material for: Neurochemical atlas of the cat spinal cord
Source: Front Neuroanat. 2022 Oct 19;16:1034395. doi: 10.3389/fnana.2022.1034395 (PMC9627295; doi:10.3389/fnana.2022.1034395)
Supplement: Supplementary file 5 [file Data_Sheet_5.PDF]

Supplementary Material

1 Abbreviations

I – lamina I

II – lamina II

III – lamina III

IV – lamina IV

V – lamina V

VI – lamina VI

VII – lamina VII

VIII – lamina VIII

IX – lamina IX

X – lamina X

CoN – Nucleus Commissuralis

DGC – Dorsal Gray Commissure

IMM – Intermediomedial Nucleus

S_{white} – area of the white matter

S_{gray} – area of the gray matter

2 Supplementary Figures

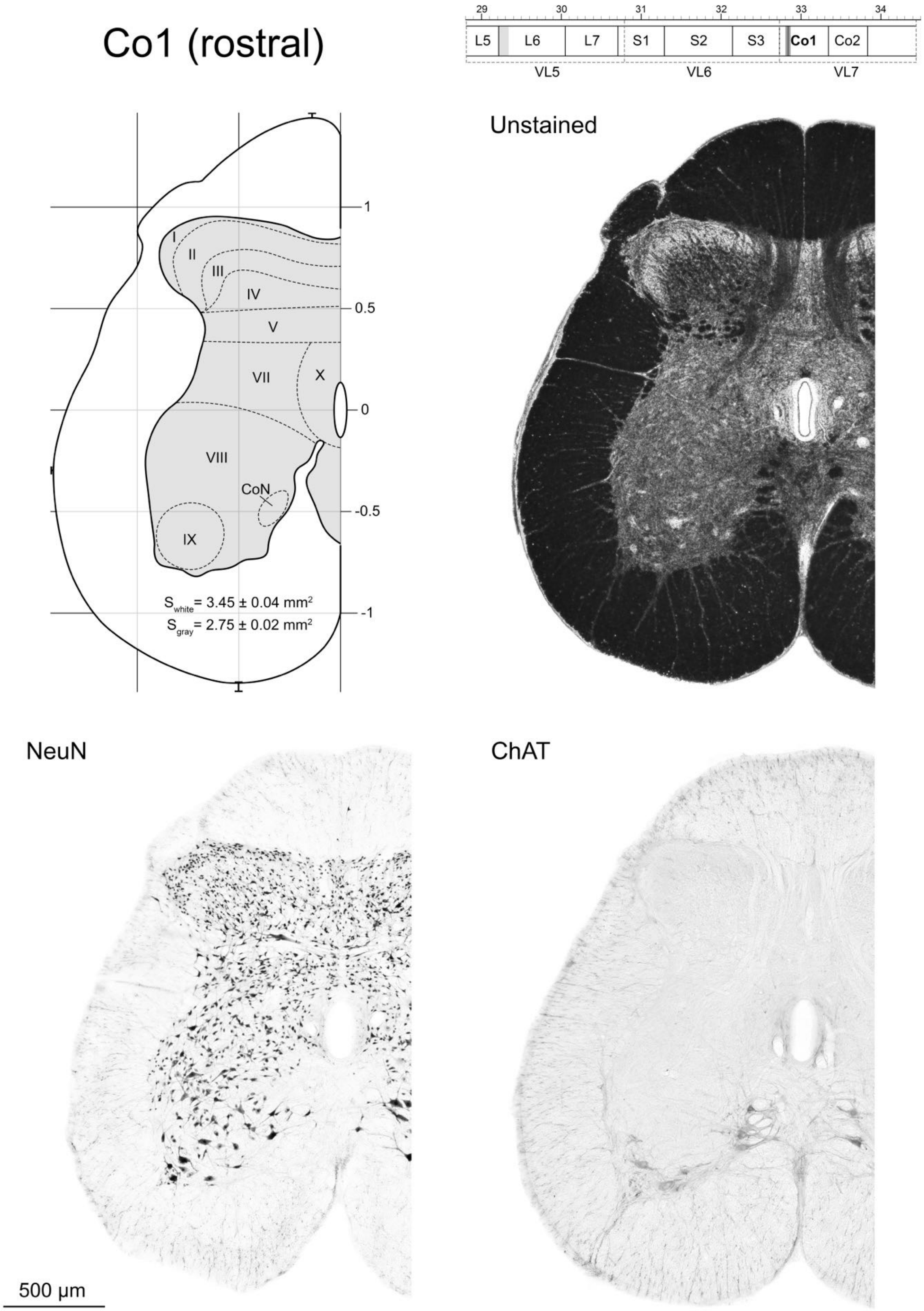

Supplementary Figure 1. Rostral part of Co1 segment of the cat spinal cord.

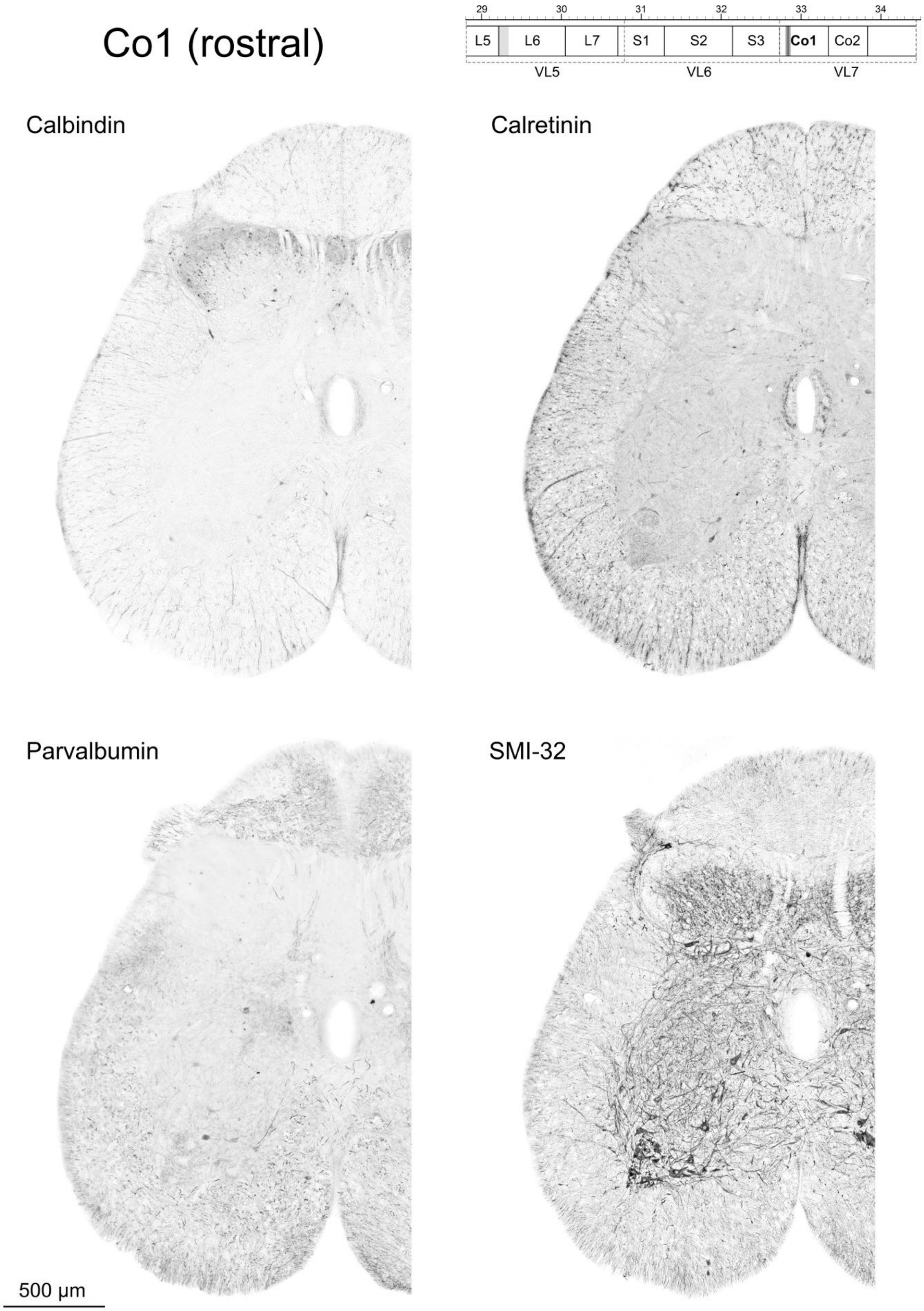

Supplementary Figure 1. Continued.

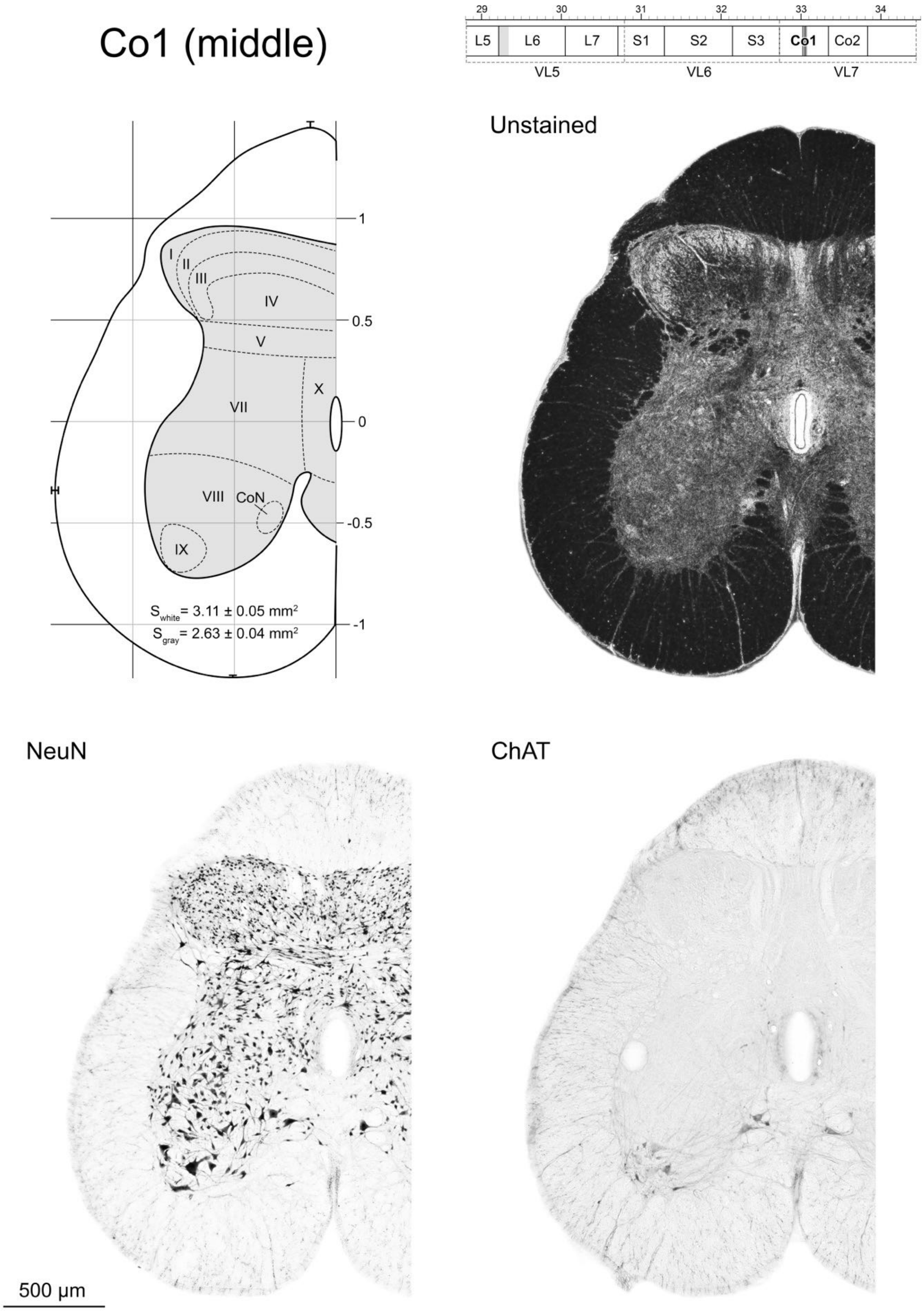

Supplementary Figure 2. Middle part of Co1 segment of the cat spinal cord.

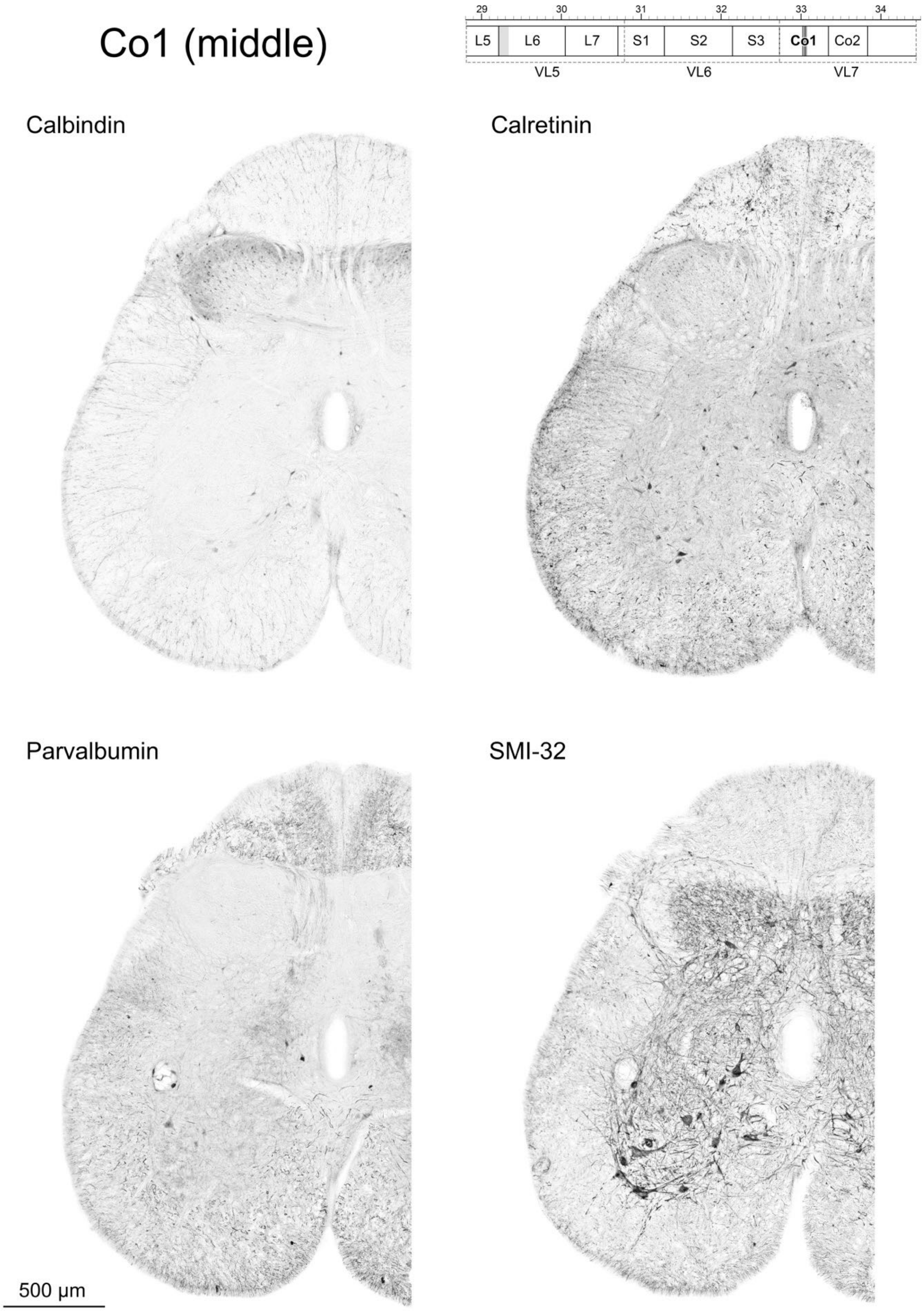

Supplementary Figure 2. Continued.

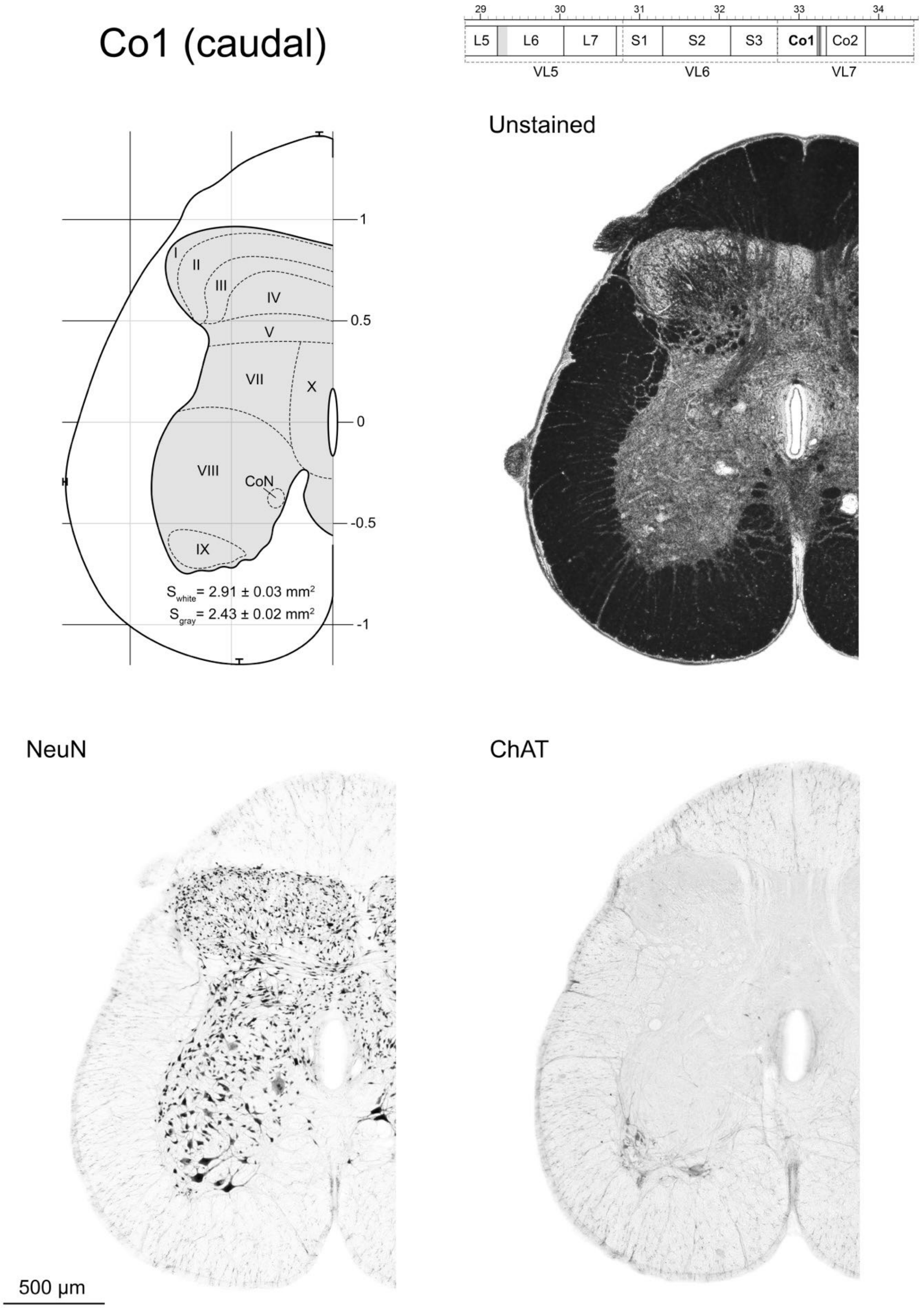

Supplementary Figure 3. Caudal part of Co1 segment of the cat spinal cord.

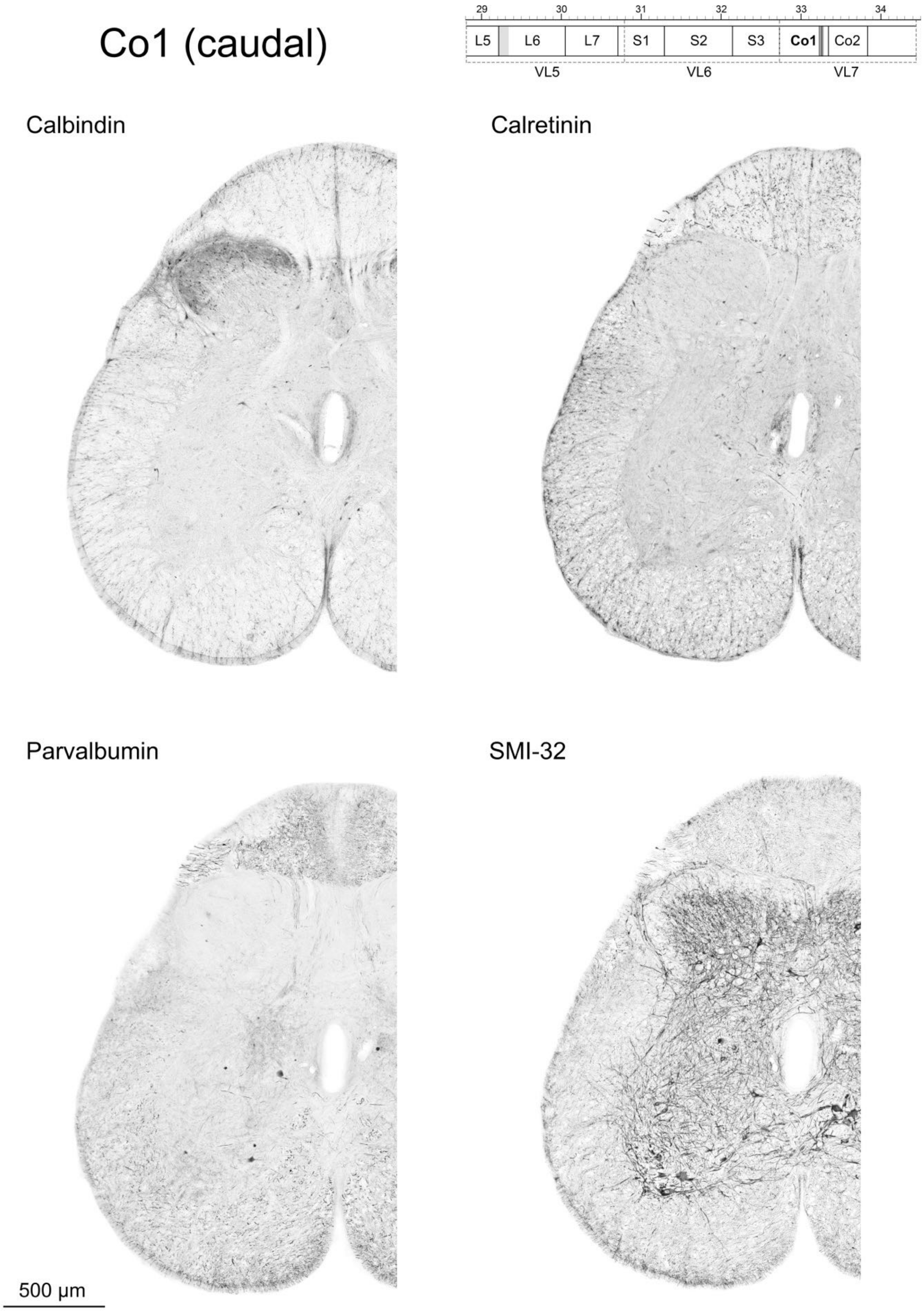

Supplementary Figure 3. Continued.

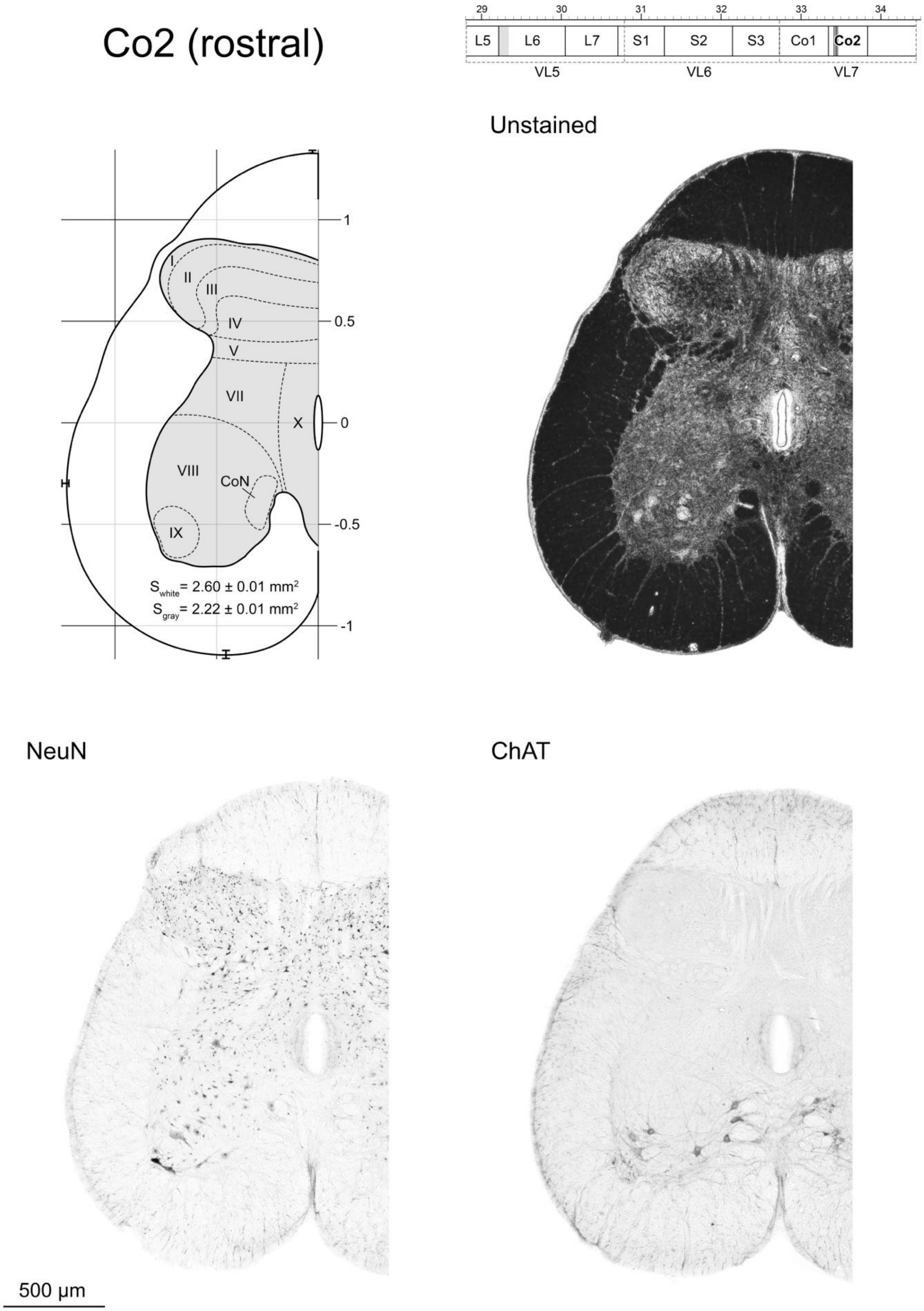

Supplementary Figure 4. Rostral part of Co2 segment of the cat spinal cord.

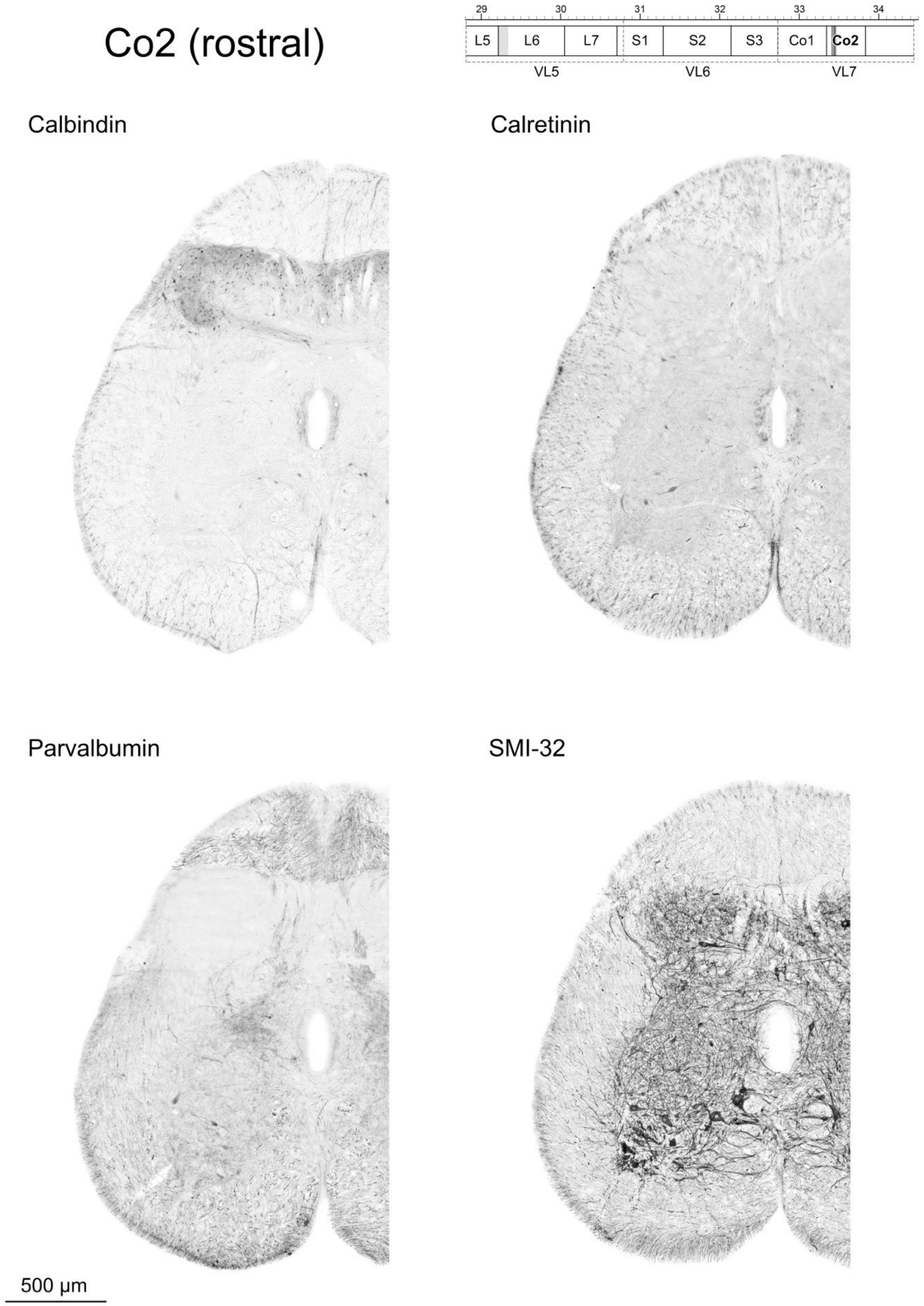

Supplementary Figure 4. Continued.

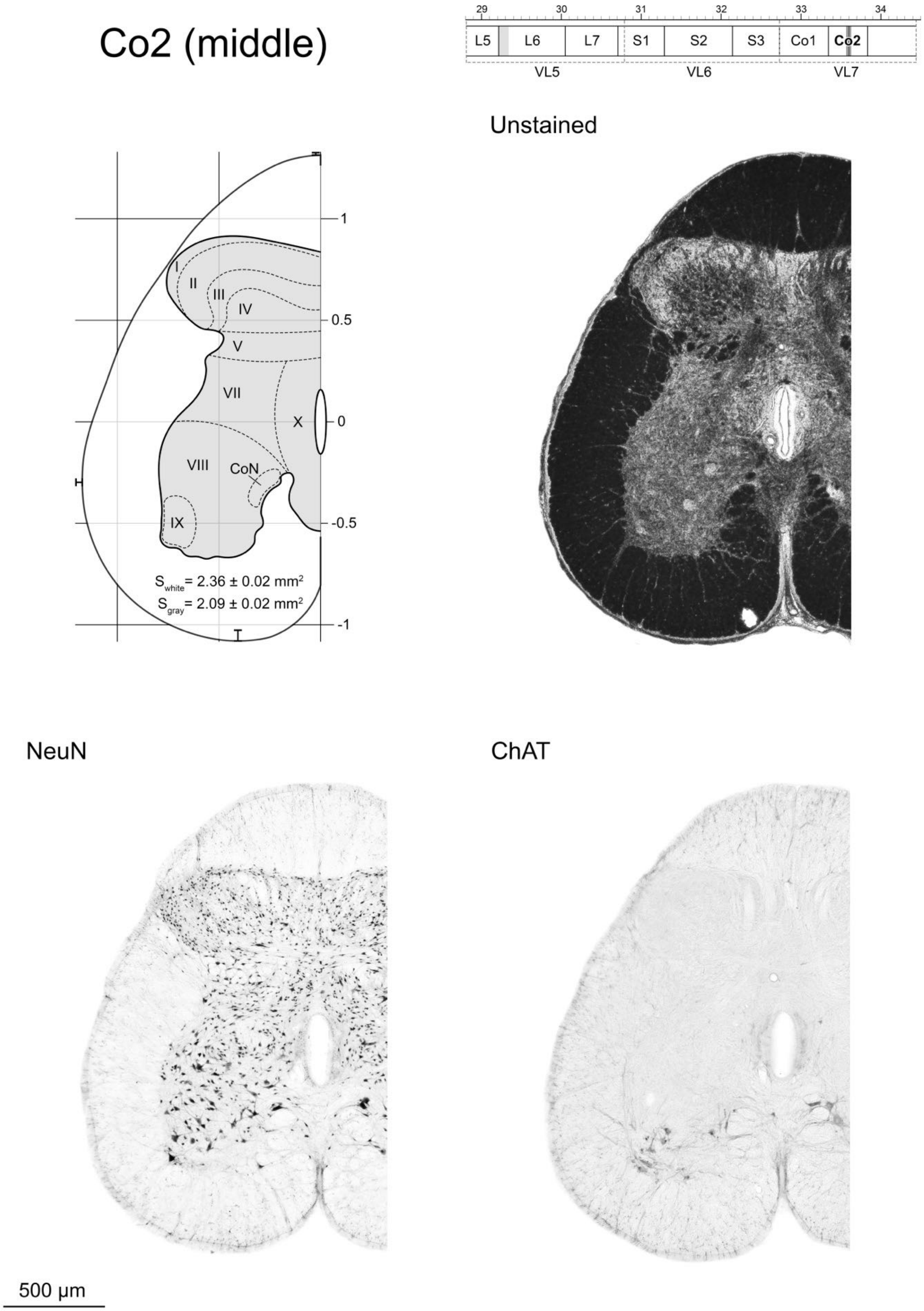

Supplementary Figure 5. Middle part of Co2 segment of the cat spinal cord.

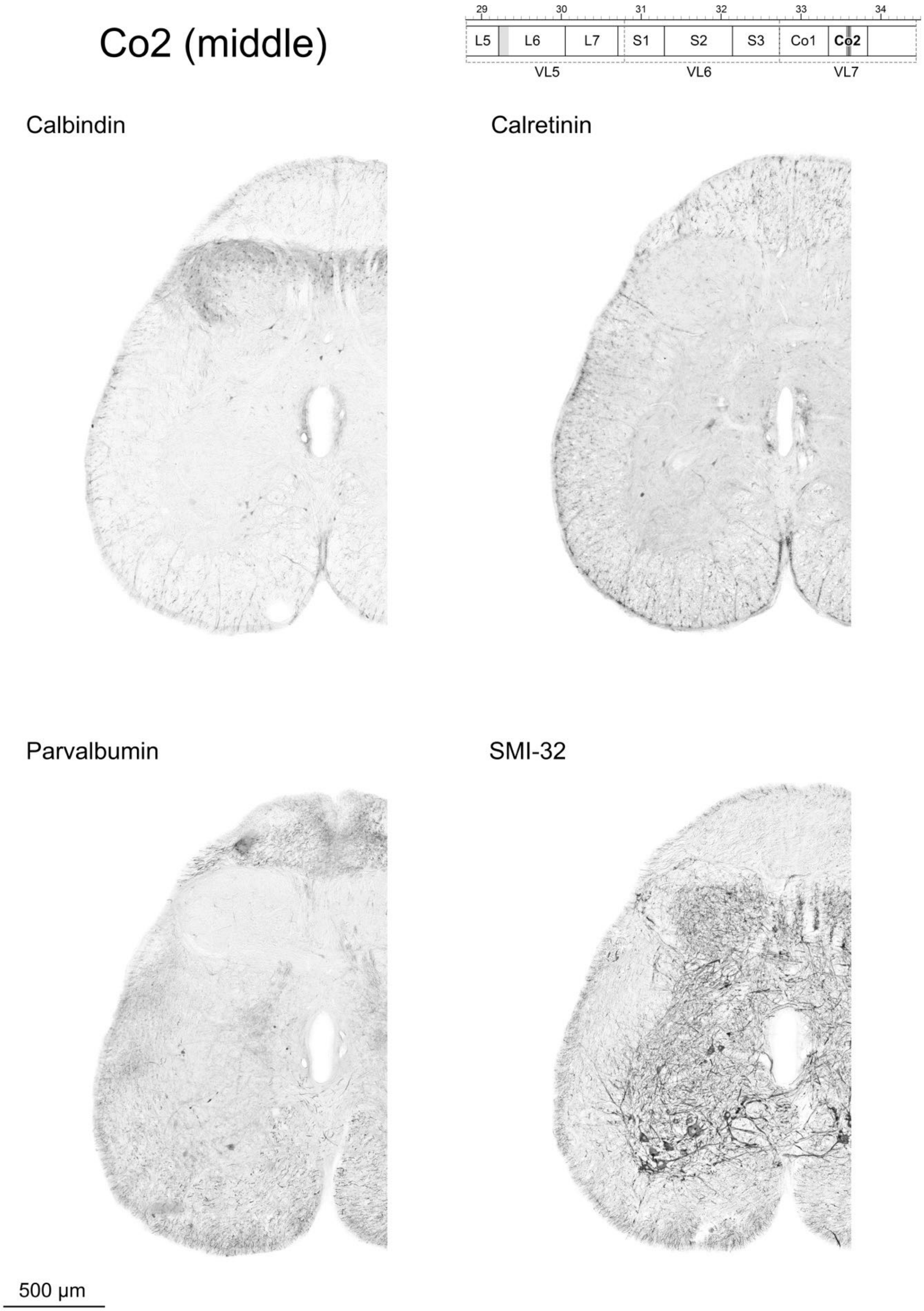

Supplementary Figure 5. Continued.

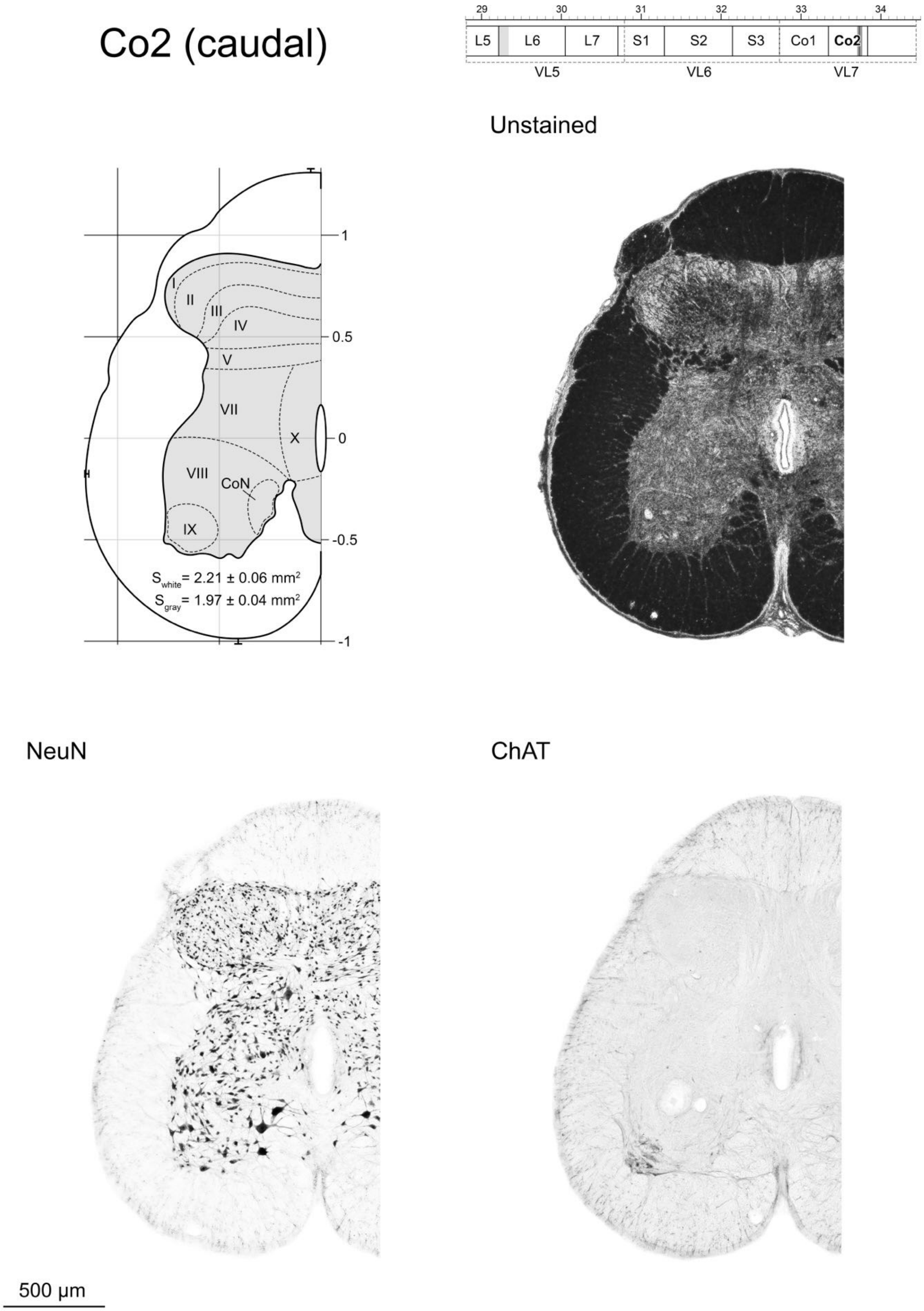

Supplementary Figure 6. Caudal part of Co2 segment of the cat spinal cord.

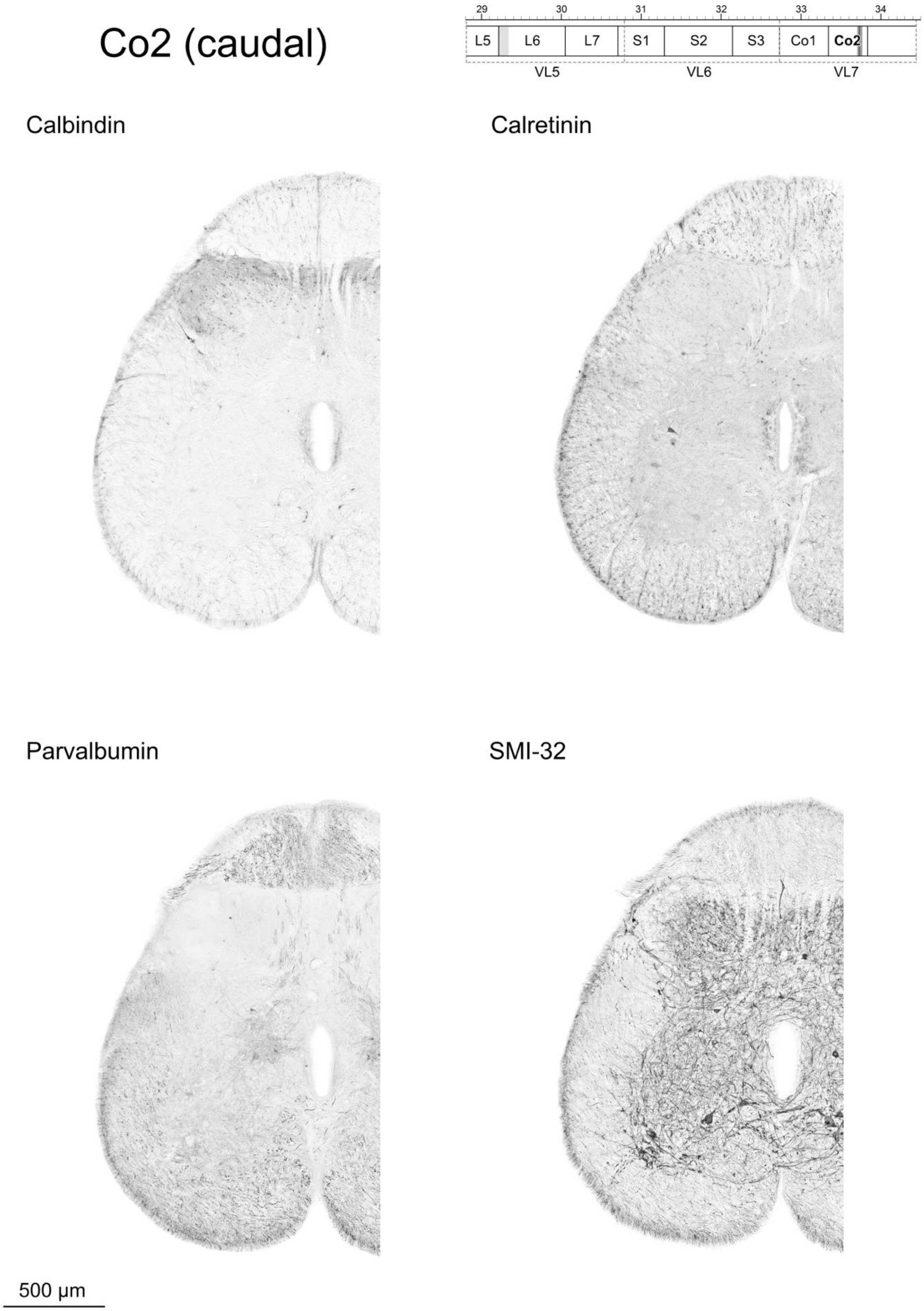

Supplementary Figure 6. Continued.
